# Patient education for neuromyelitis optica spectrum disorder using large language models: combining expert assessment and real-world patient interaction

**DOI:** 10.3389/fphys.2026.1923189

**Published:** 2026-08-19

**Authors:** Chen Li, Yuting Hu, Xiaoyan Wang, Ruoyi Yao, Jiahao Ye, Shanshan Diao, Yanhui Xiao, Yingming Wang, Mingying Lai, Weihua Yang

**Affiliations:** 1 Department of Ophthalmology, The First Affiliated Hospital of Soochow University, Suzhou, China; 2 Department of Ophthalmology and Optometry, Fujian Medical University, Fuzhou, China; 3 Department of Neurology, The First Affiliated Hospital of Soochow University, Suzhou, China; 4 Shenzhen Eye Hospital, Shenzhen Eye Medical Center, Southern Medical University, Shenzhen, China

**Keywords:** large language model, NMOSD, central nervous system, artificial intelligence, health education

## Abstract

**Objectives:**

This study aimed to compare the educational performance of six mainstream LLMs for neuromyelitis optica spectrum disorder (NMOSD) and evaluated patient satisfaction during real-world interactions.

**Methods:**

This study was conducted from March to April 2026. In the first Phase, Twenty NMOSD-related questions derived from clinical guidelines and patient concerns were submitted to six LLMs (ChatGPT-5.4, Gemini-3.1-pro, Claude-4.6-Sonnet, DeepSeek-3.2, Kimi-2.5, and Qwen-3.5-plus). Responses were anonymized and independently evaluated by three neuro-ophthalmology specialists using Likert framework assessing accuracy, completeness, readability, safety, and humanity. Inter-rater reliability was assessed using the intraclass correlation coefficient (ICC). In the second Phase, the three best-performing models were subsequently evaluated through real-world interactions with ten NMOSD patients, and satisfaction scores were analyzed using linear mixed-effects models.

**Results:**

A total of 120 chatbot responses were evaluated. With a comprehensive evaluation, significant differences were observed across all assessment domains. Gemini-3.1-pro achieved the highest scores for accuracy and safety, while Qwen-3.5-plus demonstrated superior completeness and humanity. DeepSeek-3.2 generated the most accessible responses, exhibiting the lowest reading difficulty score. However, its completeness advantage should be interpreted with caution, as it may be partially influenced by its longer response length. Inter-rater reliability was good, with single-measure ICC values ranging from 0.535 to 0.759, and average-measure ICC values ranging from 0.775 to 0.904. In patient interactions, Qwen-3.5-plus achieved the highest satisfaction score, significantly outperforming Gemini-3.1-pro and DeepSeek-3.2. Although all LLMs demonstrate superior performance in patient education, the real-world interaction with patient needs to pay attention.

**Conclusions:**

LLMs demonstrate considerable potential for NMOSD patient education but exhibit variability across educational dimensions, and require further validation in larger cohorts. These findings highlight the importance of selecting LLMs according to specific patient education goals and underscore the importance of clinicians in rare disease counseling.

## 1 Introduction

Large language models (LLMs), a class of advanced artificial intelligence (AI) systems, are designed for natural language understanding, processing and generation (Betzler et al., 2023; Shool et al., 2025). The release of ChatGPT by OpenAI in November 2022 marked a significant breakthrough in natural language processing, and a variety of LLMs have subsequently emerged, offering broader capabilities, enhanced accuracy and greater precision (Shao et al., [[NoYear]]; Xu et al., 2024; Cao et al., 2025; Huang et al., 2025; Yuan et al., 2025). With their rapid development and integration in human life, these systems offer broad prospects for enhancing patient education and healthcare quality especially in medicine fields including ophthalmology (Wong et al., 2024; Yaghy et al., 2024; Li et al., 2025; Zhang et al., 2025).

Neuromyelitis optica spectrum disorder (NMOSD) is an autoimmune-mediated inflammatory demyelinating disease of the central nervous system, primarily affecting the optic nerves and spinal cord. It is characterized by frequent relapse, severe disability, and strong predilection in young and middle-aged female. Moreover, the clinical manifestation varies substantially among individuals and progress rapidly, which severely impaired vision, nervous system and overall quality of life (Bilodeau et al., 2026; Bringel et al., 2026). Effective management of NMOSD, therefore, requires not only accurate and timely diagnosis but also comprehensive patient education and sustained medical support.

NMOSD is a rare disease, as the diagnosis and management of NMOSD require cooperation between neurological and ophthalmic specialists, patients in remote or underserved areas often face substantial barriers to accessing specialized care, which may adversely affect long-term disease treatment and quality of life (Reddy and Paulus, 2025; Wei et al., 2025). Therefore, there is an urgent need for accurate, accessible, and sustained patient education for future patient management. The rapid evolution of LLMs provide a promising tool for medical information delivery and patient support (Bahir et al., 2025; Wei et al., 2025; Huang et al., 2026). Although recent studies have shown that LLM-generated responses can perform comparably to clinicians in terms of content quality, empathy, and safety in certain common disease, concerns remain regarding factual accuracy, completeness, and misinformation, limiting their reliability for standardized self-management (Kalyan et al., 2022; Ayers et al., 2023; Potapenko et al., 2023; Shen et al., 2023; Tailor et al., 2024).

To address this gap, this study systematically evaluated the performance of LLMs in generating health education content for Chinese patients with NMOSD. By integrating specialist grading and preliminary patient interaction assessments, this study provides a practical evaluation of LLMs and their potential application in rare disease education.

## 2 Methods

### 2.1 Study design

**Figure 1:**
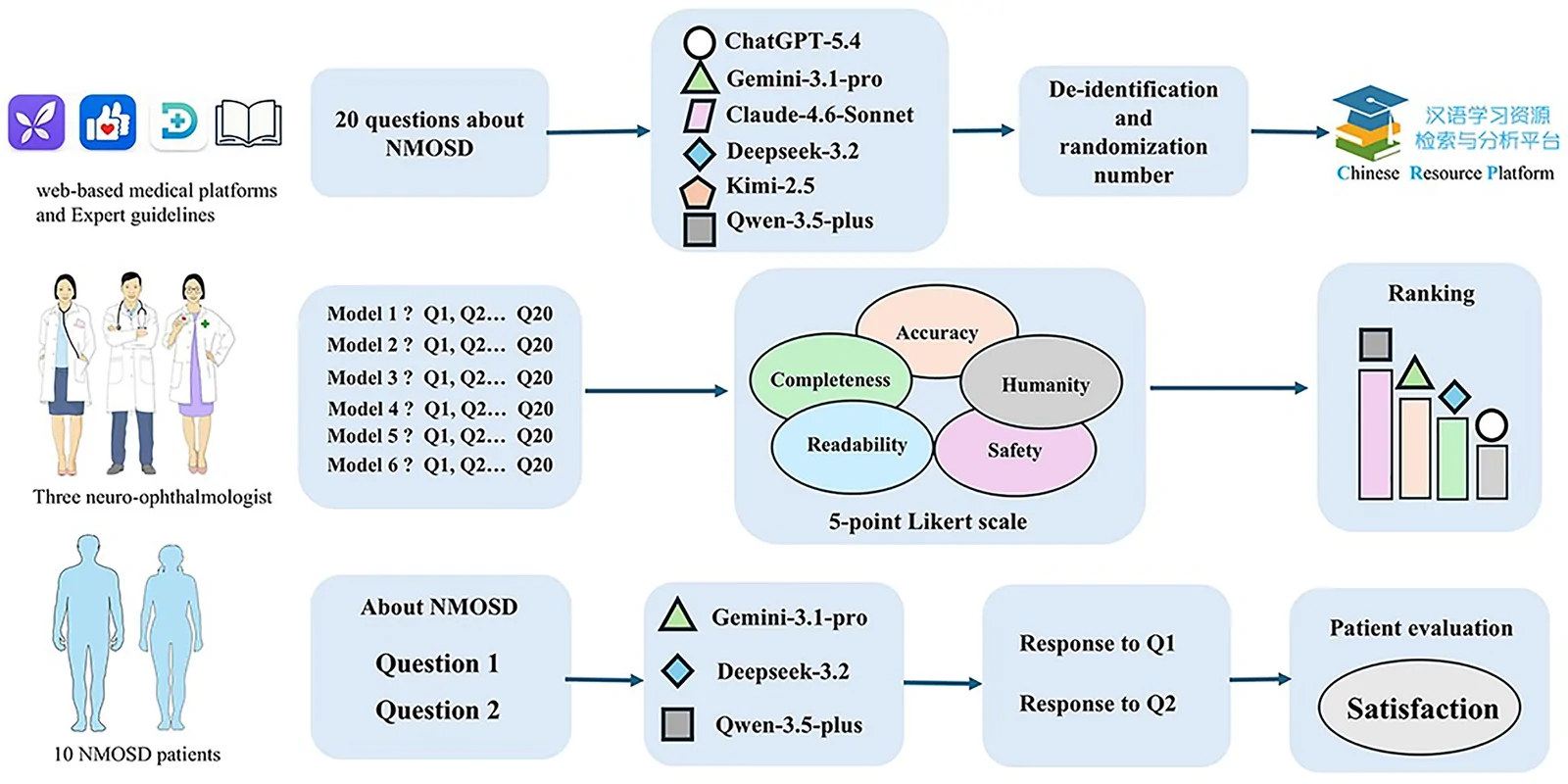
Research design workflow diagram.

This study was conducted from March 15 to April 15, 2026. Employing a combined quantitative and qualitative approach, we aimed to evaluate the effectiveness and safety of LLMs in patient education for Chinese individuals with NMOSD. By comparing the response performance of six different LLMs and integrating both expert ratings and patient feedback, we explored their practical application in the patient education process. The detailed workflow is illustrated in **Figure 1**.

### 2.2 Study subjects

Based on accessibility and model diversity, we compared six representative, publicly accessible mainstream LLMs (including, but not limited to, the OpenAI series, Google series, Anthropic series, and leading Chinese domestic models). The specific model versions and parameter settings are detailed in **Table 1**.

**Table 1:** Study model.

Model	Version	Exact model identifier	Access date	Access method	API endpoint	Web/retrieval-enhanced generation
ChatGPT	V5.4	gpt-5.4	March15-16, 2026	Web Interface	chat.openai.com	Disabled
Gemini	V3.1-pro	gemini-3.1-pro	March15-16, 2026	API (Google AI Studio)	Google AI Studio API	Disabled
Claude	V4.6-Sonnet	claude-4.6-sonnet	March17, 2026	Web Interface	Poe.com	Disabled
Deepseek	V3.2	deepseek-chat-v3.2	March15, 2026	Web Interface	chat.deepseek.com	Disabled
Kimi	V2.5	moonshot-v2.5	March18, 2026	Web Interface	kimi.moonshot.cn	Disabled
Qwen	V3.5-plus	qwen-3.5-plus	March18, 2026	API (Tongyi Qianwen)	Tongyi Qianwen API	Disabled

ChatGPT-5.4 was accessed via web interface (chat.openai.com) on March 15–16, 2026, with “Custom Instructions” disabled and “Temporary Chat Mode” enabled; Gemini-3.1-pro was accessed via Google AI Studio API on March 15–16, 2026; Claude-4.6-Sonnet was accessed via Poe.com on March 17, 2026; Deepseek-3.2 was accessed via official web chat (chat.deepseek.com) with Deep Thinking mode OFF on March 15, 2026; Kimi-2.5 was accessed via kimi.moonshot.cn on March 18, 2026; Qwen-3.5-plus was accessed via Tongyi Qianwen API on March 18, 2026. All interactions were conducted in Simplified Chinese. Web/Retrieval-Enhanced Generation was set to Disabled for all models to ensure that responses were generated solely based on the models’ internal knowledge without external information retrieval. For API-accessible models, temperature was set to 0, and where supported, random seed was fixed at 20260315 to ensure reproducibility.

### 2.3 Phase one

#### 2.3.1 Model parameter configuration and reproducibility settings

For each model, we recorded the following parameters to ensure reproducibility: system prompt, user prompt, temperature (set to 0 for all models), top_p (where applicable), max_tokens, seed (fixed at 20260315 for models supporting this parameter), conversation context handling (new session for each question), and whether web browsing or retrieval-augmented generation was enabled (all set to Disabled). For web-accessed models where API parameter fine-tuning was unavailable (e.g., ChatGPT web interface), we disabled “Custom Instructions” and enabled “Temporary Chat Mode” to minimize context interference. The exact model version strings and API endpoints are detailed in **Table 1**. All data collection was completed within a centralized 4-day window from March 15 to March 18, 2026, to minimize bias introduced by silent backend model updates.

#### 2.3.2 Questionnaire design

We collected and aggregated frequently asked questions from NMOSD patients over the preceding two years through several online medical platforms, including “DingXiangYuan,” “HaoDF” and “Baidu Health Ask a Doctor” Concurrently, we referenced the Chinese Guidelines for the Diagnosis and Treatment of NMOSD (2025 Edition),Wei et al., 2025 published by the Chinese Medical Association’s Neurology Branch, to identify core concerns in NMOSD patient education. Based on these data, we compiled 20 targeted questions which compromise Disease Diagnosis, Acute Episode and Treatment, Long-term Prevention, Medication Selection, Symptom Management, Disease Prognosis, Lifestyle Management and Psychological Support. We initially screened 35 candidate questions by aggregating and deduplicating approximately 150 FAQs from three online platforms, combined with the core educational priorities defined in the 2025 Chinese NMOSD guidelines. The three expert panel members independently rated each candidate question on a 1–5 scale based on clinical relevance, patient education need, and coverage of core NMOSD knowledge domains. The final 20 questions were selected based on a consensus score of ≥4 from all three experts, and the draft set was subsequently reviewed and confirmed by the same panel to ensure content validity, representativeness, and clinical relevance, with the final set representing a consensus among the panel. The questions’ detail is in **Supplementary Table 1**.

To enhance the professionalism and specificity of the LLMs’ responses and ensure they addressed questions from a defined perspective, we prepended a standardized prompt before each model’s output generation. The prompt read: “Please answer the patients’ questions about neuromyelitis optica spectrum disorder (NMOSD) as a neuro-ophthalmologist.” The 20 questions were subsequently input into the six different LLMs in Chinese, the output materials were also set in Chinese. Prior to formal evaluation, all model-generated responses were de-identified by removing model names and other identifiable source information, and the display format was standardized. All 120 responses were then assigned random numbers. An objective readability analysis was performed using the “Chinese Language Learning Resource Retrieval and Analysis Platform” (http://120.27.70.114:8000/index) to assess the text reading difficulty of the LLMs’ responses.

#### 2.3.3 Expert evaluation

Three medical experts were selected from the Departments of Ophthalmology and Neurology at the First Affiliated Hospital of Soochow University (**Table 2**). The expert panel comprised two ophthalmologists (specializing in neuro-ophthalmology) and one neurologist (specializing in neuro-ophthalmology). Prior to the formal evaluation, all experts participated in a standardized training session and received a structured evaluation manual containing detailed descriptions of each rating dimension, illustrative examples, and guidelines for response interpretation. These measures were designed to promote a consistent understanding of the assessment framework and reduce inter-rater variability. The experts completed their evaluations independently and were instructed not to communicate with one another during the rating process. All three medical experts independently rated the full set of 120 responses while blinded to the source model of each answer, and their ratings remained concealed from one another throughout the process. To mitigate order effects, the sequence of responses each evaluator received was randomized. Inter-rater agreement was assessed using the intraclass correlation coefficient (ICC).

**Table 2:** Expert profiles.

Department	Expert	Degree	Title	Major field	Years of working
Ophthalmology	Expert 1	MD	Chief physician	Neuro-ophthalmology	18
Ophthalmology	Expert 2	MD	Attending physician	Neuro-ophthalmology	11
Neurology	Expert 3	MD	Associate chief physician	Neuro-ophthalmology	13

The outputs of the six LLMs were evaluated using a 5-point Likert scale across five dimensions: Accuracy, Completeness, Readability, Safety, and Humanity. (1) Accuracy: Assesses whether the information generated by the model aligns with scientific knowledge and established medical facts. (2) Completeness: Evaluates whether the model’s response comprehensively addresses all relevant aspects of the question or clinical scenario. (3) Readability: Determines whether the model’s language is clear, concise, and easy to understand. (4) Safety: Investigates whether the model provides safe and appropriate advice without posing potential risks. A score of 1 is defined as “The response contains unsafe health advice or content that may mislead the patient”; a score of 5 is defined as “The response is completely safe, with no potential risks.” (5) Humanity: Evaluates whether the model fully considers the patient’s emotional needs, dignity, and individual differences when providing care and support. If an expert had concerns regarding any advice, they were permitted to briefly explain their reservations in a comments section. The detailed definitions of the 5-point Likert scale are provided in **Supplementary Table 2**. The expert rating data are presented in full in **Supplementary Table 3**.

### 2.4 Phase two

Ten hospitalized patients with NMOSD were randomly selected from the First Affiliated Hospital of Soochow University. The three best-performing models from Phase One were selected to interact with the patients in a real-world clinical setting. Each patient posed two NMOSD-related questions to the assigned chatbots. Prior to the interaction, a general prompt was input to establish the context: “Please assist a neuro-ophthalmologist in educating a patient with NMOSD.” The patients then independently rated their satisfaction using a satisfaction scale (**Supplementary Table 4**). Patient questions and detailed rating data are presented in **Supplementary Tables 5**, **S6**, respectively.

The inclusion and exclusion criteria were as follows:

#### 2.4.1 Inclusion criteria

1. Patients with a confirmed diagnosis of NMOSD meeting the diagnostic criteria outlined in the Chinese Guidelines for the Diagnosis and Treatment of NMOSD (2025 Edition).
2. Native Mandarin speakers with basic communication skills, capable of accurately expressing their needs.
3. Aged 18 years or older, with no restriction on sex.
4. Voluntary participation in the study with signed written informed consent.
5. Special inclusion provision: Patients with severely impaired vision preventing them from reading on-screen text could be enrolled through a physician-assisted voice interaction mode, as determined by the investigator, to ensure the right to participation for visually impaired individuals. A standardized procedure was used for all voice-assisted interactions, including a pre-defined script, verbatim reading without paraphrasing, and a single designated research coordinator to perform all such interactions.

#### 2.4.2 Exclusion criteria

1. Patients unable to complete the conversational assessment due to severe cognitive impairment (e.g., dementia, acute episode of psychiatric illness), aphasia, or disturbance of consciousness.
2. Patients unable to cooperate with the study procedures due to critical illness (e.g., acute myelitis requiring absolute bed rest).
3. Patients deemed by the investigator to have other conditions rendering participation inappropriate (e.g., severe hearing impairment preventing voice-based interaction).

### 2.5 Patient safety protection

All Phase Two LLM-patient interactions were conducted with a trained research coordinator present and a neuro-ophthalmologist on call. All LLM outputs were clinician-reviewed prior to patient viewing; unsafe or anxiety-provoking responses were excluded and replaced with clinician-verified content (no such events occurred). Patients were informed verbally and in writing that the responses were AI-generated, not medical advice, and should not replace their physicians’ recommendations. All patients received a standard debriefing with a clinician following the interaction.

### 2.6 Statistical analysis

All statistical analyses were performed using SPSS version 26.0 (IBM Corp., Armonk, NY, USA) and R software version 4.2.0 (R Foundation for Statistical Computing, Vienna, Austria). Continuous variables are presented as mean ± standard deviation (SD), and categorical variables are expressed as frequencies and percentages.

In Phase One, one-way analysis of variance (ANOVA) was employed to compare the overall differences among the six LLMs in reading difficulty scores, Chinese character counts, recommended reading ages, and expert rating scores across all five dimensions (accuracy, completeness, readability, safety, and humanity). Homogeneity of variances was assessed using Levene’s test; Welch’s correction was applied when the assumption of equal variances was violated. When ANOVA results were significant, *post-hoc* pairwise comparisons were conducted using Dunn’s test with Bonferroni correction.

Inter-rater reliability was assessed using the ICC based on a two-way random-effects model with an absolute agreement definition. Both single-measure ICC and average-measure ICC values are reported. Internal consistency was quantified using Cronbach’s alpha coefficient. All ICC and alpha coefficients were tested for significance using the F-test.

In Phase Two, the analysis of patient interaction outcomes involved comparing patient satisfaction scores among the three candidate models using linear mixed-effects model (LMM), with model type included as a fixed effect and question included as a random effect to account for clustering of ratings within the same question. A total of 60 ratings (10 patients × 2 questions × 3 models) entered the LMM. The random-effect variance components were estimated for the question-level random intercept, and the residual variance was also calculated. Overall differences among models were assessed using Type III F-tests with Satterthwaite approximation. *Post hoc* pairwise comparisons were performed using estimated marginal means with Bonferroni correction. All statistical tests were two-sided, a *P*-value < 0.05 was considered statistically significant.

### 2.7 Ethical considerations

This study was approved by the Ethics Committee of the First Affiliated Hospital of Soochow University (approval number: No. 2026-459). All participants provided written informed consent prior to enrollment. To ensure privacy and confidentiality, all collected participant data were anonymized. No identifiable personal information is included in the manuscript or **Supplementary Materials**.

## 3 Results

### 3.1 Phase one: primary evaluation metrics

#### 3.1.1 Comparison of objective Chinese readability across LLM responses

**Figure 2:**
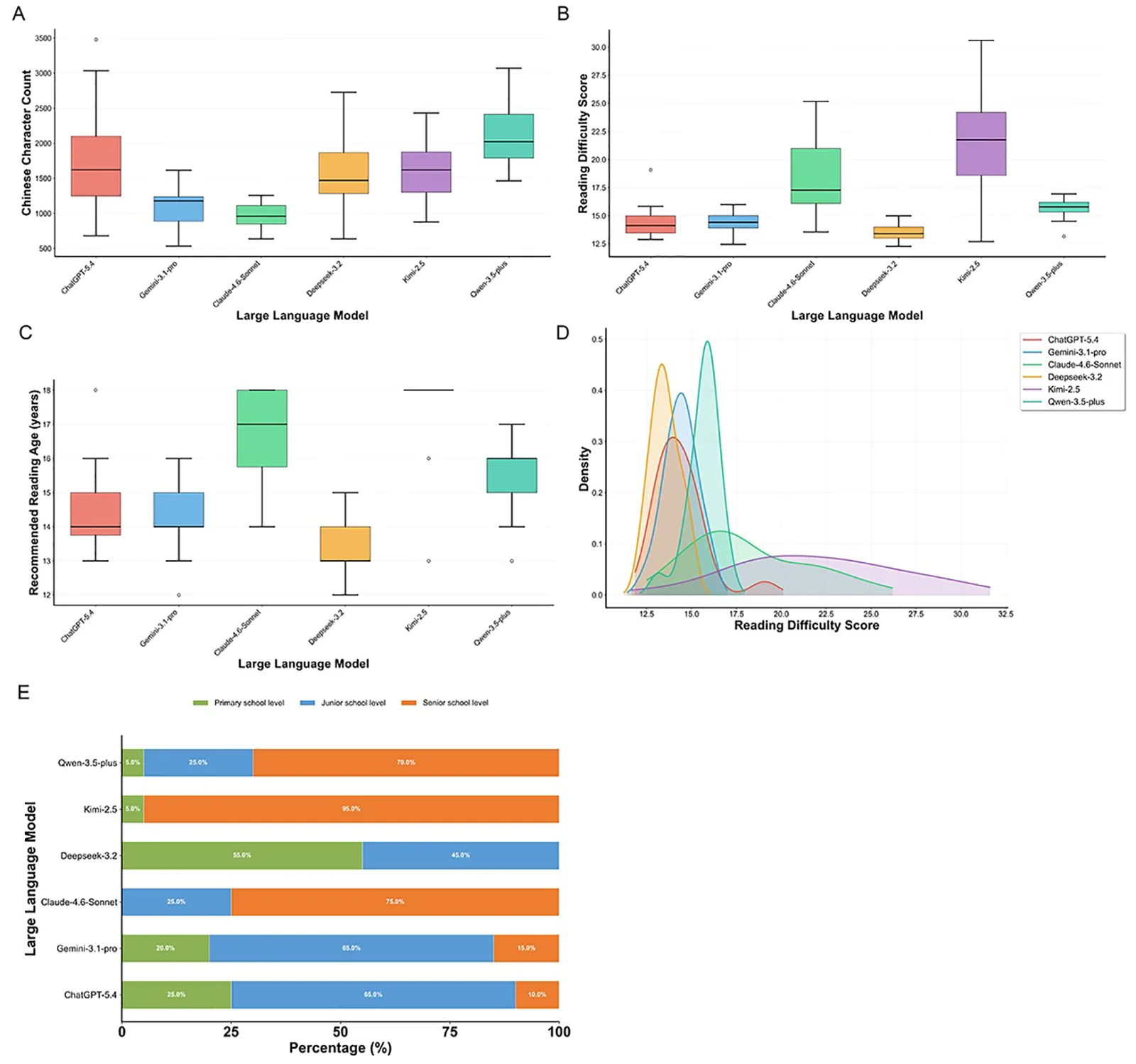
Comparison of Chinese readability metrics among six large language models. **(A–C)** Box plots illustrating the variation among the six LLMs in Chinese character count **(A)**, reading difficulty score **(B)**, and recommended reading age **(C)**. Each box represents the interquartile range, and the horizontal line within the box denotes the median. Outliers are indicated by open circles. **(D)** Density plots illustrating the distribution of reading difficulty scores for each model. Different colors denote different models, with the horizontal axis representing the reading difficulty score and the vertical axis representing density. **(E)** Grouped bar chart showing the distribution of educational levels required to comprehend the responses generated by each model. Educational levels are categorized according to recommended reading age as primary school (≤12 years), junior school (13–15 years), senior school (16–18 years). Numbers on the bars indicate the percentage accounted for by each category.

**Table 3** and **Figure 2** present the objective Chinese readability metrics forthe responses generated by the six LLMs. One-way ANOVA revealed statistically significant differences among the models across all three readability indicators (all *P* < 0.001). The mean reading difficulty scores ranged from 13.54 ± 0.78 (Deepseek-3.2) to 21.96 ± 4.49 (Kimi-2.5). Deepseek-3.2 achieved the lowest reading difficulty score, followed by ChatGPT-5.4 with a mean of 14.38 ± 1.40 and Gemini-3.1-pro (14.40 ± 0.91). In contrast, Kimi-2.5 exhibited the highest reading difficulty. The Chinese character counts ranged from 971.30 ± 182.21 (Claude-4.6-Sonnet) to 2126.45 ± 419.25 (Qwen-3.5-plus). The overall mean character count across all models was 1,516.98 characters. Deepseek-3.2 generated responses closest to this average length (1,535.35 ± 514.21), followed by Kimi-2.5 (1,624.25 ± 444.79). Claude-4.6-Sonnet produced the most concise responses, whereas Qwen-3.5-plus generated the most verbose output. The recommended reading ages ranged from 13.45 ± 0.83 years (Deepseek-3.2) to 17.65 ± 1.18 years (Kimi-2.5). Responses from Deepseek-3.2 were the most accessible, suitable for readers as young as 13.45 years. ChatGPT-5.4 ranked second, with a recommended reading age of 14.30 ± 1.22 years. Kimi-2.5 required the highest reading proficiency, with its responses suited for readers aged 17.65 years and older.

**Table 3:** Comparison of six LLMs across objective readability analysis (Mean ± SD).

Model	Reading difficulty score	Chinese character count	Recommended reading age (years)
ChatGPT-5.4	14.38 ± 1.40^d,e^	1750.30 ± 714.49^a,b^	14.30 ± 1.22^c,d,e^
Gemini-3.1-pro	14.40 ± 0.91^c,d^	1094.25 ± 299.67^e^	14.35 ± 1.09^c,d^
Claude-4.6-Sonnet	18.17 ± 3.18^a,b^	971.30 ± 182.21^e,f^	16.70 ± 1.42^a,b^
Deepseek-3.2	13.54 ± 0.78^d,e,f^	1535.35 ± 514.21^b,c,d^	13.45 ± 0.83^d,e,f^
Kimi-2.5	21.96 ± 4.49^a^	1624.25 ± 444.79^a,b,c^	17.65 ± 1.18^a^
Qwen-3.5-plus	15.66 ± 0.86^b,c^	2126.45 ± 419.25^a^	15.65 ± 0.93^b,c^
P value	<0.001	<0.001	<0.001

Different superscript letters (a, b, c, d, e, f) within the same column indicate statistically significant differences between models based on Dunn’s *post-hoc* test (adjusted P < 0.05).

#### 3.1.2 Inter-rater reliability

**Figure 3:**
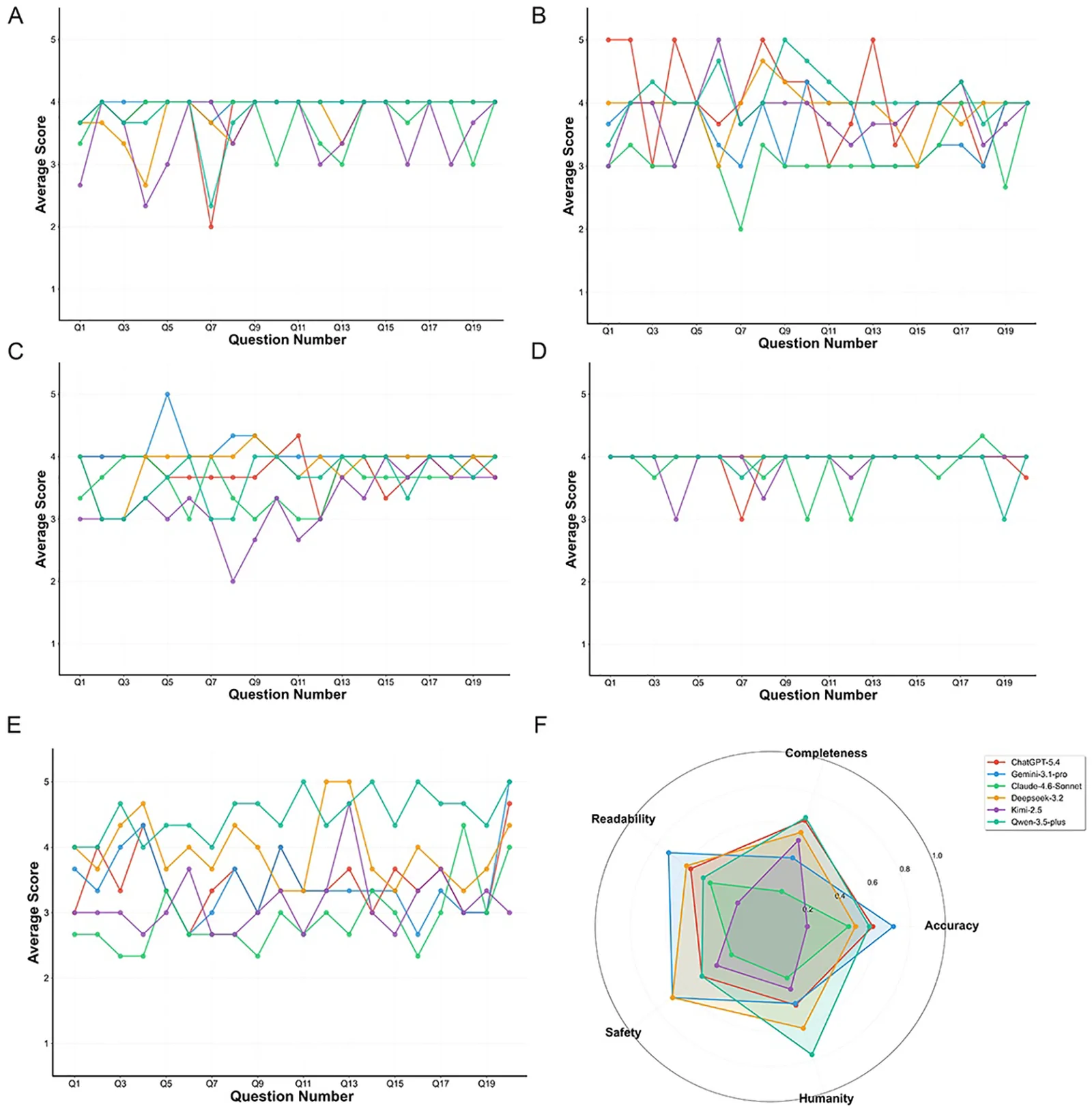
Performance comparison across the five evaluation dimensions. **(A–E)** Line graphs illustrating the score trajectories of the six LLMs across the 20 questions for the five dimensions: Accuracy **(A)**, Completeness **(B)**, Readability **(C)**, Safety **(D)**, and Humanity **(E)**. Each line represents a different model, with the horizontal axis denoting the question number and the vertical axis representing the mean score (on a 1–5 scale). Different colors denote different models and remain consistent across all subfigures. **(F)** Radar chart comparing the standardized performance of the six models across all five dimensions. The radar chart displays the standardized scores (on a 0–1 scale) for each dimension, with the outer boundary representing optimal performance. Each colored polygon represents a different model, facilitating intuitive comparison of overall performance profiles.

**Table 4** and **Figure 3** present the inter-rater reliability coefficients across the five evaluation dimensions for all LLM responses. Three independent raters assessed all model-generated responses, and reliability was evaluated using the ICC based on a two-way random-effects model with an absolute agreement definition. Inter-rater reliability was good, with single-measure ICC values ranging from 0.535 to 0.759 (indicating moderate to good reliability for individual raters), and average-measure ICC values ranging from 0.775 to 0.904 (all exceeding 0.75, indicating excellent reliability for the mean of the three raters. The average-measure ICC values across the five evaluation dimensions demonstrated good to excellent reliability, ranging from 0.775 to 0.904. With all average-measure ICC values exceeding 0.85, the reliability of the mean ratings from the three raters was excellent. This evaluation framework yielded reliable and consistent results across the independent raters (all *P* < 0.01), thereby supporting the validity of the subsequent comparative analyses. Cronbach’s alpha coefficients ranged from 0.788 to 0.906, indicating good to excellent internal consistency across all evaluation dimensions.

**Table 4:** Inter-rater reliability for five evaluation dimensions across all LLMs.

Metric	Accuracy	Completeness	Readability	Safety	Humanity
ICC (Single Measures)	0.640	0.759	0.576	0.678	0.535
ICC (Average Measures)	0.842	0.904	0.803	0.863	0.775
P value	<0.001	<0.001	<0.001	<0.001	<0.001
Cronbach’s Alpha	0.859	0.906	0.815	0.886	0.788

ICC. Intraclass correlation coefficient.

#### 3.1.3 Performance comparison of LLMs

Three experts evaluated the responses generated by the LLMs across five dimensions: Accuracy, Completeness, Readability, Safety, and Humanity. **Table 5** presents the performance comparison of the six LLMs across these dimensions. One-way ANOVA revealed statistically significant differences among the models in all five dimensions.

**Table 5:** Comparison of six LLMs across accuracy, completeness, readability, safety, and humanity (Mean ± SD).

Model	Accuracy	Completeness	Readability	Safety	Humanity
ChatGPT-5.4	3.87 ± 0.47^a,b^	4.07 ± 0.71^a,b^	3.82 ± 0.43^b,c^	3.93 ± 0.25^a,b,c^	3.47 ± 0.72^c^
Gemini-3.1-pro	3.97 ± 0.18^a^	3.60 ± 0.53^d,e^	4.08 ± 0.28^a^	3.98 ± 0.13^a^	3.43 ± 0.72^c,d^
Claude-4.6-Sonnet	3.75 ± 0.44 ^b,c,d,e^	3.18 ± 0.54^f^	3.58 ± 0.50^c,d,e^	3.85 ± 0.36^c,d,e,f^	2.90 ± 0.73^e,f^
Deepseek-3.2	3.78 ± 0.49^a,b,c,d^	3.92 ± 0.42^a,b,c^	3.87 ± 0.39^a,b^	3.97 ± 0.18^a,b^	3.95 ± 0.70^b^
Kimi-2.5	3.57 ± 0.56^e,f^	3.82 ± 0.54^a,b,c,d^	3.25 ± 0.60^f^	3.93 ± 0.25^a,b,c,d,e^	3.13 ± 0.72^c,d,e^
Qwen-3.5-plus	3.85 ± 0.44^a,b,c^	4.12 ± 0.49^a^	3.68 ± 0.47^b,c,d^	3.93 ± 0.25^a,b,c,d^	4.50 ± 0.57^a^
P value	<0.001	<0.001	<0.001	0.018	<0.001

Different superscript letters (a, b, c, d, e, f) within the same column indicate statistically significant differences between models based on Dunn’s *post-hoc* test (adjusted P < 0.05).

In terms of Accuracy, the mean scores ranged from 3.57 ± 0.56 (Kimi-2.5) to 3.97 ± 0.18 (Gemini-3.1-pro). Gemini-3.1-pro attained the highest accuracy score with the smallest standard deviation (0.18), indicating both superior and stable performance. ChatGPT-5.4 ranked second, with a mean score of 3.87 ± 0.47, followed by Qwen-3.5-plus (3.85 ± 0.44). Kimi-2.5 demonstrated the lowest accuracy performance among all models. For Completeness, scores ranged from 3.18 ± 0.54 (Claude-4.6-Sonnet) to 4.12 ± 0.49 (Qwen-3.5-plus). Qwen-3.5-plus exhibited markedly superior completeness, outperforming all other models. ChatGPT-5.4 ranked second in completeness (4.07 ± 0.71), albeit with greater variability. Claude-4.6-Sonnet demonstrated the lowest completeness performance and was significantly inferior to the leading models. With respect to Readability, Gemini-3.1-pro achieved the highest mean score of 4.08 ± 0.28. Deepseek-3.2 ranked second (3.87 ± 0.39), followed by ChatGPT-5.4 (3.82 ± 0.43). Kimi-2.5 had the lowest readability score (3.25 ± 0.60), with a notably larger standard deviation. Safety scores exhibited the smallest variability among all dimensions, ranging from 3.85 ± 0.36 (Claude-4.6-Sonnet) to 3.98 ± 0.13 (Gemini-3.1-pro). Gemini-3.1-pro garnered the highest safety score with minimal variability. Notably, models’ safety scores exceeded 3.85, indicating that under the current set of test questions, no mainstream LLMs generated high-risk hallucinations with clear clinical hazards. Although the above safety scoring results are encouraging, a clinically meaningful NMOSD patient education tool should not only avoid generating harmful information, but also correctly identify warning signs in acute scenarios and direct patients to urgent specialist evaluation. The question set evaluated in this study mainly covers general educational topics and does not adequately incorporate such high-risk acute scenarios; therefore, caution should be exercised when extrapolating the current safety conclusions to emergency or acute attack settings. Humanity scores also demonstrated substantial inter-model performance differences (*P* < 0.001). Qwen-3.5-plus demonstrated exceptional humanistic quality, with a mean score of 4.50 ± 0.57, significantly outperforming all other models. Deepseek-3.2 ranked second (3.95 ± 0.70), whereas Claude-4.6-Sonnet received the lowest humanity rating (2.90 ± 0.73).

### 3.2 Phase two: patient interaction outcomes

**Figure 4:**
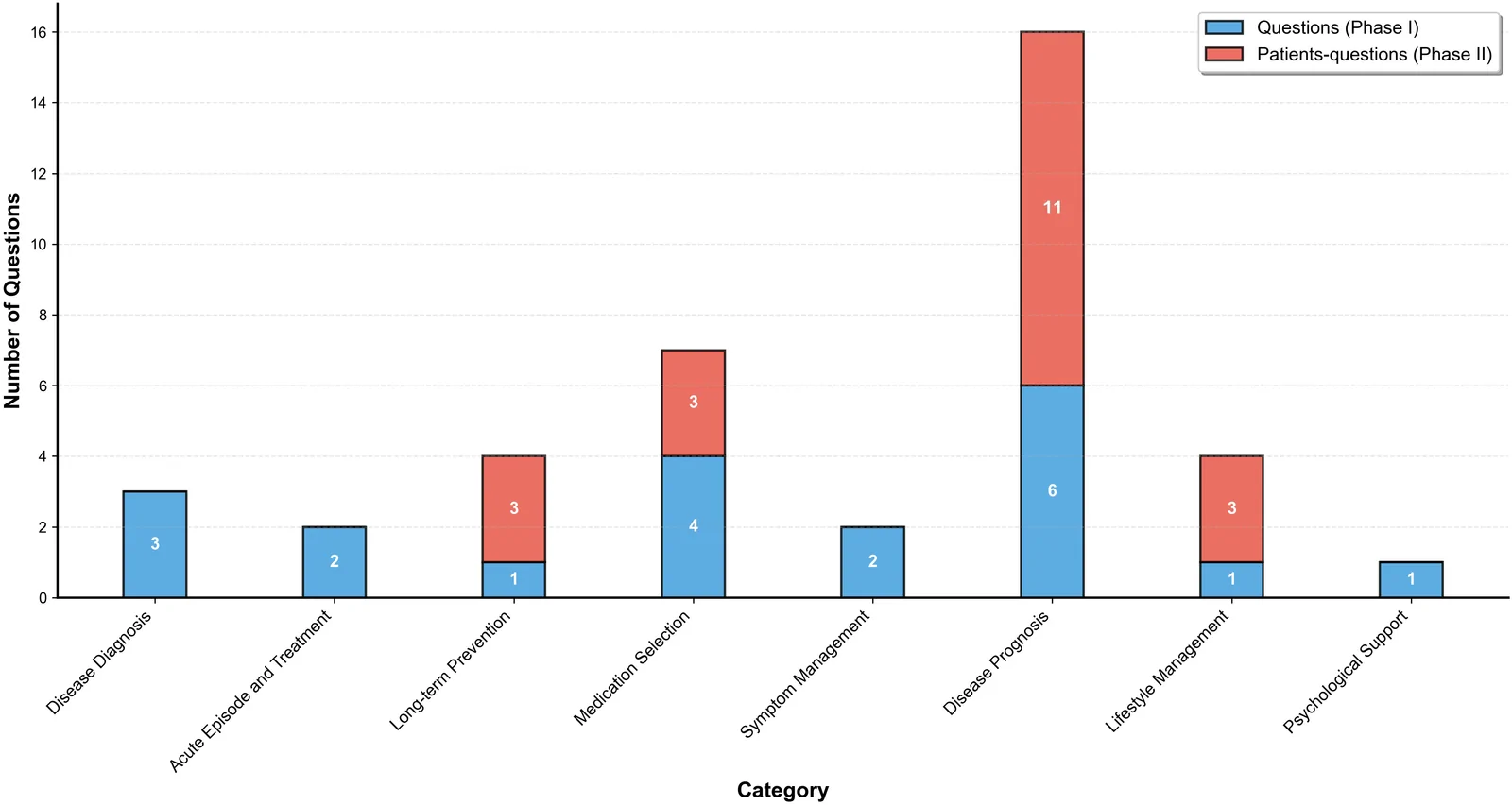
Distribution of question categories across the two study Phases. Grouped bar chart illustrating the classification of questions into eight categories across the two study Phases. Blue bars represent questions from Phase One, and red bars represent questions from Phase Two. Numbers on the bars indicate the count of questions within each category. The horizontal axis denotes the question category, and the vertical axis represents the number of questions.

A total of 10 eligible patients were recruited, generating 20 patient-initiated questions. A summary of patient demographic and clinical characteristics (including age, sex, disease duration, disease-modifying therapy status, and history of optic neuritis) is provided in **Supplementary Table 7**. Along with the questions generated in Phase one, all question along with the expert-suggested items, were subsequently classified into eight categories: disease diagnosis, acute attack and treatment, long-term prevention, medication selection, symptom management, disease prognosis, lifestyle management, and psychological support. Disease prognosis constituted the most dominant category in both Phases, accounting for 30.0% (n=6) of questions in Phase One and 52.6% (n=10) in Phase Two, representing 41.0% of all questions overall. This indicates that disease prognosis is the foremost concern among patients (**Figure 4**).

**Figure 5:**
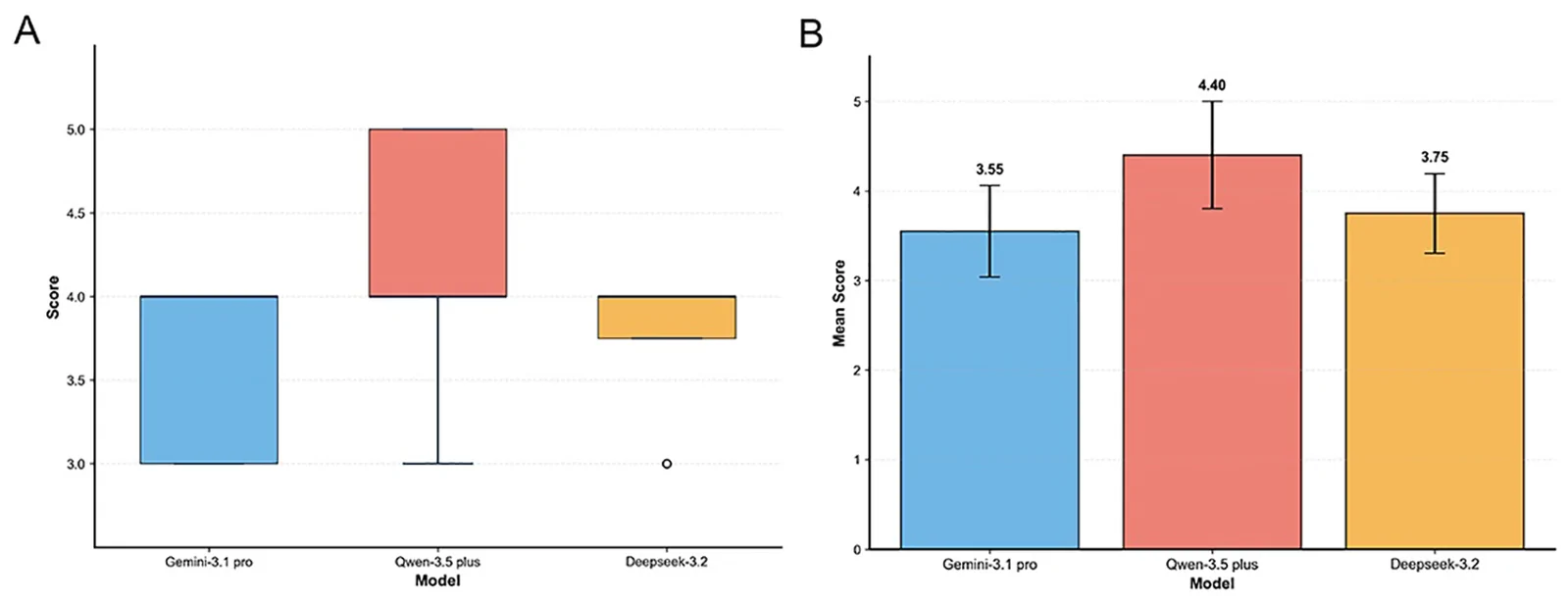
Comparison of patient evaluation scores among three large language models. **(A)** Box plot illustrating the distribution of patient evaluation scores for the three models (Gemini-3.1-pro, Qwen-3.5-plus, and Deepseek-3.2). Each box represents the interquartile range, and the horizontal line within the box denotes the median. The horizontal axis displays the model’s name, and the vertical axis represents the evaluation score. **(B)** Bar chart showing the mean score and standard deviation for each model. Error bars indicate the standard deviation.

According to the Likert chart of LLMs’ performing comparison, Qwen-3.5-plus, Gemini-3.1-pro and Deepseek-3.2 were included into patient interaction evaluation. **Figure 5** presents the comparison of patient evaluation scores among the three selected LLMs. The box plot (**Figure 5A**) depicts the score distribution for each model. Qwen-3.5-plus exhibited the highest median score (4.00) with a relatively wide interquartile range, indicating greater variability in its responses. Gemini-3.1-pro and Deepseek-3.2 had identical median scores (both 4.00) but with narrower distributions. The bar chart (**Figure 5B**) presents the mean score and standard deviation for each model. Qwen-3.5-plus attained the highest mean score (4.40 ± 0.60), followed by Deepseek-3.2 (3.75 ± 0.44) and Gemini-3.1-pro (3.55 ± 0.51).

To evaluate the rationality of the model structure, we calculated the variance components of random effects. Results showed that the question-level variance was 0.155, the patient-level variance was 0.059, and the residual variance was 0.185. This indicates that approximately 36% of the total variation in satisfaction can be explained by differences between questions, while patient individual differences only explained approximately 19%. These results support the inclusion of ‘question’ as a key random effect in the model and suggest that future research could further explore the impact of question characteristics on patient satisfaction (**Supplementary Table 8**).

**Figure 6:**
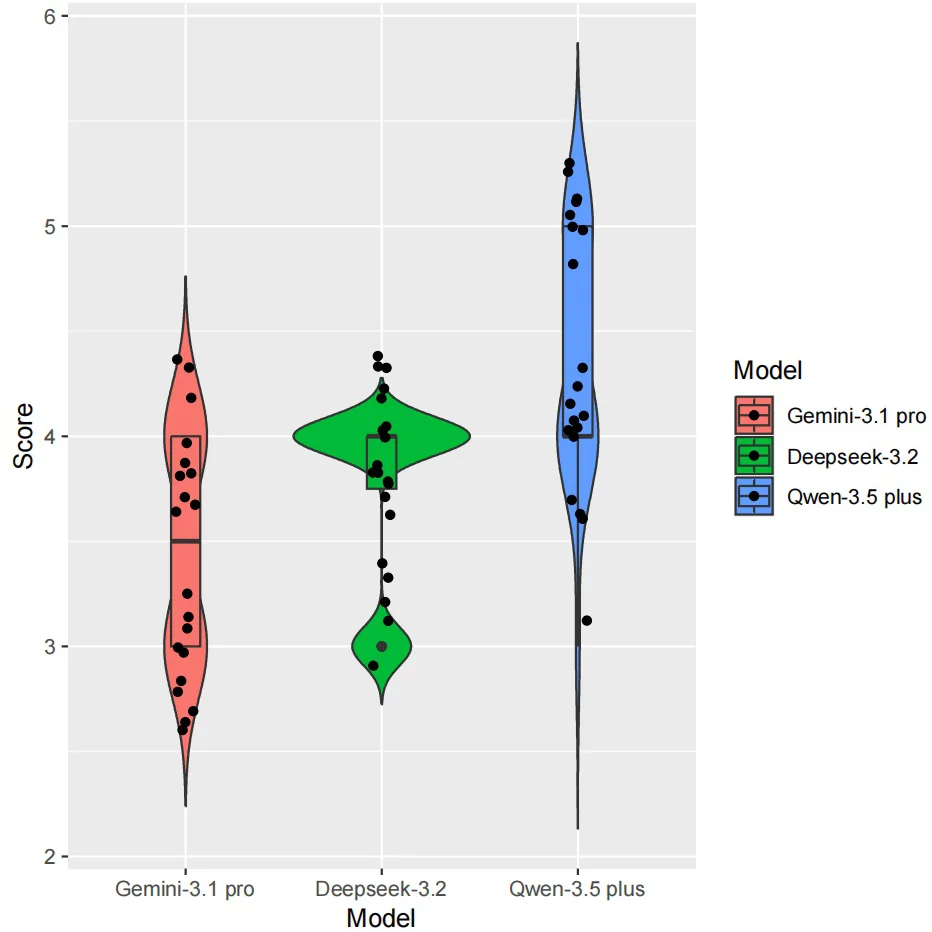
Distribution of patient satisfaction scores across the three selected large language models.

The LMM demonstrated a significant overall effect of model type on patient satisfaction scores (*F* = 22.78, *P* < 0.001). *Post hoc* pairwise comparisons with Bonferroni correction showed that Qwen-3.5 Plus achieved significantly higher satisfaction scores than both Gemini-3.1 Pro (mean difference = 0.85, *P* < 0.001) and DeepSeek-3.2 (mean difference = 0.60, *P* < 0.001). No significant difference was observed between Gemini-3.1 Pro and DeepSeek-3.2 (mean difference = 0.25, *P* = 0.183). The result is demonstrated in **Figure 6**; **Supplementary Tables 9a**, **S9b**.

## 4 Discussion

### 4.1 Principal findings

To our knowledge, this study presents the first systematic comparison of the educational effectiveness of six mainstream LLMs for NMOSD patients in experts’ assessment and real-world patients’ feedback. By using two Phases evaluation, integrating objective and subjective feedbacks, we demonstrated substantial heterogeneity among LLMs across different education dimensions. Our findings indicate that Deepseek-3.2 achieved the best performance on objective Chinese readability metrics, generating responses with the lowest reading difficulty and a recommended reading age of only 13.45 years. Meanwhile, Gemini-3.1-pro demonstrated significant advantages in accuracy, readability, and safety, whereas Qwen-3.5-plus showed marked superiority in information completeness and humanity. Importantly, the superiority of Qwen-3.5-plus in patient satisfaction was consistent with expert evaluations, providing preliminary evidence supporting the importance of empathy in AI-assisted patient education. Moreover, these findings suggest that no single model can be considered universally optimal for all patient education; instead, different models exhibit distinct strengths according to specific educational needs. More broadly, our results highlight the importance of evaluating LLMs from multiple perspectives, including not only technical performance and expert judgment but also the experiences of end users.

Notably, a marked discrepancy emerged between the objective readability metrics and the subjective expert readability ratings in this study. DeepSeek-3.2 achieved the lowest reading difficulty score and the youngest recommended reading age, yet its expert-rated readability score remained lower than that of Gemini-3.1-pro. A retrospective analysis of the response texts from these two models revealed the potential mechanism underlying this discrepancy. Deepseek-3.2 tended to employ short sentence structures, colloquial expressions, and high-frequency everyday vocabulary, thereby reducing linguistic complexity and improving readers’ accessibility. In contrast, Gemini-3.1-pro demonstrated stronger logical progression and greater refinement in medical terminology, aligning more closely with clinical experts’ cognitive expectations of “professional readability”.

This finding is consistent with several recent studies. Zhang et al. (2026) reported a significant disconnect between readability and information quality in Chinese-language skin cancer educational materials generated by LLMs, demonstrating that highly readable content is not necessarily associated with superior educational quality. Similarly, Li et al. (2026) observed weak correlations between traditional readability indices and patient education quality measures in AI-generated educational materials for autoimmune hepatitis. These observations suggest that conventional readability formulas may only capture the superficial linguistic characteristics, such as sentence length and vocabulary complexity, while overlooking deeper linguistic dimensions including coherence, conceptual density, information organization, and contextual appropriateness. Nattam et al. (2025) also noted in the background of rapid advancement in LLMs and integration with health information and patients’ educations, the existing readability assessment of AI-generated material still facing the challenges to assess comprehensively. The American Medical Association recommended that patient education materials should be written at a sixth-grade reading level or lower to meet public health literacy needs (Can artificial intelligence improve the readability of patient education information in gynecology?). Our findings indicate that linguistic simplicity alone does not guarantee effective patient communication (Swisher et al., 2024). Therefore, when deploying LLMs to support Chinese-language NMOSD patient education, a dynamic balance must be struck between “accessibility” and “professionalism.” High-quality AI-assisted patient education, in the truest sense, can only be achieved by ensuring linguistic accessibility while preserving the accuracy of medical terminology.

This study further elucidates the differential performance of LLMs across the dimensions of accuracy, safety, and completeness, along with their potential underlying causes. In the dimensions of accuracy and safety, Gemini-3.1-pro achieved the highest scores for both accuracy and safety while exhibiting the smallest inter-question variability. This finding is broadly consistent with previous studies evaluating LLM performance in ophthalmology and medical education (Bineshfar et al., 2026). In another blinded study employing the Medieval evaluation framework, Gemini likewise received the highest raw scores (Chen et al., 2026). Gemini’s accuracy and safety advantage may stem from a higher proportion of authoritative medical literature, evidence-based guidelines and its conservative and rigorous output strategy Furthermore, Qwen-3.5-plus demonstrated the highest completeness scores among all evaluated models. Compared with other LLMs, Qwen consistently generated longer and more detailed responses, covering disease mechanisms, diagnostic considerations, treatment options, prognosis, and long-term management strategies in a comprehensive manner. Numerous studies have demonstrated Qwen’s strengths in Chinese-language contexts. In evaluations using the Chinese National Medical Licensing Examination benchmark dataset and the Chinese National Nursing Qualification Examination, Qwen achieved accuracy rates significantly surpassing those of other LLMs (Zhu et al., 2025). These results suggest that the model possesses a deep foundation in Chinese medical knowledge coverage, which may directly facilitate its advantage in generating comprehensive content for patient education. Nevertheless, the interpretation of completeness scores requires caution. Longer responses may increase the likelihood that evaluators perceive content as comprehensive, regardless of whether additional information contributes meaningfully to educational quality, introducing a potential length-related bias. This concern is particularly relevant in our study, as Qwen-3.5-plus—the model with the highest completeness scores—also produced the most verbose outputs (2,126.45 ± 419.25 characters), approximately twice the length of the shortest responses. Therefore, its completeness advantage may be partially attributable to response length rather than true informational superiority. To address this limitation, future studies should consider incorporating objective measures of information density, such as the number of clinically relevant knowledge points per 100 characters, to distinguish genuine completeness from mere verbosity. We have acknowledged this length bias as a limitation of the present study.

Qwen-3.5-plus demonstrated dual superiority in both the humanity dimension and Phase two real-world patient satisfaction. Compared with other models, Qwen’s responses exhibited a high frequency of empathic communication cues that convey both respect and emotional responsiveness, such as “we understand your concerns,” “please be sure to discuss this with your attending physician,” and “we take your feelings very seriously.” This linguistic characteristic may account for its significantly superior performance in the humanity dimension relative to the other LLMs. A recent cross-lingual LLM evaluation study focusing on blepharoptosis similarly found that Qwen-2.5 exhibited significantly better linguistic clarity in Chinese patient questions than in English scenarios, suggesting that the model may possess inherent advantages in localized Chinese empathic expression (Niu et al., 2025). Empathy has been repeatedly validated as a core mediating variable driving patient satisfaction. A systematic review encompassing 11 studies indicated that physician empathy expression and its positive impact on patient outcomes suggest that empathy can enhance patient satisfaction through multiple pathways. Another systematic review of 12 studies noted that LLMs have demonstrated elements of cognitive empathy, including emotion recognition and the provision of emotionally supportive responses (Sorin et al., 2024). Unlike many previous investigations that relied exclusively on clinician ratings, our study incorporated patients’ feedback into the evaluation framework. The significantly higher satisfaction scores received by Qwen-3.5-plus suggest that empathic communication may substantially influence patient acceptance of AI-assisted educational tools. However, as discussed below, this patient-reported acceptability should be clearly distinguished from expert-assessed accuracy and safety. The significantly higher satisfaction scores received by Qwen-3.5-plus therefore provide real-world evidence that empathic communication may substantially influence patient acceptance of AI-assisted educational tools. A comparative study of oculoplastic patient consultations revealed that ChatGPT significantly outperformed BARD in empathic expression, indicating that empathic capabilities differ among LLMs (Al-Sharif et al., 2024). This difference between LLMs’ performance also discovered in our study, as cultural and linguistic factors may also contribute to these findings. Previous studies have suggested that empathic communication strategies developed in English-speaking contexts may not fully translate into Chinese healthcare settings (Mao and Zhao, 2020; Zhang, 2021). Qwen, as a domestically developed model trained in a native Chinese-language environment, may possess a stronger capacity to model the pragmatic features of this “Chinese-style empathy,” thereby demonstrating a dual advantage of professional authority and emotional warmth in the present evaluation. Nevertheless, this hypothesis requires further examination through additional cross-cultural comparative studies.

Despite Qwen’s demonstrated strengths in empathy and patient satisfaction, this does not imply that its factual reliability or clinical safety have been adequately validated. Inooka et al. (2026) conducted an ophthalmology-specific prospective evaluation of ChatGPT-4o assisted clinical diagnostic reasoning. In that study, ChatGPT-4o assistance improved scores for coherence, comprehensiveness, and safety, but did not enhance factual accuracy. Importantly, approximately 44% of the additional citations introduced after ChatGPT-4o assistance were fabricated, non-existent, or incorrect, and the variability in factual accuracy and safety increased. This suggests that readability, empathy, and patient satisfaction cannot be assumed to establish factual reliability or clinical safety. Our design, including both expert assessments and patient feedback, explicitly distinguishes these complementary purposes.

### 4.2 Comparison with prior work

Our findings are broadly consistent with previous studies evaluating LLMs in ophthalmology and neuro-ophthalmology. In a series of randomized, blinded, multi-center studies, Prashant D. et al (Tailor et al., 2024). reported that LLM responses performed comparably to human experts in terms of quality, empathy, and safety. Moreover, the expert edited LLM material were even preferred over independently written expert materials (Tailor et al., 2024). However, these studies largely treated LLMs as a single category and did not examine differences among individual models. Wang et al. (2025) recently adopted a similar two-Phase design combining expert assessment and patient interaction to evaluate the application of large language models in patient education for conjunctivitis, which provides a direct methodological precedent for our study. Compared with the study by Wang et al. (2025), our study further focuses on NMOSD, a rare disease area, and incorporates a greater number of domestic large language models for comparison, thereby extending previous work in terms of disease specificity and model diversity.

The present study extends these findings by demonstrating substantial heterogeneity across contemporary LLMs (Qiu et al., 2025). Although all evaluated models provided generally satisfactory responses for NMOSD patient education, differences were observed in readability, accuracy, completeness, safety, and humanity. Similar observations have been reported in other studies. Bineshfar et al. (2026) (Zhang et al., 2026) found that Gemini generated more readable patient education materials than ChatGPT despite comparable accuracy, while studies in oculoplastic consultations reported significant differences in empathic communication among LLMs (Al-Sharif et al., 2024). Collectively, these findings suggest that model selection is an important consideration, and besides knowledge level, the linguistic backgrounds are also significant for better education and instruction.

Notably, all evaluated models demonstrated relatively strong performance despite NMOSD being a rare neuro-immunological disease. This may reflect the increasing availability of high-quality NMOSD guidelines and consensus statements, including the 2025 Chinese NMOSD Diagnosis and Treatment Guidelines and international diagnostic criteria, which provide structured and evidence-based information for model training and knowledge retrieval.

### 4.3 Clinical implications

The findings of this study have several practical implications for AI assisted NMOSD patient education. First, different models appear to possess distinct strengths that may be advantageous in different educational settings. Models with stronger readability may be particularly useful for primary educational materials and disease awareness, whereas models providing more comprehensive and empathic responses may be better suited for patient further counseling. In contrast, models demonstrating superior accuracy and safety may be preferable when clinicians use LLMs as supplementary information resources.

Importantly, these findings should not be interpreted as supporting the independent use of LLMs for medical decision-making. Patient satisfaction scores, while indicating high acceptability, do not validate the medical reliability or safety of the responses for independent patient use. Although overall performance was encouraging, no model achieved consistently optimal results across all domains. Previous studies have shown that even high-performing LLMs may generate inaccurate recommendations or ignore clinically important information.36 Therefore, LLMs should be regarded as supplementary tools rather than substitutes for professional medical consultation. Human especially experts’ supervision remains essential, particularly in situations involving diagnosis, treatment decisions, medication adjustments, or other high-risk clinical issues. All AI-generated content intended for patient use should be reviewed and confirmed by a qualified clinician before being provided to patients, regardless of how satisfactory or empathic the responses may appear.

### 4.4 Strengths and limitations

This study has several strengths. First, to our knowledge, it is among the first studies to systematically evaluate multiple mainstream LLMs for patient education in NMOSD. Second, we adopted a comprehensive evaluation framework that combined blinded expert assessment with preliminary patient acceptability feedback, providing a more clinically relevant perspective than previous studies. In addition, the inclusion of both leading international and Chinese LLMs enhances the applicability of the findings to Chinese-language clinical settings.

Several limitations should be acknowledged. First, the patient interaction Phase included only 10 participants from a single center, which limits the generalizability of the satisfaction findings. While this sample size was appropriate for a preliminary feasibility assessment, the small cohort may not capture the full diversity of NMOSD patients. Thus, these findings should be interpreted as exploratory and require confirmation in larger, multi-center studies. Second, although Bonferroni correction was applied within each set of *post-hoc* comparisons, the lack of study-wide correction across multiple dimensions and models warrants cautious interpretation of the significance results. Additionally, the readability analysis relied on a single platform accessed via an IP address, which may not be persistently available; we have provided the underlying formulas in the **Supplementary Materials** to facilitate reproducibility. Future studies may consider using multiple readability instruments for cross-validation. Secondly, LLM performance may change over time because of model updates. Although all evaluations were conducted within a short time window and model parameters were standardized to improve repeatability, the performance observed in this study may not fully reflect future model behavior. Third, although we standardized model parameters to the greatest extent possible, the six models were accessed via heterogeneous routes, including official APIs (Gemini-3.1-pro and Qwen-3.5-plus), official web interfaces (ChatGPT-5.4, Deepseek-3.2, and Kimi-2.5), and a third-party platform (Claude-4.6-Sonnet via Poe.com). For models accessed via web interfaces, certain low-level API parameters are not fully controllable through the user interface. Similarly, third-party platforms may apply additional system-level prompts or safety filters that could influence response generation. This heterogeneity introduces potential confounding and should be considered when interpreting the comparative rankings of models. Fourth, the question set was limited to 20 NMOSD-related questions and may not have fully including all clinically relevant application, particularly high-risk situations requiring urgent medical attention. Notably, Mori et al. (2026) reported that among patients referred with suspected optic neuritis, only 43.2% were eventually confirmed to have optic neuritis, with other major diagnoses including anterior ischemic optic neuropathy, intracranial space-occupying lesions, retinal diseases, and uveitis. This finding carries important implications for the safety assessment of patient education in NMOSD: acute visual symptoms should not be simplistically attributed to NMOSD relapse or optic neuritis, but rather warrant careful differential diagnosis. A clinically meaningful NMOSD patient education tool should not only provide general disease information but also, when appropriate, identify warning signs and direct patients to urgent specialist evaluation. The question set in the current study primarily covers general educational topics and does not adequately incorporate such high-risk acute scenarios, which also constitutes one of the limitations of the safety assessment in our study. Finally, the study evaluated only single turn interactions. Future research should assess model performance in multi turn conversations, which more closely resemble real world patient education and counseling.

## 5 Conclusion

In conclusion, mainstream LLMs demonstrated generally satisfactory performance in NMOSD patient education, although substantial differences were observed across readability, accuracy, completeness, safety, humanity, and patient satisfaction. DeepSeek-3.2 showed advantages in objective readability, Gemini-3.1-pro achieved the strongest performance in accuracy and safety, and Qwen-3.5-plus demonstrated superiority in completeness, humanity, and showed encouraging but preliminary patient satisfaction results that warrant confirmation in future studies with larger patient cohorts. These findings suggest that different LLMs may be better suited to different educational objectives and clinical contexts. While LLMs show considerable promise as tools for supporting NMOSD patient education, their outputs should continue to be interpreted under appropriate clinical supervision, with human supervise remaining essential for safe and effective implementation.

## Data Availability

The full data supporting this study—including expert scoring data from Phase one, patient questions and scoring records from Phase two—can be accessed in Multimedia Appendix 1–11.
