# Supplementary material for: Patient education for neuromyelitis optica spectrum disorder using large language models: combining expert assessment and real-world patient interaction
Source: Front Physiol. 2026 Aug 19;17:1923189. doi: 10.3389/fphys.2026.1923189 (PMC13534020; doi:10.3389/fphys.2026.1923189)
Supplement: Supplementary file 1 [file Table1.docx]

**Table S12**

| **Metric** | **Model** | **Eta_squared** | ***F*_statistic** | ***P*_value** | **Mean** | **SD** | **95% CI Lower** | **95% CI Upper** |
| --- | --- | --- | --- | --- | --- | --- | --- | --- |
| Reading difficulty score | Overall | 0.608707473 | 35.46842686 | 9.5653E-22 |  |  |  |  |
| Reading difficulty score | ChatGPT-5.4 |  |  |  | 14.38 | 1.40 | 13.77 | 14.99 |
| Reading difficulty score | Gemini-3.1-pro |  |  |  | 14.40 | 0.91 | 14.00 | 14.80 |
| Reading difficulty score | Claude-4.6-Sonnet |  |  |  | 18.17 | 3.18 | 16.77 | 19.56 |
| Reading difficulty score | Deepseek-3.2 |  |  |  | 13.54 | 0.78 | 13.20 | 13.88 |
| Reading difficulty score | Kimi-2.5 |  |  |  | 21.96 | 4.49 | 19.99 | 23.92 |
| Reading difficulty score | Qwen-3.5-plus |  |  |  | 15.66 | 0.86 | 15.29 | 16.04 |
| Chinese character count | Overall | 0.430789609 | 17.25548803 | 1.099E-12 |  |  |  |  |
| Chinese character count | ChatGPT-5.4 |  |  |  | 1750.30 | 714.49 | 1437.16 | 2063.44 |
| Chinese character count | Gemini-3.1-pro |  |  |  | 1094.25 | 299.67 | 962.91 | 1225.59 |
| Chinese character count | Claude-4.6-Sonnet |  |  |  | 971.30 | 182.21 | 891.44 | 1051.16 |
| Chinese character count | Deepseek-3.2 |  |  |  | 1535.35 | 514.21 | 1309.99 | 1760.71 |
| Chinese character count | Kimi-2.5 |  |  |  | 1624.25 | 444.79 | 1429.31 | 1819.19 |
| Chinese character count | Qwen-3.5-plus |  |  |  | 2126.45 | 419.25 | 1942.70 | 2310.20 |
| Recommended reading age | Overall | 0.640466154 | 40.61544828 | 8.26987E-24 |  |  |  |  |
| Recommended reading age | ChatGPT-5.4 |  |  |  | 14.30 | 1.22 | 13.77 | 14.83 |
| Recommended reading age | Gemini-3.1-pro |  |  |  | 14.35 | 1.09 | 13.87 | 14.83 |
| Recommended reading age | Claude-4.6-Sonnet |  |  |  | 16.70 | 1.42 | 16.08 | 17.32 |
| Recommended reading age | Deepseek-3.2 |  |  |  | 13.45 | 0.83 | 13.09 | 13.81 |
| Recommended reading age | Kimi-2.5 |  |  |  | 17.65 | 1.18 | 17.13 | 18.17 |
| Recommended reading age | Qwen-3.5-plus |  |  |  | 15.65 | 0.93 | 15.24 | 16.06 |
| Accuracy_Score | Overall | 0.076011846 | 5.824358974 | 3.47426E-05 |  |  |  |  |
| Accuracy_Score | ChatGPT-5.4 |  |  |  | 3.87 | 0.47 | 3.75 | 3.99 |
| Accuracy_Score | Gemini-3.1-pro |  |  |  | 3.97 | 0.18 | 3.92 | 4.01 |
| Accuracy_Score | Claude-4.6-Sonnet |  |  |  | 3.75 | 0.44 | 3.64 | 3.86 |
| Accuracy_Score | Deepseek-3.2 |  |  |  | 3.78 | 0.49 | 3.66 | 3.91 |
| Accuracy_Score | Kimi-2.5 |  |  |  | 3.55 | 0.59 | 3.40 | 3.70 |
| Accuracy_Score | Qwen-3.5-plus |  |  |  | 3.85 | 0.44 | 3.74 | 3.96 |
| Completeness_Score | Overall | 0.254758448 | 24.2027542 | 5.8795E-21 |  |  |  |  |
| Completeness_Score | ChatGPT-5.4 |  |  |  | 4.07 | 0.71 | 3.89 | 4.25 |
| Completeness_Score | Gemini-3.1-pro |  |  |  | 3.60 | 0.53 | 3.47 | 3.73 |
| Completeness_Score | Claude-4.6-Sonnet |  |  |  | 3.18 | 0.54 | 3.05 | 3.32 |
| Completeness_Score | Deepseek-3.2 |  |  |  | 3.92 | 0.42 | 3.81 | 4.02 |
| Completeness_Score | Kimi-2.5 |  |  |  | 3.82 | 0.54 | 3.68 | 3.95 |
| Completeness_Score | Qwen-3.5-plus |  |  |  | 4.10 | 0.48 | 3.98 | 4.22 |
| Readability_Score | Overall | 0.247958258 | 23.34371041 | 2.82066E-20 |  |  |  |  |
| Readability_Score | ChatGPT-5.4 |  |  |  | 3.82 | 0.43 | 3.71 | 3.93 |
| Readability_Score | Gemini-3.1-pro |  |  |  | 4.08 | 0.28 | 4.01 | 4.15 |
| Readability_Score | Claude-4.6-Sonnet |  |  |  | 3.58 | 0.50 | 3.46 | 3.71 |
| Readability_Score | Deepseek-3.2 |  |  |  | 3.87 | 0.39 | 3.77 | 3.97 |
| Readability_Score | Kimi-2.5 |  |  |  | 3.25 | 0.60 | 3.10 | 3.40 |
| Readability_Score | Qwen-3.5-plus |  |  |  | 3.67 | 0.48 | 3.55 | 3.79 |
| Safety_Score | Overall | 0.03776361 | 2.778593272 | 0.017741009 |  |  |  |  |
| Safety_Score | ChatGPT-5.4 |  |  |  | 3.93 | 0.25 | 3.87 | 4.00 |
| Safety_Score | Gemini-3.1-pro |  |  |  | 4.00 | 0.00 | 4.00 | 4.00 |
| Safety_Score | Claude-4.6-Sonnet |  |  |  | 3.87 | 0.39 | 3.77 | 3.97 |
| Safety_Score | Deepseek-3.2 |  |  |  | 4.00 | 0.00 | 4.00 | 4.00 |
| Safety_Score | Kimi-2.5 |  |  |  | 3.90 | 0.30 | 3.82 | 3.98 |
| Safety_Score | Qwen-3.5-plus |  |  |  | 3.93 | 0.25 | 3.87 | 4.00 |
| Humanity_Score | Overall | 0.369428504 | 41.47909999 | 1.45684E-33 |  |  |  |  |
| Humanity_Score | ChatGPT-5.4 |  |  |  | 3.47 | 0.72 | 3.28 | 3.65 |
| Humanity_Score | Gemini-3.1-pro |  |  |  | 3.43 | 0.72 | 3.25 | 3.62 |
| Humanity_Score | Claude-4.6-Sonnet |  |  |  | 2.90 | 0.73 | 2.72 | 3.08 |
| Humanity_Score | Deepseek-3.2 |  |  |  | 3.95 | 0.70 | 3.77 | 4.13 |
| Humanity_Score | Kimi-2.5 |  |  |  | 3.13 | 0.72 | 2.95 | 3.32 |
| Humanity_Score | Qwen-3.5-plus |  |  |  | 4.50 | 0.57 | 4.36 | 4.64 |

**Table S13**

| **Dimension** | **Rank** | **LME_Model** | **LME_Mean** | **ANOVA_Model** | **ANOVA_Mean** | **Match** |
| --- | --- | --- | --- | --- | --- | --- |
| Accuracy | 1 | Gemini-3.1-pro | 3.966666667 | Gemini-3.1-pro | 3.97 | Yes |
| Accuracy | 2 | ChatGPT-5.4 | 3.866666667 | ChatGPT-5.4 | 3.87 | Yes |
| Accuracy | 3 | Qwen-3.5-plus | 3.85 | Qwen-3.5-plus | 3.85 | Yes |
| Accuracy | 4 | Deepseek-3.2 | 3.783333333 | Deepseek-3.2 | 3.78 | Yes |
| Accuracy | 5 | Claude-4.6-Sonnet | 3.75 | Claude-4.6-Sonnet | 3.75 | Yes |
| Accuracy | 6 | Kimi-2.5 | 3.55 | Kimi-2.5 | 3.55 | Yes |
| Completeness | 1 | Qwen-3.5-plus | 4.1 | Qwen-3.5-plus | 4.1 | Yes |
| Completeness | 2 | ChatGPT-5.4 | 4.066666667 | ChatGPT-5.4 | 4.07 | Yes |
| Completeness | 3 | Deepseek-3.2 | 3.916666667 | Deepseek-3.2 | 3.92 | Yes |
| Completeness | 4 | Kimi-2.5 | 3.816666667 | Kimi-2.5 | 3.82 | Yes |
| Completeness | 5 | Gemini-3.1-pro | 3.6 | Gemini-3.1-pro | 3.6 | Yes |
| Completeness | 6 | Claude-4.6-Sonnet | 3.183333333 | Claude-4.6-Sonnet | 3.18 | Yes |
| Readability | 1 | Gemini-3.1-pro | 4.083333333 | Gemini-3.1-pro | 4.08 | Yes |
| Readability | 2 | Deepseek-3.2 | 3.866666667 | Deepseek-3.2 | 3.87 | Yes |
| Readability | 3 | ChatGPT-5.4 | 3.816666667 | ChatGPT-5.4 | 3.82 | Yes |
| Readability | 4 | Qwen-3.5-plus | 3.666666667 | Qwen-3.5-plus | 3.67 | Yes |
| Readability | 5 | Claude-4.6-Sonnet | 3.583333333 | Claude-4.6-Sonnet | 3.58 | Yes |
| Readability | 6 | Kimi-2.5 | 3.25 | Kimi-2.5 | 3.25 | Yes |
| Safety | 1 | Gemini-3.1-pro | 4 | Gemini-3.1-pro | 4 | Yes |
| Safety | 2 | Deepseek-3.2 | 4 | Deepseek-3.2 | 4 | Yes |
| Safety | 3 | ChatGPT-5.4 | 3.933333333 | ChatGPT-5.4 | 3.93 | Yes |
| Safety | 4 | Qwen-3.5-plus | 3.933333333 | Qwen-3.5-plus | 3.93 | Yes |
| Safety | 5 | Kimi-2.5 | 3.9 | Kimi-2.5 | 3.9 | Yes |
| Safety | 6 | Claude-4.6-Sonnet | 3.866666667 | Claude-4.6-Sonnet | 3.87 | Yes |
| Humanity | 1 | Qwen-3.5-plus | 4.5 | Qwen-3.5-plus | 4.5 | Yes |
| Humanity | 2 | Deepseek-3.2 | 3.95 | Deepseek-3.2 | 3.95 | Yes |
| Humanity | 3 | ChatGPT-5.4 | 3.466666667 | ChatGPT-5.4 | 3.47 | Yes |
| Humanity | 4 | Gemini-3.1-pro | 3.433333333 | Gemini-3.1-pro | 3.43 | Yes |
| Humanity | 5 | Kimi-2.5 | 3.133333333 | Kimi-2.5 | 3.13 | Yes |
| Humanity | 6 | Claude-4.6-Sonnet | 2.9 | Claude-4.6-Sonnet | 2.9 | Yes |

**Table S14**

| **Metric** | **Deepseek_Mean** | **Deepseek_STD** | **Deepseek_N** | **Gemini_Mean** | **Gemini_STD** | **Gemini_N** | ***t*_Statistic** | ***P*_Value** | **significance** | **Cohens_*d*** |
| --- | --- | --- | --- | --- | --- | --- | --- | --- | --- | --- |
| Reading difficulty score | 13.54 | 0.781779347 | 20 | 14.3985 | 0.908030112 | 20 | -3.204230908 | 0.002741747 | ** | -1.013266782 |
| Chinese character count | 1535.35 | 514.2133773 | 20 | 1094.25 | 299.6708501 | 20 | 3.314490149 | 0.002025287 | ** | 1.048133815 |
| Recommended reading age | 13.45 | 0.825577947 | 20 | 14.35 | 1.089422831 | 20 | -2.944558122 | 0.005492761 | ** | -0.931151037 |
