# Supplementary material for: Patient education for neuromyelitis optica spectrum disorder using large language models: combining expert assessment and real-world patient interaction
Source: Front Physiol. 2026 Aug 19;17:1923189. doi: 10.3389/fphys.2026.1923189 (PMC13534020; doi:10.3389/fphys.2026.1923189)
Supplement: Supplementary file 2 [file Table2.docx]

**Table S1.** Phase 1 questionnaire design.

| No. | Question | Category |
| --- | --- | --- |
| Q1 | How is NMOSD diagnosed? | Disease Diagnosis |
| Q2 | What is the difference between NMOSD and Multiple Sclerosis? | Disease Diagnosis |
| Q3 | Can NMOSD be diagnosed if the AQP4 antibody test is negative? | Disease Diagnosis |
| Q4 | What are the treatment options for an acute attack of NMOSD? | Acute Episode and Treatment |
| Q5 | Why do people with NMOSD experience intractable hiccups, nausea, and vomiting? | Acute Episode and Treatment |
| Q6 | How can NMOSD relapses be effectively prevented? | Long-term Prevention |
| Q7 | What are the current first-line preventive medications for NMOSD during the remission phase? | Medication Selection |
| Q8 | What are the respective advantages and disadvantages of newer monoclonal antibody drugs compared to traditional immunosuppressive agents in the treatment of NMOSD? | Medication Selection |
| Q9 | What are the main side effects of long-term use of medications to prevent NMOSD relapses? | Medication Selection |
| Q10 | Can I get vaccinated while taking medication for NMOSD? | Medication Selection |
| Q11 | How can I manage long-term neuropathic pain, muscle spasms, or intractable itching caused by NMOSD? | Symptom Management |
| Q12 | What can be done for bladder and bowel dysfunction caused by NMOSD? | Symptom Management |
| Q13 | After NMOSD stabilizes, how should scientific and safe rehabilitation exercises be conducted? | Disease Prognosis |
| Q14 | Can NMOSD be cured? | Disease Prognosis |
| Q15 | What is the long-term prognosis for NMOSD? | Disease Prognosis |
| Q16 | Can women with NMOSD become pregnant and have children? | Disease Prognosis |
| Q17 | How is the disease managed during pregnancy and postpartum to reduce the risk of an NMOSD relapse? | Disease Prognosis |
| Q18 | Is NMOSD hereditary? | Disease Prognosis |
| Q19 | Are there any dietary restrictions for people with NMOSD? | Lifestyle Management |
| Q20 | Facing a high risk of relapse and disability, I feel very anxious and depressed. What should I do? | Psychological Support |

**Table S2.** Likert 5-point rating scale.

| Score | | Accuracy | Completeness | Readability | Safety | Humanity |
| --- | --- | --- | --- | --- | --- | --- |
| 1 | The response is completely inaccurate, containing misleading or erroneous information. | | The response is extremely incomplete, missing key content. | The response is difficult to understand, with unclear and confusing language. | The response contains unsafe health recommendations or content that could mislead patients. | The response is very indifferent, ignoring the emotional needs of patients. |
| 2 | The response is partially accurate but contains some errors or incomplete information. | | The response is somewhat comprehensive but omits critical or important details. | The response is somewhat understandable but includes ambiguous or unclear parts. | The response is partially safe, but some suggestions may pose potential risks. | The response is somewhat indifferent, lacking empathy for patients. |
| 3 | The response is mostly accurate but contains minor errors. | | The response is mostly comprehensive, though some information is missing. | The response is mostly understandable, but some parts may require further clarification. | The response is largely safe, though certain suggestions may require further verification or revision. | The response is mostly human-centered but still has room for improvement. |
| 4 | The response is generally accurate, with few errors. | | The response is generally comprehensive, covering most relevant information. | The response is generally clear and easy to understand. | The response is generally safe and aligns with the patient's health needs. | The response demonstrates empathy and consideration for the patient's emotions. |
| 5 | The response is completely accurate, with no errors. | | The response is highly comprehensive, covering all relevant information. | The response is highly clear, concise, and well-structured. | The response is entirely safe, with no potential risks. | The response is highly human-centered, full of care and empathy. |

**Table S3.** Experts scoring data.

| **Question_ID** | **Model_ID** | **Model_Name** | **Evaluator_ID** | **Accuracy_Score** | **Completeness_Score** | **Readability_Score** | **Safety_Score** | **Humanity_Score** |
| --- | --- | --- | --- | --- | --- | --- | --- | --- |
| Q001 | M01 | ChatGPT-5.4 | R01 | 4 | 5 | 4 | 4 | 4 |
| Q001 | M01 | ChatGPT-5.4 | R02 | 3 | 5 | 4 | 4 | 2 |
| Q001 | M01 | ChatGPT-5.4 | R03 | 4 | 5 | 4 | 4 | 3 |
| Q001 | M02 | Gemini-3.1-pro | R01 | 4 | 4 | 4 | 4 | 4 |
| Q001 | M02 | Gemini-3.1-pro | R02 | 3 | 3 | 4 | 4 | 3 |
| Q001 | M02 | Gemini-3.1-pro | R03 | 4 | 4 | 4 | 4 | 4 |
| Q001 | M03 | Claude-4.6-Sonnet | R01 | 3 | 3 | 3 | 4 | 3 |
| Q001 | M03 | Claude-4.6-Sonnet | R02 | 3 | 3 | 3 | 4 | 2 |
| Q001 | M03 | Claude-4.6-Sonnet | R03 | 4 | 3 | 4 | 4 | 3 |
| Q001 | M04 | Deepseek-3.2 | R01 | 4 | 4 | 4 | 4 | 5 |
| Q001 | M04 | Deepseek-3.2 | R02 | 3 | 4 | 4 | 4 | 3 |
| Q001 | M04 | Deepseek-3.2 | R03 | 4 | 4 | 4 | 4 | 4 |
| Q001 | M05 | Kimi-2.5 | R01 | 3 | 3 | 3 | 4 | 3 |
| Q001 | M05 | Kimi-2.5 | R02 | 2 | 3 | 3 | 4 | 3 |
| Q001 | M05 | Kimi-2.5 | R03 | 3 | 3 | 3 | 4 | 3 |
| Q001 | M06 | Qwen-3.5-plus | R01 | 4 | 3 | 4 | 4 | 4 |
| Q001 | M06 | Qwen-3.5-plus | R02 | 3 | 3 | 4 | 4 | 4 |
| Q001 | M06 | Qwen-3.5-plus | R03 | 4 | 4 | 4 | 4 | 4 |
| Q002 | M01 | ChatGPT-5.4 | R01 | 4 | 5 | 4 | 4 | 4 |
| Q002 | M01 | ChatGPT-5.4 | R02 | 4 | 5 | 4 | 4 | 4 |
| Q002 | M01 | ChatGPT-5.4 | R03 | 4 | 5 | 4 | 4 | 4 |
| Q002 | M02 | Gemini-3.1-pro | R01 | 4 | 4 | 4 | 4 | 4 |
| Q002 | M02 | Gemini-3.1-pro | R02 | 4 | 4 | 4 | 4 | 3 |
| Q002 | M02 | Gemini-3.1-pro | R03 | 4 | 4 | 4 | 4 | 3 |
| Q002 | M03 | Claude-4.6-Sonnet | R01 | 4 | 3 | 3 | 4 | 2 |
| Q002 | M03 | Claude-4.6-Sonnet | R02 | 4 | 3 | 4 | 4 | 3 |
| Q002 | M03 | Claude-4.6-Sonnet | R03 | 4 | 4 | 4 | 4 | 3 |
| Q002 | M04 | Deepseek-3.2 | R01 | 4 | 4 | 3 | 4 | 3 |
| Q002 | M04 | Deepseek-3.2 | R02 | 3 | 4 | 3 | 4 | 4 |
| Q002 | M04 | Deepseek-3.2 | R03 | 4 | 4 | 3 | 4 | 4 |
| Q002 | M05 | Kimi-2.5 | R01 | 4 | 4 | 3 | 4 | 2 |
| Q002 | M05 | Kimi-2.5 | R02 | 4 | 4 | 3 | 4 | 4 |
| Q002 | M05 | Kimi-2.5 | R03 | 4 | 4 | 3 | 4 | 3 |
| Q002 | M06 | Qwen-3.5-plus | R01 | 4 | 4 | 3 | 4 | 4 |
| Q002 | M06 | Qwen-3.5-plus | R02 | 4 | 4 | 3 | 4 | 4 |
| Q002 | M06 | Qwen-3.5-plus | R03 | 4 | 4 | 3 | 4 | 4 |
| Q003 | M01 | ChatGPT-5.4 | R01 | 4 | 3 | 4 | 4 | 4 |
| Q003 | M01 | ChatGPT-5.4 | R02 | 3 | 3 | 4 | 4 | 3 |
| Q003 | M01 | ChatGPT-5.4 | R03 | 4 | 3 | 4 | 4 | 3 |
| Q003 | M02 | Gemini-3.1-pro | R01 | 4 | 4 | 4 | 4 | 4 |
| Q003 | M02 | Gemini-3.1-pro | R02 | 4 | 4 | 4 | 4 | 4 |
| Q003 | M02 | Gemini-3.1-pro | R03 | 4 | 4 | 4 | 4 | 4 |
| Q003 | M03 | Claude-4.6-Sonnet | R01 | 4 | 3 | 4 | 4 | 2 |
| Q003 | M03 | Claude-4.6-Sonnet | R02 | 4 | 3 | 4 | 4 | 2 |
| Q003 | M03 | Claude-4.6-Sonnet | R03 | 3 | 3 | 4 | 3 | 3 |
| Q003 | M04 | Deepseek-3.2 | R01 | 4 | 4 | 3 | 4 | 4 |
| Q003 | M04 | Deepseek-3.2 | R02 | 2 | 4 | 3 | 4 | 5 |
| Q003 | M04 | Deepseek-3.2 | R03 | 4 | 4 | 3 | 4 | 4 |
| Q003 | M05 | Kimi-2.5 | R01 | 4 | 4 | 3 | 4 | 3 |
| Q003 | M05 | Kimi-2.5 | R02 | 3 | 4 | 3 | 4 | 3 |
| Q003 | M05 | Kimi-2.5 | R03 | 4 | 4 | 3 | 4 | 3 |
| Q003 | M06 | Qwen-3.5-plus | R01 | 4 | 5 | 3 | 4 | 4 |
| Q003 | M06 | Qwen-3.5-plus | R02 | 3 | 4 | 3 | 4 | 5 |
| Q003 | M06 | Qwen-3.5-plus | R03 | 4 | 4 | 3 | 4 | 5 |
| Q004 | M01 | ChatGPT-5.4 | R01 | 4 | 5 | 4 | 4 | 5 |
| Q004 | M01 | ChatGPT-5.4 | R02 | 4 | 5 | 4 | 4 | 4 |
| Q004 | M01 | ChatGPT-5.4 | R03 | 4 | 5 | 4 | 4 | 4 |
| Q004 | M02 | Gemini-3.1-pro | R01 | 4 | 4 | 4 | 4 | 5 |
| Q004 | M02 | Gemini-3.1-pro | R02 | 4 | 4 | 4 | 4 | 4 |
| Q004 | M02 | Gemini-3.1-pro | R03 | 4 | 4 | 4 | 4 | 4 |
| Q004 | M03 | Claude-4.6-Sonnet | R01 | 4 | 3 | 4 | 4 | 3 |
| Q004 | M03 | Claude-4.6-Sonnet | R02 | 4 | 3 | 4 | 4 | 2 |
| Q004 | M03 | Claude-4.6-Sonnet | R03 | 4 | 3 | 4 | 4 | 2 |
| Q004 | M04 | Deepseek-3.2 | R01 | 3 | 4 | 4 | 4 | 4 |
| Q004 | M04 | Deepseek-3.2 | R02 | 2 | 4 | 4 | 4 | 5 |
| Q004 | M04 | Deepseek-3.2 | R03 | 3 | 4 | 4 | 4 | 5 |
| Q004 | M05 | Kimi-2.5 | R01 | 3 | 3 | 3 | 3 | 3 |
| Q004 | M05 | Kimi-2.5 | R02 | 2 | 3 | 3 | 3 | 2 |
| Q004 | M05 | Kimi-2.5 | R03 | 2 | 3 | 4 | 3 | 3 |
| Q004 | M06 | Qwen-3.5-plus | R01 | 4 | 4 | 3 | 4 | 3 |
| Q004 | M06 | Qwen-3.5-plus | R02 | 3 | 4 | 3 | 4 | 4 |
| Q004 | M06 | Qwen-3.5-plus | R03 | 4 | 4 | 4 | 4 | 5 |
| Q005 | M01 | ChatGPT-5.4 | R01 | 4 | 4 | 4 | 4 | 3 |
| Q005 | M01 | ChatGPT-5.4 | R02 | 4 | 4 | 3 | 4 | 3 |
| Q005 | M01 | ChatGPT-5.4 | R03 | 4 | 4 | 4 | 4 | 4 |
| Q005 | M02 | Gemini-3.1-pro | R01 | 4 | 4 | 5 | 4 | 3 |
| Q005 | M02 | Gemini-3.1-pro | R02 | 4 | 4 | 5 | 4 | 4 |
| Q005 | M02 | Gemini-3.1-pro | R03 | 4 | 4 | 5 | 4 | 3 |
| Q005 | M03 | Claude-4.6-Sonnet | R01 | 4 | 4 | 4 | 4 | 3 |
| Q005 | M03 | Claude-4.6-Sonnet | R02 | 4 | 4 | 3 | 4 | 4 |
| Q005 | M03 | Claude-4.6-Sonnet | R03 | 4 | 4 | 4 | 4 | 3 |
| Q005 | M04 | Deepseek-3.2 | R01 | 4 | 4 | 4 | 4 | 3 |
| Q005 | M04 | Deepseek-3.2 | R02 | 4 | 4 | 4 | 4 | 4 |
| Q005 | M04 | Deepseek-3.2 | R03 | 4 | 4 | 4 | 4 | 4 |
| Q005 | M05 | Kimi-2.5 | R01 | 3 | 4 | 3 | 4 | 2 |
| Q005 | M05 | Kimi-2.5 | R02 | 3 | 4 | 3 | 4 | 3 |
| Q005 | M05 | Kimi-2.5 | R03 | 3 | 4 | 3 | 4 | 4 |
| Q005 | M06 | Qwen-3.5-plus | R01 | 4 | 4 | 3 | 4 | 5 |
| Q005 | M06 | Qwen-3.5-plus | R02 | 4 | 4 | 4 | 4 | 4 |
| Q005 | M06 | Qwen-3.5-plus | R03 | 4 | 4 | 4 | 4 | 4 |
| Q006 | M01 | ChatGPT-5.4 | R01 | 4 | 4 | 4 | 4 | 2 |
| Q006 | M01 | ChatGPT-5.4 | R02 | 4 | 3 | 3 | 4 | 4 |
| Q006 | M01 | ChatGPT-5.4 | R03 | 4 | 4 | 4 | 4 | 2 |
| Q006 | M02 | Gemini-3.1-pro | R01 | 4 | 4 | 4 | 4 | 2 |
| Q006 | M02 | Gemini-3.1-pro | R02 | 4 | 3 | 4 | 4 | 3 |
| Q006 | M02 | Gemini-3.1-pro | R03 | 4 | 3 | 4 | 4 | 3 |
| Q006 | M03 | Claude-4.6-Sonnet | R01 | 4 | 3 | 3 | 4 | 2 |
| Q006 | M03 | Claude-4.6-Sonnet | R02 | 4 | 3 | 3 | 4 | 3 |
| Q006 | M03 | Claude-4.6-Sonnet | R03 | 4 | 3 | 3 | 4 | 3 |
| Q006 | M04 | Deepseek-3.2 | R01 | 4 | 3 | 4 | 4 | 5 |
| Q006 | M04 | Deepseek-3.2 | R02 | 4 | 3 | 4 | 4 | 4 |
| Q006 | M04 | Deepseek-3.2 | R03 | 4 | 3 | 4 | 4 | 3 |
| Q006 | M05 | Kimi-2.5 | R01 | 4 | 5 | 3 | 4 | 4 |
| Q006 | M05 | Kimi-2.5 | R02 | 4 | 5 | 3 | 4 | 4 |
| Q006 | M05 | Kimi-2.5 | R03 | 4 | 5 | 4 | 4 | 3 |
| Q006 | M06 | Qwen-3.5-plus | R01 | 4 | 5 | 4 | 4 | 4 |
| Q006 | M06 | Qwen-3.5-plus | R02 | 4 | 5 | 4 | 4 | 5 |
| Q006 | M06 | Qwen-3.5-plus | R03 | 4 | 4 | 4 | 4 | 4 |
| Q007 | M01 | ChatGPT-5.4 | R01 | 2 | 4 | 4 | 3 | 3 |
| Q007 | M01 | ChatGPT-5.4 | R02 | 2 | 4 | 3 | 3 | 3 |
| Q007 | M01 | ChatGPT-5.4 | R03 | 2 | 4 | 4 | 3 | 4 |
| Q007 | M02 | Gemini-3.1-pro | R01 | 4 | 3 | 4 | 4 | 3 |
| Q007 | M02 | Gemini-3.1-pro | R02 | 3 | 3 | 4 | 4 | 3 |
| Q007 | M02 | Gemini-3.1-pro | R03 | 4 | 3 | 4 | 4 | 3 |
| Q007 | M03 | Claude-4.6-Sonnet | R01 | 4 | 2 | 4 | 4 | 2 |
| Q007 | M03 | Claude-4.6-Sonnet | R02 | 4 | 2 | 4 | 4 | 3 |
| Q007 | M03 | Claude-4.6-Sonnet | R03 | 4 | 2 | 4 | 4 | 3 |
| Q007 | M04 | Deepseek-3.2 | R01 | 4 | 4 | 4 | 4 | 3 |
| Q007 | M04 | Deepseek-3.2 | R02 | 3 | 4 | 4 | 4 | 4 |
| Q007 | M04 | Deepseek-3.2 | R03 | 4 | 4 | 4 | 4 | 4 |
| Q007 | M05 | Kimi-2.5 | R01 | 4 | 4 | 3 | 4 | 2 |
| Q007 | M05 | Kimi-2.5 | R02 | 4 | 4 | 3 | 4 | 3 |
| Q007 | M05 | Kimi-2.5 | R03 | 4 | 3 | 3 | 4 | 3 |
| Q007 | M06 | Qwen-3.5-plus | R01 | 3 | 4 | 3 | 4 | 4 |
| Q007 | M06 | Qwen-3.5-plus | R02 | 2 | 3 | 3 | 3 | 4 |
| Q007 | M06 | Qwen-3.5-plus | R03 | 2 | 4 | 3 | 4 | 4 |
| Q008 | M01 | ChatGPT-5.4 | R01 | 4 | 5 | 3 | 4 | 3 |
| Q008 | M01 | ChatGPT-5.4 | R02 | 4 | 5 | 4 | 4 | 4 |
| Q008 | M01 | ChatGPT-5.4 | R03 | 4 | 5 | 4 | 4 | 4 |
| Q008 | M02 | Gemini-3.1-pro | R01 | 4 | 4 | 5 | 4 | 4 |
| Q008 | M02 | Gemini-3.1-pro | R02 | 4 | 4 | 4 | 4 | 3 |
| Q008 | M02 | Gemini-3.1-pro | R03 | 4 | 4 | 4 | 4 | 4 |
| Q008 | M03 | Claude-4.6-Sonnet | R01 | 4 | 4 | 3 | 4 | 2 |
| Q008 | M03 | Claude-4.6-Sonnet | R02 | 4 | 3 | 3 | 3 | 3 |
| Q008 | M03 | Claude-4.6-Sonnet | R03 | 4 | 3 | 4 | 4 | 3 |
| Q008 | M04 | Deepseek-3.2 | R01 | 3 | 5 | 4 | 4 | 4 |
| Q008 | M04 | Deepseek-3.2 | R02 | 3 | 4 | 4 | 4 | 4 |
| Q008 | M04 | Deepseek-3.2 | R03 | 4 | 5 | 4 | 4 | 5 |
| Q008 | M05 | Kimi-2.5 | R01 | 3 | 4 | 2 | 4 | 2 |
| Q008 | M05 | Kimi-2.5 | R02 | 3 | 4 | 2 | 3 | 3 |
| Q008 | M05 | Kimi-2.5 | R03 | 4 | 4 | 2 | 3 | 3 |
| Q008 | M06 | Qwen-3.5-plus | R01 | 4 | 4 | 3 | 4 | 5 |
| Q008 | M06 | Qwen-3.5-plus | R02 | 3 | 4 | 3 | 4 | 4 |
| Q008 | M06 | Qwen-3.5-plus | R03 | 4 | 4 | 3 | 4 | 5 |
| Q009 | M01 | ChatGPT-5.4 | R01 | 4 | 5 | 4 | 4 | 3 |
| Q009 | M01 | ChatGPT-5.4 | R02 | 4 | 4 | 3 | 4 | 3 |
| Q009 | M01 | ChatGPT-5.4 | R03 | 4 | 4 | 4 | 4 | 3 |
| Q009 | M02 | Gemini-3.1-pro | R01 | 4 | 3 | 5 | 4 | 3 |
| Q009 | M02 | Gemini-3.1-pro | R02 | 4 | 3 | 4 | 4 | 3 |
| Q009 | M02 | Gemini-3.1-pro | R03 | 4 | 3 | 4 | 4 | 3 |
| Q009 | M03 | Claude-4.6-Sonnet | R01 | 4 | 3 | 3 | 4 | 2 |
| Q009 | M03 | Claude-4.6-Sonnet | R02 | 4 | 3 | 3 | 4 | 2 |
| Q009 | M03 | Claude-4.6-Sonnet | R03 | 4 | 3 | 3 | 4 | 3 |
| Q009 | M04 | Deepseek-3.2 | R01 | 4 | 5 | 5 | 4 | 4 |
| Q009 | M04 | Deepseek-3.2 | R02 | 4 | 4 | 4 | 4 | 4 |
| Q009 | M04 | Deepseek-3.2 | R03 | 4 | 4 | 4 | 4 | 4 |
| Q009 | M05 | Kimi-2.5 | R01 | 4 | 4 | 2 | 4 | 3 |
| Q009 | M05 | Kimi-2.5 | R02 | 4 | 4 | 3 | 4 | 3 |
| Q009 | M05 | Kimi-2.5 | R03 | 4 | 4 | 3 | 4 | 3 |
| Q009 | M06 | Qwen-3.5-plus | R01 | 4 | 5 | 4 | 4 | 4 |
| Q009 | M06 | Qwen-3.5-plus | R02 | 4 | 5 | 4 | 4 | 5 |
| Q009 | M06 | Qwen-3.5-plus | R03 | 4 | 5 | 4 | 4 | 5 |
| Q010 | M01 | ChatGPT-5.4 | R01 | 4 | 5 | 4 | 4 | 4 |
| Q010 | M01 | ChatGPT-5.4 | R02 | 4 | 4 | 4 | 4 | 4 |
| Q010 | M01 | ChatGPT-5.4 | R03 | 4 | 4 | 4 | 4 | 4 |
| Q010 | M02 | Gemini-3.1-pro | R01 | 4 | 5 | 4 | 4 | 4 |
| Q010 | M02 | Gemini-3.1-pro | R02 | 4 | 4 | 4 | 4 | 4 |
| Q010 | M02 | Gemini-3.1-pro | R03 | 4 | 4 | 4 | 4 | 4 |
| Q010 | M03 | Claude-4.6-Sonnet | R01 | 3 | 3 | 3 | 3 | 2 |
| Q010 | M03 | Claude-4.6-Sonnet | R02 | 3 | 3 | 3 | 3 | 3 |
| Q010 | M03 | Claude-4.6-Sonnet | R03 | 3 | 3 | 4 | 3 | 4 |
| Q010 | M04 | Deepseek-3.2 | R01 | 4 | 4 | 4 | 4 | 3 |
| Q010 | M04 | Deepseek-3.2 | R02 | 4 | 4 | 4 | 4 | 3 |
| Q010 | M04 | Deepseek-3.2 | R03 | 4 | 4 | 4 | 4 | 4 |
| Q010 | M05 | Kimi-2.5 | R01 | 4 | 4 | 3 | 4 | 3 |
| Q010 | M05 | Kimi-2.5 | R02 | 4 | 4 | 3 | 4 | 3 |
| Q010 | M05 | Kimi-2.5 | R03 | 4 | 4 | 4 | 4 | 4 |
| Q010 | M06 | Qwen-3.5-plus | R01 | 4 | 5 | 4 | 4 | 4 |
| Q010 | M06 | Qwen-3.5-plus | R02 | 4 | 4 | 4 | 4 | 4 |
| Q010 | M06 | Qwen-3.5-plus | R03 | 4 | 5 | 4 | 4 | 5 |
| Q011 | M01 | ChatGPT-5.4 | R01 | 4 | 3 | 5 | 4 | 3 |
| Q011 | M01 | ChatGPT-5.4 | R02 | 4 | 3 | 4 | 4 | 3 |
| Q011 | M01 | ChatGPT-5.4 | R03 | 4 | 3 | 4 | 4 | 4 |
| Q011 | M02 | Gemini-3.1-pro | R01 | 4 | 4 | 4 | 4 | 3 |
| Q011 | M02 | Gemini-3.1-pro | R02 | 4 | 4 | 4 | 4 | 3 |
| Q011 | M02 | Gemini-3.1-pro | R03 | 4 | 4 | 4 | 4 | 4 |
| Q011 | M03 | Claude-4.6-Sonnet | R01 | 4 | 3 | 3 | 4 | 3 |
| Q011 | M03 | Claude-4.6-Sonnet | R02 | 4 | 3 | 3 | 4 | 2 |
| Q011 | M03 | Claude-4.6-Sonnet | R03 | 4 | 3 | 3 | 4 | 3 |
| Q011 | M04 | Deepseek-3.2 | R01 | 4 | 4 | 3 | 4 | 3 |
| Q011 | M04 | Deepseek-3.2 | R02 | 4 | 4 | 4 | 4 | 3 |
| Q011 | M04 | Deepseek-3.2 | R03 | 4 | 4 | 4 | 4 | 4 |
| Q011 | M05 | Kimi-2.5 | R01 | 4 | 4 | 2 | 4 | 2 |
| Q011 | M05 | Kimi-2.5 | R02 | 4 | 3 | 3 | 4 | 2 |
| Q011 | M05 | Kimi-2.5 | R03 | 4 | 4 | 3 | 4 | 4 |
| Q011 | M06 | Qwen-3.5-plus | R01 | 4 | 5 | 4 | 4 | 5 |
| Q011 | M06 | Qwen-3.5-plus | R02 | 4 | 4 | 3 | 4 | 5 |
| Q011 | M06 | Qwen-3.5-plus | R03 | 4 | 4 | 4 | 4 | 5 |
| Q012 | M01 | ChatGPT-5.4 | R01 | 4 | 4 | 3 | 4 | 3 |
| Q012 | M01 | ChatGPT-5.4 | R02 | 4 | 3 | 3 | 4 | 4 |
| Q012 | M01 | ChatGPT-5.4 | R03 | 4 | 4 | 3 | 4 | 3 |
| Q012 | M02 | Gemini-3.1-pro | R01 | 4 | 4 | 4 | 4 | 3 |
| Q012 | M02 | Gemini-3.1-pro | R02 | 4 | 4 | 4 | 4 | 4 |
| Q012 | M02 | Gemini-3.1-pro | R03 | 4 | 4 | 4 | 4 | 3 |
| Q012 | M03 | Claude-4.6-Sonnet | R01 | 3 | 3 | 3 | 3 | 3 |
| Q012 | M03 | Claude-4.6-Sonnet | R02 | 3 | 3 | 3 | 3 | 3 |
| Q012 | M03 | Claude-4.6-Sonnet | R03 | 4 | 3 | 3 | 3 | 3 |
| Q012 | M04 | Deepseek-3.2 | R01 | 4 | 4 | 4 | 4 | 5 |
| Q012 | M04 | Deepseek-3.2 | R02 | 4 | 4 | 4 | 4 | 5 |
| Q012 | M04 | Deepseek-3.2 | R03 | 4 | 4 | 4 | 4 | 5 |
| Q012 | M05 | Kimi-2.5 | R01 | 3 | 3 | 3 | 4 | 3 |
| Q012 | M05 | Kimi-2.5 | R02 | 3 | 3 | 3 | 3 | 4 |
| Q012 | M05 | Kimi-2.5 | R03 | 3 | 4 | 3 | 4 | 3 |
| Q012 | M06 | Qwen-3.5-plus | R01 | 4 | 4 | 3 | 4 | 4 |
| Q012 | M06 | Qwen-3.5-plus | R02 | 4 | 4 | 4 | 4 | 4 |
| Q012 | M06 | Qwen-3.5-plus | R03 | 4 | 4 | 4 | 4 | 5 |
| Q013 | M01 | ChatGPT-5.4 | R01 | 4 | 5 | 4 | 4 | 4 |
| Q013 | M01 | ChatGPT-5.4 | R02 | 4 | 5 | 4 | 4 | 3 |
| Q013 | M01 | ChatGPT-5.4 | R03 | 4 | 5 | 4 | 4 | 4 |
| Q013 | M02 | Gemini-3.1-pro | R01 | 4 | 3 | 4 | 4 | 3 |
| Q013 | M02 | Gemini-3.1-pro | R02 | 4 | 3 | 4 | 4 | 4 |
| Q013 | M02 | Gemini-3.1-pro | R03 | 4 | 3 | 4 | 4 | 3 |
| Q013 | M03 | Claude-4.6-Sonnet | R01 | 3 | 3 | 4 | 4 | 3 |
| Q013 | M03 | Claude-4.6-Sonnet | R02 | 3 | 3 | 4 | 4 | 2 |
| Q013 | M03 | Claude-4.6-Sonnet | R03 | 3 | 3 | 4 | 4 | 3 |
| Q013 | M04 | Deepseek-3.2 | R01 | 3 | 4 | 4 | 4 | 5 |
| Q013 | M04 | Deepseek-3.2 | R02 | 3 | 4 | 3 | 4 | 5 |
| Q013 | M04 | Deepseek-3.2 | R03 | 4 | 4 | 4 | 4 | 5 |
| Q013 | M05 | Kimi-2.5 | R01 | 3 | 4 | 4 | 4 | 4 |
| Q013 | M05 | Kimi-2.5 | R02 | 3 | 3 | 3 | 4 | 5 |
| Q013 | M05 | Kimi-2.5 | R03 | 4 | 4 | 4 | 4 | 5 |
| Q013 | M06 | Qwen-3.5-plus | R01 | 4 | 4 | 4 | 4 | 4 |
| Q013 | M06 | Qwen-3.5-plus | R02 | 4 | 4 | 4 | 4 | 5 |
| Q013 | M06 | Qwen-3.5-plus | R03 | 4 | 4 | 4 | 4 | 5 |
| Q014 | M01 | ChatGPT-5.4 | R01 | 4 | 3 | 4 | 4 | 3 |
| Q014 | M01 | ChatGPT-5.4 | R02 | 4 | 3 | 4 | 4 | 3 |
| Q014 | M01 | ChatGPT-5.4 | R03 | 4 | 4 | 4 | 4 | 3 |
| Q014 | M02 | Gemini-3.1-pro | R01 | 4 | 3 | 4 | 4 | 3 |
| Q014 | M02 | Gemini-3.1-pro | R02 | 4 | 3 | 4 | 4 | 4 |
| Q014 | M02 | Gemini-3.1-pro | R03 | 4 | 3 | 4 | 4 | 3 |
| Q014 | M03 | Claude-4.6-Sonnet | R01 | 4 | 3 | 3 | 4 | 3 |
| Q014 | M03 | Claude-4.6-Sonnet | R02 | 4 | 3 | 4 | 4 | 4 |
| Q014 | M03 | Claude-4.6-Sonnet | R03 | 4 | 3 | 4 | 4 | 3 |
| Q014 | M04 | Deepseek-3.2 | R01 | 4 | 3 | 4 | 4 | 3 |
| Q014 | M04 | Deepseek-3.2 | R02 | 4 | 4 | 4 | 4 | 4 |
| Q014 | M04 | Deepseek-3.2 | R03 | 4 | 4 | 4 | 4 | 4 |
| Q014 | M05 | Kimi-2.5 | R01 | 4 | 3 | 3 | 4 | 2 |
| Q014 | M05 | Kimi-2.5 | R02 | 4 | 4 | 3 | 4 | 4 |
| Q014 | M05 | Kimi-2.5 | R03 | 4 | 4 | 4 | 4 | 3 |
| Q014 | M06 | Qwen-3.5-plus | R01 | 4 | 4 | 4 | 4 | 5 |
| Q014 | M06 | Qwen-3.5-plus | R02 | 4 | 4 | 4 | 4 | 5 |
| Q014 | M06 | Qwen-3.5-plus | R03 | 4 | 4 | 4 | 4 | 5 |
| Q015 | M01 | ChatGPT-5.4 | R01 | 4 | 4 | 3 | 4 | 4 |
| Q015 | M01 | ChatGPT-5.4 | R02 | 4 | 4 | 4 | 4 | 4 |
| Q015 | M01 | ChatGPT-5.4 | R03 | 4 | 4 | 3 | 4 | 3 |
| Q015 | M02 | Gemini-3.1-pro | R01 | 4 | 3 | 4 | 4 | 3 |
| Q015 | M02 | Gemini-3.1-pro | R02 | 4 | 3 | 4 | 4 | 4 |
| Q015 | M02 | Gemini-3.1-pro | R03 | 4 | 3 | 4 | 4 | 3 |
| Q015 | M03 | Claude-4.6-Sonnet | R01 | 4 | 3 | 4 | 4 | 3 |
| Q015 | M03 | Claude-4.6-Sonnet | R02 | 4 | 3 | 3 | 4 | 3 |
| Q015 | M03 | Claude-4.6-Sonnet | R03 | 4 | 3 | 4 | 4 | 3 |
| Q015 | M04 | Deepseek-3.2 | R01 | 4 | 3 | 4 | 4 | 3 |
| Q015 | M04 | Deepseek-3.2 | R02 | 4 | 3 | 4 | 4 | 4 |
| Q015 | M04 | Deepseek-3.2 | R03 | 4 | 3 | 4 | 4 | 3 |
| Q015 | M05 | Kimi-2.5 | R01 | 4 | 4 | 4 | 4 | 2 |
| Q015 | M05 | Kimi-2.5 | R02 | 4 | 4 | 4 | 4 | 3 |
| Q015 | M05 | Kimi-2.5 | R03 | 4 | 4 | 4 | 4 | 3 |
| Q015 | M06 | Qwen-3.5-plus | R01 | 4 | 4 | 4 | 4 | 4 |
| Q015 | M06 | Qwen-3.5-plus | R02 | 4 | 4 | 4 | 4 | 4 |
| Q015 | M06 | Qwen-3.5-plus | R03 | 4 | 4 | 4 | 4 | 5 |
| Q016 | M01 | ChatGPT-5.4 | R01 | 4 | 4 | 3 | 4 | 3 |
| Q016 | M01 | ChatGPT-5.4 | R02 | 4 | 4 | 4 | 4 | 3 |
| Q016 | M01 | ChatGPT-5.4 | R03 | 4 | 4 | 4 | 4 | 4 |
| Q016 | M02 | Gemini-3.1-pro | R01 | 4 | 3 | 4 | 4 | 2 |
| Q016 | M02 | Gemini-3.1-pro | R02 | 4 | 4 | 4 | 4 | 3 |
| Q016 | M02 | Gemini-3.1-pro | R03 | 4 | 3 | 4 | 4 | 3 |
| Q016 | M03 | Claude-4.6-Sonnet | R01 | 4 | 3 | 3 | 4 | 2 |
| Q016 | M03 | Claude-4.6-Sonnet | R02 | 3 | 3 | 4 | 3 | 2 |
| Q016 | M03 | Claude-4.6-Sonnet | R03 | 4 | 4 | 4 | 4 | 3 |
| Q016 | M04 | Deepseek-3.2 | R01 | 4 | 4 | 4 | 4 | 4 |
| Q016 | M04 | Deepseek-3.2 | R02 | 4 | 4 | 4 | 4 | 4 |
| Q016 | M04 | Deepseek-3.2 | R03 | 4 | 4 | 4 | 4 | 4 |
| Q016 | M05 | Kimi-2.5 | R01 | 3 | 4 | 3 | 4 | 3 |
| Q016 | M05 | Kimi-2.5 | R02 | 3 | 4 | 4 | 4 | 4 |
| Q016 | M05 | Kimi-2.5 | R03 | 3 | 4 | 4 | 4 | 3 |
| Q016 | M06 | Qwen-3.5-plus | R01 | 4 | 4 | 3 | 4 | 5 |
| Q016 | M06 | Qwen-3.5-plus | R02 | 4 | 4 | 4 | 4 | 5 |
| Q016 | M06 | Qwen-3.5-plus | R03 | 4 | 4 | 3 | 4 | 5 |
| Q017 | M01 | ChatGPT-5.4 | R01 | 4 | 4 | 4 | 4 | 3 |
| Q017 | M01 | ChatGPT-5.4 | R02 | 4 | 4 | 4 | 4 | 4 |
| Q017 | M01 | ChatGPT-5.4 | R03 | 4 | 4 | 4 | 4 | 4 |
| Q017 | M02 | Gemini-3.1-pro | R01 | 4 | 3 | 4 | 4 | 3 |
| Q017 | M02 | Gemini-3.1-pro | R02 | 4 | 4 | 4 | 4 | 4 |
| Q017 | M02 | Gemini-3.1-pro | R03 | 4 | 3 | 4 | 4 | 3 |
| Q017 | M03 | Claude-4.6-Sonnet | R01 | 4 | 4 | 3 | 4 | 3 |
| Q017 | M03 | Claude-4.6-Sonnet | R02 | 4 | 4 | 4 | 4 | 3 |
| Q017 | M03 | Claude-4.6-Sonnet | R03 | 4 | 4 | 4 | 4 | 3 |
| Q017 | M04 | Deepseek-3.2 | R01 | 4 | 4 | 4 | 4 | 4 |
| Q017 | M04 | Deepseek-3.2 | R02 | 4 | 3 | 4 | 4 | 4 |
| Q017 | M04 | Deepseek-3.2 | R03 | 4 | 4 | 4 | 4 | 3 |
| Q017 | M05 | Kimi-2.5 | R01 | 4 | 5 | 4 | 4 | 3 |
| Q017 | M05 | Kimi-2.5 | R02 | 4 | 4 | 4 | 4 | 4 |
| Q017 | M05 | Kimi-2.5 | R03 | 4 | 4 | 4 | 4 | 4 |
| Q017 | M06 | Qwen-3.5-plus | R01 | 4 | 5 | 4 | 4 | 5 |
| Q017 | M06 | Qwen-3.5-plus | R02 | 4 | 4 | 4 | 4 | 4 |
| Q017 | M06 | Qwen-3.5-plus | R03 | 4 | 4 | 4 | 4 | 5 |
| Q018 | M01 | ChatGPT-5.4 | R01 | 4 | 3 | 4 | 4 | 2 |
| Q018 | M01 | ChatGPT-5.4 | R02 | 4 | 3 | 4 | 4 | 4 |
| Q018 | M01 | ChatGPT-5.4 | R03 | 4 | 3 | 4 | 4 | 3 |
| Q018 | M02 | Gemini-3.1-pro | R01 | 4 | 3 | 4 | 4 | 2 |
| Q018 | M02 | Gemini-3.1-pro | R02 | 4 | 3 | 4 | 4 | 4 |
| Q018 | M02 | Gemini-3.1-pro | R03 | 4 | 3 | 4 | 4 | 3 |
| Q018 | M03 | Claude-4.6-Sonnet | R01 | 4 | 4 | 3 | 4 | 4 |
| Q018 | M03 | Claude-4.6-Sonnet | R02 | 4 | 4 | 4 | 4 | 5 |
| Q018 | M03 | Claude-4.6-Sonnet | R03 | 4 | 4 | 4 | 5 | 4 |
| Q018 | M04 | Deepseek-3.2 | R01 | 4 | 4 | 3 | 4 | 3 |
| Q018 | M04 | Deepseek-3.2 | R02 | 4 | 4 | 4 | 4 | 4 |
| Q018 | M04 | Deepseek-3.2 | R03 | 4 | 4 | 4 | 4 | 3 |
| Q018 | M05 | Kimi-2.5 | R01 | 3 | 3 | 3 | 4 | 2 |
| Q018 | M05 | Kimi-2.5 | R02 | 3 | 4 | 4 | 4 | 4 |
| Q018 | M05 | Kimi-2.5 | R03 | 3 | 3 | 4 | 4 | 3 |
| Q018 | M06 | Qwen-3.5-plus | R01 | 4 | 3 | 4 | 4 | 5 |
| Q018 | M06 | Qwen-3.5-plus | R02 | 4 | 4 | 4 | 4 | 5 |
| Q018 | M06 | Qwen-3.5-plus | R03 | 4 | 4 | 4 | 4 | 4 |
| Q019 | M01 | ChatGPT-5.4 | R01 | 4 | 4 | 4 | 4 | 2 |
| Q019 | M01 | ChatGPT-5.4 | R02 | 4 | 4 | 4 | 4 | 4 |
| Q019 | M01 | ChatGPT-5.4 | R03 | 4 | 4 | 4 | 4 | 3 |
| Q019 | M02 | Gemini-3.1-pro | R01 | 4 | 4 | 4 | 4 | 2 |
| Q019 | M02 | Gemini-3.1-pro | R02 | 4 | 4 | 4 | 4 | 4 |
| Q019 | M02 | Gemini-3.1-pro | R03 | 4 | 4 | 4 | 4 | 3 |
| Q019 | M03 | Claude-4.6-Sonnet | R01 | 3 | 2 | 4 | 4 | 2 |
| Q019 | M03 | Claude-4.6-Sonnet | R02 | 3 | 3 | 4 | 4 | 4 |
| Q019 | M03 | Claude-4.6-Sonnet | R03 | 3 | 3 | 4 | 4 | 3 |
| Q019 | M04 | Deepseek-3.2 | R01 | 4 | 4 | 4 | 4 | 3 |
| Q019 | M04 | Deepseek-3.2 | R02 | 4 | 4 | 4 | 4 | 4 |
| Q019 | M04 | Deepseek-3.2 | R03 | 4 | 4 | 4 | 4 | 4 |
| Q019 | M05 | Kimi-2.5 | R01 | 4 | 4 | 3 | 4 | 3 |
| Q019 | M05 | Kimi-2.5 | R02 | 3 | 4 | 4 | 4 | 4 |
| Q019 | M05 | Kimi-2.5 | R03 | 4 | 3 | 4 | 4 | 3 |
| Q019 | M06 | Qwen-3.5-plus | R01 | 4 | 4 | 3 | 3 | 5 |
| Q019 | M06 | Qwen-3.5-plus | R02 | 4 | 4 | 4 | 3 | 5 |
| Q019 | M06 | Qwen-3.5-plus | R03 | 4 | 4 | 4 | 3 | 3 |
| Q020 | M01 | ChatGPT-5.4 | R01 | 4 | 4 | 4 | 4 | 5 |
| Q020 | M01 | ChatGPT-5.4 | R02 | 4 | 4 | 3 | 3 | 4 |
| Q020 | M01 | ChatGPT-5.4 | R03 | 4 | 4 | 4 | 4 | 5 |
| Q020 | M02 | Gemini-3.1-pro | R01 | 4 | 4 | 4 | 4 | 5 |
| Q020 | M02 | Gemini-3.1-pro | R02 | 4 | 4 | 4 | 4 | 5 |
| Q020 | M02 | Gemini-3.1-pro | R03 | 4 | 4 | 4 | 4 | 5 |
| Q020 | M03 | Claude-4.6-Sonnet | R01 | 4 | 4 | 4 | 4 | 5 |
| Q020 | M03 | Claude-4.6-Sonnet | R02 | 4 | 4 | 4 | 4 | 4 |
| Q020 | M03 | Claude-4.6-Sonnet | R03 | 4 | 4 | 4 | 4 | 3 |
| Q020 | M04 | Deepseek-3.2 | R01 | 4 | 4 | 4 | 4 | 5 |
| Q020 | M04 | Deepseek-3.2 | R02 | 4 | 4 | 4 | 4 | 4 |
| Q020 | M04 | Deepseek-3.2 | R03 | 4 | 4 | 4 | 4 | 4 |
| Q020 | M05 | Kimi-2.5 | R01 | 4 | 4 | 4 | 4 | 3 |
| Q020 | M05 | Kimi-2.5 | R02 | 4 | 4 | 3 | 4 | 3 |
| Q020 | M05 | Kimi-2.5 | R03 | 4 | 4 | 4 | 4 | 3 |
| Q020 | M06 | Qwen-3.5-plus | R01 | 4 | 4 | 4 | 4 | 5 |
| Q020 | M06 | Qwen-3.5-plus | R02 | 4 | 4 | 4 | 4 | 5 |
| Q020 | M06 | Qwen-3.5-plus | R03 | 4 | 4 | 4 | 4 | 5 |

**Table S4.** Patient Satisfaction Scale.

| **Score** | **Satisfaction** |  |
| --- | --- | --- |
| 1 | Very Dissatisfied | The model's response is extremely poor and completely fails to meet the requirements. |
| 2 | Dissatisfied | The model's response is inadequate, with numerous issues and fails to meet expectations. |
| 3 | Neutral | The model's response is acceptable; it addresses some questions but still has room for improvement. |
| 4 | Satisfied | The model's response is acceptable; most questions are effectively answered. |
| 5 | Very Satisfied | The model's response is excellent; all questions are perfectly answered. |

**Table S5.** Patient's questions.

| Patient No. | No. | Question | Category |
| --- | --- | --- | --- |
| 01 | Q1 | Is NMOSD hereditary to the next generation? | Disease Prognosis |
|  | Q2 | If I have NMOSD, can I still work and live normally like before? | Disease Prognosis |
| 02 | Q3 | After being diagnosed with NMOSD, will I have to take medication for the rest of my life? | Long-term Prevention |
|  | Q4 | Is NMOSD contagious to my family or friends? | Disease Prognosis |
| 03 | Q5 | Will NMOSD affect my ability to have children in the future? | Disease Prognosis |
|  | Q6 | Does dietary modification help control NMOSD relapses? | Lifestyle Management |
| 04 | Q7 | Can vision loss caused by NMOSD partially recover? | Disease Prognosis |
|  | Q8 | What impact does pregnancy have on NMOSD? | Disease Prognosis |
| 05 | Q9 | If I have NMOSD, can I still work and live normally like before? | Disease Prognosis |
|  | Q10 | Is NMOSD hereditary to the next generation? | Disease Prognosis |
| 06 | Q11 | Is there currently a curative therapy targeting the cause of NMOSD? | Medication Selection |
|  | Q12 | Is cognitive decline common in NMOSD patients? | Disease Prognosis |
| 07 | Q13 | Could NMOSD cause me to use a wheelchair or become blind in the future? | Disease Prognosis |
|  | Q14 | What should I pay attention to in daily life to reduce the risk of sudden NMOSD relapses? | Lifestyle Management |
| 08 | Q15 | If I feel fine, can I stop or reduce the dosage of NMOSD medication on my own? | Long-term Prevention |
|  | Q16 | With NMOSD, are there many exercises I cannot do, such as running or swimming? | Lifestyle Management |
| 09 | Q17 | Is the relapse frequency of NMOSD related to seasonal changes? | Disease Prognosis |
|  | Q18 | Do biologics for NMOSD need to be used lifelong? | Long-term Prevention |
| 10 | Q19 | Is there currently a curative therapy targeting the cause of NMOSD? | Medication Selection |
|  | Q20 | I heard NMOSD is hard to treat. Should I try traditional Chinese medicine or unproven home remedies? | Medication Selection |

**Table S6.** Patients scoring data.

| **Patient No.** | **No.** | **Gemini-3.1 pro** | **Qwen-3.5 plus** | **Deepseek-3.2** |
| --- | --- | --- | --- | --- |
| **01** | Q1 | 3 | 4 | 3 |
|  | Q2 | 3 | 4 | 4 |
| **02** | Q3 | 4 | 5 | 4 |
|  | Q4 | 3 | 4 | 4 |
| **03** | Q5 | 4 | 5 | 4 |
|  | Q6 | 4 | 5 | 4 |
| **04** | Q7 | 4 | 4 | 4 |
|  | Q8 | 4 | 5 | 4 |
| **05** | Q9 | 4 | 5 | 4 |
|  | Q10 | 4 | 4 | 4 |
| **06** | Q11 | 3 | 4 | 3 |
|  | Q12 | 3 | 5 | 3 |
| **07** | Q13 | 4 | 4 | 4 |
|  | Q14 | 4 | 4 | 4 |
| **08** | Q15 | 3 | 3 | 3 |
|  | Q16 | 3 | 5 | 4 |
| **09** | Q17 | 3 | 4 | 3 |
|  | Q18 | 4 | 5 | 4 |
| **10** | Q19 | 3 | 5 | 4 |
|  | Q20 | 4 | 4 | 4 |

**Table S7** Demographic and clinical characteristics of Phase 2 patients

| **Patient ID** | **Age (years)** | **Sex** | **Disease duration (years)** | **DMT status** | **Optic neuritis** | **Previous attack count** | **Antibodies** |
| --- | --- | --- | --- | --- | --- | --- | --- |
| P01 | 49 | Female | 0.5 | Mycophenolate mofetil | No | 1 | MOG: 1:1280 |
| P02 | 49 | Female | 4.1 | Rituximab ×4 doses | Yes | 0 | AQP4: 1:100 |
| P03 | 47 | Female | 15 | Ofatumumab | Yes | 3 | Negative |
| P04 | 57 | Male | 5 | Rituximab ×4 doses | Yes | 2 | Negative |
| P05 | 49 | Female | 7 | Daratumumab ×13 doses, Inebilizumab ×1 dose | Yes | 2 | Negative |
| P06 | 71 | Female | 8 | Rituximab ×1 dose | Yes | 1 | AQP4: 1:100 |
| P07 | 46 | Female | 4 | Ofatumumab ×4 doses, Rituximab ×3 doses | No | 1 | AQP4: 1:16 |
| P08 | 35 | Female | 1.5 | Azathioprine | No | 0 | MOG: 1:32 |
| P09 | 51 | Female | 1.5 | Mycophenolate mofetil | No | 1 | Negative |
| P10 | 55 | Male | 2 | Mycophenolate mofetil | No | 1 | Negative |

**Table S8.** Random-effect variance components for the Phase 2 patient satisfaction LMM.

| **Variance Component** | **Variance** | **Proportion (%)** |
| --- | --- | --- |
| Question-level | 0.155 | 36 |
| Patient-level | 0.059 | 19 |
| Residual | 0.185 | 45 |
| Total | 0.399 | 100 |

**Table S9a.** Results of Linear Mixed Models for the Three Models.

| **Variable** | **Estimate** | **SE** | **t** | ***P*** |
| --- | --- | --- | --- | --- |
| Gemini-3.1-pro | 3.5 | 0.12 | 30.21 | <0.001 |
| Deepseek-3.2 | 0.25 | 0.13 | 1.93 | 0.061 |
| Qwen-3.5-plus | 0.85 | 0.13 | 6.57 | <0.001 |

**Table S9b.** Pairwise comparisons with Bonferroni correction under Three Models.

| **Comparison** | **Estimate** | **SE** | **t** | ***P*** |
| --- | --- | --- | --- | --- |
| Gemini-3.1-pro vs Deepseek-3.2 | -0.25 | 0.129 | -1.93 | 0.183 |
| Deepseek-3.2 vs Qwen-3.5-plus | -0.85 | 0.129 | -6.57 | <0.001 |
| Deepseek-3.2 vs Qwen-3.5-plus | -0.60 | 0.129 | -4.64 | <0.001 |

**Table S10**

1. NMOSD 是否会遗传给下一代？

Gemini

视神经脊髓炎谱系疾病（NMOSD）通常**不会**直接遗传给下一代。在对患者进行教育时，您可以围绕以下几个核心要点进行解释，以消除他们的顾虑：1. NMOSD不是传统的遗传病**NMOSD是一种中枢神经系统的自身免疫性疾病，而不是像色盲或白化病那样由单一基因突变引起的明确遗传病。绝大多数（97%以上）的NMOSD病例是“散发”的，这意味着患者的家族中通常没有其他成员患有此病。**2. 遗传给子女的概率极低**虽然医学研究表明，某些基因特征（如特定的人类白细胞抗原 HLA 基因型）可能使人具有自身免疫疾病的“易感性”，但这并不等同于遗传发病。父母将NMOSD直接传给孩子的概率极低，家族性NMOSD（即家族中有两人或以上患病）在临床上非常罕见，比例通常不到3%。**3. 发病是多因素共同作用的结果**可以向患者形象地解释：发病需要“土壤”（一定的遗传易感背景）加上“诱因”（环境因素，如病毒感染、过度劳累、压力、吸烟或维生素D缺乏等）。即使孩子继承了某种易感体质，如果没有后天环境因素的触发，免疫系统也不会出错去攻击视神经和脊髓。**4. 重点应放在孕期管理而非遗传担忧**您可以明确告诉患者，他们完全可以拥有健康的下一代，不需要因为担心遗传而放弃生育。但必须向患者强调以下生育相关的实际风险：*	**药物影响**：许多用于控制NMOSD的免疫抑制剂或生物制剂具有致畸风险。*	**复发风险**：妊娠期（尤其是孕早期）和产后（产后3-6个月内）是NMOSD病情复发的高危时段。*	**行动建议**：如果患者有生育计划，必须在病情稳定（通常建议至少稳定6个月以上）的前提下，提前与神经内科、眼科和产科医生沟通，调整为对胎儿安全的药物，并在整个孕期和产后接受严密监测。**总结给患者的话术建议：**“您的病不会像传家宝一样直接传给孩子，孩子得这个病的几率非常非常小，您不用为此过度焦虑。您现在最需要关注的不是遗传问题，而是如果您打算要宝宝，一定要提前告诉我们，我们需要帮您调整用药，确保您在怀孕和坐月子期间病情平稳，这才是对您和宝宝最安全的做法。”

Qwen

您好！作为眼科医师的助手，我为您整理了一份针对**视神经脊髓炎谱系疾病（NMOSD）**患者的遗传咨询教育材料。这份材料旨在帮助医师向患者清晰、科学地解释 NMOSD 的遗传风险，缓解患者的焦虑，同时提供准确的医学信息。患者教育指南：NMOSD 会遗传给下一代吗？#### 1. 核心结论**NMOSD 不是一种典型的“遗传病”，但存在“遗传易感性”。**不会直接遗传：** 父母患有 NMOSD，孩子**不会**像单基因遗传病（如色盲、血友病）那样必然患病。**风险略微增加：** 相比普通人群，NMOSD 患者的直系亲属患自身免疫性疾病的风险略有升高，但**绝对风险仍然很低**。绝大多数 NMOSD 患者的子女终身都不会发病。#### 2. 科学原理解析。为了让您更好地理解，我们可以将 NMOSD 的发病机制看作是一个“多重打击”模型：$$ P(\text{发病}) = f(\text{遗传易感基因}, \text{环境触发因素}, \text{免疫系统状态}) $$*	**遗传易感性（土壤）：** 研究发现，某些人类白细胞抗原（HLA）基因型（如 $HLA-DRB1$ 等位基因）与 NMOSD 相关。患者可能携带这些基因，但这只是意味着他们的免疫系统更容易“出错”，而不是注定会生病。*	**环境触发（种子/气候）：** 仅有基因通常不足以发病。还需要环境因素的触发，例如病毒感染（如 EB 病毒）、疫苗接种、压力或其他未知的免疫刺激。*	**非孟德尔遗传：** NMOSD 不符合显性或隐性遗传规律。它属于**复杂多基因遗传病**。#### 3. 数据参考（供医师参考）根据目前的流行病学研究数据：| 人群类别 | 患病风险估算 | 说明 || **普通人群** | 极低 (约 0.5 - 4 / 10 万) | 基础发病率很低 || **NMOSD 患者的一级亲属** | 低 (略高于普通人群) | 虽然相对风险增加，但绝对概率仍很小 || **同卵双胞胎** | 不一致 | 即使基因完全相同，若一人患病，另一人患病的 concordance rate (一致率) 也远低于 100%，证明环境因素至关重要 |#### 4. 给患者的建议与沟通话术。医师在与患者沟通时，可以参考以下流程和话术：##### 沟通要点1. **安抚情绪：** “请放心，NMOSD 不是那种‘传一代必得一代’的遗传病。”2. **解释机制：** “这就好比您给孩子遗传了一种‘敏感的体质’，但是否真的发病，还取决于后天的环境、感染等多种因素。大多数拥有这种体质的孩子一辈子都很健康。”3. **生育指导：** “目前医学界**不建议**仅仅因为患有 NMOSD 而避免生育。但在计划怀孕前，我们需要评估您的病情稳定性及用药情况。”#####特别注意事项（孕期管理）。虽然遗传风险低，但**孕期管理**对 NMOSD 患者至关重要：*	**复发风险：** 产后（尤其是产后 3-6 个月）是 NMOSD 复发的高危期。*	**药物调整：** 部分免疫抑制剂（如吗替麦考酚酯、甲氨蝶呤）具有致畸性，需在备孕前数月停药或更换为孕期安全药物（如硫唑嘌呤、利妥昔单抗需提前规划）。*	**抗体监测：** 建议在孕前和孕期定期监测 $AQP4-IgG$ 抗体滴度。5. 可视化流程图：遗传风险评估与决策路径。为了更直观地向患者展示决策过程，以下是基于 Mermaid 语法的流程图：6. 总结：作为眼科医师，您可以这样总结告诉患者：> "NMOSD **不会**像单基因病那样直接遗传给孩子。虽然孩子可能继承一些免疫相关的基因特征，导致他们比普通人对自身免疫病稍微敏感一点，但只要注意健康生活、避免特定感染，他们发病的概率是非常低的。我们现在的重点应该放在**您自身的病情控制**以及**科学的备孕计划**上，以确保母婴安全。”*注：本内容仅供医疗教育参考，具体诊疗方案请结合患者个体情况及最新临床指南执行。*

deepseek

您好，很高兴能协助您对NMOSD患者进行教育。这是一个非常重要且常见的问题。首先，请直接回答患者的核心关切：**NMOSD（视神经脊髓炎谱系疾病）本身不是一种传统意义上的“遗传病”，它通常不会像某些单基因疾病那样直接遗传给下一代。*为了帮助您更清晰、全面地向患者解释，我将从以下几个方面展开，您可以根据患者的理解程度和具体需求进行沟通：核心要点解析1. **“非遗传性”的含义**：不是“父母有病，孩子必得”**：NMOSD不是由单一的、可预测的致病基因突变引起的。因此，患者的子女**绝大多数情况下不会发病**。遗传易感性**：目前的研究认为，NMOSD的发病是**遗传易感因素**与**环境因素**（如感染、维生素D水平、肠道菌群等）共同作用的结果。遗传易感性指的是个体可能携带某些基因（如与免疫系统相关的HLA基因），这些基因会**略微增加**患自身免疫性疾病的风险，但远不足以单独致病。2. **关键数据支持（供医师参考）**：*	家族性NMOSD病例非常罕见，在所有NMOSD患者中占比**不到3%**。绝大多数患者（超过97%）都没有明确的家族史。即使有家族史，直系亲属（如子女、兄弟姐妹）的患病风险也**仅略高于普通人群**，但绝对风险仍然很低。具体风险数字尚无精确统计，但可以强调“风险极低”。### 给患者的沟通建议与安抚。您可以这样向患者解释和安抚：“王先生/女士，关于您担心的遗传问题，请您放宽。NMOSD和我们熟知的高血压、糖尿病这类有明确家族聚集性的慢性病不同，它**不属于遗传病**。目前科学界的共识是，它不会直接传给您的孩子。”“您可以这样理解：可能有些人因为体质原因，免疫系统相对容易‘出错’（遗传易感性），但必须在外界某些环境因素的‘触发’下（比如一次严重的感染），才会真正发病。这个‘触发’环节是偶然的、不可预测的，而且大多数人即使有易感体质，一生也不会被触发。所以，您的子女患病的可能性**非常非常低**。”### 需要关注的情况（供医师评估）虽然风险极低，但在极少数情况下，如果患者出现以下情况，可以稍加留意，但不必过度恐慌：*	**有明确的自身免疫性疾病家族史**：如果家族中（不仅限于直系亲属）有多人患有其他自身免疫病（如系统性红斑狼疮、干燥综合征、甲状腺疾病等），这可能提示家族存在一定的免疫异常背景。*	**极罕见的家族性病例**：如果家族中已有不止一位NMOSD患者，这种情况非常罕见，但值得在遗传咨询中提及。### 总结与行动建议：1. **核心结论**：NMOSD**不具有高遗传性**，患者子女的患病风险**极低**，无需因此过度焦虑或影响生育决策。2. **积极管理**：对于患者本人，当前的重点是**规范治疗、控制病情、预防复发**，这远比担心遗传问题更重要。3. **健康生活方式**：可以建议患者的子女保持健康的生活方式（均衡饮食、适度锻炼、充足日照、避免吸烟等），这有助于维持免疫系统平衡，是预防多种疾病（包括自身免疫病）的通用建议。4. **何时咨询**：如果患者家族中有**多位**自身免疫性疾病患者，或患者本人仍非常担忧，可以建议他们咨询**临床遗传咨询师**，获得更个体化的评估和心理支持。**给医师的最终提示**：在沟通时，语气应肯定而温和，用“风险极低”代替“有可能但概率小”，更能缓解患者的焦虑。强调当前可控的“治疗与管理”比不可控的“遗传风险”更有实际意义。希望以上信息对您的患者教育工作有所帮助。

2. 如果得了 NMOSD，我还能像以前一样正常工作和生活吗？

Gemini

很多NMOSD（视神经脊髓炎谱系疾病）患者在经过规范治疗病情稳定后，是可以重返工作岗位并维持良好生活质量的。但“像以前一样正常”通常意味着你需要学会与疾病共存，并对生活和工作方式做出一些积极的调整。您可以从以下几个方面向患者进行教育和解释：**1. 坚持规范治疗，预防复发是核心**NMOSD是一种复发性疾病，每次发作都可能对视神经或脊髓造成不可逆的损伤。能否正常工作和生活，很大程度上取决于复发控制得好不好。只要严格遵医嘱长期使用免疫抑制剂或靶向生物制剂（如序贯治疗），将复发风险降到最低，就能最大程度地保护视力和肢体功能。**2. 适应并管理遗留症状**即使在疾病的缓解期，患者也可能面临一些残留症状，如单眼或双眼视力下降、容易疲劳、神经痛或肢体麻木。*	**视力受损：** 如果视力未能完全恢复，可以通过使用大屏幕、屏幕放大软件、语音朗读工具或特殊的视觉辅助设备来协助办公。*	**疲劳与疼痛：** 慢性疲劳是常见症状。需要学会在精力充沛的时段处理重要工作，并与医生沟通，使用对症药物缓解神经痛。**3. 工作与生活节奏的调整****避免过劳和熬夜：** 极度的疲劳、压力和睡眠不足可能会诱发免疫系统异常，增加复发风险。工作可以做，但“拼命”或长期加班不可取。*	**灵活的工作安排：** 建议患者与雇主沟通，在可能的情况下申请弹性工作制、部分时间居家办公，或调换到体力/视力消耗较小的岗位。*	**预防感染：** 感染（如重感冒、肠胃炎）是NMOSD复发的常见诱因。在日常生活中需要注意个人卫生，去人员密集的场所佩戴口罩。**4. 康复训练与心理支持***	**康复介入：** 尽早且持续的物理治疗、作业治疗或视觉康复训练，有助于最大化地恢复和保持现有功能。*	**心理建设：** 接受这是一种需要终身管理的慢性疾病。面对疾病带来的落差感，出现焦虑或抑郁情绪是很正常的。鼓励患者向家人、医生表达感受，或加入NMOSD病友群获取经验和情感支持。**总结给患者的寄语：**确诊NMOSD意味着你的生活轨迹需要稍微转个弯，但这并不意味着人生的停滞。随着医学的进步，现在有越来越多的有效药物可以控制这种疾病。只要配合医生做好长期管理，量力而行地调整工作节奏，你依然可以拥有一份有价值的工作和充实的生活。

Qwen

您好！作为眼科医师的助手，我非常理解您确诊视神经脊髓炎谱系疾病（NMOSD）后的担忧。这是一个非常关键的问题。**简短的回答是：是的，许多 NMOSD 患者在经过规范治疗和科学管理后，完全能够回归正常的工作和生活。**但是，这通常意味着生活方式需要进行一些**“调整”**和**“优化”**，而不是完全照搬生病前的模式。NMOSD 是一种慢性自身免疫性疾病，主要攻击视神经和脊髓，但通过现代医疗手段，我们可以极大地控制病情。以下是为您准备的患者教育要点，您可以参考这些内容与您的主治医生进一步沟通：### 1. 核心目标：预防复发是关键。NMOSD 的特点是容易复发，每一次复发都可能留下后遗症。因此，**“像以前一样生活”的前提是“不再复发”或“极少复发”**。- **长期免疫治疗**：这是生活的基石。您需要遵医嘱长期服用免疫抑制剂（如硫唑嘌呤、吗替麦考酚酯等）或使用生物制剂（如利妥昔单抗、伊奈利珠单抗等）。- **切勿自行停药**：即使感觉良好，也不能擅自减药或停药，否则复发风险极高。### 2. 工作方面的调整与建议：大多数患者可以继续从事原本的职业，但可能需要根据视力状况和体力进行微调：| 工作情况 | 建议与对策 || **视力受损者** | 如果视神经炎导致视力下降或视野缺损，可申请 workplace accommodation（工作场所便利），如使用大屏幕显示器、放大软件、调整照明或调整座位位置。 || **疲劳感明显者** | NMOSD 患者常伴有中枢性疲劳。建议采用“能量守恒”策略，将重要工作安排在精力最充沛的时段，适当增加休息间隔。 || **高压环境** | 过度的精神压力可能诱发免疫波动。建议学习压力管理技巧，必要时与上级沟通调整工作负荷。 || **职业选择** | 避免从事对视力要求极高且无保护措施的高危职业（如高空作业、精密仪器操作且无法矫正视力者），但绝大多数办公室、教育、服务类工作均可胜任。 |### 3. 生活中的自我管理。为了维持高质量的生活，您需要成为自己健康的“管理者”：- **识别预警信号**：熟悉复发的早期症状（如眼球转动痛、视力模糊、肢体麻木无力、排尿困难等）。一旦出现，**立即就医**，黄金治疗窗口期非常短。- **避免诱因**：- **感染**：感冒、发烧是常见的复发诱因。注意个人卫生，流感季节佩戴口罩，咨询医生是否需要接种疫苗（注意：使用强效免疫抑制剂期间通常禁止接种活疫苗）。- **过度劳累**：保证充足睡眠，避免熬夜。- **极端温度**：部分患者对热敏感（Uhthoff 现象），高温环境下症状可能暂时加重，需注意降温。- **心理健康**：焦虑和抑郁在慢性病患者中很常见。保持积极心态，加入病友互助小组，或寻求心理咨询师的帮助，对整体预后大有裨益。### 4. 眼科随访的重要性

作为眼科患者，定期的眼部检查至关重要：- **监测视神经功能**：定期进行视野检查、光学相干断层扫描（OCT）和视觉诱发电位（VEP）。- **药物副作用监测**：某些免疫抑制药物（如羟氯喹、激素）可能对眼睛有影响，需定期筛查眼底。### 5. 总结与展望：虽然 NMOSD 是一场持久战，但它不再是“绝症”。随着医学的进步，越来越多的患者实现了**“无复发状态”（No Evidence of Disease Activity, NEDA）**。**给您的建议：**不要试图立刻回到生病前“百分之百”的状态，允许自己有一个适应期。与您的神经内科医生和眼科医生建立紧密的合作关系，制定个性化的治疗和康复计划。只要控制好病情，您依然可以拥有精彩的职业生涯和幸福的家庭生活。*注：以上信息仅供参考，具体治疗方案请务必遵循您的主治医师指导。*

Deepseek

\# NMOSD患者生活与工作指导。您好！作为眼科医师，我很理解您对NMOSD（视神经脊髓炎谱系疾病）诊断后的担忧。请放心，虽然NMOSD是一种需要长期管理的慢性疾病，但**绝大多数患者通过规范治疗和积极管理，仍然可以维持良好的生活质量和工作能力**。## 核心信息：您的生活不必被疾病定义### 1. **工作方面*- **可以继续工作**：许多NMOSD患者在疾病稳定期能够继续从事原有工作- **适应性调整**：根据症状（如视力变化、疲劳程度）可能需要适当调整工作节奏或环境- **法律保障**：您享有《残疾人保障法》规定的合理便利权利，可与雇主沟通调整工作安排### 2. **日常生活*- **独立生活能力**：大多数患者在缓解期可以保持日常生活自理- **活动参与**：可以参与适度的社交、休闲和家庭活动- **驾驶考虑**：如果视力或神经系统症状影响驾驶安全，需遵医嘱评估驾驶能力## NMOSD管理的关键策略## 治疗与管理要点1. **规范治疗是关键**- 急性期：及时使用激素、血浆置换等控制炎症- 长期预防：使用免疫抑制剂或生物制剂减少复发风险-定期随访：神经科和眼科定期复查至关重要。2. **症状管理**- 视力问题：配合眼科治疗，使用辅助视觉设备- 疲劳管理：合理安排休息，学习能量节省技巧- 疼痛处理：药物与非药物方法结合3. **生活方式调整**- 均衡饮食，保持健康体重- 适度运动，避免过度疲劳- 压力管理，保持良好心态- 避免感染，减少复发诱因## 支持与资源- **医疗团队**：神经科、眼科、康复科等多学科协作- **患者组织**：加入NMOSD患者支持团体，分享经验- **心理支持**：必要时寻求心理咨询，应对情绪挑战- **家庭支持**：与家人沟通，建立支持系统## 现实期望：虽然NMOSD可能需要您做出一些调整，但现代医学的进步使得:- 80-90%的患者通过治疗可有效控制疾病活动- 复发频率显著降低，严重程度减轻- 长期残疾风险大大减少**最重要的是**：每个人的病程不同，您的主治医师会根据您的具体情况制定个性化管理方案。请保持积极心态，与医疗团队密切合作，您完全有可能继续追求有意义的工作和充实的生活。如果您有具体症状或担忧，请随时与您的神经科医师和眼科医师讨论，他们会为您提供最适合您情况的建议。

3. 被确诊为 NMOSD 后，我是不是一辈子都要吃药？

Gemini

确诊NMOSD（视神经脊髓炎谱系疾病）后，通常情况下，**是的，患者需要长期甚至终身接受药物治疗。**作为医生，向患者解释这个问题时，可以从以下几个核心方面进行教育和心理建设：**1. 为什么要长期吃药？核心是为了“预防复发”**NMOSD是一种中枢神经系统的自身免疫性疾病，它最大的特点就是**高复发率**。每一次复发，免疫系统都会对视神经或脊髓发起新的攻击，导致视力下降（甚至失明）、肢体无力或瘫痪、大小便障碍等。这些神经损伤往往是**不可逆**的，复发次数越多，累积的残疾程度就越重。长期用药（序贯治疗/缓解期治疗）的目的，就是给免疫系统戴上“紧箍咒”，阻止下一次攻击的发生。**2. 感觉“病好了”，也不能擅自停药**很多患者在急性发作期经过激素冲击或血浆置换后，症状明显好转，甚至感觉和正常人一样了。这是一个极易产生误区的阶段。必须让患者明白：**症状消失不代表疾病痊愈**。NMOSD目前无法彻底根治，体内的异常抗体（如AQP4抗体）和免疫反应依然潜伏。擅自减药或停药，是导致NMOSD再次复发的最危险因素。**3. 长期用药的方案是因人而异的**终身服药听起来很有压力，但现在的治疗手段已经非常丰富。*	**传统免疫抑制剂**：如硫唑嘌呤、吗替麦考酚酯等，需要每天口服。*	**新型生物制剂（单克隆抗体）**：如利妥昔单抗、萨特利珠单抗、伊奈利珠单抗等。这类药物预防复发的效果非常好，且很多不需要每天吃药（例如有些是半年输注一次，有些是每个月皮下注射一次），极大地提高了生活便利性。医生会根据患者的抗体状态、经济条件、生育需求以及对药物的耐受性，量身定制最合适的长期方案。**4. 心理建设：把药物当成保护视力的“护盾”**建议引导患者转换心态：不要把长期用药看作是疾病的枷锁，而应将其视为保护视力和身体行动能力的“护盾”。就像高血压、糖尿病患者需要长期用药控制指标一样，只要坚持规范的维持治疗，绝大多数NMOSD患者都可以像普通人一样正常工作、生活和回归社会。需要我为您进一步详细梳理目前常用的几类NMOSD维持期治疗药物（如各类单抗与传统免疫抑制剂）的优缺点，以便您在门诊时为患者做具体方案的科普吗？

Qwen

您好！作为眼科医师，面对刚被确诊为**视神经脊髓炎谱系疾病**（NMOSD）的患者，关于“是否要一辈子吃药”的疑问是非常普遍且关键的。这直接关系到患者的治疗依从性和长期预后。以下是一份为您准备的患者教育沟通指南，旨在用通俗易懂的语言、结合图表，帮助患者理解治疗的必要性和长期性### 给 NMOSD 患者的核心解答**简短回答**：对于绝大多数 NMOSD 患者而言，**是的，通常需要长期甚至终身的维持治疗**。但这并不意味着您每天都要吃很多种药，也不意味着生活质量会一直很差。现代医学的目标是通过药物让疾病“休眠”，让您像正常人一样生活。#### 1. 为什么要长期吃药？（疾病特性）NMOSD 是一种**慢性、复发性**的自身免疫性疾病。它的最大特点是"**复发致残**"。*	**不可逆的损伤**：每一次发作（尤其是视神经炎或脊髓炎发作），都可能造成视神经或脊髓的永久性损伤。*	**累积效应**：第一次发作可能恢复得很好，但第二次、第三次发作后，残留的残疾会累积，可能导致失明或瘫痪。*	**预防胜于治疗**：目前的医疗手段很难修复已经坏死的神经，因此**预防下一次发作**是治疗的核心。我们可以用下面的流程图来展示“不治疗”与“规范治疗”的后果差异：#### 2. “一辈子吃药”的具体含义。当医生说要“长期治疗”时，并不总是指传统的口服药片，方案是多样化的：*	**传统口服免疫抑制剂**：如硫唑嘌呤、吗替麦考酚酯等。确实需要每天服用，需定期监测血常规和肝肾功能。*	**生物制剂**（靶向药）：这是近年来的重大突破。例如利妥昔单抗（Rituximab）、伊奈利珠单抗（Inebilizumab）、萨特利珠单抗（Satralizumab）等。*	**优势**：很多生物制剂是**静脉输液**或**皮下注射**，频率可能是**每 6 个月一次**或**每月一次**，大大减少了每天吞服药片的负担。*	**减药的可能性**：极少数患者在长达数年（如 5-10 年）完全无复发、且各项指标正常的情况下，医生可能会尝试极其谨慎地减量，但**完全停药**的风险依然很高，必须由专科医生严格评估。#### 3. 治疗目标的转变。请告诉患者，我们吃药的目的不是为了“治愈”这个病（目前尚无法彻底根除病因），而是为了**控制**它。| 治疗阶段 | 目标 | 患者感受 || **急性期** | 迅速扑灭炎症，挽救视力/功能 | 大剂量激素冲击，可能有副作用 || **维持期** | **防止复发**，保护剩余功能 | 长期用药，生活回归常态，副作用可控 |#### 4. 常见顾虑与应对策略。患者担心长期吃药通常是因为害怕副作用。您可以这样解释：*	**权衡利弊**：药物确实有副作用（如感染风险增加、肝肾负担等），但**疾病复发导致的失明和瘫痪后果远比药物副作用严重得多**。*	**严密监测**：我们会定期让您复查（血常规、生化、免疫球蛋白等），一旦发现副作用苗头，及时调整方案或换药。*	**生活方式配合**：除了吃药，避免劳累、预防感染、保持心情愉快也能辅助减少复发。###给患者的行动建议清单。为了帮助您更好地管理疾病，建议您做到以下几点：1. **绝不擅自停药**：即使感觉身体很好，也不要自行减药或停药。NMOSD 的复发往往悄无声息，等到出现症状时，损伤可能已经发生。2. **建立健康档案**：记录每次用药时间、复查结果以及任何身体不适。3. **定期随访**：初期：每 1-3 个月复查一次。稳定期：每 3-6 个月复查一次。检查项目通常包括：$AQP4-IgG$ 抗体滴度、血常规、肝肾功能、眼部 OCT 检查等。4. **警惕复发信号**：一旦出现视力下降、眼球转动痛、肢体麻木无力、大小便障碍等症状，**立即就医**，不要等待观察。### 医师寄语> "NMOSD 是一场马拉松，而不是短跑。虽然我们需要长期与药物为伴，但现在的医疗技术已经能让绝大多数患者实现‘零复发’，拥有完整的视力和正常的生活能力。**坚持规范治疗，就是守护您光明的未来**。"希望这份教育材料能协助您更好地与患者沟通，减轻他们的焦虑，提高治疗依从性。如果有具体的药物方案需要讨论，欢迎随时提问。

Deepseek

\## 关于NMOSD长期治疗的必要性说明。首先，请理解NMOSD（视神经脊髓炎谱系疾病）是一种**自身免疫性疾病**，您的免疫系统错误地攻击了视神经和脊髓的保护层（髓鞘）。目前的医学共识是：### 核心治疗原则- **急性期治疗**：使用大剂量激素或血浆置换控制急性发作- **长期预防治疗**：使用免疫抑制剂或生物制剂**预防未来发作**### 关键事实1. **疾病特点**：NMOSD具有**高复发倾向**，约60%患者在1年内复发，90%在3年内复发2. **复发后果**：每次发作都可能导致**不可逆的神经损伤**，累积残疾3. **治疗目标**：通过持续治疗将复发风险降至最低，保护神经功能。### 长期治疗的必要性| 考虑因素 | 说明 || **复发风险** | 停药后复发风险显著增高 || **残疾累积** | 每次复发都可能加重视力损害或肢体功能障碍 || **治疗有效性** | 规律治疗可将年复发率降低70-90% |### 治疗决策的个体化考量。虽然长期治疗是普遍推荐，但具体方案需考虑：- 您的抗体状态（AQP4抗体阳性/阴性）- 既往发作频率和严重程度- 对药物的耐受性和副作用- 年龄、生育计划等个人因素### 好消息1. **治疗选择增多**：现在有多种靶向药物（如萨特利珠单抗、伊奈利珠单抗等）效果显著2. **治疗监测**：通过定期随访和MRI检查，可以评估治疗效果并调整方案3. **生活质量**：有效控制下，大多数患者可以维持良好的生活功能### 给您的建议1. **不要自行停药**：任何治疗调整都应在神经科医生指导下进行2. **定期随访**：通常每3-6个月需要复查一次3. **关注新进展**：NMOSD治疗研究进展迅速，未来可能有更优化的方案**最重要的一点**：请与您的神经科医生保持密切沟通，他们会根据您的具体情况制定最合适的长期管理策略。目前来看，长期维持治疗是保护您免受进一步神经损伤的最有效方法。您还有其他具体担忧或问题吗？我很乐意进一步解释。

4. NMOSD 这种病会传染给我的家人或朋友吗？

Gemini

您可以这样向患者进行通俗易懂的解释：“不会的，NMOSD（视神经脊髓炎谱系疾病）**绝对不会传染**。它不是由病毒、细菌或真菌等病原体引起的传染病，因此无论是一起吃饭、拥抱、握手，还是日常的密切接触，都不会把这种疾病传染给您的家人或朋友。NMOSD实际上是一种**自身免疫性疾病**。简单来说，是您自身的免疫系统出现了紊乱，错误地将体内的正常组织（特别是视神经和脊髓）当成了敌人进行攻击。这完全是身体内部免疫系统的问题，与外界传染无关。所以，您可以完全放下心来，继续与家人和朋友保持正常的亲密互动和社交生活。”需要我为您提供关于NMOSD发病机制或遗传倾向的进一步解释，以便您应对患者可能出现的追问吗？

Qwen

您好！作为眼科医师的助手，我很乐意协助您向视神经脊髓炎谱系疾病（NMOSD）患者及其家属解答关于传染性的疑虑。这是一个非常常见且重要的问题，消除误解有助于减轻患者的心理负担和家庭的社会隔离感。以下是为您准备的患者教育内容要点：### 核心结论：NMOSD **不会** 传染。请明确告知患者及其家人：**视神经脊髓炎谱系疾病（NMOSD）绝对不是一种传染病。**您不需要担心通过以下任何日常接触方式将疾病传给家人或朋友：-握手、拥抱或亲吻-共用餐具、水杯或食物-咳嗽、打喷嚏产生的飞沫-共同居住在同一屋檐下-一般的血液接触（除非是极特殊的医疗操作，但即便如此，致病因子也不是可传播的病原体）### 为什么 NMOSD 不会传染？为了帮助患者更好地理解，我们可以用简单的语言解释其发病机制：1. **自身免疫性疾病**：NMOSD 是一种**自身免疫性疾病**。这意味着患者的免疫系统出现了“识别错误”，误将身体自身的组织（主要是视神经和脊髓中的水通道蛋白 -4，即 $AQP4$）当作敌人进行攻击。这与由细菌、病毒或真菌引起的感染性疾病（如流感、结核病或新冠肺炎）完全不同。后者是因为外来的病原体进入人体并复制传播，而 NMOSD 没有外来病原体。

2. **遗传因素 vs. 传染性**：虽然 NMOSD 不是传染的，但科学研究表明它可能与**遗传易感性**有关。- **非直接遗传**：它不像单基因遗传病那样直接传给下一代。- **易感体质**：某些基因可能让人更容易患上自身免疫病，但这需要环境因素（如病毒感染触发、激素水平变化等）共同作用才会发病。- **结论**：家人拥有相似的基因背景，意味着他们患自身免疫病的*风险概率*可能略高于普通人，但这绝不是因为您“传染”给了他们。### 给患者及家属的建议：作为眼科医生，您可以这样安抚患者：> “您的病是因为身体内部的免疫系统‘迷路’了，而不是因为您携带了某种会传染给别人的细菌或病毒。您可以放心地与家人正常生活、亲密接触。相反，家人的理解、支持和陪伴是您治疗过程中最重要的力量。”希望这些信息能帮助您有效地对患者进行教育，缓解他们的焦虑。如果您需要针对特定症状（如视力下降）的护理建议，也请随时告诉我。

Deepseek

您好，作为眼科医师的助手，我很乐意为您和您的患者解答这个问题。这是一个非常重要且常见的担忧。请您向患者明确传达以下信息：**核心答案：NMOSD（视神经脊髓炎谱系疾病）绝对不会传染给您的家人、朋友或任何其他人。****详细解释（供您向患者说明）：*1. **疾病性质**：NMOSD是一种**自身免疫性疾病**。这意味着患者的免疫系统错误地攻击了自身的健康组织，特别是视神经和脊髓。这与由细菌、病毒等病原体引起的**传染性疾病**有本质区别2. **病因**：NMOSD的发病与多种**非传染性**因素有关，主要包括：*	**自身抗体**：大多数患者体内存在一种叫做“水通道蛋白4抗体”的异常抗体，它是攻击自身中枢神经系统的“元凶”。*	**遗传易感性**：某些基因可能使人更容易患上此类自身免疫病，但**这并非直接遗传病**，不会像感冒或流感那样在人与人之间传播。*	**环境触发因素**：可能包括感染（感染本身可能触发免疫紊乱，但疾病本身不传染）、激素变化等。**您可以这样安慰和指导患者：**	**“请放心，您完全不需要担心会将NMOSD传染给任何人。您可以和家人朋友正常地生活、接触、共餐、拥抱，这没有任何风险。”***	**“您需要关注的是自身的疾病管理，比如定期复诊、坚持用药、注意识别复发的征兆（如视力下降、肢体麻木无力等），并保持良好的生活习惯。”**	**“虽然疾病本身不传染，但保持整体健康、避免感染（如感冒）很重要，因为感染有时可能诱发NMOSD的复发。”****总结给医师的关键点：*消除患者对“传染性”的恐惧，是建立良好医患关系、减轻患者心理负担、促进其积极配合治疗的第一步。强调NMOSD的**自身免疫性**和**非传染性**，可以让患者放下不必要的心理包袱，更专注于疾病本身的治疗与管理。希望这些信息对您的患者教育工作有帮助。

5. NMOSD 会不会影响我以后要小孩？

Gemini

简单来说：**NMOSD（视神经脊髓炎谱系疾病）患者是可以怀孕生小孩的，但必须是“有计划的妊娠”。**在与患者沟通时，您可以从以下几个核心方面为她进行详细解答：**1. 疾病本身不影响生育能力**NMOSD主要影响的是视神经和脊髓，属于中枢神经系统的自身免疫性疾病。它本身通常不会损害女性的生殖系统，也不会降低您怀孕的生理几率。**2. 备孕最关键的是“药物调整”**这是备孕过程中最重要的一环。目前用于控制NMOSD复发的很多免疫抑制剂（如吗替麦考酚酯/骁悉、甲氨蝶呤等）具有致畸风险，或者对胎儿发育有不良影响。*	**行动建议：** 绝对不能在服用这些药物期间意外怀孕，也绝对不能为了怀孕自行停药（这会导致疾病严重复发）。您需要提前半年到一年告诉您的神经内科医生您的生育计划，医生会帮您把药物替换成在孕期相对安全的替代药物。**3. 警惕产后复发风险**	**孕期：** 在怀孕期间，由于孕妇体内免疫系统的自然耐受变化，NMOSD的病情往往是相对稳定甚至处于保护状态的。*	**产后：** 真正的挑战在产后。**产后3到6个月是NMOSD复发的高危期**。因此，分娩后通常需要尽早（甚至在产后几天内）恢复免疫抑制治疗或进行特定的预防性治疗，这可能意味着您无法进行母乳喂养。这一点需要提前做好心理准备。**4. 关于遗传的担忧*很多准妈妈担心会把病传给孩子。NMOSD不是像血友病或白化病那样的单基因遗传病。它是一种自身免疫性疾病，虽然可能存在一定的遗传易感性（即孩子未来患自身免疫疾病的概率比普通人微高一点），但**直接把NMOSD遗传给下一代的概率是非常低的**，绝大多数NMOSD患者的孩子都是完全健康的。**5. 多学科医生的保驾护航**生宝宝不是您一个人的事，需要一个医疗团队的配合。*	**神经内科医生：** 负责调整备孕、孕期和产后的免疫用药，控制疾病不复发。*	**产科医生：** 负责监测胎儿发育和孕妇的产科情况，制定分娩计划。*	**眼科医生：** 我会定期监测您的视力和视神经情况。如果孕期出现任何视力下降、眼球转动痛等症状，需要第一时间联系我。**总结给患者的话：**“您完全可以拥有自己的宝宝，前提是我们要做好充分的准备。现在您需要做的第一件事，就是去和您的神经内科主治医生谈谈您的生育想法，制定一个安全的备孕时间表。”

Qwen

您好！作为眼科医师，在面对视神经脊髓炎谱系疾病（NMOSD）患者关于“生育”的咨询时，我们需要提供既专业又充满人文关怀的解答。NMOSD 确实是一种需要长期管理的自身免疫性疾病，但**它并不意味着您不能拥有自己的孩子**。以下是为您准备的患教内容大纲和详细解释，您可以直接用于与患者的沟通或制作宣教手册。眼科医师给 NMOSD 患者的生育指南#### 1. 核心结论：可以生育，但需“计划先行”NMOSD 本身通常**不会直接导致不孕**。大多数女性患者在病情稳定、药物调整得当的情况下，完全可以顺利怀孕并生下健康的宝宝。关键在于：**切勿意外怀孕**。必须在风湿免疫科和产科医生的共同指导下，制定周密的备孕计划。#### 2. 怀孕期间的主要风险与挑战。虽然可以生育，但 NMOSD 患者在孕期和产后面临特殊的风险，需要重点关注：*	**复发风险的变化**：*	**孕期**：由于孕期体内激素水平变化（特别是雌激素升高），部分患者的免疫系统受到抑制，复发风险可能相对降低或保持稳定。*	**产后**：这是**最高危时期**。分娩后激素水平急剧下降，免疫系统“反弹”，产后 3-6 个月内复发风险显著增加。*	**对胎儿的影响**：*	NMOSD 抗体（AQP4-IgG）理论上可以通过胎盘，但目前研究表明，新生儿发生 NMOSD 的概率极低。*	主要风险来自于**母体的复发**（如严重的视神经炎发作可能导致孕妇视力受损，影响照顾婴儿；或脊髓炎发作导致行动不便）*	**药物致畸性**：*	这是最关键的问题。许多用于治疗 NMOSD 的免疫抑制剂（如吗替麦考酚酯、甲氨蝶呤、环磷酰胺等）具有明确的**致畸性**，必须在备孕前数月停药或更换为孕期安全的药物。#### 4. 常用药物的安全性分类（简表）在向患者解释药物调整时，可以参考下表（*注：具体用药必须遵医嘱*）：| 药物类别 | 常见药物 | 备孕/孕期建议 | 备注 || **禁用/高风险** | 吗替麦考酚酯 (MMF)<br>甲氨蝶呤 (MTX)<br>环磷酰胺 (CTX) | **必须停药** | 需提前 3-6 个月停药，否则有致畸风险。 || **相对安全/可用** | 硫唑嘌呤 (AZA)<br>小剂量泼尼松<br>利妥昔单抗 (RTX)* | **可在医生指导下使用** | RTX 通常建议在受孕前 6-12 个月使用，以覆盖整个孕期。 || **生物制剂** | 伊奈利珠单抗<br>萨特利珠单抗 | **数据有限/需谨慎** | 需根据个体情况权衡利弊，通常建议孕期停用。 |*> 注：利妥昔单抗（Rituximab）半衰期长，通常在计划怀孕前进行一次输注，可保护整个孕期不复发，且药物在孕中期基本代谢完毕。*#### 5. 给患者的特别叮嘱：作为眼科医生，请您特别强调以下几点：1. **视力监测是重中之重**：孕期和产后若出现视力下降、眼球转动痛或视野缺损，必须**立即就医**，不要误认为是孕期疲劳。早期大剂量激素冲击治疗对挽救视力至关重要，且在必要时孕期也可以使用。2. **关于母乳喂养**： *	如果使用的是小剂量激素或硫唑嘌呤，通常允许母乳喂养。 *	如果使用了某些生物制剂或免疫抑制剂，可能需要暂停哺乳。这需要您在产后再次咨询医生。3. **心理支持**：焦虑和压力可能诱发复发。请告诉患者，现代医学已经有很多成功案例，保持乐观心态对母婴都有益。**医师结语示例：**> “女士/先生，NMOSD 并不是生育的‘禁区’。只要我们提前做好规划，把药物调整为对宝宝安全的种类，并在孕期和产后像守护视力一样守护您的免疫系统，您完全有机会成为一个健康的母亲/父亲。接下来，我会帮您联系风湿免疫科和产科专家，我们要一起打一场‘有准备之仗’。”希望这份资料能协助您更好地为患者答疑解惑！如有具体的病例需要讨论，欢迎随时提问。

Deepseek

作为一名眼科医师，我非常理解您在确诊视神经脊髓炎谱系疾病（NMOSD）后，对未来生育计划的关切。这是一个非常重要且常见的问题。我将为您提供清晰、专业的解答，帮助您做出明智的决策。### 核心结论：**可以生育，但需要科学规划和管理。**NMOSD本身**不直接影响**您的生育能力或胎儿的遗传健康。它并非遗传病，通常不会直接遗传给下一代。您最需要关注的核心问题是：**如何在怀孕和产后这一特殊时期，有效管理NMOSD，以最大程度地保障您和宝宝的健康，并降低疾病复发的风险。**### 关键问题解析与行动计划#### 1. NMOSD对妊娠的影响：风险与时机- **复发风险**：研究表明，妊娠期（尤其是孕早期和中期）的复发风险与孕前大致相当或略有降低，但**产后（特别是产后3-6个月）复发风险会显著增加**。这是因为产后体内激素水平剧烈变化和免疫系统重建。- **对胎儿的影响**：NMOSD本身不直接导致胎儿畸形。但**疾病活动（尤其是严重复发）** 可能通过影响母亲健康（如使用大剂量激素）而间接影响妊娠。#### 2. 至关重要的孕前准备：与神经科医生共同制定计划。在您尝试怀孕之前，**必须**与您的神经科医生和产科医生进行详细沟通和规划。这是成功妊娠的基石。- **疾病稳定是前提**：通常建议在计划怀孕前，NMOSD病情保持稳定（无临床复发）**至少6-12个月**。- **药物调整是关键**：- **免疫抑制剂**：许多用于预防复发的药物（如霉酚酸酯、甲氨蝶呤、环磷酰胺）在孕期有潜在风险，**必须在医生指导下提前数月（通常3-6个月）更换或停用**。- **生物制剂**：目前，一些靶向B细胞的生物制剂（如利妥昔单抗、萨特利珠单抗、伊奈利珠单抗）在孕期的数据有限。您需要与医生深入讨论用药利弊。**切勿自行停药**。- **安全药物**：小剂量激素（如泼尼松）和某些免疫抑制剂（如硫唑嘌呤）在孕期相对安全，常被用于维持治疗，但需在严密监测下使用。#### 3. 孕期与产后的管理- **孕期监测**：您将被视为“高危妊娠”，需要神经科和产科医生的**联合密切随访**。定期评估神经功能、视力及胎儿发育情况。- **分娩方式**：NMOSD本身不强制要求剖宫产，分娩方式应根据产科指征和您的身体状况（如脊髓病变后遗症）来决定。- **产后核心策略**：这是预防复发的重中之重。医生通常会在**产后立即（甚至产前就制定好方案）启动或加强预防性治疗**，以应对高复发风险。同时，关于母乳喂养，需要根据您所使用的药物来决定是否安全。### 给您的具体行动建议1. **立即启动对话**：下次复诊时，主动向您的神经科医生提出您的生育意愿和时间规划。2. **组建医疗团队**：确保您的神经科医生与一位熟悉高危妊娠的产科医生建立联系，共同为您管理。3. **完善孕前检查**：在医生指导下，进行全面的孕前评估，包括NMOSD相关抗体（如AQP4-IgG）滴度、神经功能状态等。4. **严格避孕直至准备就绪**：在医疗团队确认您的病情稳定、且用药方案已调整为孕期安全方案之前，请采取可靠的避孕措施。5. **关注心理健康**：焦虑和压力可能影响免疫系统。考虑寻求心理咨询支持，积极管理情绪。### 总结**请放心，通过精心的孕前规划、多学科团队的密切合作以及科学的疾病管理，绝大多数NMOSD女性患者都能成功拥有健康的宝宝。**您当前最重要的一步，就是与您的神经科医生开启这场关于未来计划的对话。您可以这样问医生：“医生，我未来有生育计划，根据我目前的病情，我们该如何制定一个安全的孕前和孕期管理方案？”**免责声明**：以上信息为疾病科普和教育，不能替代专业医疗建议。所有治疗和生育决策都必须在您的主治医生指导下进行。

6. 饮食调整对控制 NMOSD 复发有帮助吗？

Gemini

目前，**没有任何临床证据表明单纯的饮食调整可以直接预防或控制视神经脊髓炎谱系疾病（NMOSD）的复发**。预防NMOSD复发的核心必须依赖规范的医学治疗（如免疫抑制剂或单克隆抗体）。然而，饮食调整在NMOSD患者的综合管理中起着非常重要的辅助作用。您可以从以下几个方面对患者进行教育：**1. 明确饮食与药物的主次关系**必须首先向患者强调，NMOSD是一种严重的自身免疫性中枢神经系统疾病。坚持规范的免疫治疗是控制复发的唯一有效手段。切勿因为尝试某种“偏方”或特定饮食而自行停药或减药。**2. 通过饮食应对药物（特别是糖皮质激素）的副作用**NMOSD患者在急性期或维持期经常需要使用糖皮质激素，合理的饮食可以极大缓解激素带来的副作用，这也是饮食调整最直接的帮助：*	**预防骨质疏松：** 激素会导致钙流失。建议患者增加富含钙的食物（如低脂奶制品、豆制品、绿叶蔬菜）。此外，应关注维生素D水平，维生素D不仅有助于钙吸收，一些研究也提示其对调节自身免疫系统有益。若血液检测提示缺乏，应在医生指导下补充。*	**控制血糖：** 激素容易引发类固醇性糖尿病。建议选择低升糖指数（GI）的碳水化合物（如全谷物、糙米、燕麦），严格限制精制糖和含糖饮料的摄入。*	**控制血压和水肿：** 激素会引起水钠潴留。建议采取低盐饮食，每日食盐摄入量控制在5克以内，避免腌制食品、罐头和加工肉类。*	**体重管理：** 激素会导致食欲增加和向心性肥胖。建议控制总热量摄入，多吃富含膳食纤维的蔬菜以增加饱腹感，并提供充足的维生素。**3. 建议抗炎与平衡饮食**虽然不能直接阻止抗水通道蛋白4（AQP4）抗体的致病作用，但健康的饮食有助于维持良好的整体身体状态，减少其他感染风险（感染往往是NMOSD复发的诱因之一）：*	**优质蛋白质：** 有助于神经修复、维持肌肉量并支持正常免疫功能。建议选择鱼肉、去皮禽肉、蛋类和瘦肉。*	**Omega-3脂肪酸：** 具有一定的全身抗炎作用。建议适量摄入深海鱼（如鲑鱼、鲭鱼）、亚麻籽和核桃，或采用类似“地中海饮食”的模式。*	**关注肠道健康：** 越来越多的医学研究提示肠道微生态与自身免疫性疾病（包括中枢神经系统脱髓鞘疾病）存在关联。摄入充足的膳食纤维有助于维持健康的肠道菌群。**总结给患者的行动建议：告诉患者，最好的“抗复发饮食”就是**均衡、低盐、低糖、富含钙和维生素的饮食**。在服用任何营养补充剂、中药，或尝试极端饮食模式（如绝对断食、生酮饮食等）之前，务必先咨询主治医师，以免增加肝肾负担或干扰免疫抑制剂的药效。

Qwen

您好！作为眼科医师的助手，针对**视神经脊髓炎谱系疾病（NMOSD）**患者的饮食教育，我们需要基于目前的医学证据提供科学、客观且实用的建议。### 核心结论目前**没有确凿的临床证据表明某种特定的“治愈性饮食”可以直接阻止 NMOSD 的复发**。NMOSD 是一种由自身抗体（主要是 AQP4-IgG）介导的严重自身免疫性疾病，其复发的控制主要依赖于规范的免疫抑制治疗（如利妥昔单抗、硫唑嘌呤、吗替麦考酚酯等）。然而，**合理的饮食调整可以作为重要的辅助手段**，通过以下途径间接帮助患者维持病情稳定：1. **管理药物副作用**：长期使用激素或免疫抑制剂带来的代谢问题。2. **降低全身炎症水平**：优化免疫系统环境。3. **改善整体健康状况**：增强抵抗力，减少因感染诱发的复发风险。### 给 NMOSD 患者的饮食指导建议。您可以向患者传达以下具体的饮食策略：#### 1. 抗炎饮食模式（推荐）虽然不能直接治愈 NMOSD，但富含抗氧化剂和 Omega-3 脂肪酸的饮食有助于降低全身慢性炎症。*	**推荐食物**：*	**深海鱼类**：三文鱼、鲭鱼（富含omega-3 脂肪酸）。*	** colorful 蔬果**：蓝莓、菠菜、西兰花（富含维生素和植物化学物）。 *	**全谷物**：燕麦、糙米。*	**健康油脂**：橄榄油、亚麻籽油。*	**避免食物**：*	高糖食品、加工肉类（香肠、培根）、反式脂肪酸（油炸食品）。#### 2. 针对药物副作用的管理。NMOSD 患者常需长期服用糖皮质激素（如泼尼松），这会导致特定的营养需求变化：*	**预防骨质疏松**：激素会加速钙流失。*	**建议**：增加钙和维生素 D 的摄入。*	**食物来源**：牛奶、酸奶、深绿色蔬菜、强化谷物。必要时需遵医嘱补充剂。*	**控制血糖和体重**：*	**建议**：限制精制碳水化合物（白米饭、甜点），选择低升糖指数（GI）食物，严格控制总热量摄入，防止激素性糖尿病和肥胖。*	**血压管理**：*	**建议**：低盐饮食（每日钠摄入< 2.3g，即食盐< 6 g），以预防激素引起的水钠潴留和高血压。#### 3. 关于维生素 D 的特殊关注。多项观察性研究表明，低水平的维生素 D 可能与多种自身免疫性疾病的活动度相关。*	**行动建议**：建议患者定期检测血清 25羟维生素D水平。如果缺乏，应在医生指导下补充，目标通常维持在30-50 ng/mL之间。#### 4. 警惕“伪科学”和极端饮食*	**警告**：市面上流传的“断食疗法”、“极度生酮饮食”或“完全素食”声称能治愈 NMOSD，目前**缺乏高质量证据支持**，且可能导致营养不良，削弱免疫系统，反而增加感染风险（感染是 NMOSD 复发的常见诱因）。*	**原则**：任何极端的饮食改变前，必须咨询主治医生或临床营养师。### 患者教育总结表。为了方便您向患者解释，以下是关键点的总结：| 关注领域 | 建议措施 | 目的 || **核心治疗** | **坚持按时服药** | 饮食不能替代免疫抑制治疗 || **抗炎支持** | 地中海饮食风格（多鱼、多菜、少加工） | 降低全身炎症背景 || **骨骼健康** | 补钙 + 维生素 D + 适度日晒 | 对抗激素副作用，防骨折 || **代谢控制** | 低盐、低糖、控热量 | 预防高血压、高血糖、肥胖 || **感染预防** | 食品安全（煮熟煮透），避免生食 | 减少因感染诱发的复发 |### 医师沟通话术示例> “王先生/女士，关于饮食，我想明确告诉您：目前没有哪种特效食物能直接‘吃掉’NMOSD 或保证不复发。**控制复发的基石依然是我们制定的免疫治疗方案。**> 但是，吃得好非常重要。因为您长期服用激素，我们需要通过饮食来保护您的骨头、控制血糖和血压，同时通过健康的饮食让身体处于一个‘低炎症’的状态，减少生病感染的机会。毕竟，一次严重的感冒或腹泻都可能成为复发的导火索。所以，均衡营养、清淡饮食是您最好的‘助攻’。” 希望这些信息能协助您更好地对患者进行宣教！如果有具体的病例需要讨论，欢迎随时提问。

Deepseek

\# 饮食调整与NMOSD复发控制的关系。根据目前的医学研究，**饮食调整可能对NMOSD（视神经脊髓炎谱系疾病）患者的整体健康和疾病管理有辅助作用，但尚未有充分证据表明特定饮食可以直接预防或控制NMOSD复发**。## 当前科学认知- **尚无特定“NMOSD饮食”**：目前没有高质量临床研究证明某种饮食方案（如无麸质、特定补充剂等）能直接降低NMOSD复发率。- **免疫调节作用**：均衡营养有助于维持免疫系统平衡，可能间接影响疾病进程。- **共病管理**：良好饮食有助于控制肥胖、高血压、糖尿病等，这些共病可能影响NMOSD的整体治疗。## 建议的饮食原则1. **均衡营养**：推荐地中海饮食模式（富含蔬菜、水果、全谷物、优质脂肪）。2. **维生素D补充**：部分研究表明维生素D缺乏可能与自身免疫疾病活动相关，适量补充可能有益（需监测血药浓度）。3. **Omega-3脂肪酸**：来自鱼类、坚果的Omega-3具有抗炎特性。4. **限制加工食品**：减少高糖、高盐、反式脂肪摄入。5. **充足水分**：尤其对有膀胱功能障碍的患者重要。## 重要提醒- **不能替代药物治疗**：饮食调整**绝不能**替代免疫抑制剂/生物制剂等标准治疗。- **个体化差异**：食物敏感性因人而异，建议记录饮食与症状日记。- **专业指导**：建议咨询营养科医师或注册营养师制定个性化方案。## 推荐患者沟通要点> “健康饮食有助于您的整体健康，可能为药物治疗提供更好的基础，但目前没有证据表明单纯通过饮食可以控制NMOSD复发。请务必继续遵医嘱进行规范治疗，并将饮食调整作为辅助措施。”**建议患者**：在保持规范治疗的同时，可考虑咨询营养科制定个性化饮食方案，并定期与神经免疫专科医师沟通任何饮食改变。*注：以上信息基于当前医学证据，随着研究进展可能会有更新。*

7. NMOSD 导致的视力损伤能否部分恢复？

Gemini

这份为NMOSD（视神经脊髓炎谱系疾病）患者准备的教育材料，旨在用通俗易懂、客观且带有同理心的语言，帮助您向患者解释视力恢复的可能性及后续注意事项。您可以直接作为口头宣教的参考，或打印成文字资料发给患者。### NMOSD导致的视力损伤能否恢复？——致患者的一封信。面对NMOSD引起的视力突然下降，感到焦虑和害怕是非常正常的。关于您最关心的问题：“我的视力还能恢复吗？” **直接的答案是：视力是有可能实现部分恢复的，但恢复的程度因人而异。**为了帮助您更好地理解视力的恢复过程，我们需要了解以下几个关键点：#### 1. 视力恢复的潜力取决于“救火”的速度。NMOSD发作时，您的免疫系统错误地攻击了视神经，就像视神经上燃起了“大火”。*	**急性期治疗：** 我们在发病初期为您使用的大剂量激素冲击治疗，或者血浆置换（PLEX）、静脉注射免疫球蛋白（IVIG），目的就是以最快速度“扑灭大火”，减轻神经水肿。*	**治疗越早，恢复希望越大：** 炎症控制得越快，视神经受到的永久性损伤就越小，后续视力部分恢复的可能性就越高。#### 2. 神经的修复是一个缓慢的过程*	**不要期望一蹴而就：** 炎症消退后，受损的视神经需要时间来修复。视力的改善通常不会在几天内完成，而是会在发病后的**几周到几个月内**逐渐显现。*	**前6个月最关键：** 大多数的视力恢复发生在发病后的前3到6个月。在这个阶段之后，视力通常会逐渐稳定下来。#### 3. 建立合理的康复预期。NMOSD对视神经的攻击通常比一般的多发性硬化（MS）更猛烈。因此，我们需要对“恢复”有一个现实的预期：*	**部分恢复是常态：** 很多患者的视力可以从发病时的极度模糊恢复到能够看清较大物体的程度，但可能很难完全恢复到发病前100%的完美状态。*	**视力表之外的症状：** 即使您在视力表上能看清更多的字母，您可能仍然会感觉到：*	**颜色变暗淡：** 特别是对红色的辨别能力下降。*	**对比度下降：** 在光线较暗或阴雨天时看东西更吃力。*	**视野缺损：** 视野的某个角落或中心可能会有固定的暗点或模糊区域。#### 4. 最重要的一步：预防下一次“火灾”这是眼科医生和神经内科医生最希望您记住的一点：**NMOSD的视神经损伤是累加的。***	每一次复发，都会在原有的基础上造成新的、更难恢复的损伤。*	**坚持长期的免疫抑制治疗**（如利妥昔单抗、依库珠单抗、吗替麦考酚酯、硫唑嘌呤等）是保护您现有视力、防止另一只眼睛受累的唯一有效方法。**千万不要因为觉得视力好转了，或者觉得视力无法恢复了，就自行停药。**#### 5. 我们可以做些什么来适应和改善？*	**保护好眼：** 如果您是一侧眼睛受损，请在日常生活中（尤其是运动或做家务时）佩戴防护眼镜，保护好另一只健康的眼睛。*	**低视力康复：** 如果残留的视力损伤影响了生活，我们可以为您转诊至低视力康复专家。通过使用放大镜、特殊的照明设备或屏幕阅读软件，可以大幅提高您的生活质量。*	**定期复查：** 请务必按照医嘱定期进行眼科（复查视力、视野、OCT等）和神经内科的随访。**总结：** 视神经的损伤虽然严重，但并非毫无希望。请保持耐心，积极配合急性期治疗，并严格坚持长期的预防复发用药。我们医疗团队会一直在这里协助您应对这些挑战。**给医师的提示：**如果需要进一步强调某种特定免疫抑制剂的重要性，或者需要针对双眼同时受累的重症患者调整预期（这类患者恢复难度通常更大），您可以根据患者的具体临床表现对上述内容进行微调。如果您需要针对特定症状（如色觉丧失）的更详细解释，请告诉我。

Qwen

您好！作为 AI 聊天机器人，我很乐意协助您为视神经脊髓炎谱系疾病（NMOSD）患者提供关于视力恢复的教育信息。以下内容专为眼科医师设计，可用于患者宣教材料或面对面沟通的参考指南。患者教育核心信息：NMOSD 导致的视力损伤能否部分恢复？#### 1. 核心结论**是的，NMOSD 导致的视力损伤是有可能部分甚至显著恢复的，但这取决于多个关键因素。**与多发性硬化症（MS）相比，NMOSD 引起的视神经炎通常发作更急、症状更重，且自发恢复的能力较差。然而，通过**及时、强化的急性期治疗**以及**长期的预防复发管理**，许多患者能够挽回部分视力。#### 2. 影响视力恢复的关键因素。为了帮助患者理解预后，我们可以将影响因素归纳为以下几点：*	**治疗时机（最关键）**：*	“时间就是视力”。在症状出现后的**黄金窗口期**（通常建议发病后 3-5 天内，最迟不超过 2 周）启动大剂量糖皮质激素冲击治疗，是挽救视功能的关键。*	若激素治疗效果不佳，需尽早联合血浆置换（PLEX）或免疫吸附治疗。*	**发作的严重程度**：*	初始视力丧失越严重（如仅存光感或无光感），完全恢复的难度越大，但仍有部分恢复的希望。*	**复发频率**：*	NMOSD 具有极高的复发率。**每一次复发都可能导致累积性的、不可逆的视力损伤**。因此，长期服用免疫抑制剂（如利妥昔单抗、硫唑嘌呤等）预防复发，比单次急性期的治疗对保护长远视力更为重要。*	**视神经萎缩程度**：*	如果在慢性期视神经已经发生严重萎缩，恢复潜力将非常有限。#### 3. 视力恢复的时间线与过程。患者需要了解恢复是一个渐进的过程，而非一蹴而就：*	**急性期（0-2 周）**：主要目标是抑制炎症，阻止进一步损伤。*	**亚急性期（2 周 -3 个月）**：这是视力恢复的主要窗口期。部分髓鞘可能修复，水肿消退，视力逐渐提升。*	**慢性期（3 个月后）**：视力趋于稳定。如果此时仍有视力障碍，通常意味着发生了永久性的轴突丢失（视神经萎缩）。#### 4. 给患者的具体建议（医师话术参考）。您可以这样向患者解释：> “虽然 NMOSD 引起的视神经炎很凶险，但请不要放弃希望。> 1. **关于恢复**：很多患者在经过规范的冲击治疗和血浆置换后，视力都能得到不同程度的回升。即使不能回到发病前的水平，保留现有的残余视力并通过康复训练适应生活也是非常重要的目标。> 2. **关于预防**：我们要把重点放在‘防止下一次发作’上。因为 NMOSD 的特点是‘一次比一次重’，只要不复发，现有的视力就能得到最好的保护。请务必遵医嘱长期服药，不要擅自停药。> 3. **关于康复**：如果遗留了视野缺损或对比度下降，我们可以转介低视力门诊，通过助视器和生活技巧训练，提高您的生活质量。”#### 5. 数据支持（供医师参考）根据临床研究数据显示：*	未经充分治疗的严重 NMOSD 视神经炎，约 **50%-60%** 的患者最终视力可能低于 $0.1$（法定盲）。*	接受早期积极治疗（激素 + 血浆置换）的患者，视力改善率显著提高，部分患者可恢复至 $0.5$ 以上。*	复发是导致永久性失明的主要原因，有效的预防治疗可将年复发率降低 **70%-90%**。#### 医师提示：在向患者传达这些信息时，请保持**“谨慎乐观”**的态度。既要让患者认识到疾病的严重性和复发的危害，又要让他们明白现代医学手段可以有效帮助恢复视力和控制病情。建立医患之间的信任联盟，是提高治疗依从性、改善预后的基石。如果您需要针对特定病例的详细治疗方案讨论，或需要生成具体的患者宣教单页，请随时告诉我。

Deepseek

好的，非常乐意为您提供一份面向NMOSD患者的教育材料。以下内容力求清晰、准确且富有支持性，您可以根据患者的具体情况进行调整和补充。### **与NMOSD患者谈视力恢复：希望与现实的平衡**您好，我们理解您对视力问题的担忧。视神经脊髓炎谱系疾病（NMOSD）确实常攻击视神经，导致视力下降、视野缺损甚至失明。关于视力能否恢复，这是一个需要分情况讨论的问题，核心在于 **“及时、强效的治疗”** 和 **“神经损伤的性质”**。#### **一、 好消息：部分恢复是可能的****是的，许多患者的视力损伤可以得到部分甚至显著的恢复。** 这主要发生在以下情况：1. **急性期治疗及时且充分**：当视神经炎新发时，视神经的损伤主要是由于炎症和水肿（肿胀），神经纤维本身可能尚未完全、不可逆地断裂。*	**治疗关键**：立即使用大剂量静脉糖皮质激素（如甲泼尼龙）进行冲击治疗，以快速抑制炎症。对于重症或效果不佳者，可能需尽早进行血浆置换或免疫吸附治疗，清除血液中的有害抗体（如AQP4抗体）。*	**恢复潜力**：在这个“窗口期”内迅速控制炎症，水肿消退后，被压迫但未死亡的神经纤维功能可能恢复，视力随之改善。**治疗越早，恢复的希望越大。**2. **损伤以“脱髓鞘”为主**：NMOSD主要破坏视神经的髓鞘（包裹神经纤维的绝缘层）。如果神经纤维的轴心（轴索）相对完好，那么髓鞘修复后，神经信号传导可以部分重建。*	**类比**：就像一段电线的外皮破损导致短路，但里面的铜丝还好。修复外皮后，电流可以重新通过。#### **二、 现实的挑战：恢复并非总是完全**视力恢复的程度因人而异，并受以下因素限制：1. **神经纤维的永久性损伤**：如果炎症非常严重或持续时间长，导致神经纤维的轴索本身断裂、坏死，这种损伤是**不可逆的**。坏死的神经纤维无法再生，其对应的视觉功能将永久丧失。2. **反复发作与累积损伤**：NMOSD是一种易复发的疾病。每次视神经炎发作都可能造成新的损伤。反复发作会导致损伤累积，即使每次都有部分恢复，但总体视力可能呈阶梯式下降。因此，**预防复发至关重要**。3. **恢复的平台期**：视力恢复主要发生在急性发作后的**最初3-6个月内**。之后恢复速度会明显减慢，通常在1年后达到一个稳定状态。此后的进一步改善可能非常有限。#### **三、 您现在应该关注的重点：一个综合管理策略**作为您的医疗团队，我们的目标是 **“最大程度恢复本次发作的视力”** 和 **“绝对预防未来的发作”**。1. **急性期：全力出击***	一旦出现新的视力下降、眼痛或视野缺损，**请立即就医**，不要等待。时间就是视力。*	遵从医嘱，完成足量、足疗程的急性期治疗。2. **缓解期：坚守防线（预防复发）***	**长期免疫抑制治疗**：这是NMOSD管理的基石。需要长期服用药物（如吗替麦考酚酯、硫唑嘌呤、甲氨蝶呤等）或使用生物制剂（如依库珠单抗、萨特利珠单抗、伊奈利珠单抗等）来抑制免疫系统，防止攻击视神经和脊髓。**切勿自行停药或减量。***	**定期随访与监测**：定期复查视力、视野、眼底，以及必要的血液和影像学检查（如脊髓MRI），以便评估疗效和及时发现无症状的活动。3. **康复与适应***	**视觉康复**：即使有永久性视力损伤，视觉康复治疗可以帮助您更好地利用剩余的视力，学习使用助视器（如放大镜、电子阅读器），改善日常生活能力。*	**心理支持**：面对视力变化，产生焦虑、抑郁情绪是正常的。寻求心理咨询或加入患者支持团体非常有帮助。#### **总结给患者的关键信息**希望在于“快”**：新发视力下降，**立即治疗**是争取恢复的最好机会。*	**核心在于“防”**：坚持长期预防复发的治疗，是保护现有视力、避免进一步损伤的**最重要手段**。现在的预防性药物比过去有效得多。*	**恢复是个过程**：请给身体一些时间。在发作后的几个月内积极配合治疗和康复。*	**与您的医生结成伙伴**：定期沟通，告知任何变化，共同制定和调整治疗计划。**最后，请记住，您不是一个人在战斗。** 现代医学对NMOSD的认识和治疗正在飞速进步。通过医患紧密合作，我们的目标是控制疾病，最大限度地保护您的神经功能，维护您的生活质量。**（给医师的备注）**：在沟通时，请结合患者具体的视力损伤程度、发作次数、治疗反应及AQP4抗体状态进行个性化解释。对于已发生严重萎缩的视神经，需更强调预防复发的极端重要性，同时引导患者关注视觉康复和心理适应。

8. 怀孕对 NMOSD 病情有什么影响？

Gemini

这是一份为您准备的NMOSD（视神经脊髓炎谱系疾病）患者孕期教育指南，您可以直接用于向患者解释或作为健康宣教资料发放：### NMOSD患者生育指南：怀孕对病情有什么影响？对于NMOSD患者来说，怀孕是一个需要谨慎对待但并非不可触及的愿望。在科学、规范的医学管理下，许多NMOSD患者都能安全度过孕期并迎来健康的宝宝。以下是您需要了解的核心信息：#### 1. 怀孕本身会加重病情吗？*	**孕期（相对稳定但需警惕）：** 在怀孕期间，为了保护胎儿不被母体排斥，女性体内的免疫系统会发生微妙的改变（免疫耐受）。部分患者在孕中晚期病情可能相对稳定，但这并不意味着“绝对安全”，孕期仍有复发的可能。*	**产后（复发高危期）：** **这是整个生育过程中最危险的阶段。** 分娩后的前3到6个月，母体免疫系统会经历一次“重置”和反弹，导致NMOSD复发的风险显著增加。因此，产后的病情监测和及时恢复用药至关重要。#### 2. NMOSD会影响宝宝的健康吗？*	NMOSD本身不是遗传病，虽然有极低的易感基因倾向，但直接遗传给宝宝的概率非常小。*	如果您的病情在孕期控制得很好，通常不会增加胎儿畸形的风险。*	**风险在于“疾病复发”：** 如果在孕期疾病处于活动期或发生严重复发，可能会增加流产、早产、先兆子痫（妊娠高血压）以及胎儿生长受限的风险。此外，孕期复发时使用大剂量激素冲击治疗，也可能对孕妇和胎儿带来一定副作用。#### 3. 药物问题：备孕和孕期最大的挑战。怀孕对NMOSD管理最大的影响在于**药物的调整**。*	**致畸药物必须提前停用：** 许多控制NMOSD的常用免疫抑制剂（如吗替麦考酚酯/骁悉、甲氨蝶呤等）对胎儿有明确的致畸作用，必须在备孕前数月（通常要求提前3-6个月）停药或替换。*	**替代药物：** 医生会根据您的具体情况，将药物替换为孕期相对安全的类型（如硫唑嘌呤、某些特定的生物制剂或小剂量激素）。*	**绝对禁忌：** **千万不要因为想要怀孕而自行突然停药！** 骤然停药极易引发NMOSD的严重复发，对视神经和脊髓造成不可逆的损伤，也会直接威胁妊娠安全。#### 4. 给您的核心建议：如果您有生育计划，请务必遵循以下步骤：*	**计划妊娠：** 怀孕必须是“计划内”的。建议在病情完全稳定、**至少6到12个月没有任何复发迹象**的情况下再开始备孕。*	**多学科联合随访：** 您的孕期需要眼科医生、神经内科医生和产科医生共同保驾护航。*	**提前规划产后治疗：** 在分娩前，您的医生团队就会与您商定产后的治疗方案。为了防止产后复发，通常需要在分娩后尽快恢复免疫抑制治疗。这可能会影响您是否能够进行母乳喂养（因为许多药物会进入乳汁），需要您和医生共同权衡利弊。**总结：** 怀孕是一场需要精心筹备的战役。只要做到“病情稳定再备孕、医生指导调药物、产后及时防复发”，您完全有机会拥有一个健康的宝宝和稳定的未来。如果您需要针对某些特定的免疫抑制剂（如利妥昔单抗、萨特利珠单抗等）在孕期的具体使用建议，我可以进一步为您补充。

Qwen

您好！作为眼科医师，在面对视神经脊髓炎谱系疾病（NMOSD）的女性患者时，关于“怀孕与病情关系”的教育至关重要。NMOSD 是一种高复发率、高致残率的自身免疫性疾病，而怀孕期间的免疫状态变化会对病情产生复杂影响。以下是为您准备的**患者教育要点**，您可以直接用于临床沟通或制作宣教材料：### 怀孕对 NMOSD 病情的影响：核心事实#### 1. 孕期复发风险的变化：怀孕本身并不一定会导致 NMOSD 恶化，但风险分布具有明显的**时间特异性**：*	**妊娠期（尤其是中晚期）：** 由于母体免疫系统发生生理性抑制（为了容忍胎儿），大多数研究表明，**孕期内的复发风险相对较低**，甚至低于非孕期。*	**产褥期（产后 3-6 个月）：** 这是**最高危时期**。随着分娩后免疫系统迅速反弹（Rebound effect），复发风险显著增加。据统计，产后前 3 个月的复发风险是非孕期的 **3-5 倍**。#### 2. 复发的严重后果。对于 NMOSD 患者，每一次复发都可能导致不可逆的神经功能损伤：*	**视力损害：** 视神经炎复发可导致永久性视力下降甚至失明。*	**运动与感觉障碍：** 脊髓炎复发可能导致瘫痪、大小便失禁或感觉异常。*	**累积残疾：** 多次复发会导致残疾程度呈阶梯式加重。#### 3. 药物管理的关键性*	**停药风险：** 许多患者在备孕或发现怀孕后自行停用免疫抑制剂，这是导致孕期或产后复发的主要原因。*	**用药平衡：** 需要在“控制病情”和“胎儿安全”之间找到平衡点。部分药物（如利妥昔单抗、硫唑嘌呤等）在特定阶段可能是相对安全的，而另一些（如吗替麦考酚酯、甲氨蝶呤）则具有明确的致畸性，必须提前停药清洗。###孕期与产后复发风险趋势图。###给患者的具体建议（医师话术参考）您可以这样对患者说：> “王女士，关于怀孕和您的 NMOSD 病情，我们需要达成几个共识：> 1. **不要盲目停药：** 很多患者担心药物伤宝宝而擅自停药，但这往往会导致您在怀孕期间或刚生完孩子时发作。一旦发作，不仅您可能面临失明或瘫痪的风险，剧烈的炎症反应和高剂量急救激素对宝宝的伤害可能比维持治疗的药物更大。> 2. **警惕‘产后风暴’：** 怀孕中期您可能会感觉很舒服，但这不代表病好了。真正的危险期是**生完孩子后的头三个月**。我们需要提前制定好产后的预防方案（如尽早恢复用药或使用静脉注射免疫球蛋白等）。> 3. **多学科协作（MDT）：** 您的怀孕计划必须由**眼科、神经内科和高危产科医生**共同管理。我们在您怀孕前就会调整药物方案，换成对胎儿相对安全的药物，并观察一段时间确保病情稳定后再受孕。> 4. **母乳喂养：** 大多数情况下，在使用特定安全药物的前提下是可以母乳喂养的，但这需要我们要根据您具体的用药方案来个案评估。”备孕与孕期管理检查清单。为了方便患者执行，建议提供以下简表：| 阶段 | 关键行动 | 注意事项 || **备孕前 (3-6 个月)** | ✅ 病情评估：确保无复发至少 6-12 个月<br>✅ 药物调整：停用致畸药，换用安全药<br>✅ 抗体检测：复查 AQP4-IgG 滴度 | 严禁自行停药；补充叶酸 || **妊娠期间** | ✅ 定期随访：每 trimester 评估一次神经功能<br>✅ 监测视力：如有视野缺损立即就医<br>✅ 产科监测：关注胎儿发育 | 避免感染（感染是常见诱发因素） || **分娩及产后** | ✅ 预防性治疗：产后立即启动预防复发方案<br>✅ 密切观察：产后首月每周自我评估<br>✅ 哺乳决策：根据药物半衰期决定是否哺乳 | **产后 3 个月是红色警戒期** |特别提示：如果患者在孕期出现以下症状，需**立即**就诊，不可等待：*	单眼或双眼视力突然下降、眼球转动痛。*	肢体麻木、无力，尤其是双下肢对称性无力。*	排尿困难或尿潴留。*	顽固性呃逆或恶心呕吐（延髓极后区综合征表现）。通过科学的规划和严密的管理，绝大多数 NMOSD 女性患者都能拥有健康的宝宝，同时保护好自己的视力和神经功能。

Deepseek

\# 怀孕对NMOSD病情的影响。您好，作为眼科医师，您可以向NMOSD（视神经脊髓炎谱系疾病）患者提供以下关于怀孕与病情关系的教育信息：## 主要影响与风险：1. **复发风险变化**：- **孕期（尤其孕晚期）**：复发风险可能**轻度增加**，但研究数据不完全一致。- **产后前3个月**：复发风险**显著增加**（约为孕前的2-3倍），需特别关注。- **孕中期**：部分研究显示复发风险可能**降低**。2. **疾病活动度影响**：- 若怀孕前1-2年病情稳定，孕期总体风险相对可控。- 若怀孕前疾病活动度高，孕期及产后风险可能增加。## 对母婴的影响。- **对母亲**：NMOSD复发可能导致视力下降、肢体无力、感觉异常等症状，需及时干预。- **对胎儿**：多数研究表明NMOSD本身不直接增加胎儿畸形风险，但疾病活动及某些药物可能产生影响。## 关键管理建议：1. **孕前计划**：- 建议在病情稳定（通常建议≥1年无复发）后计划怀孕。- **孕前咨询神经科、产科及眼科医师**，进行多学科评估。2. **药物管理**：- **免疫抑制剂调整**：部分药物（如霉酚酸酯、甲氨蝶呤）需在孕前停药（通常提前3-6个月）。- **允许孕期使用的药物**：如糖皮质激素、部分单抗类药物（如利妥昔单抗、依库珠单抗等）可能在医师指导下使用，需权衡利弊。- **绝对避免**：未经医师评估自行停药或换药。3. **监测与随访**：- **孕期**：定期神经科、产科及眼科随访，监测症状变化。- **产后**：加强监测（尤其前6个月），考虑预防性治疗降低复发风险。4. **分娩与哺乳**：- 分娩方式根据产科指征决定，NMOSD通常不影响自然分娩。- 哺乳期用药需评估药物安全性，部分生物制剂可能允许哺乳。## 患者教育要点- **记录症状**：如出现视力下降、眼痛、肢体麻木无力、平衡障碍等，及时就医。- **心理支持**：怀孕可能带来焦虑，建议寻求心理支持或加入患者支持团体。- **个体化方案**：每位患者情况不同，治疗方案需根据具体病情、药物使用史等制定。## 总结。怀孕对NMOSD病情的影响因人而异，但通过**孕前规划、多学科协作及密切监测**，多数患者可以安全度过孕期并降低风险。请务必与您的神经科、产科及眼科医师保持沟通，制定个性化管理计划。> 注：以上信息基于当前医学共识，具体决策需结合患者个体情况由专业医师团队指导。

9. 如果得了 NMOSD，我还能像以前一样正常工作和生活吗？

Gemini

面对确诊NMOSD（视神经脊髓炎谱系疾病），产生“还能否正常生活”的焦虑是非常正常的。作为您的眼科医生，我想给您一个明确且充满希望的答案：**在坚持规范治疗和科学管理的前提下，绝大多数NMOSD患者都可以回归正常的工作和生活。**NMOSD虽然是一种罕见的自身免疫性疾病，但随着医学的进步，现在已经有多种有效的药物可以控制它。为了实现“像以前一样正常生活”的目标，您需要在以下几个方面做好准备和调整：### 1. 核心任务：坚持规范治疗，预防复发。NMOSD最大的特点是容易复发，每一次复发都可能对视神经或脊髓造成不可逆的损伤。因此，“正常生活”的基础是**绝对不能自行停药或减药**。*	**急性期治疗**：在视力突然下降或出现神经症状时，我们会使用大剂量激素冲击或血浆置换等方法，尽量挽救视力和神经功能。*	**缓解期（维持）治疗**：这是您未来生活的常态。通过长期使用免疫抑制剂或新型的生物制剂（如单克隆抗体），可以极大地降低甚至完全阻止复发。只要病情不复发，您的生活轨迹就不会被打断。### 2. 接受“新常态”与积极康复。如果您之前的发作已经留下了一些视力受损或肢体无力的后遗症，您可能需要适应一个“新常态”。*	**视力保护**：如果单眼视力受损，另一只健康眼睛完全可以承担日常工作和生活的大部分需求。我们会指导您如何保护好这只“好眼睛”，并在必要时使用助视器。*	**康复训练**：对于遗留的神经功能障碍，尽早且持续的物理康复训练可以帮助您最大限度地恢复自理和工作能力。### 3. 工作与生活节奏的调整。您依然可以工作、旅行、社交，但需要学会“聪明地”使用自己的精力：*	**避免过度劳累**：极度的身体疲劳和精神压力是诱发自身免疫疾病复发的常见因素。您可能需要调整工作强度，避免频繁熬夜和长期高压工作。*	**预防感染**：普通的感冒、发烧或肠胃炎有时会激活免疫系统，诱发NMOSD复发。在流感季节戴好口罩、勤洗手、注意饮食卫生，并在医生指导下接种相关疫苗。*	**健康的生活方式**：保持规律的作息、均衡的饮食（多吃新鲜蔬菜水果，减少高糖高脂摄入）以及适度的锻炼（如散步、瑜伽、游泳等，以不感到疲劳为宜）。### 4. 建立多学科的医疗支持网。NMOSD不仅影响眼睛，还影响神经系统。您需要定期复查，不要等到不舒服了才来医院。*	**眼科随访**：定期进行视力、视野、OCT（光学相干断层扫描）检查，监测视神经的健康状况。*	**神经内科随访**：复查脊髓和大脑核磁共振（MRI），监测血液指标，调整免疫药物。**总结给您的一句话是：**NMOSD就像是一个需要长期共存的“慢性病伴侣”，比如高血压或糖尿病。只要您按时吃药、定期看医生、照顾好自己的身体情绪，它就会安静地休眠。您依然可以去追求您的职业发展，享受您的家庭生活。如果您在工作或生活中遇到任何视力上的困难，随时来找我，我们一起解决。

Qwen

您好！作为眼科医师的助手，我非常理解您此刻的担忧。视神经脊髓炎谱系疾病（NMOSD）确实是一种需要长期管理的慢性自身免疫性疾病，尤其是它常累及视神经，导致视力问题。关于"**得了 NMOSD，我还能像以前一样正常工作和生活吗？**"这个问题，答案是：**大多数患者在经过规范治疗和科学管理后，完全可以回归正常的工作和生活，但可能需要根据身体状况做一些适当的调整**。以下是一份为您准备的患者教育指南，旨在帮助您建立信心并掌握管理疾病的关键点：### 1. 核心观点：疾病可控，生活可期。NMOSD 虽然具有复发性，但随着医学的进步，特别是特异性生物制剂（如利妥昔单抗、伊奈利珠单抗、萨特利珠单抗等）的应用，疾病的复发率已显著降低。*	**急性期治疗**：及时的大剂量激素冲击或血浆置换可以最大程度挽救视力。*	**缓解期预防**：长期规范的免疫抑制治疗是防止复发、保护剩余视力的关键。只要病情稳定（无复发），您的认知功能、肢体力量和大部分日常生活能力通常不会受到持续影响。### 2. 工作与生活的调整策略。虽然目标是“正常”，但“正常”的定义可能需要根据您的视力状况进行微调：#### 工作方面*	**视力辅助**：如果遗留有视野缺损或视力下降，可以使用放大镜、高对比度键盘、屏幕阅读软件或调整显示器字体大小。*	**环境优化**：确保工作区域光线充足且均匀，避免眩光（NMOSD 患者常对强光敏感）。*	**节奏把控**：疲劳是常见的症状。建议采用“番茄工作法”，每工作 45-60 分钟休息 10 分钟，避免过度劳累诱发假性复发（Uhthoff 现象）。*	**职业沟通**：在病情稳定期，通常无需向雇主透露详细病情。若需特殊便利（如弹性工时），可与 HR 或直属领导进行适度沟通。#### 生活方面*	**驾驶安全**：这是眼科医师最关注的问题。如果您的视野缺损严重或视力低于法定标准，为了您和他人的安全，可能需要暂停驾驶或仅限短途日间驾驶。请务必遵医嘱进行视野检查。*	**运动锻炼**：适度的有氧运动（如散步、游泳、瑜伽）有助于改善疲劳和情绪，但应避免在高温环境下剧烈运动。*	**心理调适**：焦虑和抑郁在慢性病患者中较常见。加入病友互助小组或寻求心理咨询是非常有效的支持手段。### 4. 给患者的特别叮嘱（眼科视角）。作为眼科医生，我要特别强调以下几点，因为它们直接关系到您能否“看得清”从而正常工作：1. **警惕复发信号**：一旦发现单眼或双眼视力突然模糊、眼球转动痛、色觉改变，**不要等待**，立即就医。时间就是视力。2. **定期监测**：即使感觉良好，也请每 3-6 个月进行一次详细的眼科检查（包括最佳矫正视力、视野、OCT 视网膜神经纤维层厚度分析）。3. **疫苗接种**：在使用免疫抑制剂期间，接种流感疫苗和肺炎疫苗非常重要，因为感染是诱发 NMOSD 复发的常见因素。（*注：接种前请咨询主治医生，避免接种减毒活疫苗*）。### 总结：得了 NMOSD 并不意味着职业生涯的终结或生活质量的崩塌。> **结论**：通过**规范用药 + 定期复查 + 合理的生活方式调整**，绝大多数患者能够维持稳定的病情，继续胜任原本的工作，享受丰富多彩的生活。请您与您的神经内科医生和眼科医生保持紧密合作，我们是您坚强的后盾。如果您有任何具体的视力困扰或工作场景疑问，欢迎随时向我们咨询。

Deepseek

\# NMOSD患者教育与生活指导## 理解NMOSD：首先，我想让您了解NMOSD（视神经脊髓炎谱系疾病）是一种自身免疫性疾病，主要影响视神经和脊髓。虽然这是一种慢性疾病，但**现代医学已经取得了显著进展**，大多数患者通过规范治疗可以控制病情，维持良好的生活质量。## 关于工作和生活### 1. **工作方面**- **可以继续工作**：许多NMOSD患者在病情稳定后能够重返工作岗位- **可能需要调整**：根据症状和体力情况，可能需要调整工作强度、工作时间或工作方式- **沟通很重要**：与雇主坦诚沟通您的健康状况和可能需要的合理便利- **职业转型**：如果原工作对身体要求过高，可考虑转向更适合的岗位### 2. **日常生活**- **保持积极心态**：情绪管理对疾病控制至关重要- **规律作息**：建立稳定的作息时间，保证充足休息- **适度活动**：在医生指导下进行适合的体育锻炼- **社交参与**：保持与家人朋友的正常社交活动## 疾病管理关键点### 治疗依从性### 复发识别- 学习识别复发早期症状（视力下降、肢体无力等）- 一旦出现可疑症状，立即联系医疗团队### 生活方式调整- 均衡饮食，保持健康体重- 避免感染（勤洗手、接种推荐疫苗）- 管理压力，学习放松技巧- 戒烟限酒## 支持系统1. **医疗团队**：定期随访神经科/眼科医生2. **家人朋友**：让他们了解您的状况，获得情感支持3. **患者组织**：加入NMOSD患者支持团体，分享经验4. **心理咨询**：必要时寻求专业心理支持## 现实期望：虽然NMOSD可能需要您做出一些调整，但**大多数患者能够找到新的平衡点**，继续从事有意义的工作和享受生活。疾病管理是一个持续的过程，随着治疗方案的优化和自我管理的提升，生活质量可以逐步改善。**请记住**：每个人的病程都是独特的。与您的医疗团队密切合作，制定个性化的管理计划，是维持最佳生活质量的关键。> 本信息仅供参考，具体治疗方案请遵循主治医师的专业建议。

10. NMOSD 是否会遗传给下一代？

Gemini

为NMOSD（视神经脊髓炎谱系疾病）患者进行关于遗传问题的健康教育时，建议向患者传达以下核心信息，以消除他们的顾虑并引导正确的孕产管理：**1. 明确结论：NMOSD通常不会遗传给下一代**可以直接且肯定地告诉患者，NMOSD不是传统意义上的遗传病（如血友病或白化病）。绝大多数NMOSD是散发性的，这意味着患者的家族中通常只有他们自己患病，把疾病直接遗传给孩子的概率极低。**2. 解释疾病本质：自身免疫问题而非基因缺陷*可以用通俗的语言向患者解释：NMOSD是因为体内的免疫系统出现了紊乱，“误伤”了自身的视神经和脊髓（通常与产生AQP4抗体有关），而不是因为父母的基因存在缺陷并传递给了下一代。**3. 客观说明“遗传易感性”*为了保证医学严谨性，可以补充说明：像红斑狼疮、类风湿等其他自身免疫性疾病一样，NMOSD可能存在一定的“遗传易感性”。这意味着患者的孩子可能在免疫体质上比普通人稍微敏感一些，但这绝对不等于他们会发病。医学统计显示，家族性NMOSD的病例极其罕见（比例通常不足3%）。**4. 转移重点：强调备孕与孕产期的病情管理**在解除患者对“遗传”的担忧后，应将教育重点转移到真正需要关注的生育安全问题上：*	**药物影响：** 患者目前使用的许多预防复发的药物（如某些免疫抑制剂或生物制剂）可能对胎儿有致畸风险。必须强调：**绝对不能意外怀孕，计划怀孕前必须提前半年以上与医生沟通调整用药。**	**复发风险：** 孕期尤其是产后（产后3-6个月内），是NMOSD病情复发的高危期。需要神经内科、眼科和产科医生共同制定孕产期及哺乳期的治疗与随访方案。**医生与患者沟通的话术示例：> “请您放心，NMOSD并不是遗传病，它通常不会遗传给您的孩子，您完全有机会拥有一个健康的宝宝。这是一种免疫系统‘认错人’导致的疾病，而不是基因遗传。> 虽然疾病不遗传，但如果您有生育计划，我们真正需要严格把关的是您的**用药安全**和**产后防复发**。您现在用的某些药可能对宝宝有影响，而且产后是疾病容易复发的时候。所以，如果您打算要孩子，请务必提前几个月告诉我，我们来为您调整最安全的治疗方案，为您和宝宝保驾护航。”

Qwen

您好！作为眼科医师的助手，我为您整理了一份针对**视神经脊髓炎谱系疾病（NMOSD）**患者的遗传咨询教育材料。这份内容旨在帮助医师向患者清晰、科学地解释 NMOSD 的遗传风险，缓解患者的焦虑，同时提供准确的医学信息。眼科医师患者教育指南：NMOSD 与遗传风险#### 1. 核心结论：NMOSD 不是典型的“遗传病”。首先，我们需要明确告诉患者：**NMOSD 不属于传统的单基因遗传病**（如红绿色盲或血友病），它不会像某些疾病那样直接由父母必然地传递给子女。*	**非孟德尔遗传**：NMOSD 不符合显性或隐性遗传的规律。*	**多因素致病**：目前医学界公认，NMOSD 是由**遗传易感性**（基因背景）与**环境因素**共同作用引发的自身免疫性疾病。#### 2. 遗传机制解析：易感性 vs. 确定性。*	**遗传易感性**：患者体内可能存在某些特定的基因变异（主要位于人类白细胞抗原 **HLA** 区域，如 $HLA-DRB1$ 等位基因），这些基因增加了患病的“倾向”，但**不等于**一定会发病。*	**环境触发**：仅有基因通常不足以发病，往往需要病毒感染、疫苗接种、精神压力或激素水平变化等环境因素作为“扳机”。#### 3. 数据支持：家族聚集性极低。根据目前的流行病学研究数据，NMOSD 的家族聚集现象非常罕见：| 指标 | 数据描述 | 临床意义 || **家族发病率** | < 5% | 绝大多数患者没有家族史。 || **一级亲属风险** | 略高于普通人群，但绝对风险仍很低 | 即使父母患病，子女患病的概率依然很小（远低于 50% 或 25%）。 || **双胞胎研究** | 同卵双胞胎共患率不高 | 证明基因不是唯一决定因素，环境因素至关重要。 |> **注**：相比之下，多发性硬化（MS）的家族聚集性略高于 NMOSD，但两者均非直接遗传。#### 4. 给患者的具体建议。作为眼科医师，在诊室中您可以这样对患者沟通：1. **关于生育**：*	“您完全可以正常生育。NMOSD 不会直接‘传’给孩子。”*	“孩子可能会继承一些让您容易生病的基因体质，但这就像继承了‘容易过敏’的体质一样，不代表他们一定会得 NMOSD。”2. **关于监测**：*	虽然风险低，但如果未来子女出现不明原因的视力下降、眼球转动痛或肢体麻木，应及时就医并告知医生家族史，以便早期排查 $AQP4-IgG$ 抗体。3. **孕期管理（重要提示）**：*	虽然遗传风险低，但**妊娠期和产后**是 NMOSD 复发的高风险期（尤其是产后 3-6 个月）。*	建议患者在计划怀孕前与神经内科及眼科医生共同评估病情，调整免疫抑制剂方案（部分药物需提前停用），确保在病情稳定期受孕。#### 5. 常见问答模拟 (Q&A)*	**患者问**：“医生，我妈妈也有类似的眼病，这是不是说明肯定会传给我儿子？”*	**医师答**：“家族中有病例确实提示可能存在一定的遗传背景，但这并不意味着您的儿子一定会发病。这就好比家里有人高血压，孩子风险稍高，但只要注意生活方式，完全可能终身不发病。NMOSD 更需要特定的环境触发才会显现。”*	**患者问**：“我需要给孩子做基因检测吗？” *	**医师答**：“目前**不推荐**对无症状的儿童进行常规的 NMOSD 相关基因筛查。因为即使查出易感基因，也无法预测是否发病，反而可能造成不必要的心理负担。我们主张‘有症状再检查’。”总结要点卡片：您可以将以下要点打印或展示给患者：**结论**：NMOSD **不是**直接遗传病。**机制**：是“基因易感性” + “环境触发”的结果。**概率**：子女患病概率**极低**，不必过度恐慌。**行动**：关注自身孕期管理，子女若有症状及时就医，无需常规基因筛查。希望这份材料能协助您更好地为患者答疑解惑，减轻他们的心理负担！如果有其他具体的临床场景需要模拟，请随时告诉我。

Deepseek

\### NMOSD的遗传风险：给患者的科学解答#### 核心结论**NMOSD（视神经脊髓炎谱系疾病）通常不被认为是直接遗传的疾病。目前研究认为，它主要是由免疫系统异常攻击自身神经系统引起的，而非通过基因直接传递给下一代。#### 详细解释1. **遗传模式**- **非孟德尔遗传**：NMOSD不符合典型的遗传病模式（如单基因遗传）。- **多因素疾病**：发病是**遗传易感性**、**环境因素**和**免疫状态**共同作用的结果。- **关键基因**：部分患者携带`HLA-DRB1*03:01`等基因变异，可能增加患病风险，但**单独基因不足以致病**。2. **家族风险数据**- **极低家族聚集性**：超过95%的NMOSD病例为散发性，家族中多人患病的情况罕见。- **风险估算**：若一级亲属（父母、子女、兄弟姐妹）患病，其他成员的终身患病风险**略高于普通人群，但绝对风险仍很低**（通常＜5%）。- **双胞胎研究**：同卵双胞胎共患率显著低于典型遗传病，进一步支持环境因素的关键作用。3. **关键影响因素**- **环境触发**：感染（如EB病毒）、维生素D缺乏、地理因素等可能诱发免疫异常。- **抗体核心作用**：多数患者体内存在**AQP4-IgG抗体**，这是获得性免疫异常的结果，而非先天遗传缺陷。- **性别差异**：女性患病率显著高于男性（约9:1），提示激素或性染色体相关机制可能参与。#### 给患者的实用建议1. **遗传咨询**- 若计划生育，可咨询**神经免疫专科医师**或**遗传咨询师**进行个性化风险评估。- 目前**不推荐常规基因筛查**用于预测子女患病风险。2. **孕期管理**- 多数NMOSD治疗药物在孕期需调整或暂停，**孕前务必与专科医师制定治疗计划**。- 疾病活动期可能增加妊娠并发症风险，建议在疾病稳定期（通常≥6个月无复发）计划怀孕。3. **子女健康监测**- 无需过度焦虑，但可注意子女是否出现**不明原因的视力下降、肢体麻木或排尿障碍**等罕见症状，及时就医排查。- 保持健康生活方式（均衡营养、充足日照、适度锻炼）有助于降低自身免疫病风险。#### 最新研究进展- **全基因组关联研究（GWAS）**：已识别数个NMOSD相关基因位点，但每个位点的贡献度很小。- **表观遗传学**：DNA甲基化等机制可能解释环境因素如何影响疾病发生。- **未来方向**：精准医学可能通过多基因风险评分结合环境因素进行风险评估，但目前尚未临床应用。#### 重要提醒- NMOSD的**治疗和管理远比遗传风险更重要**。坚持规范治疗、预防复发是改善长期预后的关键。- 避免过度关注遗传问题而增加心理负担，必要时可寻求心理支持。**总结**：NMOSD的遗传风险很低，不属于传统意义上的遗传病。患者可以相对安心地规划家庭，重点应放在疾病本身的长期管理上。*注：以上信息基于当前医学证据，具体个案请咨询主治医师。医学认识不断更新，建议定期随访获取最新资讯。*

11. 目前有没有针对 NMOSD 病因的根治性疗法？

Gemini

目前还没有针对NMOSD（视神经脊髓炎谱系疾病）的根治性疗法，但该疾病是完全可以通过药物有效控制的。为了协助您对患者进行教育，建议您可以从以下几个方面向患者解释：**1. 明确疾病本质，打破“去根”迷思**您可以向患者说明，NMOSD是一种自身免疫性疾病，也就是体内的免疫系统“认错了人”，错误地攻击了自身的视神经和脊髓（通常与AQP4抗体有关）。目前的医学技术还无法彻底抹除免疫系统的这种错误记忆，因此无法做到传统意义上的“断根”。**2. 强调治疗的核心目标：预防复发**需要明确告知患者，虽然不能根治，但治疗的意义极其重大。NMOSD的特点是高复发率和高致残率，每一次复发都可能对视力或神经功能造成不可逆的损伤。因此，目前的治疗目标是**最大限度地减少甚至阻止复发**，从而保护视力、防止残疾。**3. 介绍目前成熟的治疗手段**可以向患者简单介绍目前的治疗分为两步，增加他们对现代医学的信心：*	**急性期治疗：** 在视力突然下降或发病时，通过大剂量糖皮质激素冲击或血浆置换，快速把炎症压制下去，抢救视力和神经功能。*	**缓解期治疗（长期维持）：** 这是重中之重。目前有非常多且有效的武器来预防复发，包括传统的免疫抑制剂（如硫唑嘌呤、吗替麦考酚酯等），以及近年来获批的多种精准靶向生物制剂（如依库珠单抗、伊奈利珠单抗、萨特利珠单抗等）。这些新药能极大幅度地降低复发风险。**4. 给出日常行动指南**总结给患者的最终建议：虽然疾病伴随终身，但只要做到**严格遵医嘱长期规律用药、绝不擅自减药或停药、定期门诊随访复查**，绝大多数患者都可以像慢性病（如高血压、糖尿病）一样将病情控制在平稳状态，保持良好的视力和生活质量。

Qwen

您好！作为 AI 聊天机器人，我很乐意协助您为眼科医师提供关于视神经脊髓炎谱系疾病（NMOSD）的患者教育素材。针对患者最关心的核心问题："**目前有没有针对 NMOSD 病因的根治性疗法？**"，以下是建议的回答要点和教育策略：### 核心结论**目前尚无针对 NMOSD 病因的“根治性”疗法**。NMOSD 是一种慢性、复发性、自身免疫性疾病。目前的医疗水平无法彻底清除体内的致病机制（如水通道蛋白 -4 抗体，AQP4-IgG），使其永不复发。但是，**通过规范的长期免疫治疗，绝大多数患者可以实现“无发作”状态，从而避免视力丧失和瘫痪等严重残疾**。### 给眼科医师的患者教育要点。在向患者解释时，建议采用以下逻辑结构，既诚实面对现状，又给予希望：#### 1. 明确疾病性质：是“控制”而非“治愈”*	**比喻解释**：可以将 NMOSD 比作高血压或糖尿病。虽然我们不能说“治愈”了高血压（即停药后血压永远正常），但通过每天服药，患者可以像正常人一样生活，避免中风或肾衰竭。*	**关键信息**：NMOSD 的治疗目标是**预防复发**。每一次复发都可能导致不可逆的神经损伤（如永久性失明），因此“不复发”就是最大的胜利。#### 2. 解释当前的治疗策略。目前的治疗主要分为两个阶段，需向患者强调**急性期**和**缓解期**治疗的区别：*	**急性期治疗**（救火）：当视力突然下降或出现肢体无力时，需要大剂量激素冲击、血浆置换等，目的是尽快抑制炎症，挽救残存功能。*	**缓解期预防治疗**（防火）：这是长期管理的核心。即使感觉良好，也必须坚持服用免疫抑制剂或使用生物制剂。*	**传统药物**：如硫唑嘌呤、吗替麦考酚酯、利妥昔单抗等。*	**新型靶向生物制剂**：近年来获批的药物（如依库珠单抗、伊奈利珠单抗、萨特利珠单抗等）能更精准地阻断致病通路，显著降低复发率。#### 3. 为什么要长期坚持？很多患者在病情稳定后会自行停药，这是极其危险的。*	**数据支持**：未经预防治疗的 NMOSD 患者，5 年内的复发率极高（>90%）。*	**后果警示**：NMOSD 的致残具有累积性。第一次复发可能恢复较好，但第二次、第三次复发往往导致严重的、不可逆的视力丧失或截瘫。#### 4. 未来展望。虽然目前没有根治方法，但医学进步迅速。*	随着对 AQP4 抗体机制研究的深入，越来越多的靶向药物问世。*	干细胞移植等探索性疗法也在研究中，未来有望实现更深层次的缓解甚至功能性治愈。### 患者沟通辅助图表。### 总结话术示例（供医师参考）> “目前医学上还没有一种药能把 NMOSD 这个病‘断根’，让它永远不再回来。这就像高血压一样，需要长期管理。但是，请不要灰心，我们现在有很多有效的武器（药物）。只要您遵医嘱坚持预防治疗，完全有可能几十年都不发作一次，保住您的视力和行走能力。**我们的目标不是‘治愈’这个词，而是让您‘不复发’，过上和正常人一样的生活**。"希望这些信息能协助您更好地开展患者教育工作！如果有具体的药物机制或最新临床试验数据需要查询，请随时告诉我。

Deepseek

\## 关于NMOSD病因的根治性疗法。目前，**尚无针对NMOSD（视神经脊髓炎谱系疾病）病因的根治性疗法**。NMOSD是一种自身免疫性疾病，其发病机制主要与水通道蛋白4（AQP4）抗体介导的免疫攻击有关，导致中枢神经系统（尤其是视神经和脊髓）的炎症和损伤。### 当前治疗策略。虽然无法根治，但通过以下治疗手段可以有效控制疾病、减少复发、延缓残疾进展：1. **急性期治疗**：- **大剂量糖皮质激素**：如甲泼尼龙冲击疗法，用于抑制急性炎症。- **血浆置换（PLEX）**：用于重症或对激素反应不佳的患者，快速清除抗体。- **静脉注射免疫球蛋白（IVIG）**：可作为替代治疗。2. **预防复发（长期免疫治疗）**：- **靶向生物制剂**：- **抗CD20单抗**（如利妥昔单抗、奥法妥木单抗）：清除B细胞，减少抗体产生。- **抗IL-6受体单抗**（如萨特利珠单抗）：阻断炎症信号通路。- **补体抑制剂**（如依库珠单抗）：抑制补体介导的损伤。- **传统免疫抑制剂**：- **霉酚酸酯**、**硫唑嘌呤**、**甲氨蝶呤**等，用于抑制免疫系统活性。3. **对症支持治疗**：- 疼痛管理、疲劳改善、膀胱功能障碍处理等。- 康复训练（如物理治疗、职业治疗）以维持功能。### 重要注意事项- **早期诊断和治疗**是改善预后的关键，可显著降低复发风险和残疾累积。- **患者需终身管理**，定期随访监测抗体滴度、影像学变化及药物副作用。- **避免诱因**：感染、应激、疫苗接种（需谨慎评估）可能诱发复发。### 未来展望。研究正在探索更精准的免疫调节疗法、干细胞治疗等，但均处于实验阶段，尚未应用于临床根治。### 给患者的建议。1. **严格遵医嘱**用药，不可自行停药或调整剂量。2. **定期复查**：包括神经系统评估、MRI及血液检查。3. **生活方式调整**：均衡饮食、适度锻炼、避免过度劳累。4. **警惕复发迹象**：如视力下降、肢体麻木无力、排尿障碍等，需立即就医。作为眼科医师，您可以强调**早期干预和规范治疗对保护视功能至关重要**，同时提供心理支持，帮助患者建立积极的管理态度。如果您需要更详细的患者教育材料或图表，我可以进一步协助制作。

12. NMOSD 患者出现认知功能下降是否常见？

Gemini

NMOSD（视神经脊髓炎谱系疾病）患者出现认知功能下降是**相对常见的**，但在临床和患者自我认知中往往容易被其严重的躯体症状（如视力丧失、瘫痪）所掩盖而遭到忽视。以下是供眼科医师在进行患者教育时参考的核心信息：**1. 发生率**研究表明，约有 **40% 至 70%** 的NMOSD患者存在不同程度的认知功能障碍。虽然NMOSD主要以视神经炎和横贯性脊髓炎为特征，但它同样会累及大脑。**2. 常见的认知受损领域*患者最常报告和被检测出的认知下降主要集中在：*	**信息处理速度减慢**（感觉“脑子转得慢”）*	**注意力下降**（难以集中精力）*	**记忆力减退**（尤其是近期记忆和视觉记忆）*	**执行功能障碍**（如计划、组织和多任务处理能力下降）**3. 导致认知下降的原因***	**直接的脑部损伤：** 大脑中富含水通道蛋白4（AQP4）的区域（如室管膜周围、间脑）可能出现病灶。此外，即使常规MRI未发现明显脑部病灶，微观的脑白质/灰质损伤及脑萎缩也可能已经发生。*	**继发性因素（非常关键）：** 严重的视力下降本身就会减少大脑的视觉刺激输入。同时，NMOSD常伴随的慢性疼痛、严重疲劳、睡眠障碍以及抑郁/焦虑情绪，都会显著拖累患者的认知表现。*	**药物副作用：** 长期使用大剂量糖皮质激素或其他免疫调节药物也可能对情绪和记忆产生一定影响。**4. 给患者的教育与建议***	**正常化这一症状：** 明确告知患者，记忆力减退或思维迟缓是NMOSD的已知症状，验证他们的感受，减轻其心理负担。*	**管理继发因素：** 鼓励患者积极向医生反馈情绪低落、失眠或疼痛问题。改善抑郁和睡眠通常能立竿见影地提升认知状态。*	**建立代偿机制：** 建议患者在日常生活中多使用备忘录、日历、手机提醒等工具；在需要专注时，尽量减少环境干扰（如关掉背景电视）。*	**多学科评估：** 如果认知下降明显影响了日常生活、学习或工作，建议患者前往神经内科或临床心理科进行专业的神经心理学评估。如果您需要关于NMOSD认知障碍的特定文献数据或更详细的干预方案，我可以进一步补充。

Qwen

您好！作为眼科医师的助手，针对**视神经脊髓炎谱系疾病（NMOSD）**患者关于“认知功能下降”的疑问，以下是一份专业的患者教育资料。这份资料旨在澄清误区、提供科学依据，并指导患者如何应对。###患者教育核心摘要：NMOSD 与认知功能**直接回答：**与传统观念不同，**认知功能下降在 NMOSD 患者中并不像在多发性硬化（MS）中那样普遍或严重，但也绝非罕见。** 近年来的研究表明，约有 **30% - 50%** 的 NMOSD 患者可能存在不同程度的认知障碍，但其表现形式和严重程度通常轻于多发性硬化患者。### 1. 科学数据与现状分析。过去认为 NMOSD 主要影响视神经和脊髓，大脑相对“幸免”。然而，随着高分辨率 MRI 技术和神经心理学评估的普及，我们发现：*	**发生率**：研究显示，约三分之一到一半的患者在疾病过程中会出现可检测到的认知改变。*	**主要受影响领域**：*	**处理速度**（反应变慢）*	**注意力**（难以集中精神）*	**记忆力**（尤其是短期记忆和工作记忆）*	**执行功能**（计划、组织能力稍弱）*	**对比多发性硬化（MS）**：NMOSD 患者的认知障碍通常较轻微，且较少出现严重的痴呆样表现。MS 患者的认知受损率高达 40%-70%，且往往更早出现。### 2. 为什么会出现这种情况？除了疾病本身对脑组织（特别是富含水通道蛋白 -4, AQP4 的区域）的直接损伤外，以下因素也会加剧“感觉脑子不好使”的情况：1. **继发性因素**：*	**极度疲劳**：NMOSD 患者常伴有严重的中枢性疲劳，这会直接导致注意力无法集中。*	**情绪问题**：因病致残（如视力下降、肢体无力）引发的抑郁和焦虑，会显著影响认知测试表现。*	**睡眠障碍**：疼痛或不适导致的睡眠质量差。2. **治疗相关因素**：*	长期或大剂量使用**糖皮质激素**可能导致情绪波动、失眠和暂时的记忆减退（俗称“激素脑”）。### 3. 给患者的建议与应对策略。如果您或您的家属感觉记忆力或反应力有所下降，请采取以下措施：####行动清单| 步骤 | 具体行动 | 目的 || **1. 坦诚沟通** | 在复诊时主动告知眼科医师或神经科医师您的困扰。 | 排除药物副作用或其他可逆原因。 || **2. 专业评估** | 请求进行简单的神经心理筛查（如 MoCA 量表或 SDMT 测试）。 | 量化认知水平，建立基线数据。 || **3. 控制炎症** | 严格遵医嘱使用免疫抑制剂，预防复发。 | **预防复发是保护认知功能最关键的手段。** || **4. 生活方式干预** | 保证充足睡眠，适度有氧运动，管理压力。 | 改善脑血流，缓解疲劳和抑郁。 || **5. 认知训练** | 阅读、拼图、学习新技能或使用专门的认知训练 APP。 | 利用神经可塑性代偿受损功能。 |### 4. 常见误区澄清 (Q&A)> **Q: 我最近记性变差，是不是病情恶化了？**> **A:** 不一定。这可能源于疲劳、压力或激素药物。当然，也需要排除新的无症状脑部病灶。请务必就医检查，不要自行恐慌。> **Q: NMOSD 最终会导致痴呆吗？**> **A:** **极罕见。** 绝大多数 NMOSD 患者的认知障碍是轻度至中度的，通过管理和康复可以维持良好的生活质量，不会发展成阿尔茨海默病那样的严重痴呆。> **Q: 既然主要是眼科问题，我需要看神经科吗？**> **A:** **非常需要。** NMOSD 是中枢神经系统疾病，眼科医师通常与神经科医师协作诊疗。认知问题属于神经科范畴，联合诊疗能获得最佳方案。##结语。虽然 NMOSD 患者出现认知功能下降的风险确实存在，但它通常是**可控、可管理且程度较轻**的。**关键信息总结：**1. 不要忽视细微的认知变化，及时告知医生。2. 预防疾病复发是保护大脑的根本。3. 保持良好的心态和生活习惯能显著改善认知状态。作为您的医疗团队的一员，我们建议您定期进行随访，不仅关注视力，也要关注整体的神经系统健康。*(注：本内容仅供患者教育参考，不能替代专业医生的诊断和治疗建议。如有具体症状，请咨询您的主治医生。)*

Deepseek

\# NMOSD患者认知功能下降情况说明## 认知功能下降在NMOSD患者中的发生率。根据目前临床研究数据，**NMOSD患者出现认知功能下降并不罕见**，但发生率低于多发性硬化(MS)患者。具体数据如下：| 认知领域 | NMOSD患者受影响比例 | 备注 || 信息处理速度 | 约30-40% | 最常见的认知障碍 || 执行功能 | 约25-35% | 包括计划、决策等能力 || 记忆力 | 约20-30% | 尤其是工作记忆 || 注意力 | 约15-25% | 持续注意和分配注意 |## 认知障碍的可能机制1. **直接病灶影响**- 大脑半球白质病灶- 胼胝体受累- 皮层下结构损伤2. **间接因素**- 疲劳和睡眠障碍- 抑郁和焦虑情绪- 药物副作用- 疼痛干扰注意力## 临床管理建议### 1. 定期评估### 2. 干预策略- **药物治疗调整**：评估现有药物对认知的潜在影响- **认知康复训练**：针对性训练受影响领域- **生活方式干预**：- 规律作息，保证充足睡眠- 适度有氧运动（每周150分钟）- 地中海饮食模式- 认知刺激活动（阅读、 puzzles等）### 3. 患者教育要点## 重要提醒1. **不是所有NMOSD患者都会出现认知下降**，个体差异很大2. **轻度认知变化不一定影响日常生活能力**3. **认知评估应作为NMOSD综合管理的一部分**4. **如有明显变化，应及时与医疗团队沟通**## 给患者的实用建议- 使用日历、提醒工具辅助记忆- 将复杂任务分解为小步骤- 在注意力最佳时段处理重要事务- 定期进行轻度到中度体育锻炼- 保持社交活动，避免孤立**关键信息**：虽然NMOSD患者可能出现认知功能变化，但通过早期识别、适当干预和综合管理，大多数患者可以维持良好的生活质量。请定期随访，与医疗团队保持沟通，共同制定个性化的管理方案。> 注：以上信息基于当前临床证据，具体管理方案需根据个体情况由专业医疗团队制定。

13. NMOSD 会不会让我以后坐轮椅或者失明？

Gemini

面对患者的这种担忧，建议您以客观、诚恳且充满希望的语气进行沟通。NMOSD（视神经脊髓炎谱系疾病）确实具有致残风险，但现代医学的进步已经极大地改变了这种疾病的预后。您可以参考以下的话术框架和要点来对患者进行教育和心理疏导：**1. 直接回应担忧，给予希望**“我非常理解您的害怕和担忧。视力受损和行动不便确实是NMOSD可能带来的严重后果，因为这个病主要攻击的就是视神经和脊髓。但是，我想非常明确地告诉您：**这绝对不是不可避免的结局。** 只要治疗得当，很多患者都能长期保持正常的生活和工作。”**2. 解释疾病特点，强调“防复发”的核心**“NMOSD的特点是‘发作性’的。它不像某些疾病会每天持续不断地恶化，它的伤害主要来自于每一次的‘急性复发’。每一次复发，都可能对神经造成一次损伤，长期积累下来才会导致失明或坐轮椅。因此，**我们治疗的核心目标非常明确，就是：零复发。** 只要不复发，您的视力和躯体功能就能得到保护。”**3. 介绍现代医学的进步，建立信心**“现在的医学水平和十几年前已经完全不同了。过去我们手里的武器不多，但现在，除了传统的免疫抑制剂，我们还有了针对NMOSD的多种新型生物制剂（靶向药物）。这些新药在预防复发方面的效果非常显著。只要找到适合您的药物，我们有很大的把握将复发风险降到最低。”**4. 明确患者需要配合的行动指南**为了达到“不失明、不坐轮椅”的目标，向患者强调他们必须做到的几件事：*	**绝对遵医嘱用药：** 这是最重要的一点。在任何情况下，都不要因为“感觉自己好了”或者“担心药物副作用”就擅自停药或减量。停药是导致复发的最大诱因。*	**定期复查：** 我们和神经内科的医生需要定期监测您的指标，评估药物效果和副作用，及时调整方案。*	**警惕先兆，迅速就医：** 如果您突然出现视力下降、眼睛疼、肢体麻木无力、甚至是不明原因的剧烈恶心呕吐和打嗝，不要等待，必须立刻来医院。急性发作期越早用药（如大剂量激素冲击或血浆置换），神经恢复的可能性就越大。**5. 总结与安抚**“您不是一个人在面对这个疾病。眼科和神经内科的医生会组成团队为您保驾护航。把它当成一种像高血压、糖尿病一样的慢性病来管理，与医生做好配合，您完全有机会拥抱高质量的生活。”

Qwen

您好！作为眼科医师的助手，我非常理解您提出这个问题时的焦虑和恐惧。**视神经脊髓炎谱系疾病（NMOSD）** 确实是一种严重的自身免疫性疾病，主要攻击视神经和脊髓，但"**会不会坐轮椅或失明**"并不是一个非黑即白的定局。现代医学的进步，特别是新型生物制剂的出现，已经极大地改变了 NMOSD 的预后。以下我将为您详细拆解这两个核心担忧，并提供科学的应对策略。### 1. 核心结论：风险存在，但可控可防。首先直接回答您的问题：*	**如果不治疗或治疗不规范**：NMOSD 确实具有高复发率，每一次复发都可能导致不可逆的神经损伤，累积下来确实存在视力严重下降甚至失明，以及肢体瘫痪需要坐轮椅的风险。*	**如果早期诊断并规范治疗**：绝大多数患者可以**避免**复发，从而**保留**现有的视力和行走能力，长期维持正常的生活质量。**关键理念**：NMOSD 的管理目标已从“发作后治疗”转变为"**零复发**"。只要不复发，神经功能通常不会进一步恶化。### 2. 关于失明的风险与防护。NMOSD 引起的视神经炎往往比多发性硬化（MS）更严重，单次发作就可能导致严重的视力下降。*	**风险机制**：抗体（如 AQP4-IgG）攻击视神经，导致炎症、水肿，进而破坏神经纤维。*	**现状数据**：在过去，约 50% 的患者在发病 5 年内会出现单眼或双眼严重视力障碍。但在引入利妥昔单抗（Rituximab）、伊奈利珠单抗（Inebilizumab）、萨特利珠单抗（Satralizumab）等靶向药物后，复发率降低了 70%-90% 以上。*	**如何避免失明**：1. **急性期抢救**：一旦感觉视力模糊、眼球转动痛或视野缺损，必须**立即**就医。大剂量激素冲击或血浆置换（PLEX）在发病初期（最好 2 周内）使用，能最大程度挽救视力。2. **预防性维持治疗**：这是最关键的一步。即使现在视力正常，也必须终身服用免疫抑制剂或注射生物制剂，防止下一次发作。### 3. 关于坐轮椅（肢体瘫痪）的风险与防护。脊髓炎是 NMOSD 的另一大特征，可能导致肢体无力、麻木、大小便功能障碍。*	**风险机制**：脊髓长节段的炎症损伤会导致运动传导通路中断。*	**如何避免瘫痪**：1. **识别预警信号**：出现背部束带感、双腿无力、排尿困难时，切勿当作腰肌劳损处理，应立即排查 NMOSD 复发。2. **康复治疗**：即使在急性期后遗留了一些无力症状，通过早期的康复训练（物理治疗），许多患者也能恢复行走能力，不一定需要依赖轮椅。3. **长期管理**：同样依赖于严格的防复发治疗。### 4. 患者行动指南：掌握主动权。#### 给您的具体建议：1. **依从性是生命线**：千万不要因为“感觉好了”就自行停药或减量。NMOSD 的复发往往悄无声息，直到造成永久损伤。2. **建立“急救意识”**：*	如果您发现视力突然下降、视野变窄。*	如果您发现双腿突然无力、踩棉花感、大小便失控。*	**行动**：不要观察等待，立即前往医院急诊或联系您的主治医生。时间就是视力，时间就是行走的能力。3. **定期复查**： *	监测抗体滴度（AQP4-IgG）。*	进行 OCT（光学相干断层扫描）检查视网膜神经纤维层厚度，这是评估视神经受损程度的敏感指标。*	定期进行脊髓和脑部 MRI 检查。### 5. 总结与心理支持**NMOSD 不等于绝症，也不等于必然残疾。**虽然它是一种慢性病，需要长期管理，但随着医疗技术的发展，越来越多的 NMOSD 患者能够正常工作、生活、甚至生育。*	**悲观的看法**：担心未来会坐轮椅或失明。*	**科学的看法**：只要我严格遵医嘱用药，敏锐捕捉复发信号，我就有极大的概率**避免**这些严重后果。请与您的眼科医师和神经内科医师保持紧密合作。您不是一个人在战斗，我们整个医疗团队都在帮助您守护光明和行动的自由。*注：以上信息仅供健康教育参考，具体诊疗方案请务必遵循您的主治医生的指导。*

Deepseek

\### 您好，我是您的AI健康助手。作为眼科医师的辅助工具，我将为您提供关于NMOSD（视神经脊髓炎谱系疾病）的专业医学信息，帮助您更好地理解和管理这一疾病。首先，请允许我强调：**以下所有信息均为医学科普和教育支持，不能替代您的主治医师的面对面诊疗建议。请务必与您的医疗团队保持密切沟通。**### **核心问题解答：NMOSD会不会导致坐轮椅或失明？**这是一个非常现实且重要的问题。答案是：**存在这种可能性，但通过现代规范化的治疗和管理，绝大多数患者可以避免这些严重后果，或将其影响降到最低。**我们可以从两个方面来理解：#### **1. 风险确实存在（为什么会有这样的担忧）**NMOSD是一种主要攻击视神经和脊髓的中枢神经系统自身免疫性疾病。*	**对脊髓的攻击**：可能导致严重的肢体无力、麻木、大小便功能障碍。如果不及时有效控制，反复发作的脊髓炎可能造成神经功能的不可逆损伤，累积导致行走困难，甚至需要轮椅辅助。*	**对视神经的攻击**：可能导致视神经炎，引起视力急剧下降、视野缺损、色觉异常。反复或严重的视神经炎发作，可能对视神经造成永久性损害，导致视力严重丧失，甚至失明。**因此，您的担忧是完全合理的，这也正是我们需要积极、规范治疗的根本原因。**#### **2. 现状与希望（如何最大程度避免）**过去，由于认识不足和治疗手段有限，NMOSD的致残率确实较高。但**近年来，诊疗理念和药物已取得革命性进步**，治疗目标已经从“控制急性发作”转变为 **“零复发”或“最小化复发”** ，从而保护神经功能，防止残疾累积。**避免严重后果的三大支柱：**| 支柱 | 具体措施 | 目标与作用 || **① 长期预防治疗** | 使用**免疫抑制剂**或**靶向生物制剂**（如利妥昔单抗、萨特利珠单抗、伊奈利珠单抗等）。这是治疗的基石。 | **核心目标：** 抑制异常免疫系统，从根源上**大幅降低复发率**（可达90%以上）。没有复发，就没有新的神经损伤。 || **② 急性期强化治疗** | 一旦出现新发症状（如视力下降、肢体麻木无力），**立即就医**。通常采用大剂量激素冲击和/或血浆置换。 | **核心目标：** 快速抑制炎症，**最大程度减轻本次发作的损伤**，挽救神经功能。时间就是神经！ || **③ 全程自我管理与监测** | **识别复发“红旗信号”**：如新出现的视力模糊、肢体麻木/无力、疼痛、持续恶心呕吐等。<br>**定期随访**：按时复查（MRI、血液抗体滴度、视力视野等）。<br>**健康生活方式**：避免感染（常见诱因）、均衡营养、在医生指导下适度活动。 | **核心目标：** 实现**早发现、早治疗**，并与医疗团队形成管理合力。 |### **给您的关键建议与教育要点**1. **建立信心，但保持警惕**：NMOSD已从“不治之症”转变为一种**可治疗、可管理的慢性病**。许多患者通过规范治疗，可以长期不复发，维持正常的工作和生活。但绝不能掉以轻心。2. **治疗依从性是生命线**：**切勿自行停药或减药**。长期预防治疗就像为神经系统筑起一道“防火墙”，随意停药等于撤掉防火墙，复发风险会急剧升高。3. **您是自身健康的第一责任人**：请与您的神经内科和眼科医生组成“战队”。您需要：*	**熟记复发征兆**。*	**记录症状日记**。*	**随身携带“医疗警示卡”**，注明疾病名称、主治医生联系方式、常用药物，以备急诊时需要。4. **关注整体健康**：NMOSD可能合并其他自身免疫病（如干燥综合征、甲状腺疾病），并可能因长期使用激素或免疫抑制剂带来一些副作用（如骨质疏松、感染风险增加）。定期进行全面的健康检查非常重要。### **总结****“会不会坐轮椅或失明？”——这个问题的主动权，很大程度上掌握在您和您的医疗团队手中。****现代医学的目标，正是通过“预防复发”和“急性期高效干预”这两大策略，让您远离这些严重的功能障碍。** 这条路需要耐心、信心和良好的医患合作。请您带着这些信息，与您的主治医生进行更深入的沟通，制定个性化的治疗和监测方案。您不是一个人在战斗。*免责声明：本内容由AI生成，旨在提供健康知识科普和信息支持，不能替代专业医疗建议、诊断或治疗。如有医疗问题，请务必咨询合格的医疗保健提供者。*

14. 我平时应该注意什么，才能减少 NMOSD 突然复发的风险？

Gemini

视神经脊髓炎谱系疾病（NMOSD）的复发可能会导致视力或神经功能出现不可逆的损伤，因此“预防复发”是日常管理的重中之重。您可以向患者强调以下几个核心注意事项：**1. 绝对遵医嘱，规范用药（最重要的一点）***	**按时按量：** NMOSD的维持期治疗通常需要长期使用免疫抑制剂或生物制剂。必须严格按照医生的处方用药。*	**切勿自行停药或减量：** 即使感觉症状已经完全消失，或者担心药物副作用，也绝对不能自行停药或改变剂量。自行停药是导致NMOSD突然复发的最常见、最危险的原因。如有不适，应及时与医生沟通调整方案。**2. 积极预防感染***	**感染是诱发：** 感冒、发烧、肠胃炎、泌尿系统感染等都可能激活免疫系统，从而诱发NMOSD复发。*	**日常防护：** 在流感高发季节或人员密集的封闭场所，建议佩戴口罩。养成勤洗手的习惯，注意饮食卫生，多喝水以预防泌尿系感染。注意天气变化，及时增减衣物。**3. 避免过度劳累，保证充足睡眠***	**劳逸结合：** 身体的极度疲劳会使免疫系统处于不稳定状态。日常工作和家务应量力而行，避免熬夜和透支体力。*	**适度运动：** 鼓励进行散步、瑜伽、太极等温和的运动，但要避免剧烈运动和过度出汗，同时避免在高温环境下长时间停留（体温升高可能会使原有的神经症状暂时加重）。**4. 管理情绪与压力***	**保持平稳心态：** 长期的精神紧张、焦虑、抑郁或突然的重大精神创伤，都会对内分泌和免疫系统产生负面影响。寻找适合自己的减压方式，必要时可寻求心理咨询。**5. 疫苗接种需高度谨慎***	**接种前必问诊：** 由于患者通常在使用免疫抑制药物，接种疫苗（尤其是减毒活疫苗）可能会引发严重的感染或诱发疾病复发。在接种任何疫苗（如流感疫苗、带状疱疹疫苗等）之前，务必先咨询您的神经内科或眼科主治医师。**6. 戒烟限酒***	吸烟已被证实会加重多种自身免疫性疾病的病情并影响药物疗效，强烈建议戒烟，并尽量避免二手烟。**7. 密切监测，警惕复发“信号”***	请患者牢记，一旦出现以下任何疑似复发的症状，不要等待自行缓解，必须在**第一时间（最好24小时内）**就医：*	单眼或双眼视力突然下降、视野缺损、眼球转动时疼痛。*	肢体麻木、无力、针刺感或束带感（感觉胸腹部被紧紧勒住）。*	顽固性的打嗝、恶心、呕吐（持续数日不能缓解）。*	大小便困难或失禁。您可以将这些要点打印成小册子或通过口头宣教的方式传达给患者，帮助他们在日常生活中建立起防范意识。

Qwen

您好！作为您的 AI 健康助手，我很乐意协助眼科医师为您梳理视神经脊髓炎谱系疾病（NMOSD）的日常管理要点。NMOSD 是一种自身免疫性疾病，主要攻击视神经和脊髓。虽然目前无法彻底治愈，但通过科学的自我管理，可以显著降低复发风险，保护您的视力和肢体功能。以下是为您整理的**核心注意事项**：### 1. 严格遵医嘱用药（重中之重）这是预防复发的基石。NMOSD 的复发往往具有破坏性，且不可逆。- **切勿擅自停药或减量**：即使您感觉身体完全正常，体内的免疫异常可能依然存在。突然停用免疫抑制剂（如硫唑嘌呤、麦考酚钠等）或生物制剂（如利妥昔单抗、伊奈利珠单抗等）极易诱发严重复发。- **定期复查**：按照医生要求定期检查血常规、肝肾功能及免疫指标，以便医生及时调整药物剂量，平衡疗效与副作用。### 2. 警惕感染，做好防护。感染是 NMOSD 复发最常见的诱因之一。由于您可能正在服用免疫抑制药物，抵抗力相对较弱。- **呼吸道防护**：在流感高发季或前往人群密集场所时，请佩戴口罩。- **个人卫生**：勤洗手，注意口腔卫生，避免泌尿系统感染。- **疫苗接种**：在接种任何疫苗（尤其是减毒活疫苗）前，**务必先咨询您的主治医生**。部分疫苗可能在免疫抑制期间禁忌，而灭活疫苗通常需要在病情稳定期接种。### 3. 生活方式的调整。健康的生活习惯有助于维持免疫系统的稳定。- **避免过度疲劳**：保证充足的睡眠，避免熬夜和重体力劳动。疲劳是许多患者 reported 的复发前兆。- **温度管理**：部分患者对热敏感（ Uhthoff 现象），高温环境（如桑拿、热水浴、烈日暴晒）可能导致症状暂时加重，虽不一定是真复发，但应尽量避免极端温度刺激。- **均衡饮食**：无需特殊忌口，但建议清淡饮食，多摄入富含维生素和蛋白质的食物。若长期服用激素，需注意补钙和维生素 D，预防骨质疏松。### 4. 识别早期预警信号NMOSD 的复发通常起病急骤。一旦出现以下症状，请**立即就医**，不要等待观察：- **眼部症状**：单眼或双眼视力突然下降、眼球转动痛、视野缺损、色觉异常。- **脊髓症状**：肢体麻木、无力、束带感（感觉胸部或腹部像被带子勒住）、大小便功能障碍（尿潴留或失禁）。- **脑部症状**：顽固性呃逆（打嗝）、恶心呕吐（延髓极后区综合征表现）、意识障碍。> **重要提示**：治疗复发的“黄金窗口期”很短（通常为发作后数天内）。越早使用大剂量激素冲击或其他急救治疗，恢复的可能性越大，遗留后遗症越少。### 5. 心理支持与情绪管理。长期的慢性病管理可能会带来焦虑或抑郁情绪，而巨大的精神压力也可能成为复发的诱因。- 保持乐观心态，必要时寻求心理咨询。- 加入病友互助组织，分享经验，获得情感支持。### 给患者的特别叮嘱。NMOSD 的管理是一场“马拉松”，而不是“短跑”。请您与眼科医师及神经内科医师保持紧密合作，建立个人的**健康档案**，记录每次用药、复查结果及身体变化。如果您有任何突发的不适，请记住：**宁可虚惊一场，不可延误时机**。祝您病情稳定，生活愉快！

Deepseek

您好，作为眼科医师的助手，我非常理解您对减少NMOSD（视神经脊髓炎谱系疾病）复发风险的关切。NMOSD是一种需要长期管理的自身免疫性疾病，预防复发是治疗的核心目标。为了帮助您更好地管理疾病，降低突然复发的风险，请您务必在主治医师（神经内科和/或眼科医师）的指导下，重点关注以下几个方面：### 一、 核心基石：坚持规范治疗与定期随访。这是**最重要、最有效**的预防措施。1. **坚持长期预防治疗**：NMOSD的复发预防需要长期使用免疫抑制剂或生物制剂（如利妥昔单抗、萨特利珠单抗、伊奈利珠单抗等）。**切勿自行停药、减量或更改用药方案**，这是诱发复发最常见的原因。2. **定期复查与监测**：*	**定期复诊**：严格按照医嘱时间复诊（神经内科、眼科）。*	**关键检查**：*	**血液检查**：监测血常规、肝肾功能、免疫功能（如IgG水平）、以及**水通道蛋白4抗体**滴度（AQP4-IgG）的变化。抗体滴度显著升高可能提示复发风险增加。*	**影像学检查**：定期进行**脊髓和头颅的磁共振**检查，即使没有症状，也能早期发现无症状的炎症活动*	**眼科检查**：定期进行视力、视野、光学相干断层扫描等检查，评估视神经健康状况。### 二、 生活方式与日常注意事项1. **避免感染**：*	感染是诱发NMOSD复发的重要诱因。请注意个人卫生，勤洗手，在流感季节或人群密集处佩戴口罩。*	一旦出现发热、咳嗽、腹泻等感染症状，应及时就医并告知医生您的NMOSD病史，因为某些感染可能需要调整免疫治疗方案。*	**疫苗接种**：接种疫苗前**必须咨询您的主治医师**。通常建议接种灭活疫苗（如流感疫苗、肺炎球菌疫苗），但应避免接种减毒活疫苗。2. **管理压力与保证休息**：*	长期精神压力、焦虑、情绪剧烈波动和**过度疲劳、睡眠不足**是常见的复发诱因。*	尝试学习压力管理技巧，如冥想、深呼吸、听音乐等。*	**保证充足、规律的睡眠**，避免熬夜和过度劳累。3. **谨慎应对体温升高**：*	NMOSD患者可能出现“乌托夫现象”，即体温轻微升高（如洗热水澡、运动后、发热）导致原有神经症状一过性加重。*	避免长时间洗热水澡、蒸桑拿、日光暴晒。发热时及时采取物理降温（如温水擦浴）并就医。4. **均衡营养与适度活动**：*	保持均衡饮食，确保营养充足。目前没有特殊的“NMOSD饮食”，但健康的饮食有助于维持整体免疫状态。*	在身体允许的情况下，进行**适度、温和的康复锻炼**（如散步、瑜伽、太极），有助于改善体能和情绪，但避免剧烈运动导致体温过高和过度疲劳。5. **女性患者特别注意事项**：*	**妊娠与分娩**：计划怀孕**必须提前与神经科和产科医生共同制定详细计划**。孕期和产后是复发的高风险期，需要严密监测和可能的治疗调整。*	某些激素变化可能影响病情，需与医生保持沟通。### 三、 识别复发早期迹象，立即就医。即使做好了所有预防，仍需保持警惕。如果出现以下**任何新发症状或原有症状加重**，应**立即联系您的主治医师或前往医院**：*	**眼部**：视力下降、视野缺损、眼球转动疼痛。*	**脊髓**：肢体麻木、无力、感觉异常（如束带感）、大小便困难或失禁。*	**脑部**：顽固性呃逆、恶心呕吐、嗜睡、头晕、平衡障碍等。**总结与核心建议**：请您与您的医疗团队建立牢固的合作关系。**坚持规范用药、定期复查、避免诱因、警惕症状**，是您手中最有力的武器。请随身携带您的疾病诊断卡片，在急诊时告知医生您患有NMOSD及正在使用的药物。本信息旨在提供健康教育和支持，不能替代专业医疗建议。所有治疗和生活方式调整都应在您的主治医师指导下进行。祝您管理顺利，保持稳定！

15. 如果我觉得身体还好，可以自己停药或减少 NMOSD 的药量吗？

Gemini

**绝对不可以自行停药或减量。**作为医生，您可以向患者传达以下几个核心观点，帮助他们理解维持用药的重要性：**1. “感觉还好”正是药物在起效的证明**NMOSD（视神经脊髓炎谱系疾病）是一种慢性的自身免疫性疾病。患者在缓解期“感觉身体还好”，并不是因为疾病已经彻底消失或自愈了，而是因为当前的药物成功地压制了体内异常的免疫攻击。**2. 停药的代价极高，损伤通常不可逆**NMOSD最大的特点是“高复发、高致残”。每一次复发都可能对视神经或脊髓造成新的、不可逆的破坏。自行停药或减量极易诱发疾病反扑，可能导致突然的视力断崖式下降（甚至永久失明）、肢体瘫痪或大小便失禁。预防复发是治疗NMOSD的重中之重，不能拿现有的视力和神经功能去冒险。**3. 一个通俗的比喻**您可以对患者说：“吃药就像在下雨天打伞。你现在觉得身上没湿（感觉身体还好），是因为伞（药物）替你挡住了雨（免疫攻击）。如果你因为身上没湿就把伞收起来，马上就会被暴雨淋透（疾病复发）。”**4. 调整药物必须有科学依据**如果患者是因为药物副作用、经济压力、或者有备孕需求而想要停药或减量，请务必鼓励他们直接向您或神经内科医生坦诚沟通。医生可以通过评估临床症状、复查核磁共振（MRI）、检测血液中的AQP4抗体滴度等客观指标，在安全的前提下为您调整治疗方案或更换药物，而不是盲目地自行决定。**总结给患者的一句话：**“保护您现在的视力和健康状态是我们共同的目标，在没有医生指导的情况下，请务必坚持规律用药，这是防止复发最有效的武器。”

Qwen

**绝对不可以。**作为眼科医师，我必须非常严肃且明确地告诉您：**即使您感觉身体完全健康、视力正常，也绝不能自行停药或减少 NMOSD（视神经脊髓炎谱系疾病）的药物剂量。**NMOSD 是一种慢性、复发性、致残性的自身免疫性疾病。以下是您需要了解的关键医学事实，请务必仔细阅读：### 1. “感觉良好”不等于“疾病治愈”NMOSD 的治疗分为两个阶段：- **急性期治疗**：当发作时（如视力突然下降、肢体无力），使用激素或血浆置换来挽救功能。- **缓解期治疗（预防复发）**：这就是您目前正在服用的药物（免疫抑制剂或生物制剂）。**关键点**：您现在的“感觉良好”，恰恰是因为药物正在体内发挥作用，压制了异常的免疫反应。一旦停药或减量，被压制的免疫系统会迅速反弹，导致疾病复发。NMOSD 目前尚无法彻底根治，需要长期甚至终身维持治疗。### 2. 复发的代价是惨痛的NMOSD 最可怕的特点是"**累积性残疾**"- 每一次复发，都会对您的视神经或脊髓造成新的、往往是**不可逆**的损伤。- 第一次发作可能导致视力模糊，第二次可能导致单眼失明，第三次可能波及双眼或导致瘫痪。- **眼科视角的警示**：视神经一旦萎缩，目前的医疗手段无法使其再生。自行停药导致的复发，极大概率会造成永久性的视力丧失，甚至失明。### 3. 为什么不能自己减量？药物剂量的调整是一个极其精密的医疗决策，必须基于：- 血液检查指标（如 AQP4 抗体滴度、肝肾功能、血常规等）。- 影像学检查结果（MRI 是否有新发病灶）。- 临床评估（神经系统查体）。医生会根据这些客观数据，在确保疾病不复发的前提下，极其谨慎地尝试微调方案。**患者自我的主观感觉（“我觉得好了”）不能作为减药的依据。**### 4. 如果您担心副作用怎么办？很多患者想停药是因为担心长期的药物副作用（如感染风险、肝肾负担等）。这是可以理解的，但正确的做法是：- **不要沉默**：立即与您的主治医生沟通您的担忧。- **寻求替代方案**：现代医学有很多不同机制的药物（如利妥昔单抗、伊奈利珠单抗、萨特利珠单抗等），如果当前药物副作用大，医生可以为您更换更安全或耐受性更好的药物，而不是直接停药。### 总结与建议。为了您的光明未来，请遵守以下原则：| 错误做法 |正确做法 || 感觉好了就自行停药 | **严格遵医嘱按时按量服药** || 觉得药量太大自己减半 | **定期复查，由医生评估是否调整** || 听信偏方或非专业建议 | **只听从神经内科或眼科专科医生的指导** || 出现不适默默忍受 | **及时就医，讨论换药或对症处理** |**医师寄语**：NMOSD 的管理是一场马拉松，而不是短跑。您现在的每一次按时服药，都是在为未来的视力买保险。请不要拿您宝贵的视力去冒险。如果您有任何关于药物的疑虑，请在下一次复诊时详细与我们讨论，我们共同寻找最适合您的治疗方案。

Deepseek

\# 关于NMOSD用药的重要提醒**绝对不可以自行停药或减少药量。**即使您感觉身体良好，自行调整药物也是非常危险的行为。以下是您需要了解的关键信息：## 为什么不能自行停药或减药？1. **NMOSD是一种慢性自身免疫性疾病**- 疾病在“表面平静”时，免疫系统异常可能仍在活跃- 感觉良好不代表疾病已治愈或炎症已完全控制2. **预防复发是关键**- NMOSD药物主要目的是预防复发，而非治疗已出现的症状- 每次复发都可能导致不可逆的神经损伤和视力丧失- 即使轻微复发也可能累积成严重的功能障碍3. **药物调整需要专业评估**- 医师会根据多项指标评估疾病活动性，包括：- 近期MRI检查结果- 血液中AQP4抗体水平（如适用）-其他免疫指标- 您的整体健康状况## 如果您考虑调整用药，应该怎么做？1. **与您的眼科医师和神经科医师沟通**- 表达您的顾虑和感受- 讨论任何药物副作用或经济负担2. **在医师指导下进行调整**- 任何剂量调整都需基于专业评估- 可能需要逐步调整而非突然停药- 调整期间可能需要更密切的监测3. **了解风险与收益**- 医师会帮助您权衡维持治疗与调整治疗的风险- 某些情况下，在严密监控下调整方案可能是合适的## 紧急情况。如果您遇到以下情况，请立即联系医疗团队：- 出现新的神经系统症状（视力变化、肢体无力、感觉异常等）- 严重药物副作用- 计划怀孕或已怀孕**请记住**：NMOSD治疗是长期管理过程，维持稳定的药物方案是保护视力和神经功能的最有效方法。您感觉良好正是药物起效的表现，而不是停止治疗的理由。如果您对当前治疗方案有任何疑问或顾虑，请预约与您的医疗团队讨论，而不是自行做出改变。

16. 得了 NMOSD，是不是很多运动都不能做了，比如跑步或游泳？

Gemini

得了NMOSD并不意味着要完全放弃运动。相反，在病情稳定期（缓解期），适度、规律的运动对NMOSD患者非常有益，可以帮助改善疲劳、增强肌肉力量、提高平衡能力并缓解情绪压力。跑步和游泳都是可以进行的，但需要根据患者的具体身体状况进行调整。您可以这样向患者解释：1. **游泳是非常推荐的运动：** 水的浮力可以减轻关节和肌肉的负担，降低因平衡感差而跌倒的风险。更重要的是，水温通常较低，可以防止体温过高引发的“乌托夫现象”（Uhthoff's phenomenon，即体温升高导致视力模糊、肢体无力等神经症状暂时加重），这对脱髓鞘疾病患者非常友好。2. **跑步也可以，但需量力而行：** 如果患者的视力、下肢力量和平衡能力允许，完全可以进行慢跑。但要注意选择平坦、熟悉的场地以防跌倒，并避免在炎热的天气下跑步。3. **运动的核心原则：**避免过热：** 保持运动环境凉爽，运动前后注意补充水分。**倾听身体：** 感到疲劳或出现症状波动时，应立即停止并休息，不要强迫自己完成目标。**循序渐进：** 从低强度、短时间（如每次10-15分钟）开始，身体适应后再逐渐增加。**避开急性期：** 在疾病急性发作期应以休息和接受医学治疗为主，暂停锻炼。建议患者在开始任何新的运动计划前，最好能结合自身的神经功能缺损情况，咨询神经内科医生或专业的康复理疗师，制定个性化的方案。需要我为您提供一份更详细的、可直接打印给患者阅读的“NMOSD患者日常运动与生活指南”吗？

Qwen

您好！作为眼科医师，在接诊视神经脊髓炎谱系疾病（NMOSD）患者时，除了关注视力保护，对患者进行生活方式和运动指导也是康复管理的重要一环。关于"**得了 NMOSD 是否就不能跑步或游泳了**"这个问题，答案通常是：**并不是完全不能做，但需要根据病情阶段、身体状况和环境因素进行科学调整和严格防护**。以下是为您准备的患教内容要点，您可以直接用于与患者的沟通或制作宣教材料：### 核心原则：分期管理与个体化评估。NMOSD 患者的运动能力并非“一刀切”地被禁止，而是取决于疾病是处于**急性发作期**还是**缓解期**。#### 1. 急性发作期（Absolute Rest）*	**状态**：出现视力下降、肢体无力、感觉异常等新发症状时。*	**建议**：**严禁**剧烈运动（如跑步、游泳）。*	**原因**：此时神经系统处于高度炎症状态，过度劳累可能加重损伤，延缓恢复。首要任务是遵医嘱使用激素或免疫抑制剂治疗，绝对卧床休息。#### 2. 缓解期/稳定期（Controlled Activity）*	**状态**：症状稳定，无新发体征，体力有所恢复。*	**建议**：**鼓励**适度运动。*	**益处**：适度的有氧运动可以改善心肺功能，预防肌肉萎缩，缓解抑郁焦虑情绪，提高生活质量。*	**关键限制**：必须避免**体温过高**（Uhthoff 现象）和**过度疲劳**。针对具体运动的分析与建议#### 关于跑步*	**风险点**：跑步产热快，容易导致核心体温升高。NMOSD 患者对热敏感，体温升高可能导致暂时性的传导阻滞，使旧症状复发或加重（即 Uhthoff 现象），虽然这通常是可逆的，但会引起恐慌和不适。此外，若患者存在下肢无力或平衡障碍，跑步有跌倒风险。*	**医师建议**： *	若平衡能力好且无严重下肢无力，可尝试**慢跑**，但需严格控制时间和强度。*	**环境选择**：避免在高温、高湿天气下户外跑步。建议选择清晨、傍晚或在恒温空调房内跑步机上进行。*	**替代方案**：如果跑步导致过热或疲劳，建议改为**快走**。#### 关于游泳*	**优势**：水的浮力可以减轻关节负担，水温通常低于体温，有助于散热，理论上比跑步更适合怕热的患者。*	**风险点**：*	**感染风险**：NMOSD 患者常长期使用免疫抑制剂，抵抗力较低。公共泳池的细菌可能增加尿路感染或呼吸道感染风险，而**感染是诱发 NMOSD 复发的最常见因素之一**。 *	**安全性**：若患者存在肢体麻木、无力或突发痉挛，在水中发生溺水的风险极高。*	**水温过低**：过冷的水也可能诱发痉挛。*	**医师建议**：*	**首选私人泳池**或水质极佳、人少的场所，严格注意卫生。 *	**必须有人陪同**，严禁独自游泳。*	下水前充分热身，出水后立即保暖擦干，防止感冒。*	若正在使用大剂量免疫抑制剂，建议暂停公共泳池活动。### 运动中的“红线”与注意事项。为了安全起见，请提醒患者遵守以下"**三不**"原则：1. **不过热**：一旦感到身体发热、出汗过多或原有症状（如视力模糊、肢体麻木）因受热而暂时加重，必须立即停止运动并降温。*	*公式化理解体温影响*：神经传导速度与温度相关，脱髓鞘神经纤维在高温下传导阻滞风险显著增加。2. **不力竭**：运动应以“微汗、稍感疲劳但休息后可迅速恢复”为度，避免精疲力竭。3. **不独行**：鉴于 NMOSD 可能突发肢体无力或眩晕，运动时最好有家属或朋友陪同。###给眼科医师的沟通话术示例> “王先生/女士，得了 NMOSD 并不意味着您要从此告别运动场。相反，在病情稳定的时候，科学的运动对您的神经修复很有帮助。> 但是，我们需要像保护眼睛一样保护您的神经系统。**跑步**要注意别让自己太热，因为热量会让神经信号传导变慢，让您觉得症状加重；**游泳**是个好选择，能帮您散热，但因为您吃着免疫药，一定要去干净的水域，并且千万别一个人去，以防万一抽筋或无力发生危险。> 我们的目标是：**动起来，但别累着，别热着，别感染着**。如果您在运动中感觉视力有任何波动或肢体不对劲，马上停下来，我们随时调整方案。”###总结| 运动项目 | 推荐指数 (缓解期) | 关键风险提示 | 医师建议措施 || **跑步** | 体温过高 (Uhthoff 现象)、跌倒 | 低温环境、缩短时间、监测心率 || **游泳** | 感染诱发复发、溺水风险 | 洁净水源、专人陪同、防寒保暖 || **瑜伽/太极**| 平衡失控 | 动作舒缓、避免高温瑜伽 || **力量训练**| 过度疲劳 | 轻重量、多组数、有人辅助 |希望这些信息能帮助您更好地为患者提供专业、温暖且实用的指导！

Deepseek

\# NMOSD患者运动指导：安全与健康并重。首先，请理解我的回答不能替代专业医疗建议。您应该与您的神经科医生和康复科医生讨论个性化的运动方案。以下是一般性指导：## 核心原则：个体化与安全性。NMOSD（视神经脊髓炎谱系疾病）患者的运动能力**取决于疾病状态、神经损伤程度和个人整体健康状况**，而非一概而论。### 可以进行的运动（在医生评估后）| 运动类型 | 注意事项 | 潜在益处 || **低冲击有氧运动** | 心率监测，避免过热 | 改善心血管健康，提升情绪 || **游泳/水中运动** | 水温适宜（避免过冷），有监护 | 低关节压力，增强肌肉力量 || **瑜伽/太极** | 避免过度拉伸，选择温和流派 | 改善平衡、柔韧性和压力管理 || **力量训练** | 轻到中度重量，注重形式 | 维持肌肉质量，支持日常功能 |### 需要谨慎评估的运动1. **跑步**：取决于平衡能力、视觉功能和疲劳程度- 平坦地面优于不平坦地形- 考虑使用跑步机（有扶手）而非户外跑步- 注意体温调节（NMOSD患者可能对温度敏感）2. **高强度/接触性运动**：如篮球、足球等- 增加跌倒和碰撞风险- 可能诱发疲劳或症状加重## 关键考虑因素### 1. 疾病活动期 vs. 稳定期- **活动期**：通常建议休息，避免加重炎症- **稳定期**：在医生指导下逐步引入适当运动### 2. 具体症状考量- **视力障碍**：选择对视觉要求低的运动，使用适当辅助- **平衡问题**：优先坐姿或支持性运动- **疲劳管理**：短时间、分散的运动优于长时间持续运动- **温度敏感**：避免过热环境，注意运动时降温### 3. 安全第一原则- 运动前咨询医生，特别是调整药物或疾病状态变化时- 从低强度开始，缓慢增加- 倾听身体信号，出现疼痛、过度疲劳或症状加重时停止- 考虑与物理治疗师合作制定个性化计划## 运动对NMOSD的潜在益处。适量运动可能有助于：- 改善心血管健康- 维持肌肉力量和关节灵活性- 管理体重（某些药物可能导致体重增加）- 提升情绪和生活质量- 可能改善疲劳（适度运动可增加能量水平）## 给您的具体建议：1. **与医疗团队沟通**：在开始任何新运动前，咨询您的神经科医生和康复专家2. **循序渐进**：从5-10分钟开始，逐渐增加时长和强度3. **记录反应**：记录运动类型、时长和身体反应，帮助医生优化建议4. **考虑监督环境**：初期可在康复中心或物理治疗师指导下进行**最重要的是**：NMOSD患者**不必完全避免运动**，但需要智慧地选择适合自己当前状况的活动。许多患者通过适度运动显著改善了生活质量。请与您的医疗团队讨论这些建议，制定适合您个人情况的运动计划。

17. NMOSD 复发频率与季节变化有关吗？

Gemini

目前关于视神经脊髓炎谱系疾病（NMOSD）复发与季节变化的直接关系，医学界尚无绝对定论，但**季节交替带来的环境变化确实会间接增加复发风险**。您可以从以下几个关键点对患者进行教育：1. **感染是核心诱因（冬春季高发）：** 季节交替（尤其是冬春季）是上呼吸道感染、流感和肠道病毒感染的高发期。病原体感染会激活机体免疫系统，是诱发NMOSD复发的最常见且最明确的危险因素。2. **温度变化与应激：** 气温骤降或极端的冷热刺激会引起机体应激反应。此外，环境温度过高（如夏季酷暑）可能导致患者原有的神经功能缺损症状一过性加重（类似假性复发），引起患者恐慌。3. **日照与维生素D（秋冬季减少）：** 秋冬季日照时间缩短可能导致体内维生素D水平下降。虽然维生素D缺乏与多发性硬化（MS）的关联更为确切，但越来越多的研究提示，低维生素D水平也可能对NMOSD患者的免疫稳态产生不利影响。**建议向患者强调的行动指南：***	**严防感染：** 在流感季和季节交替时，务必注意戴口罩、勤洗手，避免前往人群密集的封闭场所。*	**平稳过渡：** 随天气变化及时增减衣物，避免受凉感冒；夏季注意防暑降温。*	**坚持规范治疗：** 强调NMOSD的维持治疗是长期的。无论处于哪个季节、自我感觉多么良好，都必须严格遵医嘱使用免疫抑制剂或生物制剂，绝不能因季节变化自行减药或停药。需要我为您进一步提供关于NMOSD患者日常护理或用药依从性教育的详细资料吗？

Qwen

您好！作为眼科医师的助手，我为您整理了一份针对**视神经脊髓炎谱系疾病（NMOSD）**患者的教育资料。这份资料旨在解答患者关于“复发频率与季节变化”的疑问，并强调眼部症状监测的重要性。患者教育指南：NMOSD 复发与季节变化的关系。亲爱的患者朋友：很多病友都在问：“我的病情会不会像过敏一样，到了春天或冬天就容易发作？”这是一个非常重要的问题。以下是基于当前医学研究的详细解答。#### 1. 核心结论：季节性关联尚不明确。目前的医学研究数据显示，**NMOSD 的复发与季节变化之间没有像多发性硬化（MS）那样明确的、一致的强相关性。**- **多发性硬化 (MS)**：研究表明 MS 的复发在春季和初夏往往较高，可能与病毒感染或日照维生素 D 水平变化有关。- **NMOSD**：多项大规模回顾性研究并未发现 NMOSD 复发存在显著的“季节性高峰”。这意味着，**一年四季中，任何时间点都可能发生复发**。#### 2. 为什么会有“季节影响”的错觉？虽然疾病本身可能没有季节性，但**诱发因素**可能具有季节性，从而间接导致复发风险波动：| 潜在诱发因素 | 季节关联 | 对 NMOSD 的影响机制 || **感染** | 冬季/春季高发 | 呼吸道或胃肠道感染是 NMOSD 复发最常见的诱因。流感季节需格外警惕。 || **疫苗接种** | 秋季/特定时期 | 部分患者在接种疫苗后可能出现免疫反应波动（需遵医嘱评估）。 || **产后状态** | 无季节性 | 分娩后（尤其是前 3-6 个月）是复发的高危期，与季节无关。 || **停药/减药** | 人为因素 | 不规律服药或自行减量是导致复发的首要原因，与季节无关。 |#### 3. 眼科医师的特别叮嘱。作为眼科医生，我们要特别提醒您关注**视神经炎**这一 NMOSD 最常见的首发或复发症状。无论什么季节，一旦出现以下迹象，请**立即**就医，不要等待：*	**视力急剧下降**：通常在数小时至数天内发生。*	**眼球转动痛**：转动眼睛时感到疼痛。*	**视野缺损**：看东西中间有黑影，或者两边看不见。*	**色觉异常**：感觉颜色变淡，尤其是红色。> **重要提示**：NMOSD 导致的视神经炎往往比多发性硬化更严重，且恢复较差。**“时间就是视力”**，早期大剂量激素冲击治疗至关重要。#### 4. 全年通用的预防策略#### 5. 给患者的行动清单1. **不要因季节放松警惕**：夏天不要因为感觉良好就自行减药，冬天不要因为怕冷而减少户外活动导致维生素 D 缺乏（可在医生指导下补充）。2. **预防感染是关键**：在冬春流感高发季，注意保暖，避免去人群密集场所，必要时咨询医生是否接种灭活疫苗。3. **建立“视力日记”**：简单记录每天的视力感受，以便复诊时向医生提供准确信息。4. **紧急联络卡**：随身携带疾病诊断卡，注明"AQP4 抗体阳性/NMOSD"，以便在突发视力丧失时，急诊医生能迅速识别并给予正确治疗。**医师寄语**：NMOSD 是一场持久战，虽然它不像某些疾病那样有明显的“季节规律”，但只要我们坚持规范治疗、严密监测，就能最大程度地保护您的视力和神经功能。如有任何疑虑，请随时与我们沟通。*(注：本内容仅供参考，具体诊疗方案请务必遵循您的主治医生建议。)*

Deepseek

根据现有研究，**NMOSD的复发频率与季节变化之间没有明确、一致的关联性**。目前，没有强有力的证据表明特定季节（如冬季或夏季）会普遍导致NMOSD复发率显著升高。不过，在向患者进行教育时，可以基于现有研究和临床观察，提供以下更全面的信息：### 1. **核心结论：无明确季节性规律** 多数大型研究和临床数据并未发现复发率随季节呈现规律性波动。复发更可能与个体自身的免疫状态、感染、应激等因素直接相关。### 2. **可能相关的间接因素**虽然季节本身不是直接诱因，但某些**与季节相关的因素**可能间接影响复发风险，需要提醒患者注意：- **感染**：秋冬季是上呼吸道感染（如感冒、流感）高发期，感染可能激活免疫系统，**可能**触发复发。- **维生素D水平**：冬季日照减少，可能导致维生素D水平降低。一些研究提示低维生素D水平可能与自身免疫疾病活动度有关，但其与NMOSD复发的直接因果关系尚未确立。- **应激与作息**：季节变化可能影响生活作息、情绪和心理压力，而**应激是已知的潜在复发诱因之一**。### 3. **个体差异的重要性**- 少数患者可能主观感觉在特定季节更容易复发，这可能与他们的个人触发因素（如对特定季节的过敏原、感染频率等）有关。应鼓励患者记录自己的“复发日记”，观察个人规律。### 4. **给患者的实用建议**- **坚持预防治疗**：无论季节如何，**坚持使用免疫抑制剂或生物制剂（如利妥昔单抗、依库珠单抗等）进行长期预防是降低复发风险最有效的方法**。- **管理感染风险**：在感染高发季节注意防护（如接种流感疫苗、勤洗手、戴口罩），感染后密切观察症状。- **保持健康习惯**：均衡饮食、适度锻炼、管理压力、保证充足日照（或遵医嘱补充维生素D），维持整体免疫稳定。- **及时识别复发**：教育患者识别复发早期症状（如视力下降、肢体麻木无力、疼痛等），并强调**一旦出现新症状或旧症状加重，应立即联系医疗团队，无需等待季节变化结束**。### 总结陈述建议：“目前没有证据表明NMOSD复发会像某些过敏性疾病那样随季节规律性变化。复发主要与个体免疫状态、感染等因素相关。因此，无论什么季节，坚持规范治疗、避免诱因、密切监测症状都是最重要的。”这样的解释既基于当前证据，又能帮助患者避免不必要的季节焦虑，将注意力集中在可控的预防措施上。

18. 生物制剂治疗 NMOSD 需要终身使用吗？

Gemini

目前，针对视神经脊髓炎谱系疾病（NMOSD），生物制剂治疗通常被建议为**长期甚至终身使用**。为了协助您向患者进行清晰、易懂且具有同理心的宣教，建议从以下几个核心方面向患者解释：**1. 明确治疗目的：预防复发是核心***	**通俗解释：** NMOSD是一种高复发率的自身免疫性疾病。每一次复发都像是一次“地震”，会对视神经和脊髓造成不可逆的损伤（如视力严重下降甚至失明、肢体瘫痪等）。*	**生物制剂的作用：** 生物制剂（如伊奈利珠单抗、萨特利珠单抗、依库珠单抗或利妥昔单抗等）的作用相当于给过度活跃的免疫系统“踩刹车”，防止它错误地攻击自身的视神经。长期用药是为了维持这把“保护伞”，而不是仅仅为了缓解眼前的症状。**2. 为什么不能在感觉良好时停药？***	**停药风险：** 即使患者感觉视力已经稳定，或者很长时间没有不适，体内的异常免疫反应仍可能在潜伏。一旦擅自停药或拉长用药间隔，免疫系统失去压制，极易导致疾病“反扑”。*	**防患于未然：** 预防下一次复发，远比复发后再去抢救视力和神经功能要重要得多。失去的视力往往很难完全找回。**3. “终身”意味着一成不变吗？***	**动态评估与调整：** 虽然大原则是长期用药，但治疗方案是动态的。医生会定期评估患者的病情控制情况、药物耐受性、副作用以及是否有备孕/生育需求等，从而对药物种类或给药方案进行个体化调整。*	**医学的进步：** 随着医学的快速发展，未来可能会有更先进、甚至能彻底治愈的手段出现。但在那之前，坚持现有的有效治疗是保护视力的最佳选择。**4. 给患者的日常行动指南***	严格遵医嘱按时用药，切勿自行停药或减量。*	定期在眼科和神经内科复查（包括视力视野检查、OCT、血液指标和核磁共振等），监测药物疗效和潜在副作用。*	如果出现任何新的视力模糊、眼痛、肢体麻木无力或频繁打嗝呕吐等症状，应立即就医。需要我为您将这些要点转化为一份可以直接打印发给患者的“患者宣教单页”草稿吗？

Qwen

您好！作为眼科医师的助手，针对**视神经脊髓炎谱系疾病**（NMOSD）患者关于“生物制剂是否需要终身使用”的疑问，以下是一份专业的患者教育内容草案。您可以直接用于临床沟通或制作宣教材料。###患者教育：NMOSD 与生物制剂治疗的长期性#### 1. 核心结论：通常需要长期维持，但非绝对“终身”对于大多数 NMOSD 患者而言，生物制剂（如伊奈利珠单抗、萨特利珠单抗等）的治疗目标是**预防复发**。由于 NMOSD 是一种高复发率、致残率高的自身免疫性疾病，目前的国际共识和临床指南建议：**只要疾病处于活动期或复发风险较高，就需要持续进行免疫抑制治疗**。*	**一般情况**：多数患者需要接受**数年甚至终身**的维持治疗。*	**特殊情况**：在极少数特定条件下（如长期无复发、抗体转阴、出现严重副作用或患者有强烈生育计划等），经专家评估后可能尝试减量或停药，但这必须在严密监控下进行。#### 2. 为什么要长期用药NMOSD 的病理基础是体内产生了攻击视神经和脊髓的错误抗体（主要是 **AQP4-IgG**）。*	**复发即损伤**：NMOSD 的每一次复发都可能导致不可逆的视力下降或肢体瘫痪。*	**药物机制**：生物制剂的作用是清除产生抗体的 B 细胞或阻断炎症通路。一旦停药，免疫系统可能会重新“失控”，导致抗体滴度回升，引发复发。*	**数据支持**：研究表明，擅自停药患者的年复发率显著高于持续治疗者。#### 3. 什么情况下可以考虑调整或停药？虽然默认策略是长期治疗，但在以下情形下，医生会与患者讨论调整方案：1. **长期缓解**：患者在规范治疗下，连续 **5 年以上** 无任何临床复发，且影像学（MRI）无新发病灶。2. **血清学转换**：多次检测显示 **AQP4-IgG 转为阴性**。3. **安全性问题**：患者出现了严重的药物不良反应（如严重感染、肝肾功能异常等），获益小于风险。4. **特殊生理阶段**：如女性患者计划怀孕，部分生物制剂可能需要暂停或更换为妊娠期相对安全的药物（需提前规划）。> **重要提示**：任何减药或停药的决定都必须由经验丰富的神经免疫专科医生做出，**严禁患者自行停药**。#### 4. 眼科医师的特别叮嘱。作为眼科医生，我们最关注的是您的**视力保护**。NMOSD 引起的视神经炎往往发作急骤且后果严重。*	**定期眼底与视野检查**：即使没有症状，也建议每 **3-6 个月** 进行一次光学相干断层扫描（OCT）和视野检查，以发现亚临床的视神经损伤。*	**警惕早期信号**：若出现眼球转动痛、视力模糊、色觉异常，请立即就医，不要等待下次复诊时间。*	**依从性是关键**：按时注射生物制剂是守护您光明的最重要防线。#### 5. 总结。生物制剂治疗 NMOSD 目前被视为一种**长期的慢性病管理策略**，类似于高血压或糖尿病需要长期服药控制。*	**目标**：零复发，保护视力和运动功能。*	**现状**：绝大多数患者需长期用药。*	**未来**：随着医学进步，未来可能会有更精准的指标指导安全停药，但目前请以“长期备战”的心态对待治疗。**给患者的行动建议清单**：| 事项 | 频率/时机 | 目的 || **生物制剂注射** | 严格按医嘱周期 | 维持血药浓度，抑制免疫反应 || **AQP4 抗体检测** | 每 6-12 个月 | 评估疾病活动度及停药可能性 || **眼科专项检查** | 每 3-6 个月 | 早期发现视神经受损迹象 || **全身感染筛查** | 每次给药前 | 确保用药安全，避免严重感染 || **医患沟通** | 每次复诊 | 讨论是否有减量或停药的机会 |希望这份解答能帮助您和患者更好地建立治疗信心，共同守护视力健康。如有具体病例需要深入分析，欢迎随时提问。

Deepseek

\# 生物制剂治疗NMOSD需要终身使用吗？您好，作为眼科医师，您可以向NMOSD（视神经脊髓炎谱系疾病）患者解释以下内容：## 核心答案**不一定需要终身使用，但通常需要长期维持治疗。** 治疗时长取决于患者的具体情况、疾病活动性、药物反应及个体化治疗目标。## 关键说明### 1. **治疗目标**- **诱导缓解**：在急性期或疾病活动期快速控制炎症。- **维持缓解**：长期预防复发、减少神经功能损伤累积、保护视神经和脊髓功能。### 2. **影响治疗时长的因素**- **疾病活动性**：若长期无复发（如 >3-5 年），部分患者可能考虑减量或停药，但需严密监测。- **药物安全性**：长期使用需平衡疗效与潜在风险（如感染、免疫抑制等）。- **患者意愿与生活质量**：结合患者对治疗负担的接受度。### 3. **当前生物制剂治疗策略**- **单克隆抗体**（如**依库珠单抗**、**萨特利珠单抗**、**伊奈利珠单抗**等）通常需**定期持续给药**（如每2周、每月或每6个月一次），以维持足够的血药浓度。- 若停药，疾病复发风险可能显著升高，尤其是AQP4抗体阳性患者。## 患者教育要点1. **个体化方案**：治疗时长由神经科/眼科医师根据您的病情动态调整，**切勿自行停药**。2. **定期评估**：需通过MRI、视力/视野检查、抗体滴度等监测疾病活动。3. **长期管理**：即使症状稳定，NMOSD仍属慢性自身免疫病，多数患者需要长期治疗以预防不可逆性神经损伤（如永久性视力下降）。4. **新兴研究**：未来可能随着更精准的预后标志物或新疗法出现，治疗策略可能优化。## 建议沟通方式> “生物制剂像一道‘防火墙’，长期使用是为了持续阻挡免疫系统攻击视神经和脊髓。目前建议维持治疗，但我们会定期评估，若病情持续稳定，未来可能在严密监测下调整方案。治疗目标是让您长期保持视力功能和日常生活能力。”请结合患者具体情况（如抗体状态、既往复发频率、器官损伤程度等）进一步个体化解释。如有条件，建议神经免疫专科医师共同参与决策。

19. 目前有没有针对 NMOSD 病因的根治性疗法？

Gemini

目前还没有针对NMOSD（视神经脊髓炎谱系疾病）病因的根治性疗法。在向患者进行科普和教育时，建议您参考以下沟通要点，既能客观传达医学事实，又能给予患者信心：1. **客观说明现状**：NMOSD是一种自身免疫性疾病，这意味着患者的免疫系统错误地攻击了自身的神经系统（主要是视神经和脊髓）。目前的医学手段还无法彻底“重置”免疫系统来根除病因，因此该病目前无法被彻底治愈。2. **强调疾病可控**：虽然无法根治，但NMOSD是**高度可治、可控**的。可以向患者比喻，就像高血压或糖尿病等慢性病一样，通过规范的长期用药，患者完全可以控制病情，与疾病和平共处。3. **明确治疗核心（预防复发）**：目前的治疗主要分为两部分：急性发作期的治疗（如大剂量激素冲击、血浆置换）旨在快速挽救视力和神经功能；而缓解期的长期维持治疗，核心目标是**预防复发**。需要让患者明白，NMOSD的致残率主要来自于反复发作造成的损伤累积，因此坚持长期用药至关重要。4. **传递治疗信心**：近年来NMOSD的治疗领域取得了突破性进展。除了传统的免疫抑制剂（如硫唑嘌呤、吗替麦考酚酯等），现在已有多种针对性更强、预防复发效果更好的新型靶向生物制剂（如依库珠单抗、伊奈利珠单抗、萨特利珠单抗等）投入临床使用。许多患者在规范治疗下，可以实现长期“零复发”，维持正常的生活和工作质量。如果您需要关于具体药物的详细对比、急性期标准治疗方案或患者日常护理注意事项的资料，我可以进一步为您提供。

Qwen

您好！作为眼科医师的助手，针对**视神经脊髓炎谱系疾病（NMOSD）**患者的教育咨询，以下是关于“是否存在根治性疗法”的专业解答建议。您可以参考以下内容与患者进行沟通：### 核心结论：目前尚无针对病因的“根治性”疗法。首先需要明确告知患者：**截至目前，全球医学界尚未发现能够彻底清除 NMOSD 病因、保证永不复发的“根治性”疗法。**NMOSD 是一种由自身免疫机制介导的中枢神经系统脱髓鞘疾病，其主要致病因子是**水通道蛋白 -4 抗体（AQP4-IgG）**。虽然我们无法像切除肿瘤那样通过一次手术或短期用药彻底“治愈”该病，但现代医学已经拥有了非常有效的**长期管理策略**。### 当前的治疗目标与策略。虽然没有“根治”方法，但治疗目标非常明确且可实现：1. **预防复发**：这是治疗的核心。NMOSD 具有极高的复发率，每一次复发都可能导致不可逆的视力丧失或肢体瘫痪。2. **保护残存功能**：防止进一步的神经损伤。3. **提高生活质量**：通过控制症状和减少残疾累积，让患者回归正常生活。#### 主要治疗手段分类。目前的治疗方案主要分为急性期治疗和缓解期（预防）治疗：| 治疗阶段 | 目的 | 常用手段 || **急性期治疗** | 迅速抑制炎症，挽救视力/功能 | 大剂量糖皮质激素冲击、血浆置换（PLEX）、静脉注射免疫球蛋白（IVIG） || **缓解期治疗** | **长期预防复发**（关键） | 传统免疫抑制剂、生物靶向制剂 |### 革命性的进展：生物靶向制剂。近年来，NMOSD 的治疗取得了突破性进展，特别是针对致病机理（B 细胞、IL-6 通路、补体系统）的**单克隆抗体药物**。这些药物虽非“根治”，但能极大程度地降低复发风险，部分患者在长期使用下可实现“无复发状态”。目前国际及国内指南推荐的主要靶向药物包括：1. **抗 CD19 单抗**：如伊奈利珠单抗（Ineblizumab），通过清除 B 细胞减少抗体产生。2. **抗 IL-6 受体单抗**：如萨特利珠单抗（Satralizumab），阻断炎症因子 IL-6 的信号传导。3. **抗 C5 补体单抗**：如依库珠单抗（Eculizumab），阻断补体介导的神经损伤。这些药物的临床试验数据显示，它们能将复发风险降低 **70% - 90%** 以上。### 给患者的关键教育信息。作为眼科医生，在随访中请向患者强调以下几点：*	**不要轻信“根治”广告**：目前任何宣称能“彻底断根”、“祖传秘方治愈”的宣传均缺乏科学依据，盲目停药或尝试偏方极易导致病情爆发，造成永久性失明。*	**依从性是生命线**：NMOSD 是慢性病，需要像高血压、糖尿病一样进行**终身管理**。即使感觉良好，也绝不能擅自停用预防复发的免疫抑制药物。*	**定期监测**：需定期复查 AQP4 抗体滴度、肝肾功能及眼部 OCT（光学相干断层扫描）检查，以评估视神经纤维层厚度变化。*	**早期干预**：一旦出现视力下降、眼球转动痛或肢体麻木等新发症状，必须立即就医，争取在“黄金窗口期”内进行抢救性治疗。**医师寄语**：虽然目前我们还无法承诺“彻底治愈”，但通过规范的长期治疗，绝大多数 NMOSD 患者可以有效控制病情，避免失明，拥有高质量的长寿人生。请与您的医疗团队保持紧密合作，共同守护您的光明。

Deepseek

根据当前医学证据，NMOSD（视神经脊髓炎谱系疾病）**尚无根治性疗法**。这是一种自身免疫性疾病，目前治疗策略主要集中在以下方面：### 1. **急性期治疗**- **目标**：快速抑制炎症、减轻神经损伤。- **主要方法**：- **大剂量糖皮质激素冲击治疗**（如甲泼尼龙）。- **血浆置换**（PE）或**免疫吸附**：用于激素反应不佳的重症患者。- **静脉注射免疫球蛋白**（IVIG）：可作为替代方案。### 2. **缓解期治疗（预防复发）*- **目标**：长期抑制免疫系统、降低复发率、延缓残疾进展。- **一线药物**： - **单克隆抗体**：- **萨特利珠单抗**（Satralizumab）：靶向IL-6受体。 - **伊奈利珠单抗**（Inebilizumab）：靶向CD19 B细胞。- **托珠单抗**（Tocilizumab）：靶向IL-6受体（部分国家/地区适用）。- **免疫抑制剂**：- **吗替麦考酚酯**（MMF）、**硫唑嘌呤**、**甲氨蝶呤**等（需密切监测副作用）。### 3. **对症支持治疗*- **神经痛、痉挛、疲劳等症状管理**。- **康复训练**（物理治疗、职业治疗等）。- **定期眼科与神经科随访**（监测视力、脊髓功能等）。### 4. **重要注意事项**- **AQP4抗体检测**：多数患者为阳性，阴性者需进一步鉴别诊断。- **避免诱发因素**：感染、应激、疫苗接种（需个体化评估）可能诱发复发。- **妊娠期管理**：需多学科协作调整用药。### 5. **未来研究方向*- **新型靶向药物**（如补体抑制剂、B细胞靶向疗法）处于临床试验阶段。- **干细胞移植**：探索性治疗，风险较高且证据有限。### 总结：目前NMOSD需**长期规范治疗**以控制病情，患者应遵医嘱用药、定期复查，并保持健康生活方式。任何治疗方案调整均需在专科医师指导下进行。**温馨提示**：以上信息仅供参考，具体诊疗请遵从主治医师的指导。

20. 听说 NMOSD 很难治，我是不是应该试试中药或偏方？

Gemini

面对NMOSD（视神经脊髓炎谱系疾病）患者的这种焦虑和疑问，建议您在沟通时既要理解患者“急病乱投医”的心理，又要用通俗易懂的语言向他们讲清利害关系。您可以参考以下话术和要点对患者进行教育：**1. 纠正“很难治”的观念，给予患者信心**“我非常理解您得了这个病之后焦虑的心情。以前大家觉得NMOSD难治，是因为当时没有专门针对这个病的药。但现在医学发展非常快，我们已经有了很多非常有效的药物（比如各种免疫抑制剂，以及近年来上市的多种靶向生物制剂）。只要坚持规范治疗，绝大多数患者都可以很好地控制病情，做到长期不复发，像正常人一样生活和工作。所以，这个病现在是**可防可控**的，您千万不要灰心。”**2. 讲清NMOSD的致盲/致残风险，强调规范治疗的不可替代性*“NMOSD是一种自身免疫性疾病，它的特点是‘高复发、高致残’。每一次复发，免疫系统都会对您的视神经或脊髓造成一次破坏。视神经的损伤往往是不可逆的，复发次数越多，视力越差，甚至可能导致永久失明或瘫痪。目前在全世界范围内，**只有正规的西药（免疫抑制剂或生物制剂）被科学证实能够有效抑制免疫系统的错误攻击，从而预防复发。** 保护您的视力，这是唯一的防线。”**3. 客观评价中药的作用（辅助而非替代）*“关于中药，您需要明确一点：**中药不能代替西药来预防NMOSD的复发。**如果您在坚持使用西药预防复发的前提下，想用中药来调理身体、缓解一些比如疲劳、疼痛、或者激素带来的副作用，这是可以的。但是，您必须去正规医院找专业的中医师，并且一定要告诉医生您患有NMOSD。因为有些中药（如人参、黄芪等补药）具有增强免疫力的作用，而您的病恰恰是因为免疫系统过度活跃引起的，吃错了中药反而可能**诱发病情复发**。**4. 坚决抵制偏方**

“至于偏方、秘方，**绝对不要尝试**。首先，偏方没有经过科学验证，根本无法阻止视神经的损伤；其次，很多所谓的‘神药偏方’里面偷偷掺杂了大量的不明激素，或者含有重金属，不仅会干扰您现在的正规治疗，还可能导致严重的肝肾功能衰竭。为了试偏方而把正规的药停了，导致视力突然丧失甚至瘫痪的惨痛教训，我们在临床上见过太多了。”**总结性建议（给患者的定心丸）：**“您的眼睛现在最需要的是稳定和保护。请把预防复发的任务交给正规的现代医学。我们会根据您的具体情况，为您制定最适合的治疗方案。按时用药、定期复查，有任何不舒服随时来找我，这才是保护您视力最稳妥、最有效的方法。”

Qwen

您好！作为您的 AI 健康助手，我非常理解您听到“视神经脊髓炎谱系疾病（NMOSD）难治”时的焦虑心情。这种担忧在患者中非常普遍。针对您提出的“是否应该尝试中药或偏方”的问题，我需要从眼科医师和神经免疫专家的角度，为您提供科学、客观且负责任的建议：### 核心结论：切勿擅自停用西药改用偏方**直接回答您的问题：*1. **NMOSD 确实是一种严重的慢性疾病**，如果不规范治疗，复发率极高，且每次复发都可能导致不可逆的视力丧失或肢体瘫痪2. **绝对不建议**用中药或民间偏方**替代**标准的免疫抑制/免疫调节治疗（如利妥昔单抗、伊奈利珠单抗、萨特利珠单抗或传统的激素/免疫抑制剂）3. **风险警示**：目前没有任何高质量的临床证据表明单用中药或偏方能有效预防 NMOSD 复发。盲目尝试偏方往往会导致治疗窗口期延误，造成永久性残疾。###为什么标准治疗至关重要？NMOSD 的核心病理机制是体内产生了攻击视神经和脊髓的抗体（主要是 AQP4-IgG）。我们需要通过药物强行压制这种异常的免疫反应。以下是标准治疗与偏方/非规范治疗的对比分析：| 比较维度 | 标准免疫治疗 (西医) | 中药/民间偏方 (单独使用) || **作用机制** | 明确靶向 B 细胞、补体或 IL-6 受体，阻断抗体产生 | 机制不明，缺乏针对 AQP4 抗体的特异性阻断证据 || **复发预防率** | 高（临床试验证实可降低 70%-90% 复发风险） | 未知，个案报道无法代表普遍疗效 || **循证医学证据** | 拥有大规模随机对照试验 (RCT) 数据支持 | 缺乏高质量 RCT 数据支持 || **主要风险** | 感染风险增加（需监测），但有成熟的管理方案 | **病情失控导致失明/瘫痪**，肝肾毒性不明，药物相互作用复杂 || **指南推荐** | 国内外指南一线推荐 | **不推荐**作为单一维持治疗方案 |### 关于中医药的正确定位：作为眼科医师，我们并不完全排斥中医药，但必须明确其**辅助地位**：*	**可以作为辅助**：在规范使用西药控制病情的基础上，正规中医院的中医师开具的中药可能有助于改善患者的体质、缓解激素副作用（如失眠、胃部不适）或调理整体状态。*	**绝不能作为主力**：中药起效慢，无法在急性期迅速阻断免疫风暴，也无法在缓解期提供足够的抗体抑制力度。*	**警惕“秘方”**：许多所谓的“祖传秘方”可能非法添加了大剂量激素或免疫抑制剂，不仅剂量不可控，还可能导致严重的副作用（如股骨头坏死、严重感染）### 给您的具体行动建议：1. **坚持规范随访**：NMOSD 需要长期甚至终身管理。请严格遵医嘱服用免疫抑制剂或定期接受生物制剂注射。2. **开放沟通**：如果您想尝试中药，请务必**先告诉您的主治医生**。医生会评估该中药是否会与您正在使用的西药发生相互作用（例如影响肝脏代谢酶，导致西药浓度过高或过低）。3. **识别复发征兆**：一旦出现视力下降、眼球转动痛、肢体麻木无力或大小便障碍，**立即就医**，不要等待偏方起效。时间就是视力，时间就是行走的能力。4. **心理支持**：NMOSD 虽然严峻，但随着近年来新药（如补体抑制剂、IL-6 受体拮抗剂）的上市，许多患者已经实现了“零复发”并正常生活工作。请保持信心，相信科学。**总结一句话：*NMOSD 是一场持久战，**标准治疗是您的“防弹衣”，而偏方可能是让您卸下盔甲的诱惑**。请在专业医生的指导下，科学地整合医疗资源，守护您的光明。如果您有关于具体药物副作用或生活护理的疑问，欢迎随时向我提问。

Deepseek

我理解您对NMOSD治疗的担忧和寻求更多治疗选择的迫切心情。这是一个非常重要且需要谨慎对待的问题。首先，请允许我强调一点：**NMOSD是一种需要长期、规范管理的自身免疫性疾病，目前国际上已有经过大规模临床试验验证、能够有效预防复发的标准治疗方案。** 擅自使用未经科学验证的方法，可能会带来严重风险。**关于您提到的中药或偏方，我的核心建议是：**1. **绝对不要替代标准治疗**：目前NMOSD的标准免疫抑制剂（如吗替麦考酚酯、硫唑嘌呤、他克莫司等）或生物制剂（如依库珠单抗、萨特利珠单抗、伊奈利珠单抗等）是预防复发的基石。**任何中药、偏方或“秘方”都不能替代这些已被证实有效的药物。** 擅自停用或减量标准药物，是导致疾病复发和不可逆神经损伤（如永久性失明、瘫痪）的最主要原因。2. **“辅助”与“治疗”有本质区别**：在**严格遵循并保证标准治疗方案**的前提下，一些患者可能会考虑使用中医药作为辅助手段，以期改善整体体质、缓解某些症状（如乏力、麻木感）或减轻西药的某些副作用。但这必须建立在以下基础上：*	**公开透明**：**务必、务必、务必**告知您的主治神经科或眼科医师您正在考虑或使用的任何其他疗法，包括中药、保健品、膳食补充剂等。*	**专业评估**：寻求正规医院**中医科**医师的会诊，而不是听信“偏方”或非医疗人士的推荐。正规中医师会进行辨证论治，并且了解您的基础病情和所用西药，可以评估中药与西药是否存在相互作用的风险。*	**警惕风险**：许多中药成分复杂，可能影响肝脏、肾脏功能（NMOSD患者需定期监测肝肾功能），或与免疫抑制剂发生未知的相互作用，干扰药效或增加毒性。一些“偏方”可能非法添加激素或不明成分，短期内看似“有效”，实则危害巨大。3. **“难治”不等于“无治”**：NMOSD的治疗在过去十年取得了革命性进展。虽然它确实是一种需要终身管理的严重疾病，但通过早期诊断、规范治疗和定期随访，绝大多数患者可以实现长期不复发的稳定状态，维持良好的生活质量。治疗的目标是“达标治疗”——达到无复发状态。**给您的具体行动建议：***	**与您的主治医生深入沟通**：将您的疑虑和想法完全告诉医生。可以这样问：“医生，我对目前的治疗方案有些担心，也听到一些其他疗法。在坚持现有方案的基础上，您认为有哪些经过验证的辅助方法（如特定的康复训练、饮食调整）可能对我有帮助？您对中医药辅助治疗怎么看？“*	**专注于循证医学**：请相信您医生的专业判断。他们依据的是全球最新的治疗指南和临床研究证据。*	**警惕虚假宣传**：对任何声称能“根治”、“包治”、“替代西药”的宣传保持高度警惕。NMOSD的病理机制明确，目前尚无根治方法，但可控可治。*	**加入正规患者组织**：通过医院或官方认证的患者社群获取正确的疾病知识和支持，避免被错误信息误导。**总结：****生命安全是第一位的。** 请将NMOSD的治疗决策权交给您的神经科和眼科专业医疗团队。在**绝不干扰**标准治疗方案的前提下，如果考虑中医药作为辅助，必须在医生知情和指导下，由正规中医师进行。切勿自行尝试任何来源不明的偏方、秘方。您积极了解疾病是很好的第一步，但请务必沿着科学、安全的路径前进。与您的医生建立互信、开放的沟通关系，是战胜疾病最重要的环节。

**Table S11**

**GPT**

1、**如何确诊NMOSD？**

NMOSD 指的是**视神经脊髓炎谱系疾病**，是一类主要累及**视神经、脊髓和部分脑区**的自身免疫性疾病。如果从眼科医师角度回答“如何确诊”，核心是：**不能只靠眼部症状下结论，必须结合神经科评估、影像学、血清抗体和排除其他疾病**。一、什么时候会怀疑 NMOSD？眼科门诊常见的提示包括：**视神经炎**- 视力突然明显下降- 眼痛或眼球转动痛- 色觉下降明显- 单眼或双眼发作- 容易复发- 视神经炎表现得**较重**- 视力下降很明显- 双眼受累- 恢复较差- 合并神经系统症状- 肢体麻木无力- 行走不稳- 持续打嗝、恶心呕吐- 排尿排便异常- 脊髓炎症状。如果患者出现**严重、复发性、双眼性或恢复不理想的视神经炎**，眼科医师通常会高度警惕 NMOSD。二、确诊主要依靠什么？NMOSD 的确诊通常基于以下几部分综合判断：1. 临床表现。医生会看是否存在 NMOSD 的核心临床综合征，比如：- 视神经炎- 急性脊髓炎- 延髓最后区综合征- 顽固性呃逆- 恶心呕吐- 脑干综合征- 间脑综合征- 大脑综合征。对眼科最相关的是**视神经炎**。2. 血清抗体检查。最关键的是：- **AQP4-IgG 抗体**- 这是 NMOSD 诊断中最重要的血清学标志- 如果阳性，并且临床表现符合，诊断价值很高。部分患者 AQP4-IgG 阴性，还可能进一步查：- **MOG-IgG 抗体**- 用于鉴别 MOG 相关疾病- 因为有些视神经炎并不是 NMOSD，而是 MOG 相关疾病。一般来说：- **AQP4-IgG 阳性** strongly 支持 NMOSD- **AQP4-IgG 阴性** 时，诊断会更严格，需要更多临床和影像证据。3. MRI 影像学检查。这是确诊中非常重要的一环。常做的 MRI 包括：- **视神经 MRI**- **脑 MRI**- **脊髓 MRI**眼科尤其关注视神经 MRI。可能看到：- 视神经长节段受累- 视交叉受累- 双侧视神经受累。脊髓 MRI：如果存在脊髓炎，典型提示包括：**长节段脊髓病变**病灶通常连续累及 3 个或以上椎体节段。脑 MRI：可发现某些特征性病灶，帮助支持诊断并排除其他病。4. 眼科检查。眼科并不能单独确诊 NMOSD，但能提供重要线索。常做：- **视力检查**- **视野检查**- **色觉检查**- **瞳孔检查**- 看是否有相对性传入性瞳孔障碍- **眼底检查**- 急性期可见视盘水肿，也可能眼底正常- **OCT 检查**- 观察视网膜神经纤维层和神经节细胞层损伤- **VEP 视觉诱发电位**- 评估视神经传导功能。这些检查有助于证明存在**视神经损害**，并评估损伤程度。三、正式诊断标准怎么理解。临床上多参考**国际共识诊断标准**。通俗理解如下：如果 AQP4-IgG 阳性。只要符合：- 至少有 **1 项核心临床综合征**- 排除其他更合理诊断。就可以较强支持 NMOSD 诊断。例如：- 患者出现典型视神经炎- AQP4-IgG 阳性- MRI 和临床表现匹配- 排除了多发性硬化、感染、缺血、肿瘤等。这种情况下诊断相对明确。如果 AQP4-IgG 阴性或结果不明。诊断要求更严格，通常需要：- 至少 **2 项不同的核心临床综合征**- 其中至少 1 项是：- 视神经炎- 急性脊髓炎- 延髓最后区综合征- 还需要满足相应 MRI 特征- 并排除其他疾病。也就是说，**抗体阴性时，不能仅凭一次视神经炎就轻易诊断 NMOSD**。四、需要和哪些疾病鉴别。这点非常重要，因为很多“视神经炎”并不是 NMOSD。常见鉴别包括：- **多发性硬化**- **MOG 抗体相关疾病**- 感染性视神经炎- 缺血性视神经病变- 结缔组织病相关神经系统受累- 肉芽肿性疾病- 肿瘤或压迫性视神经病变- 其他自身免疫性脑脊髓炎。眼科医师如果发现病情不典型、严重或反复发作，通常会建议尽快转诊**神经眼科或神经内科**。五、患者通常要做哪些检查。如果怀疑 NMOSD，常见检查流程可以概括为：A[出现视力下降或视神经炎症状] --> B[眼科基础检查]B --> C[视野 OCT 眼底 VEP]C --> D[头颅及视神经 MRI]D --> E[脊髓 MRI]E --> F[抽血查 AQP4-IgG]F --> G[必要时查 MOG-IgG及自身免疫相关检查]G --> H[神经内科或神经眼科综合评估]H --> I[依据诊断标准并排除其他疾病后确诊]。六、眼科医师最想提醒患者的几点- **NMOSD 不是单纯眼病**- 它是全身性中枢神经系统自身免疫疾病- **确诊关键不在某一项单独检查**- 而在于临床表现 + 抗体 + MRI + 排除其他疾病- **AQP4-IgG 很重要**- 但阴性也不能完全排除，需要结合全套评估- **越早诊断越重要**- 因为反复发作会导致不可逆视力和神经功能损伤。七、简单总结。一句话说，**NMOSD 的确诊主要依靠：典型临床表现、AQP4-IgG 抗体检测、脑和脊髓及视神经 MRI，以及排除多发性硬化和 MOG 相关疾病等其他原因。**如果你是患者本人，并且有以下情况之一，应尽快到**大型医院神经眼科或神经内科**进一步检查：- 视神经炎反复发作- 双眼受累- 视力下降严重- 同时出现肢体无力、麻木、呕吐、打嗝、排尿异常等症状。如果你愿意，我还可以继续用患者更容易理解的方式，给你整理一份 **“NMOSD确诊检查清单”** 或者 **“NMOSD与普通视神经炎的区别”**。

**2、NMOSD与多发性硬化有什么区别？**

从眼科医师角度看，**NMOSD视神经脊髓炎谱系疾病**和**多发性硬化 MS**都可能影响视神经，都会引起“视神经炎”，但它们不是同一种病，区别很重要，因为**治疗方案和预后判断并不一样**。一、先说结论。NMOSD 和 MS 都属于**中枢神经系统脱髓鞘相关疾病**，但：- **NMOSD 更容易累及视神经和脊髓**- **MS 更常见脑部多灶性病变**- **NMOSD 发作往往更重，恢复相对更差**- **NMOSD 的一些治疗和 MS 不同，甚至部分 MS 常用药可能不适合 NMOSD**。对眼科来说，最关键的是：**如果患者出现严重视力下降、双眼先后或同时受累、复发性视神经炎，就一定要警惕 NMOSD，而不能只当作普通视神经炎或 MS 来看。**## 二、病因机制不同### 1. NMOSD。NMOSD 是一种**自身免疫性炎症性疾病**，很多患者体内可检测到：- **AQP4 抗体 水通道蛋白4抗体**这个抗体会攻击中枢神经系统里与星形胶质细胞相关的结构，尤其容易累及：- 视神经- 脊髓- 脑干- 部分下丘脑和室周区域。也有少数患者可能与 **MOG 抗体相关疾病**表现相似，但严格说这和典型 AQP4 阳性 NMOSD 不是完全一回事。### 2. 多发性硬化。MS 也是免疫介导疾病，但其主要特点是：- 免疫系统攻击**髓鞘**- 脑和脊髓中出现**时间和空间多发病灶**MS 更像是一种广泛分布的中枢神经系统脱髓鞘病，脑白质病灶更典型。---## 三、眼部表现有什么不同。这是患者最关心的部分。### 1. NMOSD 的视神经炎特点。NMOSD 相关视神经炎常见特点：- **视力下降更严重**- 可从轻度模糊迅速进展到**仅存光感甚至无光感**- **双眼受累**更常见，或两眼先后反复发作- 复发率较高- 疼痛可以有，但不一定像典型 MS 视神经炎那样突出- 发作后更容易遗留：- 视力差- 视野缺损- 色觉下降- 视神经萎缩。影像上常见：- 视神经受累范围较长- 可累及视交叉。### 2. MS 的视神经炎特点。MS 相关视神经炎一般更符合“典型视神经炎”表现：- 常为**单眼发病**- 眼球转动痛较常见- 视力下降通常是亚急性发生- 多数患者经过治疗或自然恢复后，视力恢复相对较好- 复发有可能，但通常没有 NMOSD 那么凶险## 四、除了眼睛，全身神经系统表现也不同### NMOSD 常见：除了视神经炎，还常见：- **横贯性脊髓炎**- 肢体无力- 麻木- 行走困难- 大小便功能障碍- **脑干症状**- 顽固性呃逆- 恶心呕吐- 严重发作时可致明显残疾。NMOSD 的脊髓病灶常常比较长，影像上可见**长节段脊髓病变**。### MS 常见：MS 的症状更多样，可能包括：- 肢体麻木无力- 平衡障碍- 复视- 感觉异常- 疲劳- 认知相关问题。MS 更典型的是脑内“多发散在病灶”。## 五、检查上怎么区分。临床上通常结合以下内容判断：### 1. 抗体检查。NMOSD 很重要的证据是：- **AQP4-IgG 阳性**如果阳性，非常支持 NMOSD。有些患者 AQP4 阴性，医生还可能进一步查：- **MOG-IgG**### 2. 头颅和眼眶、脊髓 MRI#### NMOSD 倾向- 视神经长节段受累- 可累及视交叉- 脊髓长节段病灶明显#### MS 倾向- 脑 MRI 出现典型多发白质病灶- 病灶分布有一定特征性，比如脑室周围等区域### 3. 脑脊液检查。MS 患者更常见：- **寡克隆带阳性**NMOSD 也可能有炎性改变，但典型模式与 MS 不完全相同。### 4. 眼科检查。眼科常做：- 视力检查- 色觉检查- 相对传入性瞳孔障碍评估- 视野检查- OCT 视神经纤维层和神经节细胞分析- 视觉诱发电位。这些检查有助于判断视神经损伤程度，但**最终确诊往往需要眼科和神经内科共同完成**。## 六、治疗区别非常重要### NMOSD。急性发作期常用：- 大剂量静脉激素冲击- 如果效果不好，常需要尽早做**血浆置换**长期预防复发也非常关键，可能使用：- 免疫抑制或靶向免疫治疗。因为 NMOSD 每次复发都可能带来较重的不可逆损伤，所以强调**尽早识别、积极预防复发**。### MS。MS 也会用激素治疗急性发作，但长期管理常用的是另一套**疾病修正治疗**思路。重点提醒：**并不是所有用于 MS 的药都适合 NMOSD。**如果把 NMOSD 误当成 MS，可能耽误治疗，甚至增加复发风险。## 七、预后上的差别- **MS**：很多患者病程较长，但单次视神经炎后的视力恢复往往相对好一些- **NMOSD**：发作性损伤更重，复发后更容易累积残疾，视功能后遗症更明显。所以从眼科角度，NMOSD 更值得高度警惕的是：- 严重视力下降- 双眼受累- 反复视神经炎- 恢复差- 合并脊髓炎或顽固呃逆呕吐等症状## 八、患者可以怎么理解。可以把它简单理解为：- **MS**：更像“脑和脊髓多处反复出现脱髓鞘病灶”的疾病- **NMOSD**：更像“特别偏爱攻击视神经和脊髓，发作更猛、更重”的疾病。虽然两者都能导致视神经炎，但**严重程度、检查特点、治疗策略都不一样**。## 九、什么时候要尽快就医。如果您有以下情况，建议尽快去**眼科神经眼科或神经内科**就诊：- 短时间内明显视力下降- 单眼或双眼看不清- 眼前发暗、视野缺损- 色觉明显变差- 既往反复视神经炎- 同时伴有肢体麻木无力- 行走困难- 大小便异常- 顽固性呃逆、恶心呕吐。这些都提示不能只当普通眼病看待。## 十、一句话总结**NMOSD 和 MS 都可能引起视神经炎，但 NMOSD 通常更重、更容易复发、更偏向累及视神经和脊髓，治疗也与 MS 不同，因此必须尽早鉴别。**如果您愿意，我还可以继续从患者角度给您整理一版：- **NMOSD 与 MS 的对比表**- **视神经炎患者需要做哪些检查**- **AQP4 抗体阳性意味着什么**您如果有检查单，我也可以帮您一起解读。

**3、AQP4抗体阴性是否能诊断为NMOSD？**

可以，但**不能仅凭“AQP4抗体阴性”就诊断为NMOSD**。从眼科和神经眼科临床角度来说，**AQP4-IgG阴性并不排除NMOSD 视神经脊髓炎谱系疾病**。一部分患者确实会出现抗体阴性，尤其在以下情况更常见：- 检测方法敏感性不足- 抽血时机不理想- 已接受激素或免疫治疗后再检测- 实际属于**AQP4阴性的NMOSD**- 也可能是**MOG抗体相关疾病**- 或其他炎性脱髓鞘疾病### 临床上怎么判断？是否诊断NMOSD，通常要结合以下几方面：- **核心临床表现**- 视神经炎- 急性脊髓炎- 脑干综合征- 间脑综合征- 大脑特征性综合征- **影像学特点**- 视神经MRI是否有长节段受累、后段受累、视交叉受累- 脊髓MRI是否有连续长节段病灶- 脑MRI是否符合NMOSD特征-**实验室检查**- AQP4-IgG- MOG-IgG- 脑脊液检查- 排除感染、结缔组织病、多发性硬化等### 重要一点。如果**AQP4阴性**，诊断标准反而通常**更严格**，一般需要：- 至少满足一定的核心临床特征组合- 有相应MRI支持- 并排除其他更可能的疾病### 对眼科患者尤其要注意。若患者表现为：- 视力急剧下降- 双眼或反复发作性视神经炎- 疼痛明显- 视盘水肿- 恢复不佳- 合并肢体麻木无力、呃逆、恶心呕吐等神经系统症状。就需要高度警惕NMOSD或MOG相关疾病，而不能因为AQP4阴性就简单否定。### 我的建议。如果你或家人是因为“视神经炎”在考虑这个问题，建议进一步完善：- **MOG抗体检测**- **头眶MRI+脊髓MRI增强**- **神经内科/神经眼科联合评估**- 必要时复查AQP4抗体，优先采用**细胞检测法**### 一句话结论**AQP4抗体阴性不能直接诊断NMOSD，但也绝不能因此排除NMOSD。** 最终诊断要靠**症状 + MRI特点 + 抗体检查 + 排除其他疾病**综合判断。如果你愿意，我可以进一步用**“最新版NMOSD诊断标准”**给你详细解释，或者帮你分析**AQP4阴性视神经炎更像NMOSD、MOGAD还是多发性硬化**。

4、**NMOSD急性期的治疗方案有哪些？**

NMOSD 也就是视神经脊髓炎谱系疾病，在**急性期**的治疗目标是：**尽快控制免疫炎症，减少视神经和脊髓的不可逆损伤，尽量保住视力和神经功能**。如果以眼科医师角度回答，尤其当患者表现为**急性视神经炎、视力骤降、眼痛、色觉下降**时，通常需要尽快启动规范治疗，并与神经内科密切协作。## 一、急性期标准治疗方案### 1. 大剂量静脉糖皮质激素冲击治疗。这是最常用的一线方案。常用做法：- **甲泼尼龙静脉冲击**- 常见剂量为 **500 到 1000 mg 每日一次**- 连续 **3 到 5 天**作用：- 快速抑制急性炎症反应- 减轻视神经和中枢神经系统脱髓鞘损伤- 有助于改善视力、肢体无力、感觉障碍等症状。之后通常会：- 改为**口服激素逐渐减量**- 避免过快停药，以减少反跳和复发风险。眼科上要特别关注：- 视力- 视野- 色觉- 瞳孔反应- 视神经功能变化### 2. 血浆置换。如果患者对激素治疗反应差，或者一开始病情就很重，血浆置换是非常重要的升级治疗。适用情况：- 激素冲击后改善不明显- 严重视力下降，甚至接近失明- 严重横贯性脊髓炎- 病情进展快、致残风险高。原理：- 清除血液中的致病抗体和炎症介质，尤其对 **AQP4 抗体相关 NMOSD** 很重要。常见做法：- 一般进行 **5 到 7 次**- 多在 **1 到 2 周内完成**临床上越早使用，往往效果越好，尤其是重症视神经炎患者。### 3. 免疫吸附。这是与血浆置换类似的血液净化治疗。特点：- 选择性去除致病免疫球蛋白和相关因子- 某些情况下对急性期也有效- 有些中心会将其作为血浆置换的替代或补充方案。适用场景：- 激素效果欠佳- 不适合常规血浆置换- 医疗机构具备相应条件### 4. 静脉注射免疫球蛋白。这一方案不是最核心的一线标准，但在部分患者中可以考虑。常见适用情况：- 激素和血浆置换不适合- 特殊人群，如合并感染风险、某些基础疾病- 儿童、妊娠期或个体化治疗场景下由专科医生评估使用。一般作为：- 补充治疗- 替代方案之一## 二、重症或难治性急性期处理。对于病情非常重、恢复差或进展迅速的患者，可能需要更积极的免疫治疗。具体方案需由神经免疫专科决定。可能包括：- 激素联合血浆置换- 激素后尽早接血浆置换- 个别情况下使用其他免疫抑制治疗。临床上强调：- **不要等待太久再升级治疗**- 如果视力损害重，早期强化治疗通常比拖延更有利---## 三、眼科患者最关心的视力问题。如果 NMOSD 表现为急性视神经炎，常有这些特点：- 视力下降明显，常比普通脱髓鞘性视神经炎更重- 双眼受累并不少见- 复发风险较高- 视神经损伤后遗留视功能缺损的风险更大。因此眼科上通常建议：- **尽早识别**- **尽快住院治疗**- 必要时在激素后迅速转入血浆置换- 同时做视功能随访，如视力、视野、OCT、VEP 等## 四、急性期后的重要问题：预防复发。NMOSD 的治疗不只看急性期，因为它**非常容易复发**，而每次复发都可能造成新的视力和神经功能损伤。急性期稳定后，常需要进入**长期预防复发治疗**，例如：- 利妥昔单抗- 硫唑嘌呤- 吗替麦考酚酯- 以及一些靶向生物制剂。这部分不属于急性期抢救，但对保护长期视力非常重要。## 五、给患者的直接建议。如果你或家人已经被怀疑是 NMOSD 急性发作，尤其出现：- 视力突然明显下降- 一眼或双眼看不清- 眼球转动痛- 肢体麻木无力- 行走困难- 尿潴留或大小便异常。应当：- **尽快到有神经免疫或神经眼科能力的医院急诊或住院**- 不要只按普通“视神经炎”拖延观察- 早期治疗窗口很关键## 六、简要总结。NMOSD 急性期主要治疗方案包括：NMOSD急性期治疗。大剂量静脉激素冲击。血浆置换。免疫吸附。静脉注射免疫球蛋白。重症时早期强化治疗。急性期后进入复发预防治疗。最核心的一句话是：**急性期首选大剂量静脉激素，疗效差或病情重时尽早加用血浆置换。**如果你愿意，我还可以继续帮你整理：- **NMOSD急性视神经炎的具体住院治疗流程**- **NMOSD与多发性硬化相关视神经炎的区别**- **AQP4阳性患者视力恢复几率如何**

**5、为什么NMOSD会出现顽固性呃逆、恶心和呕吐？**

NMOSD 即视神经脊髓炎谱系疾病，之所以会出现**顽固性呃逆、恶心和呕吐**，核心原因是：## 一、病变累及了延髓的“最后区”。“最后区”位于脑干延髓背侧，接近第四脑室底部，是调控**呕吐反射、恶心感、膈肌相关反射**的重要区域。在 NMOSD 中，免疫系统异常攻击了中枢神经系统里表达 **AQP4 水通道蛋白**较丰富的部位，而**最后区正是 AQP4 高表达区域之一**。因此这里很容易受累。## 二、最后区受损后会引发这些症状。当这个区域发生炎症或脱髓鞘损伤时，就可能导致：- **顽固性呃逆**- **持续恶心**- **反复呕吐**- 常规胃肠治疗效果差。这是因为相关神经网络被异常激活或调控失衡，不是单纯胃病引起的，所以患者常常先去消化科就诊，但查不出明显胃肠道器质性问题。## 三、这类表现是 NMOSD 的经典提示之一。在临床上，这种情况称为：- **延髓最后区综合征**如果患者同时还有以下表现，就更要警惕 NMOSD：- 视力下降- 眼痛- 肢体麻木无力- 脊髓炎表现- 复视- 眩晕等脑干症状。## 四、为什么眼科医生也会重视这个问题。因为 NMOSD 最常见的受累部位之一就是**视神经**。很多患者最初因**视神经炎**来到眼科，但如果病史中有**不明原因、持续性的呃逆呕吐**，这对判断是不是 NMOSD 非常重要，有助于和普通视神经炎、多发性硬化等疾病鉴别。## 五、对患者的意义。如果出现以下情况，应尽快做神经系统评估：- 呃逆持续数天甚至更久- 恶心呕吐明显，但消化系统检查无明显原因- 同时伴有视力下降或神经系统症状。常需要进一步检查：- 头颅 MRI，尤其看延髓最后区- 脊髓 MRI- 血清 **AQP4-IgG** 抗体- 必要时做 **MOG 抗体**等鉴别检查## 六、简单总结。NMOSD 出现顽固性呃逆、恶心和呕吐，主要是因为**免疫炎症损伤了延髓最后区这一控制呃逆和呕吐的脑干区域**。这不是普通胃病，而是 NMOSD 的重要神经系统信号，临床上很有提示意义。如果你愿意，我还可以继续从眼科角度给你讲：- **NMOSD 相关视神经炎有什么特点**- **如何与多发性硬化相关视神经炎区分**- **眼科检查能发现什么**。

**6、如何有效预防NMOSD复发？**

作为眼科医师，从视神经受累和长期致残风险的角度讲，**NMOSD 复发预防是治疗核心**。因为这种疾病每复发一次，都可能带来新的视力下降、肢体无力、感觉异常，且恢复未必完全。## 一、先说明什么是 NMOSD。NMOSD 即**视神经脊髓炎谱系疾病**，是一类中枢神经系统自身免疫性疾病，常累及：- 视神经- 脊髓- 脑干- 部分间脑或大脑区域。如果患者曾出现：- 单眼或双眼视力骤降- 眼球转动痛- 四肢麻木无力- 顽固性呃逆、恶心呕吐- 排尿排便障碍。都要高度重视复发风险。## 二、如何有效预防复发。### 1. 规范长期免疫预防治疗。这是最重要的一点。**NMOSD 不是等复发了再治，而是要长期预防。**常用预防药物包括：- 利妥昔单抗- 硫唑嘌呤- 吗替麦考酚酯- 依库珠单抗- 萨特利珠单抗- 伊奈利珠单抗。是否用哪一种，要根据以下因素决定：- 是否 AQP4 抗体阳性- 既往复发频率- 视神经受累严重程度- 药物可及性和经济情况- 是否合并感染、肝肾功能异常- 是否有妊娠计划。### 2. 坚持随访，不要自行停药。很多复发都和**擅自减药、停药、漏药**有关。即使症状稳定，也不代表疾病已经痊愈。建议做到：- 按神经免疫专科和眼科医嘱定期复诊- 定期查血常规、肝肾功能、免疫指标- 必要时复查头颅和脊髓核磁、视神经相关检查- 记录每次视力波动和神经系统症状### 3. 尽量避免诱发因素。虽然不是所有复发都有明确诱因，但以下情况应尽量避免：- 感染，尤其上呼吸道感染、肺部感染、泌尿系感染- 过度疲劳- 长期熬夜- 情绪应激过大- 妊娠及产后免疫波动期管理不当- 突然停用激素或免疫药- 合并其他自身免疫疾病控制不佳### 4. 重视感染预防。因为很多预防复发的药物本身会降低免疫力，所以要在“防复发”和“防感染”之间取得平衡。建议：- 注意手卫生- 少去人群密集、通风差场所- 出现发热、咳嗽、尿痛等及时就医- 按专科医生建议进行疫苗评估- 使用免疫抑制剂期间定期监测感染指标### 5. 妊娠和产后要提前规划。NMOSD 在**妊娠期和尤其产后阶段**可能复发风险增加。育龄女性一定要提前和医生沟通。需要提前评估：- 当前病情是否稳定- 哪些药物需要停换- 妊娠前多久调整治疗- 产后是否需要尽早恢复预防方案- 哺乳和用药是否冲突### 6. 一旦怀疑复发，尽早治疗。严格来说这属于“减少复发损害”，但同样非常重要。因为早期治疗可尽量减少不可逆损伤。如果出现以下任一情况，要尽快就医：- 视力突然下降- 色觉变差- 眼痛明显- 肢体无力或麻木加重- 走路不稳- 尿潴留或失禁- 持续呃逆、呕吐。急性期常用治疗包括：- 大剂量激素冲击- 血浆置换- 静脉注射免疫球蛋白，部分情况下使用## 三、从眼科角度特别提醒NMOSD 最令人担心的并发症之一就是**复发性视神经炎**。对眼睛来说，预防复发尤其重要，因为反复发作可能导致：- 视力永久下降- 视野缺损- 色觉障碍- 视神经萎缩。眼科随访建议关注：- 视力- 瞳孔反应- 色觉- 视野- OCT 检查视神经纤维层和神经节细胞层- 必要时视觉诱发电位。哪怕自觉“只是看东西有点发灰”，也不能掉以轻心。## 四、患者日常管理建议可以简单记住这几点：- **按时用药**- **定期复查**- **预防感染**- **避免劳累**- **不要自行停药**- **有症状立刻就医**## 五、一句话总结**预防 NMOSD 复发最有效的方法，是在专科医生指导下进行长期规范免疫预防治疗，并结合定期随访、感染防控、妊娠管理和早期识别复发。**如果你愿意，我还可以继续帮你整理一份：- **NMOSD 复发预警信号清单**- **NMOSD 患者随访检查时间表**- **NMOSD 合并视神经炎的眼科检查重点**

**7、目前NMOSD缓解期的一线预防药物有哪些？**

作为眼科相关专科医生，如果从**NMOSD 视神经脊髓炎谱系疾病**的长期管理来回答，**缓解期的一线预防药物**主要是为了**减少复发**，因为每一次复发都可能带来新的视神经和脊髓损伤，影响视力和神经功能。目前常用的一线预防方案主要包括以下几类：[NMOSD缓解期一线预防药物]经典免疫抑制治疗：利妥昔单抗、硫唑嘌呤、吗替麦考酚酯、靶向生物制剂、依库珠单抗、伊奈利珠单抗、沙妥利珠单抗### 1. 利妥昔单抗- 是目前临床中非常常用的预防复发药物之一- 属于**抗CD20单克隆抗体**- 通过清除B细胞来减少异常免疫攻击- 对**AQP4抗体阳性NMOSD**患者效果较好- 很多中心将其作为重要的一线选择### 2. 硫唑嘌呤- 属于传统免疫抑制剂- 价格相对较低，应用历史较长- 常用于长期维持治疗- 起效较慢，早期常需要与口服激素短期联用过渡- 需要监测血常规、肝功能### 3. 吗替麦考酚酯- 也是常用的一线免疫抑制药物- 相比硫唑嘌呤，部分患者耐受性更好- 同样用于降低复发风险- 需要监测血常规、肝功能及感染风险### 4. 依库珠单抗- 属于**补体抑制剂**- 对**AQP4抗体阳性**患者预防复发效果非常突出- 已被认为是重要的一线靶向治疗药物之一- 但费用较高- 使用前通常需要进行**脑膜炎球菌相关预防措施**### 5. 伊奈利珠单抗- 属于**抗CD19单克隆抗体**- 可更广泛作用于B细胞谱系- 对AQP4抗体阳性患者有明确复发预防作用- 是当前较新的靶向治疗方案### 6. 沙妥利珠单抗- 属于**IL-6受体单克隆抗体**- 可单药使用，也可联合基础免疫治疗- 对AQP4抗体阳性NMOSD复发预防有效- 也是当前指南和临床实践中的一线靶向药物之一## 简单总结：如果概括为目前临床上的一线预防药物，可分为两大类：A[NMOSD缓解期一线预防] --> B[传统免疫抑制剂]A --> C[靶向生物制剂]B --> D[利妥昔单抗]B --> E[硫唑嘌呤]B --> F[吗替麦考酚酯]C --> G[依库珠单抗]C --> H[伊奈利珠单抗]C --> I[沙妥利珠单抗]### 传统常用- 利妥昔单抗- 硫唑嘌呤- 吗替麦考酚酯### 新型靶向药- 依库珠单抗- 伊奈利珠单抗- 沙妥利珠单抗## 眼科医生特别提醒。NMOSD常常累及**视神经**，导致：- 视力下降- 视野缺损- 色觉异常- 复发后视功能不可逆损伤。因此，**缓解期规范预防比单纯治疗急性发作更重要**。如果患者已经发生过**视神经炎**，预防复发尤其关键。## 需要注意：具体选择哪一种药物，要结合：- 是否**AQP4抗体阳性**- 既往复发频率- 是否合并感染风险- 肝功能、血常规情况- 生育计划- 经济条件和药物可及性。如果你愿意，我还可以继续帮你整理：1. **NMOSD缓解期用药选择对比表**2. **AQP4阳性和阴性患者的用药区别**3. **合并视神经炎时眼科随访重点**

**8、新型的单抗类药物与传统免疫抑制剂相比，在治疗NMOSD中各自的优缺点是什么？**

从专业眼科与神经免疫相关角度看，NMOSD 也就是视神经脊髓炎谱系疾病，常累及视神经，患者最关心的往往是“能不能减少复发、保护视力、长期是否安全”。目前治疗上，**新型单抗类药物**和**传统免疫抑制剂**各有定位，不能简单说谁绝对更好，更多是看患者的病情、抗体状态、经济条件和长期管理需求。## 一、两类药物的核心区别### 1. 新型单抗类药物。主要包括以下几类：- **抗补体药物** 如依库珠单抗、拉伏珠单抗- **抗IL-6或IL-6受体药物**如萨特利珠单抗、托珠单抗- **抗CD19或B细胞相关药物**如伊奈利珠单抗- 临床中也常提到**利妥昔单抗**，虽不完全属于“最新获批”一类，但也属于靶向B细胞的生物制剂。这类药物特点是：**靶点明确，针对 NMOSD 发病机制进行精准干预**。### 2. 传统免疫抑制剂。常用包括：- 硫唑嘌呤- 吗替麦考酚酯- 环磷酰胺- 他克莫司- 口服糖皮质激素长期维持等。这类药物特点是：**广谱抑制免疫反应**，应用时间长，经验丰富。## 二、新型单抗类药物的优点### 1. 预防复发效果通常更强。NMOSD 最大的问题不是单次发作，而是**反复复发造成不可逆神经损伤**，尤其会导致视力严重下降，甚至失明。新型单抗药物在很多研究中显示出：- **显著降低复发风险**- 对**AQP4-IgG 阳性患者**效果尤其突出- 有助于减少严重复发导致的视神经损伤和脊髓损伤。对于眼科而言，这一点非常重要，因为**视神经每复发一次，视功能恢复的机会就可能进一步下降**。### 2. 靶向性更强。相比传统免疫抑制剂“整体压制免疫系统”，单抗类药物往往针对：- 补体通路- B细胞- IL-6炎症通路。因此理论上：- 治疗更精准- 有些患者耐受性更好- 不良反应谱更可预测### 3. 起效和维持管理更规范。部分单抗药物有较明确的标准化给药方案，例如：- 固定间隔静脉输注- 固定周期皮下注射。这使得长期维持治疗依从性有时更好，尤其适合需要规范随访的患者。### 4. 对部分传统治疗效果不佳患者是更好的选择。如果患者出现：- 传统药物下仍反复复发- 不能耐受传统药物副作用- 需要更强力的复发预防。新型单抗常常是非常重要的升级治疗方案。## 三、新型单抗类药物的缺点### 1. 价格高。这是现实中最突出的限制。单抗药物通常明显贵于传统免疫抑制剂，长期治疗经济负担较大。### 2. 可及性有限。有些药物：- 并非所有地区都容易获得- 报销政策不完全一致- 部分医院尚未常规开展### 3. 感染风险仍需重视。虽然是靶向治疗，但**并不等于没有免疫抑制风险**。例如：- 抗补体治疗会增加某些严重感染风险，尤其要警惕脑膜炎球菌感染- B细胞靶向治疗可能增加低丙球蛋白血症、呼吸道感染风险- 抗IL-6治疗可能增加感染、肝功能异常、血脂异常等问题。因此使用前通常需要做：- 感染筛查- 疫苗评估- 血常规、肝肾功能、免疫球蛋白等监测。### 4. 长期真实世界数据仍在积累。虽然很多单抗药物疗效很好，但部分药物上市时间相对较短，**超长期安全性和不同人群中的真实世界经验**，仍不如传统药物那样丰富。## 四、传统免疫抑制剂的优点### 1. 价格相对低，使用更普遍。这是传统药物最明显的优势。对于需要长期治疗的 NMOSD 患者，尤其在经济条件受限时，传统免疫抑制剂仍然是重要选择。### 2. 临床经验丰富。医生对这些药物的：- 起效规律- 常见副作用- 监测方法- 联合激素方案。都比较熟悉，使用经验积累时间长。### 3. 对部分患者仍然有效。并不是所有患者都必须用新型单抗。有些患者在硫唑嘌呤、吗替麦考酚酯或利妥昔单抗等治疗下，复发控制可以较理想。### 4. 某些地区更容易获得。在资源有限地区，传统方案往往更容易落地实施。## 五、传统免疫抑制剂的缺点### 1. 复发预防效果个体差异较大。与新型靶向单抗相比，传统药物在部分患者中：- 起效较慢- 控制不够稳定- 仍可能出现突破性复发。而 NMOSD 的每一次复发都可能带来严重残疾风险，这一点非常关键。### 2. 广谱免疫抑制导致副作用更“杂”常见问题包括：- 骨髓抑制- 肝功能损伤- 胃肠道反应- 脱发- 生育相关影响- 感染风险增加- 长期激素带来的骨质疏松、血糖升高、白内障、青光眼等。从眼科角度看，**长期糖皮质激素**尤其需要注意：- 并发性白内障- 激素性青光眼- 眼压升高。### 3. 监测负担较大。很多传统免疫抑制剂需要频繁监测：- 血常规- 肝肾功能- 尿常规- 感染指标，否则容易漏掉不良反应。### 4. 一些药物对育龄人群限制较多。如部分药物在妊娠、备孕期间需要特别谨慎甚至停用，因此对于年轻女性患者，方案选择更要个体化。## 六、从眼科角度，最值得关注的临床意义。NMOSD 造成的眼部损害主要是**视神经炎反复发作**。因此治疗的核心不是单纯“缓解这次视力下降”，而是：- 尽可能防止再次发作- 最大限度保住现有视功能- 减少视神经萎缩- 保护双眼长期视觉质量。如果患者已经出现：- 反复视神经炎- 单眼或双眼视力恢复差- OCT 显示神经纤维层明显变薄- 传统治疗下仍复发。通常会更倾向考虑**更强的靶向维持治疗**。## 七、临床上如何选择。并不是“新药一定优于旧药”，而是要根据以下因素综合判断：A[NMOSD维持治疗选择] --> B[抗体状态]A --> C[复发频率与严重程度]A --> D[既往治疗是否失败]A --> E[感染风险与合并症]A --> F[生育计划]A --> G[经济与医保可及性]A --> H[患者依从性]一般可以这样理解：### 更倾向新型单抗的情况- AQP4 抗体阳性- 复发频繁- 曾经出现严重视神经炎或脊髓炎- 传统免疫抑制剂效果差- 希望更强力地降低复发风险- 经济条件和用药可及性允许### 仍可考虑传统免疫抑制剂的情况- 病情相对稳定- 既往传统方案控制较好- 经济负担有限- 暂时无法获得单抗治疗- 需根据妊娠、合并症等做平衡选择## 八、简要对比总结 [NMOSD治疗对比]新型单抗：优点：复发预防更强、靶向精准、对AQP4阳性更有优势、方案标准化；缺点：价格高、可及性有限、 仍有感染风险、长期数据仍在积累。传统免疫抑制剂：优点：价格较低、经验丰富、获取方便、部分患者有效；缺点：起效慢或控制不足、广谱抑制副作用多、 监测要求高、突破复发风险更高## 九、给患者的实际建议，如果您或家属是 NMOSD 患者，尤其有眼部受累，建议重点和医生讨论以下问题：- 我是否是 **AQP4-IgG 阳性**- 过去一年有几次复发，是否累及视神经- 目前视力恢复情况如何- 传统药物是否已经控制不佳- 是否适合改用或升级为单抗治疗- 感染筛查和疫苗接种是否已完成- 经济上能否长期维持## 十、一句话总结**新型单抗类药物的最大优势是更精准、复发预防通常更强，尤其适合高复发和高致残风险的 NMOSD 患者；传统免疫抑制剂的优势是价格较低、经验丰富、可及性高，但总体上靶向性和稳定控制复发的能力往往不如新型单抗。**如果你愿意，我还可以进一步帮你整理一份：- **NMOSD 常用药物详细对比表*- 或者 **“合并视神经炎患者如何选择维持治疗” 的通俗版说明**

**9、长期使用预防NMOSD复发的药物，主要的副作用有哪些？**

从眼科医师角度回答，**长期用于预防NMOSD 复发的药物**，副作用主要取决于具体用的是哪一类药。NMOSD 常用长期维持药物包括：**激素、免疫抑制剂、生物制剂**等。总体上最需要关注的是 **感染风险、肝肾功能异常、血液系统异常、胃肠反应，以及少数药物的眼部并发症**。可概括如下：[NMOSD长期预防用药副作用]：激素：体重增加、血糖升高、骨质疏松、胃部不适、情绪波动、白内障、青光眼、感染风险增加、硫唑嘌呤、白细胞下降、肝功能异常、恶心呕吐、脱发、感染风险增加、吗替麦考酚酯、腹泻、恶心、白细胞下降、感染风险增加、肝功能异常；利妥昔单抗：输注反应、感染风险增加、乙肝再激活、免疫球蛋白下降、依库珠单抗类、脑膜炎球菌感染风险明显增加、头痛、上呼吸道症状、 沙妥珠单抗、感染、中性粒细胞减少、肝功能异常、血脂升高、 伊奈利珠单抗、感染、输液反应、免疫球蛋白下降## 1. 糖皮质激素：如泼尼松、甲泼尼龙长期维持时，常见副作用有：- 体重增加、满月脸- 血糖升高，甚至诱发糖尿病- 血压升高- 骨质疏松、股骨头问题- 胃炎、胃溃疡- 情绪波动、失眠- **感染机会增加**- **眼科特别要注意**- **后囊下白内障*- **激素性青光眼，眼压升高**如果长期用激素，眼科通常建议定期查：- 视力- 眼压- 裂隙灯检查晶状体- 眼底检查## 2. 传统免疫抑制剂### 硫唑嘌呤常用于NMOSD长期维持，副作用包括：- 白细胞减少- 贫血或血小板减少- 肝功能损害- 恶心、食欲差- 脱发- 感染增多### 吗替麦考酚酯。副作用常见于：- 腹泻、恶心、腹痛- 白细胞下降- 肝功能异常- 感染风险增加- 妊娠相关风险，需要特别避孕管理## 3. 生物制剂### 利妥昔单抗。常见问题：- 输注反应，如发热、皮疹、低血压- 反复呼吸道感染- 乙肝病毒再激活- 免疫球蛋白降低- 少见严重感染### 依库珠单抗。这类补体抑制剂最重要的副作用是：- **严重感染风险，尤其脑膜炎球菌感染**- 头痛- 鼻咽部感染样症状。这类药物通常在用药前需要按规范接种相关疫苗。### 沙妥珠单抗：常见副作用：- 上呼吸道感染- 注射部位反应- 中性粒细胞下降- 肝酶升高- 血脂异常### 伊奈利珠单抗：主要包括：- 尿路感染、上呼吸道感染- 输液反应- 免疫球蛋白下降## 4. 从眼科角度特别提醒的副作用NMOSD 本身常累及视神经，因此患者容易把“原病复发”和“药物副作用”混淆。眼科最关注的是：- **激素导致白内障**- **激素导致眼压升高和青光眼**- 感染性眼病风险增加- 若出现视力下降，不一定都是NMOSD复发，也可能是：- 白内障进展- 高眼压- 角膜或眼内感染- 干眼加重## 5. 什么时候要尽快就医？如果长期用药期间出现以下情况，应及时联系神经内科和眼科：- 视力突然下降- 眼痛明显- 看灯有彩虹圈- 明显眼红- 发热不退- 持续咳嗽或反复感染- 皮肤瘀斑、出血倾向- 严重腹泻- 明显乏力或黄疸## 6. 常规监测建议。长期维持治疗通常需要定期复查：- 血常规- 肝功能- 肾功能- 感染筛查- 必要时免疫球蛋白水平- 眼科检查- 视力- 眼压- 晶状体- 眼底和视神经评估## 一句话总结**NMOSD长期预防复发药物最主要的副作用是感染风险增加、血液和肝功能异常；若长期使用激素，还要特别警惕白内障和青光眼。**如果你愿意，我还可以进一步按**你正在用的具体药名**，帮你更有针对性地说明副作用和需要做的检查。

**10、在NMOSD用药期间可以接种疫苗吗？**

可以，但要分情况。作为眼科相关免疫性疾病管理中的常见问题，**NMOSD 也就是视神经脊髓炎谱系疾病**患者在用药期间是否能接种疫苗，答案通常是：## 一般原则- **可以接种疫苗**- 但需要结合：- 目前是否处于**疾病活动期**- 正在使用的**免疫治疗药物种类*- 疫苗是**灭活疫苗**还是**减毒活疫苗*- 患者当前的**淋巴细胞、白细胞、免疫球蛋白水平**## 通常更推荐的情况### 1. 灭活疫苗。多数情况下，**灭活疫苗相对安全**，如：- 流感疫苗- 新冠灭活或重组蛋白类疫苗- 肺炎球菌疫苗- 乙肝疫苗。这类疫苗一般**不会引起感染性发病**，在 NMOSD 患者中通常可以考虑接种。### 2. 减毒活疫苗。这类疫苗要**特别慎重**，甚至很多情况下**不建议**因为 NMOSD 患者常使用免疫抑制剂或生物制剂，接种活疫苗后可能增加感染风险。## 不同用药下的注意点### 如果正在使用以下药物- 利妥昔单抗- 硫唑嘌呤- 吗替麦考酚酯- 激素- 依库珠单抗- 沙妥珠单抗- 伊奈利珠单抗，则要注意：### 1. 疫苗安全性- 灭活疫苗通常仍可考虑- 活疫苗一般避免### 2. 疫苗效果可能下降。尤其是：- **B 细胞清除治疗**，如利妥昔单抗、伊奈利珠单抗。这类药物会让疫苗产生抗体的能力下降，因此即使接种了，**保护效果也可能打折扣**### 3. 接种时机很重要。通常建议：- **最好在开始免疫抑制治疗前完成疫苗接种**- 如果已经在治疗中，尽量选择医生建议的“免疫抑制相对较弱”或“下一次用药前合适窗口期”接种。例如：- 利妥昔单抗治疗者，常常会考虑在**下次输注前一段时间**评估接种机会- 但具体间隔要由神经免疫科或风湿免疫科医生根据化验结果决定。## 眼科角度特别提醒。NMOSD 常累及**视神经炎**，患者最担心的是：“接种疫苗会不会诱发复发，导致视力再下降？”目前总体认为：- **规范接种灭活疫苗并不等于一定诱发复发**- 相比之下，**严重感染本身更可能诱发免疫波动和疾病复发**所以很多时候，**预防感染反而是有意义的**## 建议你这样做。在接种前最好完成以下评估：- 当前 NMOSD 是否稳定- 最近是否刚发生视神经炎或脊髓炎复发- 正在用什么药，最近一次用药时间- 血常规- 淋巴细胞计数- 免疫球蛋白水平- 是否有发热或感染## 什么时候暂缓接种，以下情况通常建议先缓一缓：- 正处于急性复发期- 正在大剂量激素冲击治疗期间- 有明确感染- 医生判断当前免疫抑制过强## 一句话总结**NMOSD 用药期间并非绝对不能打疫苗，灭活疫苗多可考虑，活疫苗通常应避免；关键在于药物种类、病情稳定性和接种时机。**如果你愿意，我可以进一步帮你按**具体药物**来分析，例如：- 利妥昔单抗期间怎么打疫苗- 沙妥珠单抗期间能不能打流感疫苗- 新冠疫苗接种时机怎么选

**11、如何缓解NMOSD引起的长期神经性疼痛、肌肉痉挛或顽固性瘙痒？**

NMOSD 相关的长期神经性疼痛、肌肉痉挛、顽固性瘙痒，常见于视神经脊髓炎谱系疾病累及脊髓或中枢感觉通路之后，属于“神经病理性症状”。这类不适往往不是普通止痛药就能完全解决，通常需要神经科主导、眼科与康复科协作长期管理。先说重点：如果你是 NMOSD 患者，出现这些症状，不代表一定在复发，但也不能完全掉以轻心。若症状在短时间内明显加重，或伴随视力下降、肢体无力、感觉平面变化、排尿排便异常，应尽快就医，排查复发。## 一、如何缓解长期神经性疼痛。NMOSD 的疼痛常表现为:- 灼烧样痛- 电击样痛- 刀割样痛- 触碰就痛- 胸腹“束带感”- 四肢麻痛、深部酸痛### 常用治疗思路#### 1. 一线常用药物。这类疼痛一般优先考虑神经病理性疼痛药物，而不是单纯消炎止痛药。常见包括：- 加巴喷丁- 普瑞巴林- 度洛西汀- 阿米替林。这些药物有时能减轻：- 灼痛- 麻痛- 夜间痛- 影响睡眠的疼痛。#### 2. 对普通止痛药的认识- 对乙酰氨基酚- 布洛芬等非甾体抗炎药。这类药对 NMOSD 的“神经性疼痛”通常效果有限，但如果同时有肌肉骨骼痛、姿势性劳损，也可作为辅助。#### 3. 顽固疼痛时。若症状持续、影响睡眠和活动，医生可能会：- 联合用药- 缓慢加量- 必要时转疼痛科或神经科专病门诊## 二、如何缓解肌肉痉挛和僵硬。NMOSD 脊髓受累后，常见：- 小腿抽紧- 大腿内收- 夜间痉挛- 肌张力增高- 走路发硬。### 常用办法#### 1. 药物治疗。常见解痉药包括：- 巴氯芬- 替扎尼定。部分患者可改善：- 肌肉发紧- 抽动- 夜间痛性痉挛- 翻身困难。#### 2. 康复和拉伸。药物之外，规律康复非常重要：- 每日温和拉伸- 关节活动训练- 步态训练- 物理治疗- 热疗。很多患者单靠吃药效果一般，配合康复后会更稳定。#### 3. 痉挛诱因管理。以下情况常使痉挛加重：- 尿路感染- 便秘- 疼痛刺激- 睡眠差- 过度疲劳- 体位不适。处理诱因，往往能明显减轻痉挛。## 三、如何缓解顽固性瘙痒。NMOSD 可出现“中枢性瘙痒”，特点是：- 皮肤看起来没明显皮疹- 越抓越难受- 常规抗过敏药效果差- 可伴针刺感、灼热感或麻木### 处理原则#### 1. 先判断是不是神经性瘙痒：如果是中枢性瘙痒，常规方法如：- 外用止痒药- 抗组胺药，往往效果不理想。#### 2. 常用药物：可考虑与神经性疼痛相似的药物：- 加巴喷丁- 普瑞巴林- 某些抗抑郁药医生会根据瘙痒部位、严重程度、睡眠影响来调整。#### 3. 皮肤护理：虽然根源在神经，但皮肤护理仍有帮助：- 避免热水烫洗- 使用温和保湿剂- 穿柔软透气衣物- 避免反复搔抓## 四、最关键的一点：控制原发病：如果 NMOSD 控制不好，这些症状可能反复或加重。所以除了对症缓解，更重要的是坚持规范的复发预防治疗。通常需要神经科评估是否正在使用合适的长期免疫治疗方案。若频繁复发，疼痛、痉挛、瘙痒就更难控制。## 五、生活中能做什么？你可以尝试以下方法辅助缓解：- 保持规律睡眠- 避免过劳- 适度活动，不要久卧- 记录症状日记- 识别加重因素- 保持大便通畅- 及时处理泌尿系统问题- 焦虑和抑郁明显时寻求帮助。因为：- 疼痛会加重睡眠障碍- 睡眠差会放大疼痛和痉挛- 情绪问题又会进一步放大症状。这是一个相互影响的循环，需要一起干预。## 六、什么时候需要尽快就医？出现以下情况要尽快联系神经科或急诊：- 疼痛、瘙痒、痉挛突然明显加重- 新发视力下降或眼痛- 新发肢体无力- 走路明显变差- 尿潴留或失禁- 持续发热或怀疑感染- 药物后明显嗜睡、头晕、跌倒## 七、眼科医师特别提醒：从眼科角度说，NMOSD 最重要的眼部问题是视神经炎。如果在疼痛、瘙痒、痉挛之外，还出现：- 视力下降- 色觉变差- 眼球转动痛- 视野缺损。要警惕视神经受累复发，不能只当作普通疲劳或干眼。## 简要总结：缓解 NMOSD 引起的长期神经性疼痛、肌肉痉挛、顽固性瘙痒，核心是三件事：- 用对药：神经性疼痛药、解痉药，而不是只靠普通止痛药- 做好康复和诱因管理：拉伸、睡眠、感染和便秘控制- 控制 NMOSD 本身：防止复发，减少新的神经损伤。如果你愿意，我还可以继续帮你整理一版更实用的内容，比如：- NMOSD 疼痛用药一览表- 痉挛和瘙痒的家庭管理清单- 哪些症状提示可能复发，哪些更像后遗症

**12、NMOSD导致的大小便功能障碍怎么办？**

NMOSD也就是视神经脊髓炎谱系疾病，如果已经出现大小便功能障碍，通常提示脊髓受累较明显，这类问题**不能只当作单纯泌尿或肠道问题处理**，需要把它看作**神经系统急症或重要并发症**。从眼科相关角度讲，NMOSD患者常因视神经炎来就诊，但**大小便异常更多提示脊髓病变活动**，应尽快由**神经内科为主导**处理，必要时联合泌尿外科、康复科。## 先说结论：如果是**新近出现**或**突然加重**的以下情况，应尽快去急诊或联系神经内科：- 尿潴留，尿不出来- 尿失禁明显加重- 大便失禁- 严重便秘伴腹胀、腹痛- 双下肢无力、麻木加重- 行走困难- 发热、尿痛、腰痛，怀疑尿路感染。因为这可能意味着：- NMOSD复发- 脊髓炎加重- 神经源性膀胱或肠功能障碍- 合并尿路感染、肾功能损害等。## 常见表现：NMOSD导致的大小便功能障碍，常见包括：- 尿频、尿急- 排尿困难- 尿不尽- 尿潴留- 夜尿增多- 尿失禁- 便秘- 排便费力- 大便失禁。本质上多与**脊髓炎后神经传导受损**有关。## 该怎么办### 1 先判断是不是复发。如果大小便问题是近期新出现，或在几天内明显恶化，尤其同时有：- 视力下降- 眼痛- 双腿麻木无力- 背部紧束感- 感觉异常。要高度怀疑NMOSD活动。这时通常需要神经内科评估，常见会做：- 脊髓和脑MRI- 尿常规、尿培养- 残余尿量检查- 肾功能检查- 必要时泌尿系超声。急性期治疗通常由神经内科决定，常见包括：- 大剂量激素冲击- 血浆置换- 免疫治疗调整### 2 处理排尿障碍。这部分很关键，因为长期处理不当会反复感染，甚至影响肾脏。如果是**尿潴留、排不净**：- 需要测残余尿量- 残余尿多时，医生可能建议**间歇导尿**- 不建议长期憋尿- 不要自行反复使用抗生素。如果是**尿急、尿频、尿失禁**：- 先排除尿路感染- 医生会根据膀胱功能类型用药- 可能需要膀胱训练- 控制饮水节奏，不是盲目少喝水。日常建议：- 定时排尿- 记录排尿日记- 避免浓茶、咖啡、酒精等刺激- 保持会阴部清洁- 若反复感染，要规范做尿培养后再用药### 3 处理排便障碍。如果是便秘：- 保证足够饮水- 增加膳食纤维- 尽量固定时间排便- 适当活动和康复训练- 必要时在医生指导下使用缓泻药或开塞露。如果是大便失禁：- 需要康复科或神经内科评估- 建立规律排便程序- 注意肛周皮肤护理- 必要时使用护理用品，减少皮肤损伤和感染### 4 预防并发症：NMOSD合并大小便功能障碍时，要特别注意：- 尿路感染- 肾积水- 肾功能损害- 粪便嵌塞- 压疮- 焦虑和抑郁。建议定期复查：- 尿常规- 肾功能- 泌尿系超声- 残余尿量### 5 长期核心是控制原发病：如果NMOSD控制不好，脊髓损伤可能反复发生，大小便问题也更容易持续或加重。因此长期最重要的是：- 规律随访神经内科- 规范使用预防复发的免疫治疗- 不随意停药- 出现复发征象尽早就医## 眼科医师要特别提醒的一点：很多NMOSD患者最先关注的是视力，但**大小便功能障碍往往比眼部症状更提示脊髓受累严重程度**。所以如果你本身是NMOSD患者，哪怕这次是因为眼睛问题来问诊，只要合并：- 排尿困难- 尿失禁- 便秘明显加重- 大便失禁。都建议尽快转神经内科评估，而不是只在眼科观察。## 什么时候必须立即就医？出现以下任一情况，不要拖：- 完全尿不出来- 下腹胀痛明显- 高热、寒战- 尿中带血- 腰痛伴发热- 双腿突然无力- 会阴部感觉减退- 大便完全失控## 一句话总结NMOSD导致的大小便功能障碍，多数是**脊髓受累后的神经源性膀胱和肠功能障碍**，关键不是单纯对症，而是要**尽快评估是否复发、规范神经内科治疗、联合泌尿和康复管理、预防感染和肾损害**。如果你愿意，我还可以继续帮你整理一份：- **NMOSD患者大小便功能障碍就诊清单**- **居家护理要点**- **什么时候提示复发的自查表**

**13、NMOSD病情稳定后，应该如何进行科学、安全的康复训练？**

NMOSD 也就是视神经脊髓炎谱系障碍。病情稳定后，康复训练的目标不是“拼强度”，而是**在不诱发复发、不加重疲劳的前提下，逐步恢复视功能、肢体功能、平衡能力和日常生活能力**。如果患者曾有视神经炎、脊髓炎或脑干受累，康复方案需要个体化，由神经科、眼科、康复科共同评估后执行。下面我从临床上更实用的角度讲。## 一、开始康复前，先确认是否真的进入“稳定期”。通常建议满足以下几点再进入系统康复训练：- 近期无新发复发症状- 视力、肢体无明显进行性下降- 激素冲击或血浆置换等急性期治疗已结束- 生命体征平稳，感染已控制- 主治医生认为可以开始康复。如果出现以下情况，应先暂停强化训练并尽快复诊：- 视力突然下降- 眼痛加重，尤其转眼痛明显- 新出现肢体无力、麻木、行走不稳- 尿便功能明显变化- 持续发热、感染、明显疲劳恶化## 二、NMOSD稳定期康复的总原则### 1. 循序渐进。训练强度从低到中等，避免一开始就高强度运动。### 2. 避免过度疲劳。NMOSD患者很容易出现疲劳加重，过量训练反而会让功能下降。遵循“**练后轻度疲劳，24小时内能恢复**”这一原则较安全。### 3. 避免热暴露。高温环境、热水澡、桑拿、闷热天气下训练，可能让神经传导暂时变差，症状加重。建议在凉爽、通风环境训练。### 4. 重视安全防跌倒。若存在下肢无力、痉挛、平衡差、视力障碍，训练时必须有保护措施。### 5. 个体化。不同患者后遗症不同：- 以视神经损伤为主：重点是视觉康复和生活适应- 以脊髓损伤为主：重点是步态、肌力、平衡、膀胱功能管理- 有痉挛、疼痛、感觉异常者：训练方案要调整## 三、稳定后可以做哪些康复训练## 1. 有氧训练。适合大多数稳定期患者，有助于改善体能、疲劳、情绪和睡眠。可选方式：- 平地步行- 固定自行车- 椭圆机- 水温适中的水中运动- 太极、舒缓体操。建议：- 每周 3到5 次- 每次 10到30 分钟起步- 以低到中等强度为主- 运动时可以正常说话，但不应气喘明显。如果体力差，可以采用：- 5到10分钟一段- 中间休息- 一天分 2到3 次完成## 2. 肌力训练。如果存在肢体无力、久病后肌肉萎缩、步态差，肌力训练很重要。训练部位重点：- 下肢：股四头肌、臀肌、踝背伸肌- 核心：腰腹肌群- 上肢：肩带和手部功能。常见方法：- 弹力带训练- 小重量抗阻训练- 坐站训练- 台阶训练- 床上主动运动。建议：- 每周 2到3 次- 每个动作 8到12 次- 1到3 组- 先练大肌群，再练精细动作。注意：- 若训练后痉挛明显加重，要降低强度- 不建议“练到力竭”## 3. 平衡与步态训练。如果脊髓受累后有走路不稳、拖步、容易跌倒，平衡训练非常关键。训练内容可包括：- 站立重心转移- 扶物单脚站立- 直线行走- 跨步训练- 转身训练- 上下台阶- 助行器或手杖辅助步行训练。安全要点：- 一开始必须有人保护- 家中先清除地面障碍物- 穿防滑鞋- 夜间起床注意照明。如果步态明显异常，建议由康复治疗师先做评估，再决定是否使用：- 踝足矫形器- 手杖- 助行器- 轮椅## 4. 牵伸与痉挛管理。很多NMOSD患者会有肌张力增高、腿紧、抽筋、关节活动受限。适合的训练：- 小腿后侧牵伸- 腘绳肌牵伸- 髋屈肌牵伸- 肩颈放松- 关节活动度训练。建议：- 每天进行- 每个动作保持 20到30 秒- 重复 3到5 次- 动作缓慢，不要弹震式拉伸。如果痉挛严重到影响走路、睡眠或护理，需要和医生讨论药物或肉毒毒素等治疗，而不仅靠训练。## 5. 视觉相关康复。如果患者有视神经炎后遗留的视力下降、视野缺损、对比敏感度下降、畏光、视觉疲劳，眼科康复很重要。可考虑以下方向：### 视觉环境优化- 增加室内均匀照明- 阅读时使用高对比度材料- 放大字体- 减少眩光- 必要时佩戴遮光镜片### 视觉辅助工- 放大镜- 电子助视器- 大字阅读设备- 高对比度手机与电脑设置- 语音辅助功能### 视觉训练：对于部分患者，可在专业人员指导下进行：- 注视稳定训练- 眼球追踪训练- 扫视训练- 阅读定位训练。但要强调：**NMOSD导致的视神经损伤，本质上不是单纯“眼肌问题”，视觉训练不能替代神经炎治疗，也不能保证恢复视力。** 它更多是帮助提升残余视功能利用效率。### 日常用眼原则- 20到30分钟近距离用眼后休息- 避免长时间连续盯屏- 光线舒适，不要过暗或过亮- 出现明显眼痛、视物模糊加重时及时停止## 6. 精细动作与作业训练如果有手麻、手无力、动作不灵活，可做作业治疗训练，例如：- 握力训练- 捏夹训练- 扣扣子、拿筷子、写字训练- 日常生活动作训练。目标是提高：- 穿衣- 吃饭- 洗漱- 如厕- 使用手机和电脑的能力## 7. 膀胱、肠道和盆底管理：NMOSD脊髓受累后，常见排尿困难、尿急、尿频、尿失禁或便秘。康复建议：- 规律排尿- 记录饮水与排尿日记- 避免憋尿- 预防尿路感染- 便秘者增加膳食纤维和适量活动- 必要时做盆底康复或膀胱训练。如果出现：- 尿潴留- 反复尿路感染- 明显失禁。需要泌尿或康复专科进一步评估。## 四、一个相对安全的康复训练框架。下面给你一个稳定期常用的基础版模板，前提是医生已同意开始训练。A[开始前评估 视力 肌力 平衡 疲劳 痉挛 排尿情况] --> B[热身 5到10分钟]B --> C[有氧训练 10到20分钟]C --> D[肌力训练 15到20分钟]D --> E[平衡步态训练 10到15分钟]E --> F[牵伸放松 5到10分钟]F --> G[记录训练后疲劳 疼痛 视力变化]G --> H[根据次日恢复情况调整强度]### 每周参考安排- 有氧训练：3到5天- 肌力训练：2到3天- 平衡训练：3天以上- 牵伸放松：每天- 视觉与精细动作训练：根据症状每日少量进行## 五、怎样判断训练强度是否合适。比较实用的标准：### 合适- 训练中微微累，但能坚持- 训练后休息一晚或24小时内恢复- 第二天不觉得症状明显加重### 过量- 训练后极度疲劳，恢复超过24小时- 视物更模糊、眼胀痛明显- 下肢发软明显，走路更差- 痉挛、疼痛明显增加- 出现“越练越差”的感觉一旦过量，就要减量，而不是硬撑。## 六、NMOSD患者特别需要避免的康复误区### 1. 以为越累越有效。神经系统疾病不是健身训练，过度疲劳可能适得其反。### 2. 只练眼睛，不管全身。即使主要表现为视神经炎，长期活动减少也会导致体能下降、焦虑、睡眠差。### 3. 发作刚缓解就做高强度运动。急性恢复期神经功能还不稳定，训练需要温和过渡。### 4. 忽视长期复发预防治疗。康复不能替代规范免疫治疗。NMOSD是否稳定，核心仍取决于规范随访和复发预防。### 5. 天热或洗热水澡后马上运动。热应激可能让症状一过性加重。## 七、哪些患者更需要专业康复团队介入。以下情况建议尽快到正规医院康复医学科就诊：- 视力损害影响阅读、工作、出行- 反复跌倒- 下肢痉挛严重- 无法独立行走- 手功能明显受限- 尿便障碍明显- 疲劳严重影响生活- 合并焦虑、抑郁、睡眠障碍## 八、眼科角度特别提醒如果你问的是“眼科相关康复”，我特别强调三点：### 1. 视功能下降后不要盲目做所谓“视力恢复训练”NMOSD相关视力损伤多源于视神经炎后的神经损害，不是普通近视训练那一套。### 2. 重点是低视力康复与视觉适应。包括：- 提高阅读效率- 训练视线扫描- 使用辅助器具- 改善光环境- 防跌倒和安全出行### 3. 任何新发视力变化都按复发先处理。如果突然视力再降、颜色变淡、视野缺损加重、眼球转动痛再现，应优先排查复发，而不是继续训练。## 九、给患者的简明建议。可以记住这 6 句话：- 稳定后再练，不在复发期硬练- 少量多次，比一次练猛更好- 微微累可以，累到第二天恢复不过来就过量了- 避热、防跌倒、重安全- 视觉康复重在“利用剩余功能”，不是保证把视力练回来- 康复和免疫治疗要两条线一起走。如果你愿意，我还可以进一步帮你做一份更实用的内容，例如：- **NMOSD稳定期一周康复训练计划表**- **合并视神经炎后遗症的视觉康复方案**- **合并下肢无力和痉挛的家庭训练方案**- **适合家属陪同执行的康复注意事项清单**如果需要，我可以直接按你的具体情况继续细化。

**14、NMOSD能治愈吗？**

NMOSD一般**难以称为“治愈”**，但**可以通过规范治疗实现长期控制**，尽量减少复发、保护视神经和脊髓功能，部分患者可以长期稳定。### 先说结论。NMOSD，中文常称**视神经脊髓炎谱系疾病**，是一种**免疫介导的中枢神经系统疾病**，常累及**视神经**，所以在眼科常表现为反复视神经炎、视力下降、眼痛等。它的特点不是“自限后彻底根除”，而是**容易复发**。因此临床目标通常是：A[急性期尽快控制炎症] --> B[缓解视力与神经损伤]B --> C[长期预防复发]C --> D[尽量保留视功能和生活能力]### 为什么说不能轻易讲“治愈”。因为NMOSD往往与异常免疫反应有关，尤其部分患者可检测到**AQP4抗体**。这类疾病目前医学上更多是：- **可治疗**- **可控制**- **可长期缓解**- **但复发风险仍存在**一次复发就可能造成比较明显的视力或神经功能损伤，所以比起“能不能根治”，更重要的是**能不能尽早诊断并长期规范管理**。### 目前怎么治疗。通常分为两部分：#### 1. 急性发作期治疗。目的是尽快压制炎症，减少本次攻击造成的损害，常见方案包括：- 大剂量糖皮质激素冲击- 血浆置换- 必要时其他免疫治疗。如果是眼科首发表现，比如突然视力下降、视物发暗、眼球转动痛，应尽快就诊，越早治疗越好。#### 2. 缓解期预防复发。这是NMOSD治疗的核心。医生会根据抗体情况、复发次数、病情严重程度等选择长期免疫治疗方案。规范维持治疗后，很多患者复发次数可明显减少。### 对视力的影响能恢复吗？这取决于多个因素：- 是否首次发作- 发作时病情轻重- 治疗是否及时- 是否反复复发- 视神经损伤是否已经较重。有些患者经过积极治疗后视力能恢复较好；也有部分患者因视神经受损明显，恢复有限。 **NMOSD最怕反复发作**，因为每复发一次，都可能让视功能进一步下降。### 患者最需要关注什么？如果已经明确是NMOSD，建议重点做到：- **规律随访**- **按医嘱长期用药，不要自行停药**- 出现视力下降、肢体麻木无力、呕吐顽固、走路不稳等症状要及时就医- 同时由**神经内科**与**眼科**协作管理。### 可以这样理解。NMOSD更像一种**需要长期管理的慢性高复发性疾病**，目前多数情况下**不能简单说彻底治愈**，但**通过规范治疗，很多患者可以获得长期稳定，减少失明和致残风险**。如果你愿意，我还可以继续从眼科角度给你讲：- **NMOSD和普通视神经炎有什么区别**- **AQP4阳性意味着什么**- **NMOSD视力恢复的概率怎么看**

**15、NMOSD的长期预后怎么样？**

NMOSD 也叫视神经脊髓炎谱系障碍。若从眼科和神经免疫角度看，它的长期预后可以概括为一句话：**预后差异很大，但总体属于“可长期管理、需积极预防复发”的疾病。**## 一、长期预后取决于什么？NMOSD 的残疾累积，**主要不是来自疾病慢慢退化**，而是**来自一次次复发后的损伤遗留**。也就是说：- **复发越多，长期功能受损风险越高**- **早期规范治疗越充分，预后通常越好**- **视神经炎、脊髓炎、脑干受累后恢复程度**，决定今后视觉和行动能力。影响预后的关键因素包括：- **是否为 AQP4 抗体阳性**- AQP4 阳性患者通常更符合经典 NMOSD，若不治疗，复发风险往往较高- **首次发作严重程度**- 比如视力骤降到仅光感、双眼受累，或脊髓炎导致明显瘫痪、大小便障碍- **是否反复发作*- 每次复发都可能留下不可逆后遗症- **是否尽早使用预防复发的长期免疫治疗**- 这是改善长期预后的核心- **治疗反应是否良好**- **是否合并感染、药物副作用或其他自身免疫病**## 二、从眼科角度看，视力长期预后如何？NMOSD 常见严重视神经炎。其视力预后比普通脱髓鞘性视神经炎往往更重一些。可能出现的长期情况包括：- 单次发作后视力恢复较好，长期相对稳定- 视力恢复不完全，遗留：- 视力下降- 视野缺损- 色觉下降- 对比敏感度下降- 反复视神经炎后出现：- 视神经萎缩- 持续性低视力- 双眼严重视力损害，少数可致功能性失明### 视力预后较差的提示因素- 发作时视力损伤非常重- 双眼受累- 治疗启动较晚- 激素冲击效果不佳且未及时行血浆置换- 反复复发。因此，**NMOSD 患者一旦出现视力下降、眼痛、色觉变差，应视为急症尽快处理**。## 三、整体神经功能的长期预后。除眼部外，NMOSD 还可能影响脊髓、脑干和部分脑区。长期可能遗留：- 肢体无力或瘫痪- 麻木、感觉异常- 行走困难- 痉挛和神经痛- 膀胱、肠道功能障碍- 顽固性呃逆、恶心呕吐等脑干症状- 疲劳、睡眠问题、情绪问题。过去在缺乏有效维持治疗时，患者反复复发后致残率较高。而现在随着：- 早期识别- 急性期规范抢救- 长期免疫预防治疗- 康复与并发症管理**很多患者的长期结局比过去明显改善。**## 四、现在的长期生存和生活质量怎么样### 1. 生存率比过去好了很多以前 NMOSD 因重症复发、感染、呼吸衰竭等原因，长期结局较差。现在随着诊断和治疗进步，**总体生存率明显提高**。### 2. 生活质量仍可能受影响。即使没有明显瘫痪或失明，患者也可能长期存在：- 视觉质量下降- 慢性疼痛- 疲劳- 焦虑抑郁- 对复发的担忧- 工作和驾驶能力受限。因此评估预后，不能只看“能不能活”，还要看：- 视力和视野- 步行能力- 排尿排便功能- 疼痛与疲劳- 心理状态- 社会功能。## 五、哪些情况提示预后相对较好。通常来说，以下情况更有利：- 早期明确诊断- 复发次数少- 每次发作后恢复较好- 尽早开始并坚持维持治疗- 随访规范- 复发时能迅速就医并接受激素或血浆置换等治疗- OCT、视野、MRI、神经功能评估较稳定## 六、哪些情况提示需要更警惕。以下情况说明未来残疾风险更高，需要严密管理：- 频繁复发- 每次复发恢复都不理想- 曾有严重双眼视神经炎- 长节段脊髓炎- 脑干受累明显- 治疗依从性差，擅自停药- 合并严重感染或药物不耐受## 七、患者最关心的问题：能不能“治好”。从目前医学角度，NMOSD 一般认为：- **多数情况下不能简单理解为“根治”**- 但**可以通过长期规范治疗，把复发风险尽量降到最低**- 很多患者能够长期稳定生活、学习和工作。所以治疗目标是：- 急性发作时尽量减少神经损伤- 缓解期尽量预防复发- 尽量保住视力、行动能力和生活质量## 八、给患者的实际建议。如果你或家人已经确诊 NMOSD，最重要的是：- **坚持在神经免疫或神经眼科专科长期随诊**- **不要自行停用维持治疗**- 出现以下情况立即就医：- 视力下降- 色觉明显变差- 新发肢体无力或麻木- 行走困难- 排尿困难或尿潴留- 持续呃逆、恶心呕吐- 定期复查：- 视力- 视野- OCT- 眼底或视神经评估- 脑和脊髓 MRI- 相关抗体及安全性化验## 九、一句话总结**NMOSD 的长期预后核心看“复发控制得好不好”。** 若未规范治疗，复发后可造成严重视力损害和神经功能残疾；若早诊断、早治疗、长期规范管理，很多患者可以明显减少复发，获得更好的长期视力和生活质量。如果你愿意，我还可以继续帮你详细讲：- **NMOSD 视神经炎和普通视神经炎的区别**- **NMOSD 常用长期药物怎么选**- **AQP4 阳性和阴性患者预后差别**

**16、NMOSD患者可以怀孕生子吗？**

可以，但需要**在神经免疫科、眼科和产科联合评估后，有计划地妊娠**。NMOSD 也就是视神经脊髓炎谱系疾病，患者**并不是绝对不能怀孕生子**。很多患者经过规范管理后可以顺利妊娠并分娩。但这类疾病有几个特点需要特别重视：A[NMOSD患者有生育需求] --> B[先评估病情是否稳定]B --> C[评估当前用药是否影响胎儿]C --> D[制定孕前停药或换药方案]D --> E[孕期严密随访]E --> F[产后预防复发]### 一、为什么说可以怀孕，但要谨慎。NMOSD 属于复发性自身免疫性疾病，妊娠本身不一定阻止生育，但**怀孕前后尤其产后复发风险可能增加**。如果在疾病活动期怀孕，可能带来这些问题：- 视神经炎复发，导致视力下降- 脊髓炎复发，出现肢体无力、麻木、排尿排便障碍- 产后数月内复发风险上升- 某些治疗药物可能对胎儿有致畸风险。所以核心不是“能不能怀”，而是“**是否适合现在怀、如何安全地怀**”。### 二、什么情况下更适合备孕。通常建议满足以下条件再考虑怀孕：- **病情稳定一段时间**- 一般希望至少 **6到12个月没有明显复发**- **视力和神经功能相对稳定**- **目前使用的药物可以在医生指导下调整为妊娠相对安全方案**- **没有严重残疾或重要器官并发症**- **有规律随访条件**### 三、最重要的是药物问题。这点非常关键。NMOSD 常用药物中，有些**不能在怀孕前后随意继续使用**，也不能自行停药。常见原则如下：- **某些免疫抑制药可能影响胎儿**- 需要提前停药，并留出“洗脱期”- **某些药物在妊娠期相对更常被考虑**- 但是否适用，要结合个人病情- **生物制剂是否能继续用**- 需要专科医生根据妊娠阶段、复发风险、既往病情判断- **急性复发期的治疗**- 妊娠期如果复发，通常仍有可用治疗手段，但必须由医生决定**不要因为想怀孕就自行停药。**对 NMOSD 患者来说，擅自停药可能比继续规范管理更危险。### 四、怀孕期间要注意什么。孕期管理重点包括：- 定期看神经免疫专科- 同步接受产科高危妊娠管理- 眼科评估视力、视野、视神经情况- 一旦出现以下情况尽快就医- 视物模糊- 眼痛- 肢体麻木无力- 行走不稳- 尿潴留或失禁- 持续呕吐、顽固性打嗝等脑干症状### 五、产后更需要警惕。很多 NMOSD 患者**产后是复发高风险阶段**，尤其是产后前几个月。因此在分娩前就要制定好产后方案：- 是否尽早恢复维持治疗- 是否母乳喂养- 哺乳与用药是否冲突- 出现复发征象时如何快速就诊### 六、关于遗传和孩子健康NMOSD **不是典型的直接遗传病**，一般不是“母亲有病，孩子一定会得”。但自身免疫性疾病可能存在一定体质易感性，因此：- 不必过度担心“肯定遗传”- 也不能完全忽视家族自身免疫背景- 孩子大多数情况下可以正常出生和成长### 七、我给患者的实际建议。如果你是 NMOSD 患者并想怀孕，建议按这个顺序做：A[明确有生育计划] --> B[预约神经免疫专科]B --> C[同时就诊眼科与产科]C --> D[评估近一年复发情况]D --> E[核对AQP4或MOG抗体与影像资料]E --> F[调整妊娠期方案]F --> G[病情稳定后再备孕]### 八、结论**NMOSD患者通常可以怀孕生子，但属于高风险妊娠，需要在病情稳定期、医生指导下计划怀孕。**关键点是：- 怀孕前先评估病情- 药物必须提前规划- 孕期密切随访- 产后重点预防复发。如果你愿意，我还可以进一步帮你整理一份：- **NMOSD备孕前检查清单**- **孕期与哺乳期常见用药风险提示**- **出现眼部症状时何时需要立刻就医的判断标准**

**17、孕期和产后如何管理疾病，以降低NMOSD复发风险？**

以下内容供一般医学科普参考，不能替代你的神经免疫专科医生与产科医生的个体化方案。NMOSD在妊娠期尤其是**产后前3到6个月**复发风险可升高，目标是：**孕前稳定、孕中严密监测、产后尽早预防**。## 一、先说结论。如果你患有NMOSD并计划怀孕，通常最重要的是：A[孕前评估 disease activity] --> B[尽量在病情稳定后再妊娠]B --> C[与神经免疫专科和产科共同制定用药方案]C --> D[孕期持续监测 relapse signs]D --> E[产后尽早启动预防复发策略]E --> F[重点防范产后3到6个月复发]## 二、孕前管理最关键### 1. 尽量在疾病稳定后再怀孕。一般希望在怀孕前已有一段相对稳定期，这样妊娠和产后复发风险通常更低。### 2. 孕前做完整评估。建议和医生确认：- 是否为AQP4抗体阳性- 既往复发频率和严重程度- 近期是否有视神经炎或脊髓炎- 目前使用的免疫治疗是否适合备孕- 是否合并其他自身免疫病- 基础血常规、肝肾功能、感染筛查### 3. 提前调整药物。这一步非常重要，因为不同NMOSD药物对妊娠安全性差异很大。原则是：- **不要自行停药**- **高风险药物需提前停用并过渡**- 选择孕期相对可管理的方案，以兼顾母体疾病控制和胎儿安全。临床上常见思路是：- 对明确不适合妊娠的药物，提前停药并留出洗脱期- 必要时改用医生认为孕期可继续或相对更可控的治疗- 对既往高复发患者，孕前就要把产后预防方案一起设计好## 三、孕期如何降低复发风险。NMOSD并不像某些其他脱髓鞘病那样在孕期一定明显“被保护”，所以孕期仍需警惕复发。### 1. 规律随访。建议同时接受：- 神经免疫专科随访- 高危妊娠产科管理- 必要时眼科随访，尤其既往有视神经炎者### 2. 警惕早期复发信号。一旦出现以下情况，要尽快就医：- 视力下降- 眼痛- 新发肢体无力- 感觉异常- 持续呕吐、顽固性打嗝- 行走困难- 排尿排便异常### 3. 避免诱发因素。虽然不能完全避免复发，但应尽量减少诱因：- 感染- 过度疲劳- 睡眠不足- 情绪压力过大- 擅自停药。### 4. 复发时及时治疗。如果孕期出现明确复发，医生通常会根据孕周和严重程度权衡处理。重在：- 尽早识别- 尽早治疗- 避免遗留不可逆神经功能损害。常见急性期处理思路包括：- 大剂量糖皮质激素- 对激素反应差或重症者考虑血浆置换等救治方式。具体是否适合，要由专科团队根据孕周和病情决定。## 四、产后是复发高风险期。这是NMOSD管理中最需要重视的阶段。### 1. 产后尽早复诊。建议在分娩后尽早联系神经免疫专科，不要等症状明显了才处理。### 2. 尽快恢复或启动维持治疗。很多患者产后是否复发，和**维持治疗是否及时接上**关系很大。尤其既往复发频繁、AQP4阳性、停药时间较长者，更要提前安排。### 3. 哺乳与用药要提前规划。这是患者最常见的困惑之一。核心原则：- 想母乳喂养，要在孕晚期就和医生讨论药物兼容性- 有些药物可在哺乳期权衡使用- 有些药物可能需要暂停母乳或改方案- 如果你的复发风险很高，通常**优先保证母体疾病控制**换句话说：**产后是否母乳，不应只看喂养意愿，更要看NMOSD复发风险和所需药物。## 六、患者最关心的几个实际问题### 1. 怀孕了能不能把药全停掉**不建议自行全部停药。**对NMOSD来说，停药可能显著增加复发风险，尤其产后更明显。### 2. 母乳喂养能不能预防复发。目前不能简单认为母乳喂养本身能明显保护NMOSD不复发。是否母乳，需要与疾病控制一起权衡。### 3. 如果孕期复发，会不会影响宝宝。轻重程度不同，影响也不同。关键是尽快处理母体复发，因为严重复发本身会影响母体健康，间接也可能影响妊娠结局。### 4. 剖宫产会不会比顺产更安全。分娩方式通常主要依据产科指征决定，不是单纯因为NMOSD就一定要剖宫产。## 七、给你的实用建议清单 [降低NMOSD妊娠相关复发风险]孕前：专科评估-病情稳定后怀孕- 药物提前调整-制定产后方案；孕期：定期随访-识别复发信号-避免感染疲劳-出现症状立即就医；产后：早复诊-尽快恢复维持治疗-讨论母乳与用药- 严密观察3到6个月## 八、什么时候必须尽快就医。如果你在孕期或产后出现以下任何一种情况，请尽快急诊或联系专科：- 单眼或双眼视力突然下降- 四肢无力或麻木加重-不能走路- 尿潴留或失禁- 持续呕吐、严重打嗝- 呼吸困难- 明显背痛合并神经功能恶化## 九、一句话总结**NMOSD患者在孕期和产后降低复发风险的核心，是孕前稳定病情、不要擅自停药、孕期严密监测、产后尽早恢复预防性治疗，尤其重视产后前3到6个月。**如果你愿意，我还可以继续帮你整理一份：- **NMOSD备孕用药风险对照表**- 或者 **孕期与哺乳期常见药物怎么和医生沟通的提问清单**

**18、NMOSD会遗传给下一代吗？**

NMOSD通常**不属于经典遗传病**，一般**不会像某些单基因遗传病那样直接遗传给下一代**。### 简单说。NMOSD是一种**自身免疫性疾病**，主要与免疫系统异常有关。它的发病机制比较复杂，目前认为和以下因素相关：A[NMOSD发病相关因素] --> B[免疫系统异常]A --> C[体内自身抗体 例如AQP4抗体]A --> D[环境因素]A --> E[个体易感性]### 会不会遗传：大多数患者的情况是：- **没有明确家族遗传模式**- **父母患病 子女并不会必然得病**- 可能存在一定的**遗传易感性**，也就是家族成员在免疫相关疾病方面可能比普通人更容易出现问题，但这种风险总体还是**较低**### 需要注意的几点：如果您或家人患有NMOSD，下一代通常**不需要因为这一点就过度担心**，但建议注意：- 若家族中有多位成员患自身免疫病，应提高关注- 出现以下症状时尽早就诊神经内科或眼科：- 视力下降- 眼痛- 肢体麻木无力- 顽固性呕吐- 排尿排便异常### 备孕或怀孕时：如果患者本人正准备怀孕，还需要额外关注：- 疾病是否处于稳定期- 目前所用药物是否影响妊娠- 是否需要神经内科与产科联合管理### 总**NMOSD一般不会直接遗传给下一代，但可能存在轻度遗传易感性。**也就是说，**子女患病风险通常不高，不属于“必然遗传”或“高概率遗传”疾病。**如果您愿意，我还可以进一步从患者角度给您讲：- **NMOSD与视神经炎的关系**- **AQP4抗体阳性意味着什么**- **NMOSD患者怀孕生孩子要注意什么**

**19、NMOSD有什么饮食禁忌？**

NMOSD 也叫视神经脊髓炎谱系疾病。先说结论：**目前没有被高质量医学证据明确证实的“NMOSD专属饮食禁忌”**，也没有哪一种食物被证明会直接诱发或加重 NMOSD。饮食管理的重点是 **均衡营养、避免诱发因素、配合激素和免疫治疗减少并发症**。## 一、总体饮食原则### 1. 没有绝对禁忌，但应避免以下几类- **酒精**- 可能影响睡眠、加重疲劳- 与部分药物联用会增加肝脏负担- **高盐饮食**- 若正在使用激素治疗，容易加重水钠潴留、血压升高、浮肿- **高糖饮食**- 激素治疗期间可能升高血糖，增加体重- **高脂油炸食物**- 不利于体重控制和血脂管理- **生冷、不洁食物**- 免疫抑制治疗期间感染风险更高，应尽量避免- **来路不明的保健品、偏方、草药** - 可能伤肝肾，或与免疫治疗药物相互作用### 2. 建议多吃- **优质蛋白**- 鱼、蛋、奶、瘦肉、豆制品- 有助于组织修复和维持体力- **新鲜蔬菜水果**- 补充维生素、矿物质和膳食纤维- **富含钙和维生素D的食物**- 牛奶、酸奶、低糖奶制品、小鱼虾等- 尤其适合长期用激素者，帮助骨健康- **全谷物和高纤维食物**- 有助于稳定血糖、改善便秘- **充足饮水**- 但如有严重排尿功能障碍，需要结合医生建议调整## 二、如果正在用药，饮食要更注意### 1. 使用糖皮质激素时。常见如甲泼尼龙、泼尼松等，饮食建议- **低盐**- **控糖**- **适量补钙、维生素D**- **增加蛋白质摄入**- 避免暴饮暴食，防止体重快速增加### 2. 使用免疫抑制剂或生物制剂时。如硫唑嘌呤、吗替麦考酚酯、利妥昔单抗等：- 注意 **食品卫生**- 少吃生食，如生鱼片、未熟鸡蛋、未经消毒奶制品- 避免感染性腹泻- 不要自行服用“增强免疫力”类保健品## 三、NMOSD患者常见误区### 误区1：必须严格忌口发物**没有明确证据**说明所谓“发物”会导致 NMOSD 复发。若某种食物你本人每次吃后都明显不适，可以个体化回避，但没必要盲目大范围忌口。### 误区2：必须吃某种特殊饮食才能控制病情。目前没有充分证据证明：- 纯素饮食- 生酮饮食- 无麸质饮食- 某种抗炎饮食。能替代正规治疗控制 NMOSD。**规范治疗仍是关键。**### 误区3：保健品能代替药物。不能。NMOSD 是复发性自身免疫疾病，视神经受累后可影响视力，延误规范治疗风险较大。## 四、如果合并眼科问题，饮食上怎么理解？NMOSD 常累及视神经，出现视力下降、眼痛等。饮食本身**不能直接逆转视神经炎**，但有助于维持整体状态。真正重要的是：- 按时复诊- 规范使用神经科或眼科医生开的药- 一旦出现视力下降、色觉变差、眼球转动痛，应尽快就医## 五、这些情况建议尽快问医生如果你有以下情况，饮食方案需要个体化：- 糖尿病- 高血压- 骨质疏松- 肝功能异常- 肾功能异常- 吞咽困难- 明显便秘或排尿障碍- 正在长期服用激素或免疫抑制剂## 六、一句话总结**NMOSD没有公认的特殊饮食禁忌，重点是低盐、控糖、少酒、注意食品卫生、避免乱用保健品，并根据激素或免疫治疗情况调整饮食。**如果你愿意，我还可以进一步帮你整理一份：- **NMOSD患者一周饮食建议**- **激素治疗期间饮食清单**- **视神经炎发作期生活注意事项**

**20、面对高复发风险和高致残率，我感到非常焦虑和抑郁，该怎么办？**

理解你的感受。NMOSD 这种高复发、高致残风险的疾病，确实很容易让人长期处在“担心下一次发作”的压力里。出现焦虑、抑郁、失眠、反复担心病情恶化，并不说明你“脆弱”，而是很多患者都会经历的正常心理反应。作为医生，我想告诉你：**这件事需要重视，而且是可以干预的。**## 先说最重要的。如果你已经出现以下情况之一，请**尽快就医**，最好当天联系家人陪同去医院的神经内科或精神心理科：- 持续觉得活着没有意义- 有伤害自己或轻生的想法- 明显吃不下、睡不着，持续很多天- 焦虑到心慌、发抖、无法正常生活- 情绪低落持续两周以上且越来越重。这不是“想太多”，而是需要专业帮助的信号。## 为什么 NMOSD 容易带来焦虑和抑郁。常见原因包括：- 害怕复发，担心视力、肢体功能、生活能力受影响- 病程不确定，缺乏掌控感- 长期治疗、复查和经济压力- 激素治疗后可能带来的情绪波动、失眠、烦躁- 疼痛、视力下降、乏力等症状本身也会影响情绪- 社交、工作、家庭角色发生变化。也就是说，你的情绪问题往往不是单一“心理原因”，而是**疾病、生理、治疗和生活压力共同作用**的结果。## 你现在可以怎么做？### 1 先把“复发恐惧”变成“可执行的管理计划”焦虑最怕不确定。把担心转化为行动，通常会明显减轻无助感。你可以和主管医生一起明确这几件事：- 你目前属于哪种复发风险水平- 正在用什么预防复发治疗，效果如何- 需要多久复查一次- 出现哪些症状要立刻就诊- 家里是否需要准备紧急就医方案。建议你写一个个人疾病管理清单：- 当前用药名称、剂量、时间- 复诊时间- 既往发作表现- 预警症状- 主治医生联系方式- 家属联系人。当你知道“如果出现问题，我该怎么办”时，焦虑通常会下降很多。### 2 接受正规心理评估。如果焦虑、抑郁已经持续两周以上，建议尽快到：- 精神心理科- 心理医学科- 神经内科联合心理门诊。医生通常会评估：- 焦虑和抑郁程度- 是否与激素等药物相关- 是否伴有失眠、惊恐、躯体化症状- 是否需要心理治疗、药物治疗或联合干预**心理问题和 NMOSD 并不冲突，越早干预，生活质量越好。**### 3 优先处理睡眠。睡眠不好会明显放大焦虑和抑郁，也会让你对白天症状更敏感。你可以先尝试：- 固定上床和起床时间- 白天适度活动，避免整天卧床- 下午后少喝浓茶、咖啡- 睡前少刷病友负面信息- 睡前做 10 分钟缓慢呼吸或渐进性肌肉放松。如果失眠明显，尤其是在用激素期间，应告诉医生，必要时可以短期干预。### 4 学会识别“灾难化思维”。很多患者会反复想：- “我一定会复发”- “一旦复发就会完全失明或瘫痪”- “以后我什么都做不了了”。这些念头会让大脑持续处于警报状态。你可以试着把想法改成更客观的表达：- “我有复发风险，但我正在接受预防治疗和随访”- “即使出现异常，我也有明确的就医方案”- “疾病会影响生活，但不等于生活全部失控”。这不是自我安慰，而是帮助大脑回到事实层面。### 5 不要一个人扛。建议让至少一位家人知道：- 你的病情特点- 复发预警信号- 你近期的情绪状态- 何时需要陪你去医院。有些患者表面上很平静，实际上内心压力很大。**把情绪说出来，本身就是治疗的一部分。**### 6 适度获取信息，避免信息过载。很多人越焦虑越不停查资料，但碎片化信息容易放大恐惧。建议你：- 只看正规医院、专业医生、权威科普的信息- 固定时间了解疾病，不要全天反复搜索- 少看极端个案，避免拿最坏结果套在自己身上### 7 规律生活，尽量保留“正常感”。即使患病，也要尽量维持基本生活节奏：- 规律吃饭- 适度运动- 保留简单社交- 给自己设定小目标。哪怕只是今天洗澡、散步、按时吃药、完成一次复诊，都是在重建掌控感。## 哪些治疗方式可能会帮助你。医生可能会根据情况建议：### 心理治疗。常用的是：- 认知行为治疗- 支持性心理治疗- 压力管理训练- 家庭支持干预。这类治疗对“复发恐惧、长期担忧、睡眠差、情绪低落”都很有帮助。### 药物治疗。如果焦虑或抑郁达到中度以上，或已经影响睡眠、食欲、治疗配合，医生可能会考虑抗焦虑或抗抑郁药物。是否使用、如何选择，要结合：- 你现在的 NMOSD 治疗方案- 是否正在用激素- 肝肾功能- 睡眠情况- 既往精神心理病史**不要自行买药吃。**## 从眼科角度提醒你。如果你的 NMOSD 曾累及视神经，除了情绪问题外，也要特别注意视觉相关变化。一旦出现以下情况，应尽快就医：- 视力下降- 眼痛，尤其转眼时痛- 视野缺损- 色觉下降- 双眼看东西明显不一样。因为对 NMOSD 来说，**新发视觉症状可能提示视神经受累或复发，需要尽快评估。**## 你可以马上做的 5 件小事A[告诉一位家人你现在很焦虑] --> B[预约神经内科或心理科]B --> C[整理用药和复发记录]C --> D[今晚按时休息少刷负面信息]D --> E[若有轻生念头立即就医]## 我想特别对你说，你现在的焦虑和抑郁，不代表你不坚强，而是你承受了太多不确定性。NMOSD 的管理目标不仅是减少复发、保护神经功能，也包括**保护你的情绪、睡眠和生活质量**。心理状态变差时，主动求助是成熟和负责的表现。如果你愿意，我还可以继续帮你做两件事之一：- 帮你整理一份 **NMOSD 焦虑就诊清单**- 或者帮你做一个 **判断自己是否需要尽快看心理科的简单自测问答**

**Gemini**

1、**如何确诊NMOSD？**

视神经脊髓炎谱系疾病（NMOSD）是一种主要累及视神经和脊髓的中枢神经系统自身免疫性疾病。在临床上，确诊NMOSD是一项严谨的过程，主要依据2015年国际视神经脊髓炎诊断小组（IPND）制定的标准。作为医师，我们通常会通过以下几个步骤和检查来为您明确诊断：**1. 临床症状评估**我们会首先详细询问您的病史，寻找NMOSD的“核心临床特征”。最常见的三大核心症状包括：***视神经炎：** 表现为单眼或双眼视力急剧下降、视野缺损，常伴有眼球转动时的疼痛。***急性脊髓炎：** 表现为肢体无力、麻木、感觉异常，以及尿潴留或大便失禁等括约肌功能障碍。***极后区综合征：** 表现为找不到其他原因的、顽固性的打嗝、恶心和呕吐。**2. 核心化验：AQP4-IgG抗体检测**这是确诊NMOSD最关键的步骤。我们会抽取您的血液或脑脊液，检测**水通道蛋白4抗体（AQP4-IgG）**。**如果AQP4抗体呈阳性：** 只要您具备至少1个核心临床症状，并且排除了其他可能的疾病，我们就可以为您确诊NMOSD。**如果AQP4抗体呈阴性（或无法检测）：** 诊断要求会更加严格。您需要经历过至少两次临床发作，具备至少2个不同的核心临床症状（其中必须包含视神经炎、脊髓炎或极后区综合征中的一种），并且MRI影像学表现必须符合特定要求。**3. 关键辅助检查**为了确认病变部位并排除其他疾病（如多发性硬化症），您需要进行以下检查：***核磁共振成像 (MRI)：**脊髓MRI：** NMOSD的一个典型特征是“纵向延伸的长节段横贯性脊髓炎（LETM）”，即脊髓病灶长度通常超过3个甚至更多椎体节段。***视神经和脑部MRI：** 观察视神经是否有炎症信号，以及脑部是否有非典型的脱髓鞘病灶。**腰椎穿刺（脑脊液检查）：** 用于检测脑脊液中的白细胞计数、蛋白质水平以及“寡克隆区带（OCB）”。NMOSD患者的脑脊液OCB通常为阴性，这有助于将其与多发性硬化（MS）区分开来。**眼科专科检查：****光学相干断层扫描 (OCT)：** 用于精确测量视网膜神经纤维层（RNFL）和神经节细胞层（GCIPL）的厚度，评估视神经受损的程度。***视觉诱发电位 (VEP)：** 评估视神经传导视觉信号的速度和完整性。**其他血液免疫学检查：** NMOSD患者常伴随其他自身免疫疾病。我们会检测抗核抗体（ANA）、干燥综合征抗体（SSA/SSB）、甲状腺自身抗体等，以排除或确认是否合并系统性红斑狼疮、干燥综合征等疾病。此外，有时也会检测MOG抗体，以排除MOG抗体相关疾病（MOGAD）。**就诊建议：**NMOSD虽然首发症状常在眼科（视力下降），但它本质上属于神经免疫性疾病。如果您怀疑自己患有此病，建议您尽快前往大型综合医院的**神经内科（脱髓鞘疾病专科）**或**神经眼科**就诊，尽早完善AQP4抗体和MRI检查。早期确诊并启动免疫治疗对于挽救视力和预防复发至关重要。

**2、NMOSD与多发性硬化有什么区别？**

NMOSD（视神经脊髓炎谱系疾病）和多发性硬化（MS）虽然都是中枢神经系统的自身免疫性疾病，且常常都以视神经炎（视力下降、眼球转动痛）为首发症状，但它们是两种完全独立的疾病。作为医生，我们在临床上必须严格区分这两者，因为它们的治疗方案截然不同，误诊可能会导致病情恶化。以下是它们的主要区别：**1. 发病机制与攻击靶点不同***	**NMOSD：** 主要是一种“星形胶质细胞”疾病。患者体内的免疫系统错误地产生了针对水通道蛋白4（AQP4）的抗体，主要攻击视神经和脊髓。*	**MS：** 是一种“脱髓鞘”疾病。免疫系统主要攻击包裹在神经纤维外层的髓鞘（类似于电线的绝缘皮），病变可广泛分布于大脑、视神经和脊髓。**2. 临床症状与严重程度不同***	**视神经受累：** NMOSD引起的视神经炎通常非常严重，可能导致迅速的重度视力下降甚至失明，且双眼同时受累的概率较高。MS引起的视神经炎相对较轻，多为单眼发病，视力恢复通常较好。*	**脊髓受累：** NMOSD常引起纵向延伸的横贯性脊髓炎（病变长度通常跨越3个或以上脊椎节段），导致严重的截瘫、大小便失禁。MS的脊髓病变通常较短（小于2个节段），症状相对较轻或呈非对称性。*	**特殊症状：** NMOSD患者常出现顽固性呃逆（打嗝）、恶心和呕吐（延髓极后区综合征），这在MS中非常罕见。MS患者则更容易出现认知功能下降、疲劳和共济失调（走路不稳）。**3. 辅助检查结果不同***	**特异性抗体：** 约70%-80%的NMOSD患者血液中可检测出**AQP4抗体**阳性，这是确诊NMOSD的“金标准”。而MS患者该抗体为阴性。*	**脑脊液检查：** MS患者的脑脊液中通常存在**寡克隆区带（OCB）**（阳性率>85%），而NMOSD患者通常为阴性或仅在急性期短暂出现。*	**核磁共振（MRI）：** NMOSD的脊髓病变长且连续，脑部病变初期较少；MS的脊髓病变短且分散，脑部（尤其是脑室周围）常有典型的多发性脱髓鞘斑块。**4. 治疗方案与预后截然不同（最重要的一点）***	**治疗禁忌：** 许多用于治疗MS的疾病修饰药物（如干扰素、芬戈莫德、那他珠单抗等），如果误用于NMOSD患者，**不仅无效，反而会诱发NMOSD的严重复发**。*	**维持治疗：** NMOSD的预防复发主要依赖传统免疫抑制剂（如硫唑嘌呤、吗替麦考酚酯）或靶向生物制剂（如利妥昔单抗、依库珠单抗、萨特利珠单抗等）。MS则使用专门的疾病修饰治疗（DMT）药物。*	**预后：** NMOSD的致残率主要来自于每一次复发的累积（发作后恢复较差），因此治疗核心是“绝对预防复发”。MS在早期多为复发-缓解型，发作后恢复较好，但后期可能转为进展型。如果您或您的家属正在经历相关症状，建议尽快完善AQP4抗体、MOG抗体以及脑脊液和全中枢神经系统MRI检查，以便尽早明确诊断并开始针对性治疗。

**3、AQP4抗体阴性是否能诊断为NMOSD？**

您好。作为医生，我可以直接地回答您：**AQP4（水通道蛋白4）抗体阴性，依然是可以诊断为NMOSD（视神经脊髓炎谱系疾病）的**。但是，AQP4抗体阴性患者的诊断标准，要比阳性患者**严格得多**。为了让您更好地理解，我为您做以下详细拆解：**1. AQP4抗体在诊断中的地位**AQP4抗体是NMOSD的“特异性标志物”。大约70%~80%的NMOSD患者该抗体呈阳性。如果患者AQP4抗体阳性，通常只需要有一次典型的临床发作（比如视神经炎导致的视力骤降，或者脊髓炎导致的肢体麻木无力），就可以确诊为NMOSD。**2. AQP4抗体阴性时的确诊条件*如果您的AQP4抗体检测为阴性，根据2015年国际NMO诊断小组（IPND）制定的标准，医生必须找到更多的“证据”来拼凑出完整的诊断，以防误诊。具体需要满足以下条件：*	**多部位受累（空间多发性）**：患者必须经历过至少**两次**核心临床症状的发作，并且这两个症状不能是完全一样的。比如，不能仅仅是两次视神经炎，必须是“视神经炎 + 脊髓炎”，或者“视神经炎 + 极后区综合征（表现为不明原因的顽固性打嗝、恶心、呕吐）”等组合。*	**严格的影像学（MRI）证据**：核磁共振检查必须显示出高度符合NMOSD特征的病灶。例如，如果是脊髓炎，核磁共振上的病灶长度必须跨越至少3个连续的椎体段（这被称为长节段横贯性脊髓炎，LETM）。*	**严格排除其他疾病**：这是阴性确诊中最关键的一环。**3. 医生给您的重要建议（下一步该怎么做）**如果您目前表现出类似NMOSD的症状（如视神经炎），但AQP4抗体是阴性，建议您和您的主治医生确认以下几点：*	**检测方法是否最敏感？** 目前医学界公认最准确的AQP4抗体检测方法是**CBA法（基于细胞底物的检测法）**。如果之前使用的是ELISA或TBA法，可能会出现假阴性，建议用CBA法复查一次。*	**是否检测了MOG抗体？** 这一点非常重要。很多过去被认为是“AQP4阴性NMOSD”的患者，后来被发现**MOG（髓鞘少突胶质细胞糖蛋白）抗体**呈阳性。现在医学界已经将这类情况独立出来，称为MOG抗体相关疾病（MOGAD）。虽然它的症状和NMOSD很像，但未来的复发风险、预后和部分治疗用药是有区别的。*	**是否排除了多发性硬化（MS）？** 多发性硬化也是一种常见的中枢神经系统脱髓鞘疾病，同样会引起视神经炎，需要通过脑部和脊髓的MRI特征以及脑脊液检查（如寡克隆区带OCB）来严格鉴别。**总结来说：** AQP4阴性不能排除NMOSD，但需要神经眼科或神经内科（尤其是擅长神经免疫/脱髓鞘疾病的专科医生）结合您的病史、详细的核磁共振片子以及全面的血液/脑脊液化验结果，进行非常谨慎的综合判断。建议您携带所有的检查报告和影像学资料，前往专业的神经免疫专科进行评估。

4、**NMOSD急性期的治疗方案有哪些？**

NMOSD（视神经脊髓炎谱系疾病）的急性期发作往往来势凶猛，主要表现为严重的视力下降、肢体无力或感觉异常等。急性期治疗的核心目标是尽快控制全身和局部的炎症反应、挽救神经功能、尽量减少后遗症。针对急性期，临床上主要有以下几种标准的治疗方案，我们会根据您的具体病情严重程度和身体状况进行选择：**1. 大剂量糖皮质激素冲击治疗（一线首选方案）***	**治疗方式**：这是最常用的首选方法。通常使用甲泼尼龙静脉滴注（通常为每天1克，连续使用3到5天）。之后，会根据您的神经功能恢复情况，逐渐减少剂量，并过渡到口服激素（如醋酸泼尼松）维持一段时间。*	**作用**：迅速抑制体内的急性免疫炎症，减轻视神经和脊髓的水肿。*	**配合治疗**：大剂量激素可能会引起血糖升高、血压波动、失眠、胃黏膜损伤等副作用。因此，在输液期间，我们会同时给您开具护胃药、补钾、补钙药物，并密切监测您的血糖和血压。**2. 血浆置换（PLEX）或免疫吸附（IA）***	**适用情况**：如果您对上述的激素冲击治疗反应不佳，或者本次发作非常严重（例如导致了重度瘫痪，或视力急剧下降至接近失明），我们会建议尽早（通常在发病几天内）启动血浆置换。*	**治疗方式**：NMOSD的发病与血液中的致病抗体（尤其是AQP4抗体）密切相关。血浆置换相当于给血液做一次“透析”，通过机器直接将血液中的有害抗体和炎性物质清除出去。一般需要隔日进行一次，共进行5到7次。**3. 静脉注射免疫球蛋白（IVIG）***	**适用情况**：通常作为激素或血浆置换的替代或辅助方案。如果患者存在严重的感染风险、有血浆置换的禁忌症，或者对前两种治疗效果都不理想时，我们会考虑使用。*	**治疗方式**：连续5天静脉输注免疫球蛋白，通过中和体内的有害抗体来调节免疫系统。**4. 对症与支持治疗***	在急性期，您可能会经历剧烈的神经痛或痛性痉挛。我们会给您使用加巴喷丁、卡马西平或巴氯芬等药物来缓解疼痛和肌肉痉挛。*	如果您暂时需要卧床，我们会指导您进行肢体被动活动，以预防下肢深静脉血栓和肺部感染。**医师的特别叮嘱：**急性期的“灭火”治疗只是第一步。NMOSD是一种复发率较高的疾病，每一次复发都可能带来不可逆的神经损伤。因此，在急性期症状初步稳定后，**必须尽早过渡到长期的预防复发治疗（即缓解期治疗）**。这通常需要长期使用免疫抑制剂（如硫唑嘌呤、吗替麦考酚酯）或靶向生物制剂（如利妥昔单抗、萨特利珠单抗、伊奈利珠单抗等）。请您保持平稳的心态，积极配合当前的治疗。我们会根据您每天的症状变化和复查指标，随时调整最适合您的个体化方案。用药期间如果有任何不适（如胃痛、心慌等），请随时呼叫医护人员。

**5、为什么NMOSD会出现顽固性呃逆、恶心和呕吐？**

NMOSD（视神经脊髓炎谱系疾病）患者出现顽固性呃逆、恶心和呕吐，在医学上被称为**极后区综合征（Area Postrema Syndrome）**。这是该疾病非常典型且核心的症状之一，其根本原因在于免疫系统对大脑特定区域的异常攻击。具体原因可以归结为以下几个关键点：1. **病变部位（延髓极后区）：** 极后区位于大脑的脑干（延髓）部位。这个区域包含了人体的“呕吐中枢”，专门负责控制和调节恶心、呕吐以及呃逆（打嗝）等生理反射。2. **靶点高度集中（AQP4蛋白）：** 大多数NMOSD是由体内产生的一种异常自身抗体引起的，即水通道蛋白4抗体（AQP4-IgG）。而在延髓极后区，恰好密集分布着大量的AQP4蛋白。3. **血脑屏障薄弱：** 大脑的大部分区域都有“血脑屏障”保护，阻止血液中的有害物质进入脑组织。但极后区是一个特殊的结构，它缺乏紧密的血脑屏障。这使得血液中循环的AQP4抗体能够非常容易地“长驱直入”到达这里。4. **免疫炎症反应：** 当抗体进入极后区后，会与这里的AQP4蛋白结合，引发强烈的自身免疫炎症反应，导致呕吐中枢受到持续的异常刺激。因为这种恶心和呕吐是由中枢神经系统的炎症引起的，而不是胃肠道本身的病变，所以普通的止吐药或胃药往往没有效果，表现为“顽固性”。很多患者在出现视力下降或脊髓损伤症状之前，会首发这些症状，因此常在消化内科被误诊。如果您需要了解关于如何通过药物（如激素冲击或其他免疫抑制治疗）来缓解这些症状，或者关于NMOSD的长期管理方案，我可以为您进一步详细说明。

**6、如何有效预防NMOSD复发？**

视神经脊髓炎谱系疾病（NMOSD）具有高复发性，且每次复发造成的神经损伤（如视力下降、肢体瘫痪）往往是累积且不可逆的。因此，预防复发是NMOSD治疗的核心。有效预防复发需要通过规范的药物治疗和科学的日常管理相结合：**1. 坚持长期的免疫维持治疗（核心）**NMOSD患者在急性期症状控制后，必须尽早开始长期的免疫维持治疗。目前主要有两类药物：*	**传统免疫抑制剂：** 如利妥昔单抗（Rituximab）、吗替麦考酚酯（MMF，骁悉）、硫唑嘌呤（Azathioprine）。其中，利妥昔单抗通过靶向清除B细胞，在临床上预防复发的效果非常显著，是目前广泛使用的一线治疗方案。*	**新型生物靶向制剂：** 近年来获批的针对NMOSD特定发病机制的药物，如依库珠单抗（Eculizumab，补体C5抑制剂）、伊奈利珠单抗（Inebilizumab，CD19单抗）和萨特利珠单抗（Satralizumab，IL-6受体单抗）。这些药物能极大地降低复发率，通常用于对传统药物反应不佳或有条件的患者。**2. 严格遵医嘱，切勿自行停药**口服糖皮质激素或免疫抑制剂的减量必须在医生指导下缓慢进行。自行停药、漏服或快速减量是导致NMOSD复发的最常见原因。**3. 定期随访与指标监测***	定期复查血液指标，包括血常规、肝肾功能（监测药物副作用）、B细胞亚群（评估利妥昔单抗或伊奈利珠单抗的疗效）以及AQP4抗体滴度。*	定期进行眼科检查（如OCT视网膜断层扫描、视野检查、视神经视觉诱发电位 VEP）和头颅/脊髓MRI检查，以便在出现亚临床病灶时及早干预。**4. 预防感染与疫苗管理**感冒、肠道感染等引发的免疫系统激活是诱发NMOSD复发的常见诱因。日常应注意个人卫生，流感季节去人群密集场所建议佩戴口罩。接种任何疫苗前必须咨询您的主治医师，因为某些减毒活疫苗可能存在引发免疫反应的风险，且在使用B细胞耗竭剂（如利妥昔单抗）期间，疫苗的保护效果可能会大打折扣。**5. 避免使用多发性硬化（MS）的治疗药物**NMOSD曾被认为是多发性硬化的一种亚型，但两者发病机制完全不同。必须明确诊断，因为某些用于治疗MS的疾病修饰药物（如干扰素-β、芬戈莫德、那他珠单抗等）不仅对NMOSD无效，反而会加重病情或诱发严重复发。**6. 健康的生活方式**保持规律的作息，避免熬夜、过度劳累和精神高度紧张；适当补充维生素D；戒烟限酒。情绪波动和长期的精神压力也会影响免疫系统的稳定性。具体的预防方案需要根据您的AQP4抗体状态、既往发作的严重程度、合并症以及对药物的耐受性进行个体化定制。如果您想详细了解某一种具体药物的机制与副作用管理，或者需要制定日常随访的具体时间表，我可以为您进一步解答。

**7、目前NMOSD缓解期的一线预防药物有哪些？**

视神经脊髓炎谱系疾病（NMOSD）具有高复发、高致残的特点，因此缓解期的核心治疗目标是严格预防复发。目前临床上用于NMOSD缓解期的一线预防药物主要分为两大类：新型靶向生物制剂和传统免疫抑制剂。**1. 新型生物制剂（单克隆抗体）**这类药物靶点明确，预防复发的疗效总体优于传统免疫抑制剂，目前在国内外指南中被优先推荐，特别是针对AQP4-IgG抗体阳性的患者：*	**利妥昔单抗（Rituximab）**：靶向CD20的单抗，临床应用经验丰富，能有效清除B细胞，显著降低复发率。*	**萨特珠单抗（Satralizumab）**：靶向白介素-6（IL-6）受体的单抗，可皮下注射，患者依从性较好。*	**伊奈利珠单抗（Inebilizumab）**：靶向CD19的单抗，能更广泛地耗竭B细胞及其谱系。*	**依库珠单抗（Eculizumab）**：靶向补体C5的单抗，起效快，对控制重症复发效果显著。**2. 传统免疫抑制剂**在生物制剂不可及、存在禁忌症或经济条件受限的情况下，传统经典免疫抑制剂仍是一线治疗的重要基石：*	**吗替麦考酚酯（MMF）**：耐受性相对较好，是目前最常用的口服一线免疫抑制剂之一。*	**硫唑嘌呤（Azathioprine）**：经典的免疫抑制剂，但起效较慢（通常需要数月），且需密切监测肝功能和骨髓抑制等副作用（用药前建议筛查NUDT15基因）。具体用药方案的制定需要由专业医师结合患者的AQP4抗体状态（阳性或阴性）、既往发作严重程度、经济条件、生育意愿以及对药物的耐受性进行个体化评估。如果您需要了解某款具体药物的给药方式、起效时间、常见副作用或用药期间的监测指标，我可以进一步为您详细说明。

**8、新型的单抗类药物与传统免疫抑制剂相比，在治疗NMOSD中各自的优缺点是什么？**

你好。作为一名医生，我非常理解你对视神经脊髓炎谱系疾病（NMOSD）治疗方案的关注。NMOSD 是一种高复发、高致残性的自身免疫性疾病，因此预防复发是治疗的核心。目前，治疗药物已从传统的“经验性”免疫抑制剂迈向了“精准化”的单克隆抗体时代。以下是这两类药物在临床应用中的详细优缺点对比：### 1. 传统免疫抑制剂：这类药物主要包括**硫唑嘌呤（AZA）、支原酚酯（MMF，也称骁悉）、甲氨蝶呤（MTX）**等。**优点：***	**临床经验丰富：** 医生对其长期使用的副作用和疗效有长达数十年的观察。*	**价格低廉：** 医保报销比例高，患者经济负担较轻。*	**给药方便：** 大多为口服剂型，不需要频繁去医院输液或注射。**缺点：***	**起效较慢：** 通常需要 3-6 个月才能达到稳定的免疫抑制状态，在此“窗口期”内仍有复发风险。*	**非特异性抑制：** 它们是“地毯式轰炸”，会无差别地抑制免疫系统。*	**副作用明显：** 长期使用可能导致骨髓抑制（白细胞减少）、肝肾功能损害、胃肠道反应以及增加恶性肿瘤的潜在风险。*	**复发控制率：** 虽然有效，但仍有相当比例的患者在用药期间出现突破性复发。### 2. 新型单克隆抗体（生物制剂）目前主流的药物包括**依库珠单抗（Eculizumab）、萨特利珠单抗（Satralizumab）、伊奈利珠单抗（Inebilizumab）**，以及虽然属于老一代但在临床广泛应用的**利妥昔单抗（Rituximab）**。**优点：***	**精准打击（靶向性）：** 它们针对 NMOSD 发病机制中的关键环节（如补体系统、白介素-6 受体或 B 细胞）。*	**疗效显著：** 临床试验显示，新型单抗能显著降低 AQP4 受体阳性患者的年复发率（ARR），部分药物可使复发风险降低 70%-90% 以上。*	**起效相对较快：** 相比传统药物，能更快地建立免疫屏障。*	**副作用相对集中：** 因为是靶向治疗，对骨髓和肝肾的毒性较小。**缺点：***	**价格昂贵：** 尽管部分药物已进入医保，但自付费用通常仍高于传统药物。*	**给药方式限制：** 需要定期进行静脉输注或皮下注射，可能需要往返医院。*	**特定感染风险：** 例如，使用补体抑制剂（依库珠单抗）需严格预防脑膜炎双球菌感染；B 细胞耗竭剂可能增加呼吸道感染风险。*	**适用人群限制：** 目前大多数新型单抗主要获批用于 **AQP4-IgG 阳性**的成年患者，对于阴性患者的证据相对较少。### 医生总结与建议| 特性 | 传统免疫抑制剂 | 新型单克隆抗体 || **精准度** | 低（广泛抑制） | 高（靶向治疗） || **复发预防能力** | 中等 | 极高 || **起效速度** | 慢（数月） | 较快 || **安全性监控** | 需频繁查血常规、肝肾功 | 侧重于特定感染预防 || **经济性** | 优 | 较差（但正在改善） |**我的专业建议：**1. **首选评估：** 如果你的 AQP4 抗体检测为阳性，且经济条件允许或有相关医保支持，**新型单抗通常是更优的选择**，因为它们能更有效地保护你的视力和脊髓功能，减少伤残积累。2. **个体化选择：** 如果病情较轻、处于缓解期多年且对传统药物耐受良好，或者存在特定生物制剂禁忌症，传统药物仍是合理的方案。3. **严密监测：** 无论选择哪种方案，都必须在医生指导下定期复查，绝不可擅自停药，因为 NMOSD 的每一次复发都可能带来不可逆的损伤。建议你与主治医生详细沟通你的抗体状态、既往发病频率以及经济承受能力，共同制定最适合你的“个体化”治疗方案。如果你需要了解某种具体药物（如萨特利珠单抗或伊奈利珠单抗）的细节，可以随时询问。

**9、长期使用预防NMOSD复发的药物，主要的副作用有哪些？**

预防视神经脊髓炎谱系疾病（NMOSD）复发的长期用药主要包括传统免疫抑制剂、新型生物制剂以及糖皮质激素。不同药物的作用机制不同，其主要副作用也有所区别：**1. 传统免疫抑制剂（如硫唑嘌呤、吗替麦考酚酯/骁悉）*** **骨髓抑制：** 这是最常见的副作用，表现为白细胞、红细胞或血小板减少，从而增加感染、贫血或出血的风险。* **肝肾功能损伤：** 尤其是硫唑嘌呤，容易引起肝转氨酶升高，引发药物性肝损。* **胃肠道反应：** 恶心、呕吐、食欲减退、腹泻（腹泻在吗替麦考酚酯使用者中较为常见）。* **长期风险：** 长期抑制免疫系统可能轻微增加特定恶性肿瘤的风险。**2. 生物制剂（如利妥昔单抗、伊奈利珠单抗、萨特利珠单抗、依库珠单抗）*** **感染风险增加：** 这类药物通过精准靶向抑制免疫系统（如耗竭B细胞或抑制补体），会使患者更容易发生上呼吸道感染、泌尿系统感染。**特别注意：** 使用依库珠单抗（补体C5抑制剂）的患者感染脑膜炎奈瑟菌的风险显著增加，用药前必须按规定接种脑膜炎球菌疫苗。* **低免疫球蛋白血症：** 长期使用利妥昔单抗等抗B细胞药物，可能导致血液中免疫球蛋白（IgG等）水平持续下降，进一步增加继发感染风险。* **输液/注射反应：** 在静脉输注或皮下注射时，可能出现发热、寒战、皮疹、血压波动等过敏或输液反应。**3. 糖皮质激素（如醋酸泼尼松片，常作为过渡或小剂量维持用药）*** **代谢与内分泌异常：** 体重增加、向心性肥胖（满月脸、水牛背）、血糖升高（类固醇性糖尿病）、血压升高。* **骨与关节系统：** 骨质疏松，长期使用存在股骨头无菌性坏死的风险。* **眼部并发症：** 作为眼科医生需要特别提醒您，长期全身使用激素可能诱发或加重白内障，以及引起激素性青光眼（眼压升高）。* **其他：** 消化性溃疡、失眠、情绪波动、皮肤变薄及多毛等。**日常监测建议：**长期使用上述任何一种药物，都必须定期到医院复查。通常需要监测血常规、肝肾功能、乙肝/结核等潜伏感染指标，以及眼压和骨密度。如果您能提供您目前正在使用的具体药物名称，我可以为您提供更详细的针对性用药指导和随访计划。

**10、在NMOSD用药期间可以接种疫苗吗？**

你好。作为一名眼科医生，我非常理解视神经脊髓炎谱系疾病（NMOSD）患者在面对疫苗接种时的顾虑。NMOSD 是一种自身免疫性疾病，主要累及视神经和脊髓，其治疗通常涉及免疫抑制剂或生物制剂。关于在用药期间能否接种疫苗，不能一概而论，需要根据**疫苗的类型**、**您正在使用的药物**以及**病情的稳定性**来综合判断。以下是基于临床共识的专业建议：### 1. 疫苗类型的区别（最关键）**灭活疫苗、重组蛋白疫苗、mRNA疫苗：**（如：流感灭活疫苗、新冠灭活/重组疫苗、乙肝疫苗、肺炎球菌疫苗等）*	**结论：** 通常是**安全**的。这些疫苗不含活病毒，不会导致感染。*	**注意：** 由于您正在使用免疫抑制药物，疫苗产生的保护性抗体水平可能会比普通人低，即免疫效果可能“打折扣”。*	**减毒活疫苗：**（如：麻疹-风疹-腮腺炎疫苗(MMR)、水痘疫苗、黄热病疫苗、口服轮状病毒疫苗等）*	**结论：** **原则上禁忌**。在接受中高剂量糖皮质激素、免疫抑制剂（如硫唑嘌呤、吗替麦考酚酯）或生物制剂（如利妥昔单抗）治疗期间，接种活疫苗存在病毒复制导致感染的风险。### 2. 常用 NMOSD 药物对接种的影响*	**B细胞耗竭剂（如利妥昔单抗 Rituximab、伊奈利珠单抗 Inebilizumab）：*这类药物会清除产生抗体的B细胞。如果在用药期间接种，疫苗可能完全失效。*	**建议：** 理想情况下，疫苗接种应在下一次输药前 4 周完成，或在输药后 6 个月（待B细胞开始恢复）进行。*	**补体抑制剂（如依库珠单抗 Eculizumab）：**接种**流脑疫苗（脑膜炎球菌疫苗）**是必须的，因为这类药物会增加脑膜炎球菌感染风险。通常要求在首次给药前至少 2 周接种。*	**糖皮质激素与传统免疫抑制剂（如硫唑嘌呤、吗替麦考酚酯）：**通常不影响灭活疫苗的安全性，但会降低免疫应答。### 3. 给您的具体建议。1. **咨询主治医生：** 在接种任何疫苗前，**务必**咨询您的神经内科或眼科主治医生。他们最了解您目前的免疫状态和用药周期。2. **把握接种时机：** *	如果病情处于急性发作期，应推迟接种。*	如果条件允许，建议在启动长期的免疫抑制治疗前 2-4 周，完善必要的疫苗接种（如乙肝、带状疱疹、肺炎疫苗等）。3. **优先选择灭活疫苗：** 如果必须接种（如流感季节或新冠流行期间），请务必选择灭活路径的疫苗。4. **家属接种：** 建议与患者共同居住的家属接种流感等疫苗，形成“保护圈”，减少患者的感染风险。**总结：**NMOSD 患者在用药期间**可以**接种灭活疫苗，但需避开药物作用的峰值期以确保效果；**严禁**接种减毒活疫苗。请问您目前具体正在使用哪种药物？计划接种哪种疫苗？了解这些信息后我可以为您提供更具体的参考。

**11、如何缓解NMOSD引起的长期神经性疼痛、肌肉痉挛或顽固性瘙痒？**

NMOSD（视神经脊髓炎谱系疾病）引起的神经性疼痛、肌肉痉挛和顽固性瘙痒，主要是由脊髓病灶（尤其是颈髓和胸髓）导致的中枢神经传导异常和神经敏化引起的。由于这些症状源于中枢神经系统，常规的消炎止痛药（如布洛芬）或抗过敏止痒药（如氯雷他定）通常无效。在临床上，针对这三大类症状的缓解需要采取以神经调节药物为主的综合治疗方案：### 1. 缓解长期神经性疼痛 (Neuropathic Pain)神经病理性疼痛常表现为烧灼感、针刺感、电击感或束带感。*	**一线药物：** 钙通道调节剂是首选，包括**普瑞巴林 (Pregabalin)** 或 **加巴喷丁 (Gabapentin)**。这类药物通过抑制神经元的异常放电来减轻疼痛。*	**二线/联合用药：** 如果单药效果不佳，常联合使用特定类型的抗抑郁药物（这类药物在低剂量时具有很好的中枢镇痛作用），如**度洛西汀 (Duloxetine)** 或 **阿米替林 (Amitriptyline)**。*	**非药物干预：** 经皮神经电刺激（TENS）、热敷或冷敷（取决于患者对温度的敏感度）、针灸以及正念冥想，可以辅助降低大脑对疼痛信号的感知。### 2. 缓解肌肉痉挛 (Spasticity)脊髓损伤会导致上运动神经元受损，失去对肌肉的抑制作用，从而引发僵硬和痉挛。*	**口服药物：** 中枢性肌肉松弛剂是核心治疗药物。最常用的是 **巴氯芬 (Baclofen)** 和 **替扎尼定 (Tizanidine)**。用药原则是“小剂量起始，缓慢加量”，以避免过度嗜睡或肌肉无力。*	**局部注射：** 如果痉挛局限在特定的几块肌肉，且口服药物副作用过大，可以考虑由专业医师进行 **A型肉毒毒素 (Botulinum toxin A)** 局部肌肉注射，效果通常可维持3-6个月。*	**物理康复：** 每日规律的、温和的被动拉伸训练至关重要。这可以防止肌肉挛缩和关节畸形。温水浴（水疗）也能有效放松紧张的肌肉。### 3. 缓解顽固性瘙痒 (Intractable Pruritus)NMOSD引起的瘙痒属于“中枢性神经性瘙痒”，常见于颈髓病变，患者常会抓挠至皮肤破溃。*	**口服药物：** 治疗神经性疼痛的药物同样适用于中枢性瘙痒。**加巴喷丁** 和 **普瑞巴林** 是最常用的止痒药物。对于部分表现为阵发性剧烈瘙痒的患者，**卡马西平 (Carbamazepine)** 或 **奥卡西平 (Oxcarbazepine)** 可能有奇效。*	**局部处理：** 尽管病因在中枢，但局部使用 **辣椒素软膏 (Capsaicin cream)** 或含有 **利多卡因/丙胺卡因** 的表面麻醉贴剂，可以通过耗竭末梢神经递质或阻断局部感觉输入来暂时缓解症状。冰敷也能通过温度感受器竞争性地抑制瘙痒信号传导。*	**行为干预：** 保持皮肤湿润，穿宽松柔软的棉质衣物。建议修剪指甲或夜间戴手套，防止无意识抓挠导致继发性皮肤感染。### 综合管理建议1. **控制原发病：** 长期症状的控制建立在NMOSD病情稳定的基础上。坚持使用免疫抑制剂或生物制剂（如依库珠单抗、伊奈利珠单抗、萨特利珠单抗等）预防复发，是避免产生新神经损伤的根本。2. **改善睡眠与情绪：** 疼痛和瘙痒极易导致严重的失眠和抑郁/焦虑，而糟糕的心理状态会放大神经系统的敏感度，形成恶性循环。必要时，应由神经内科或精神心理科医生介入，使用改善睡眠和情绪的药物。**医师提示：** 以上提到的神经调节药物均属于处方药，且存在个体差异和潜在的副作用（如头晕、嗜睡、肝肾功能影响）。请务必记录您症状发作的频率、程度以及诱发因素，在您的主治神经内科或眼科医师的指导下制定个性化的用药和滴定方案，切勿自行购药或调整剂量。

**12、NMOSD导致的大小便功能障碍怎么办？**

NMOSD（视神经脊髓炎谱系疾病）导致的大小便功能障碍，主要是由于疾病累及脊髓（横贯性脊髓炎），损伤了大脑与膀胱、肠道括约肌之间传递信号的神经通路。处理这一问题需要结合急性期抗炎、长期的对症处理以及并发症预防。以下是具体的临床处理建议：**1. 急性期病因治疗（针对新发或加重的症状）**如果大小便障碍是近期突然出现或加重的，这通常意味着脊髓炎症正在急性发作。*	**一线治疗：** 需尽快使用大剂量静脉糖皮质激素（如甲泼尼龙）进行冲击治疗，以迅速减轻脊髓水肿和炎症，挽救神经功能。*	**二线治疗：** 若激素效果不佳，可能需要尽早启动血浆置换（PLEX）或静脉注射大剂量免疫球蛋白（IVIG）。**2. 泌尿功能障碍（神经源性膀胱）的对症处理**需要通过“尿动力学检查”和“泌尿系超声”明确是哪种类型的排尿障碍，再对症用药：*	**尿潴留（排不出、排不尽）：** 膀胱逼尿肌无力或括约肌痉挛导致残余尿量过多。首选方案是**清洁间歇导尿（CIC）**，即每天定时使用一次性导尿管排空膀胱。这能有效防止尿液反流导致的肾积水和肾功能衰竭。有时会辅助使用α受体阻滞剂（如坦索罗辛）帮助放松括约肌。*	**尿频、尿急、尿失禁（膀胱过度活动）：** 膀胱极度敏感或不受控制地收缩。通常使用抗胆碱能药物（如索利那新、托特罗定）或β3受体激动剂（如米拉贝隆）来放松膀胱肌肉，增加储尿量。若药物无效，可考虑膀胱逼尿肌内注射肉毒素。**3. 肠道功能障碍（神经源性肠道）的对症处理***	**便秘（最常见）：** 神经受损导致肠蠕动减慢。 *	**生活方式：** 保证充足的水分摄入（每日1.5-2升）和高纤维饮食。建立固定的排便时间（如餐后30分钟利用胃结肠反射）。*	**药物治疗：** 优先使用渗透性泻药（如聚乙二醇、乳果糖），避免长期使用刺激性泻药导致肠道依赖。必要时可使用甘油灌肠剂（开塞露）或进行灌肠。*	**大便失禁：** 括约肌松弛导致。需通过定时排便排空肠道，避免直肠内粪便积聚，同时配合盆底肌训练。**4. 康复训练与长期预防***	**盆底肌训练（凯格尔运动）与生物反馈治疗：** 可以在康复科指导下进行，帮助增强盆底肌肉的控制力。*	**预防复发：** NMOSD极易复发，每一次复发都可能导致脊髓造成不可逆的损伤，加重大小便障碍。必须在神经内科医生指导下坚持使用免疫抑制剂或生物制剂（如利妥昔单抗、萨特利珠单抗、伊奈利珠单抗等）进行长期序贯治疗。**特别提醒：**大小便功能障碍极易引发泌尿系统感染（UTI）。如果您出现发热、尿液浑浊、尿痛或下腹部胀痛，请立即就医进行尿常规和尿培养检查。处理NMOSD的括约肌障碍通常需要神经内科、泌尿外科和康复科的多学科协作。如果您需要了解某一种具体药物的副作用，或者想了解如何进行间歇性导尿和盆底肌训练，我可以进一步为您详细说明。

**13、NMOSD病情稳定后，应该如何进行科学、安全的康复训练？**

你好。NMOSD（视神经脊髓炎谱系疾病）进入病情稳定期，意味着急性期的炎症已经得到控制，此时是进行康复训练、最大程度恢复神经功能和提高生活质量的黄金时期。由于NMOSD主要累及视神经和脊髓，科学、安全的康复训练需要针对视觉功能、肢体运动以及整体体能进行综合干预。以下是具体的康复建议和安全原则：### 一、 视觉功能康复。视神经炎往往会遗留不同程度的视力下降、视野缺损或色觉异常。1. **视觉追踪与刺激训练：** 可以在专业人员指导下，利用高对比度（如黑白相间）的视标或特定的视觉训练软件，进行眼球的转动、注视和追踪训练，以激活残存的视觉神经通路，提高视觉敏锐度。2. **低视力康复：** 如果视力受损较重且已稳定，应前往视光门诊进行评估，验配和学习使用助视器（如光学放大镜、电子助视器、滤光镜等），以满足阅读和日常生活的需要。3. **环境适应与改造：** 改善居家照明条件，避免眩光；在台阶、门框等边缘使用高对比度的颜色标记，以防因视觉障碍导致跌倒。### 二、 脊髓与肢体功能康复。针对横贯性脊髓炎带来的肢体无力、感觉异常或括约肌障碍。1. **关节活动度与肌力训练：** 从被动关节活动开始，防止肌肉萎缩和关节挛缩；随着力量恢复，逐渐过渡到主动的抗阻训练。重点加强核心肌群和下肢力量，以改善步态。2. **平衡与协调训练：** 通过坐位平衡、站立平衡以及使用辅助器具（如助行器、拐杖）进行步态训练，降低跌倒风险。3. **盆底功能训练：** 如果遗留尿频、尿急或排尿困难，可进行凯格尔运动（Kegel exercises）以增强盆底肌力量，必要时可结合生物反馈治疗。### 三、 必须遵循的安全与科学原则。NMOSD患者的神经系统较为脆弱，康复训练必须把“安全”放在首位：1. **避免高温（防范乌托夫现象）：** 许多中枢神经系统脱髓鞘疾病患者在体温升高（如环境炎热、剧烈运动出汗、洗热水澡）时，会出现视力模糊或肢体无力等症状的短暂加重。**训练环境必须保持凉爽、通风，运动量以微微出汗为宜，切忌大汗淋漓。**2. **遵循“疲劳管理”与“能量保存”法则：** 极度疲劳是NMOSD常见的遗留症状。训练必须循序渐进，将每日的活动和训练拆分成多个短时段进行，中间充分休息。**不要等到精疲力竭才停止，要在感觉轻微疲劳时就休息。**3. **保护感觉障碍区域：** 如果肢体存在麻木或痛温觉减退，在日常活动和训练中要特别注意防烫伤、防冻伤、防摩擦破损，因为这些区域受伤后极难愈合且容易引发感染。4. **专业评估先行：** 每个患者的受损部位和程度不同。在开始任何系统的康复计划前，务必由专业的康复科医师和治疗师（PT/OT）进行全面的基线评估，制定个性化的处方，切忌盲目照搬他人的锻炼方式。最后提醒您，康复训练是辅助手段，**按时服用预防复发的免疫抑制药物、定期复查神经眼科和神经内科**，才是保持病情稳定的根本。如果您需要了解针对某种特定症状（如神经痛缓解或具体的眼球运动方法）的详细训练步骤，我可以为您进一步解答。

**14、NMOSD能治愈吗？**

目前，视神经脊髓炎谱系疾病（NMOSD）还无法被彻底治愈，但它是一种高度可治、可控的疾病。作为一种由免疫系统错误攻击自身中枢神经系统（主要是视神经和脊髓）引起的慢性自身免疫性疾病，NMOSD的治疗目标并不在于“断根”，而在于“长期控制并预防复发”。在临床上，我们的治疗主要分为两个关键部分：1. **急性期治疗：** 在疾病突然发作，导致视力急剧下降或肢体麻木无力时，我们会迅速采取措施（如大剂量糖皮质激素冲击治疗、血浆置换或静脉注射免疫球蛋白）来减轻神经炎症，最大程度挽救视力和神经功能。2. **缓解期（维持）治疗：** 这是NMOSD治疗的重中之重。NMOSD具有较高的复发率，而每一次复发都可能累积不可逆的神经损伤（如永久性视力损害或瘫痪）。因此，患者需要长期规范地使用免疫抑制剂（如利妥昔单抗、吗替麦考酚酯、硫唑嘌呤等）或新型靶向生物制剂（如萨特利珠单抗、伊奈利珠单抗、依库珠单抗等）来抑制异常的免疫反应，将复发风险降到最低。虽然医学上还无法将其完全消除，但随着近年来新型药物（尤其是生物制剂）的广泛应用，NMOSD的预后已经得到了极大的改善。只要严格遵医嘱坚持长期用药、定期复查，许多患者可以实现长期“零复发”（即临床缓解状态），并保持正常的工作和生活质量。面对NMOSD，最忌讳的是在症状好转后自行减药或停药，这往往会导致严重的复发。如果您希望了解针对您当前抗体状态（如AQP4抗体是否阳性）和具体病情的个性化用药方案，我们可以进一步详细探讨。

**15、NMOSD的长期预后怎么样？**

视神经脊髓炎谱系疾病（NMOSD）的长期预后存在显著的个体差异，总体而言，**预后主要取决于疾病的复发频率、每次急性发作的严重程度以及对预防性治疗的反应**。作为一种主要累及视神经和脊髓的中枢神经系统自身免疫性疾病，NMOSD的特点是高复发率和高致残率。以下是关于其长期预后的几个核心专业评估：**1. 损伤的累积性**与多发性硬化（MS）不同，NMOSD在两次发作之间的缓解期通常不会出现隐匿性的神经功能恶化。患者的残疾（如视力下降、失明、肢体无力、截瘫、大小便失禁等）几乎完全是由**急性发作造成的不可逆神经损伤累积**导致的。因此，每一次复发都可能对长期预后造成重大打击。**2. 现代医学极大地改善了预后**在过去，NMOSD的长期预后较差，患者往往在确诊后的几年内出现严重的视力或运动功能丧失，高位颈髓发作甚至可能因呼吸衰竭危及生命。然而，近年来随着医学的发展，NMOSD的预后已经得到了**革命性的改善**。通过早期诊断和积极的长期免疫抑制治疗，我们现在有能力极大地降低甚至完全阻止复发。**3. 维持治疗决定长期走向**如果不接受长期的免疫抑制治疗，超过90%的患者会经历复发。目前临床上广泛使用的药物包括：*	**传统免疫抑制剂**：如利妥昔单抗（Rituximab）、吗替麦考酚酯（MMF）、硫唑嘌呤等。*	**新型靶向生物制剂**：如依库珠单抗（Eculizumab）、萨特利珠单抗（Satralizumab）、伊奈利珠单抗（Inebilizumab）等。只要患者对药物敏感并严格依从医嘱，许多人可以实现数年甚至十几年以上的“零复发”，从而像健康人一样正常生活和工作。**4. 抗体状态的影响***	**AQP4-IgG阳性**：这类患者复发风险极高，几乎必须终身接受免疫抑制治疗来维持良好的预后。*	**MOG抗体阳性（现多独立为MOGAD）或双阴性**：这部分患者的复发率和预后特征可能与AQP4阳性患者有所不同，部分MOGAD患者的恢复情况相对较好，但同样需要密切监测。**医师建议：**要获得良好的长期预后，最关键的一点是**绝对依从长期的预防性免疫治疗，切忌在病情稳定时自行减药或停药**。即使感觉完全恢复，潜在的免疫异常依然存在。定期进行门诊随访、复查核磁共振（MRI）和血液指标，有助于医师及时调整治疗方案，最大程度保护您的神经功能。如果您希望了解基于您个人具体病史（如发作次数、当前神经功能评分、抗体状态等）的详细预后评估，可以提供更多临床信息。

**16、NMOSD患者可以怀孕生子吗？**

NMOSD（视神经脊髓炎谱系疾病）患者是可以怀孕生子的，但由于这是一种好发于育龄期女性的中枢神经系统自身免疫性疾病，妊娠和生育过程不能“顺其自然”，而是需要极其谨慎的“计划妊娠”和严格的医疗干预。作为医师，我需要向您强调以下几个核心评估和管理要点：1. **病情稳定是备孕的前提**：通常强烈建议患者在病情完全稳定、无复发至少6到12个月以上再考虑备孕。如果在疾病活动期怀孕，不仅可能加重病情，母婴面临的风险也会显著增加。2. **孕前药物调整至关重要**：许多用于控制NMOSD的常规免疫抑制剂（如吗替麦考酚酯、甲氨蝶呤、环磷酰胺等）具有明确的致畸性，必须在备孕前几个月（甚至半年）停用并彻底洗脱。部分药物（如小剂量糖皮质激素、硫唑嘌呤，或在特定时机评估后使用的利妥昔单抗等）可能被作为替代方案。药物调整必须在神经内科或眼科专科医生指导下进行，切勿自行停药以免引发严重复发。3. **警惕产后复发的高风险**：临床数据表明，孕期由于体内激素和免疫状态的改变，NMOSD的复发率可能相对平稳，但在**产后前3到6个月内，病情复发的风险会急剧上升**。严重的复发可能导致不可逆的视力损伤或截瘫。因此，产后通常需要立即恢复或强化免疫治疗，这也会直接影响到患者是否能够进行母乳喂养。4. **妊娠期并发症监测**：NMOSD患者（尤其是AQP4抗体阳性者）在孕期发生流产、早产或先兆子痫的风险可能略高于健康人群。因此，怀孕后必须在产科作为高危妊娠建档，进行更频密的产检。5. **多学科联合管理**：整个备孕、妊娠、分娩和产后阶段，需要神经眼科/神经内科医生与产科医生密切配合，共同制定个性化的治疗、分娩和随访方案。总而言之，通过科学的规划和多学科的严密监测，许多NMOSD患者都成功孕育了健康的宝宝。您是否需要我进一步详细说明您目前正在使用的特定药物在孕期的安全性，或者产后如何预防复发及母乳喂养的相关建议？

**17、孕期和产后如何管理疾病，以降低NMOSD复发风险？**

作为处理视神经脊髓炎谱系疾病（NMOSD）的医生，我非常理解你对孕期和产后疾病复发的担忧。NMOSD患者完全可以拥有健康的宝宝，但由于妊娠期和尤其是产后是免疫状态剧烈波动的时期，确实需要严格的医学管理来降低复发风险。以下是孕期和产后管理NMOSD的核心策略：**1. 孕期管理：维持稳定，调整用药***	**药物调整**：孕期并非绝对不能用药。如果停药导致严重复发，对母婴的风险更大。必须停用致畸药物（如吗替麦考酚酯、甲氨蝶呤、环磷酰胺），这些通常在备孕前就应完成替换。孕期相对安全的维持药物包括硫唑嘌呤和低剂量糖皮质激素。对于单克隆抗体（如利妥昔单抗、依库珠单抗等新型生物制剂），需由医生根据你的具体病情、既往复发频率严格评估利弊后，决定是否在孕期继续使用或调整用药间隔。*	**密切随访**：整个孕期需要神经内科、眼科和产科（高危妊娠专科）进行多学科联合随访，定期复查视力、视野、神经系统体征及相关抗体滴度。*	**急性发作的处理**：如果在孕期出现视力下降、眼球转动痛或肢体麻木无力等疑似复发症状，必须立即就医。孕期急性期治疗通常首选大剂量甲泼尼龙冲击治疗，必要时可安全使用静脉注射免疫球蛋白（IVIG）或血浆置换（PLEX）。**2. 产后管理：高度警惕，尽早干预（复发极高危期）***	**产后是复发的最危险期**：由于分娩后母体免疫耐受状态突然解除，免疫系统会出现强烈的“反弹”，产后前3至6个月是NMOSD发生严重复发风险最高的阶段。*	**立即恢复或强化免疫抑制治疗**：这是降低产后复发风险的最关键步骤。通常建议在分娩后（甚至产后24-48小时内）立即恢复、维持或启动足量的免疫抑制治疗或靶向生物制剂治疗。对于极高危患者，医生可能会建议在产后早期预防性使用静脉注射免疫球蛋白（IVIG）。*	**哺乳与用药的权衡**：母乳喂养本身通常不会增加复发风险，但你的维持治疗方案决定了是否可以安全哺乳。例如，中低剂量的糖皮质激素和硫唑嘌呤在哺乳期通常被认为是相对安全的（服药后间隔4小时以上再哺乳可减少婴儿摄入）；但如果需要使用某些特定的免疫抑制剂，可能需要放弃母乳喂养。**切勿为了坚持哺乳而自行停用或减少控制NMOSD的药物**，产后复发对视神经和脊髓的打击往往是不可逆的。**3. 日常生活与自我监测***	**预防感染**：感染是诱发NMOSD复发的常见触发因素。孕期和产后需特别注意防寒保暖、饮食卫生，避免接触呼吸道或肠道感染者。*	**保证休息与情绪稳定**：产后疲劳、睡眠剥夺和激素骤降易导致情绪波动，家属的全面照料支持至关重要。过度劳累会显著增加免疫系统紊乱的风险。*	**症状自查**：学会识别复发先兆，如单眼或双眼疼痛、视力模糊、色觉异常、顽固性呃逆/恶心呕吐、肢体麻木或无力、排便排尿障碍等。一旦出现上述任何症状，立刻前往急诊或专科就诊，争取在第一时间开始干预。请注意，每个NMOSD患者的抗体状态（AQP4阳性或阴性）、复发史和对药物的反应都不尽相同。上述建议不能替代面对面的个体化诊疗。请务必在你的神经内科/眼科主治医生以及产科医生的共同指导下，制定一份专属的妊娠与产后管理计划。需要我针对你目前正在使用的某种具体药物，为你做进一步的孕产期安全性解读吗？

**18、NMOSD会遗传给下一代吗？**

视神经脊髓炎谱系疾病（NMOSD）不是一种传统意义上的遗传病，通常不会直接遗传给下一代。绝大多数NMOSD病例都是散发性的。作为一种自身免疫性疾病，NMOSD的发病机制是机体免疫系统错误地攻击了中枢神经系统（主要是视神经和脊髓），这与环境因素、感染以及免疫系统失调有关，而不是由特定的单一基因突变引起的。在医学上，我们需要区分“遗传病”和“遗传易感性”：1. **非遗传病**：NMOSD不遵循孟德尔遗传规律，不会像某些经典遗传病（如血友病、白化病）那样有明确的代际传递概率。2. **遗传易感性**：与许多自身免疫性疾病一样，NMOSD可能存在一定的遗传易感性（例如与某些HLA基因型相关）。这意味着患者的子女在基因层面可能比普通人更容易受到某些触发因素的影响，但这种风险增加的幅度非常小。即使携带了易感基因，如果没有后天环境因素的触发，也不会发病。总体而言，NMOSD患者的下一代患上该病的概率极低，患者完全可以生育健康的后代。如果您或您的家属有生育计划，主要需要关注的不是遗传问题，而是**妊娠期和产后的病情管理**。建议在病情稳定期备孕，并提前与您的神经内科医生和产科医生充分沟通，评估当前使用的免疫抑制剂或激素是否对胎儿安全，并在医生指导下调整用药方案。

**19、NMOSD有什么饮食禁忌？**

视神经脊髓炎谱系疾病（NMOSD）是一种主要累及视神经和脊髓的自身免疫性中枢神经系统疾病。从医学角度来看，NMOSD本身并没有某种绝对会导致疾病复发的“单一毒药型”食物，但由于该病的自身免疫机制，以及患者通常需要长期使用**糖皮质激素**和**免疫抑制剂**（或生物制剂），饮食管理对于减轻药物副作用、预防并发症和维持免疫平衡至关重要。作为医师，我建议NMOSD患者在饮食上注意以下几大类“禁忌”或限制：### 1. 针对糖皮质激素副作用的饮食禁忌：激素（如泼尼松、甲泼尼龙）是治疗NMOSD的常用药物，长期使用会带来一系列代谢问题，因此需严格忌口：*	**忌高盐饮食：** 激素会导致水钠潴留，引起水肿和高血压。应尽量做到低钠饮食（每日食盐不超过5克），**禁忌**腌制食品（咸菜、腊肉）、加工肉类（火腿肠、培根）以及高钠调味料。*	**忌高糖饮食：** 激素会引起糖耐量异常，增加类固醇性糖尿病的风险。**禁忌**各种含糖饮料、甜点、糖果，主食应减少精白米面的比例，增加全谷物和粗粮，以平稳血糖。*	**忌高脂高胆固醇：** 激素易引起脂代谢紊乱和向心性肥胖（满月脸、水牛背）。应**限制**动物内脏、肥肉、黄油及油炸食品，避免反式脂肪酸。*	**注意补钙和钾：** 激素会加速钙流失并增加钾的排泄。饮食中不能缺乏富含钙（牛奶、豆制品）和钾（香蕉、菠菜、土豆）的食物，以预防骨质疏松和低钾血症。### 2. 针对免疫抑制状态的饮食禁忌。服用免疫抑制剂（如硫唑嘌呤、吗替麦考酚酯）或接受生物制剂（如利妥昔单抗、萨特利珠单抗等）治疗期间，患者的抵抗力会显著下降，预防感染是第一要务：*	**绝对禁忌生食或半生食物：** 严禁食用生鱼片、醉虾、未全熟的牛排、溏心蛋、未经巴氏消毒的奶制品或鲜榨果汁。*	**避免不洁食物：** 水果必须彻底清洗并削皮，蔬菜必须煮熟。避免在卫生条件不佳的餐馆就餐，防止肠道感染（严重的系统性感染有时会诱发NMOSD复发）。### 3. 针对自身免疫系统的饮食禁忌：NMOSD的本质是免疫系统过度活跃并错误攻击自身的中枢神经系统。*	**慎用“提高免疫力”的滋补品：** 这是很多患者容易踩的雷区。**不建议**盲目食用人参、黄芪、鹿茸、灵芝、紫锥菊等具有免疫刺激作用的补品或中药。过度激活免疫系统可能增加疾病复发的风险。*	**减少促炎食物：** 尽量避免高度加工食品。建议采用抗炎饮食（类似地中海饮食），多摄入富含优质蛋白（瘦肉、鱼类）、Omega-3脂肪酸（深海鱼、亚麻籽）和抗氧化维生素的新鲜蔬果。**总结建议：**NMOSD患者的饮食核心可以概括为**“低盐、低糖、低脂、高钙、高蛋白、绝对卫生”**。每个患者的具体用药方案和肝肾功能、代谢状况不同，建议您在定期复查神经内科的同时，将近期的常规生化检查（血糖、血脂、电解质）结果告知医生，必要时由临床营养师为您制定个体化的饮食方案。

**20、面对NMOSD高复发风险和高致残率，我感到非常焦虑和抑郁，该怎么办？**

你好。作为一名眼科医师，我非常理解你现在的感受。视神经脊髓炎谱系疾病（NMOSD）不仅是对身体的挑战，更是对心理韧性的巨大考验。这种“不可预测性”带来的焦虑和对失能的恐惧，是绝大多数患者都会经历的过程。面对这种情况，我建议从以下几个专业的维度来应对：### 1. 科学认知：现在的医疗手段已非往昔。你所担心的“高复发”和“高致残”，在很大程度上是基于过去缺乏特效药时代的统计数据。*	**进入“生物制剂时代”：** 近年来，针对NMOSD的创新药物（如萨特利珠单抗、伊奈利珠单抗、依库珠单抗等）相继上市。这些药物能精准打击致病靶点，将年复发率降低了百分之七十甚至更多。*	**规范化治疗：** 只要与神经内科和眼科医生紧密配合，进行长期、规范的免疫抑制治疗，绝大多数患者是可以维持在长期稳定的状态，像普通人一样生活的。### 2. 建立“预警机制”：变被动为主动。焦虑往往源于对失控的恐惧。如果你能掌握主动权，焦虑感会减轻：*	**识别复发征兆：** 留意视力下降、眼痛、肢体麻木或无力等早期信号。*	**及时就诊：** 一旦发现异常，在“黄金24小时”内进行大剂量激素冲击或血浆置换，可以极大程度地挽回视功能和运动功能，显著降低致残风险。### 3. 心理干预：寻求专业支持。NMOSD引起的焦虑和抑郁，有时不仅仅是心理压力，也可能与神经系统炎症或长期使用激素带来的副作用有关。*	**心理咨询：** 寻找擅长慢性病管理的心理咨询师，学习如何与疾病共处。*	**药物辅助：** 如果抑郁和焦虑已经影响了睡眠和日常生活，不要排斥去精神科就诊。适量的抗焦虑、抗抑郁药物可以帮助你度过最艰难的心理低谷期。### 4. 寻找“战友”：加入患者组织。孤军奋战会放大恐惧。加入正规的NMOSD患者组织（如“罕见病中心”下的相关社群），你会发现很多患病十年、二十年的“老病友”依然生活得很好。他们的经验和支持能给你最真实的信心。### 5. 调整生活节奏*	**避免诱因：** 预防感冒、避免过度劳累、保持体温稳定（避免洗过热的热水澡），这些都能减少免疫系统的波动。*	**接纳不完美：** 允许自己有情绪低落的时候，但请记住，你的价值并不由你的疾病决定。**医师寄语：**NMOSD是一场长跑。我们现在的目标不是“彻底消灭”它，而是通过科学的手段“驯服”它，让它成为你生命中一个可以控制的背景音，而不是主旋律。如果你最近感到视力有波动，或者情绪压抑到无法呼吸，请务必尽快回访你的主治医生。我们一直在你身边。你愿意和我聊聊你目前具体的治疗方案，或者最近一次复查的情况吗？

**Claude**

1、**如何确诊NMOSD？**

\# NMOSD（视神经脊髓炎谱系疾病）的确诊标准## 一、核心诊断框架。目前国际通用的是 **2015年国际NMO诊断小组（IPND）诊断标准**。二、关键诊断要素。1. 生物标志物检测（最重要）。AQP4-IgG（水通道蛋白4抗体）检测**这是确诊的"金标准"核心指标。推荐使用 **细胞底物法（CBA法）**，敏感性和特异性最高（>99%）。阳性即高度支持NMOSD诊断**MOG-IgG检测**- AQP4-IgG阴性时需检测- 阳性归类为 **MOGAD**（MOG抗体相关疾病，目前已独立分类）。2. 核心临床症候群（6类） 临床表现 | 特征 | **视神经炎** | 常为重度，双眼受累多见 | **急性脊髓炎** | 横贯性，MRI≥3个节段（LETM）| **极后区综合征** | 顽固性呃逆、恶心呕吐 | **急性脑干综合征** | **症状性发作性睡病/间脑综合征** | **症状性大脑综合征** | 3. MRI影像特征。脊髓MRI：**- 纵向延伸横贯性脊髓炎（LETM）：**≥3个椎体节段**- 急性期脊髓肿胀，中央灰质受累（"亮点征"）。**脑MRI：*- 极后区/第四脑室周围病灶- 下丘脑、胼胝体、大脑半球白质病灶。**视神经MRI：**- 视神经长节段受累（>1/2视神经长度）- 视交叉受累常见4. 脑脊液检查。| 指标 | NMOSD表现 | 白细胞 | 可升高，甚至>50×10⁶/L | 中性粒细胞 | 急性期可出现 | OCB（寡克隆区带） | **多为阴性**（区别于MS）| 蛋白 | 可轻度升高 |三、AQP4-IgG 阳性 vs 阴性诊断路径。AQP4-IgG 阳性└─→ 具备≥1个核心临床症候群└─→ 排除其他诊断 → 确诊NMOSDAQP4-IgG 阴性或未知└─→ 需≥2个核心临床症候群+ 至少1项MRI附加条件+ 排除其他诊断 → 确诊NMOSD。四、重要鉴别诊断。需排除以下疾病：- **多发性硬化（MS）** — OCB阳性，脊髓病灶短小。- **结核/感染性脊髓炎**。- **系统性红斑狼疮、干燥综合征**相关神经病变。- **维生素B12缺乏**。- **肿瘤/副肿瘤综合征**。五、临床建议。**重要提示：**- 抗体检测应在**免疫治疗前**采血，以避免假阴性。- 单次阴性不能排除，病情活动期重复检测。- 建议至**神经眼科或神经内科专科**就诊，结合临床综合判断。如您有具体症状或检查结果需要进一步解读，欢迎详细描述，我可以帮助进一步分析。

**2、NMOSD与多发性硬化有什么区别？**

\# NMOSD与多发性硬化症（MS）的鉴别## 一、疾病本质| 特征 | NMOSD | 多发性硬化（MS） || 全称 | 视神经脊髓炎谱系疾病 | Multiple Sclerosis || 核心机制 | **体液免疫**为主（B细胞/抗体介导） | **细胞免疫**为主（T细胞介导） || 标志性抗体 | **AQP4-IgG**（水通道蛋白4抗体）阳性率约70-80% | 无特异性抗体（OCB寡克隆带阳性） |## 二、临床特征对比| 特征 | NMOSD | MS || 视神经炎严重程度 | **重**，常双眼，视力预后差 | 相对轻，多单眼，预后较好 || 脊髓炎 | **长节段**（≥3个椎体，LETM） | 短节段（<2个椎体） || 极后区综合征 | **特征性**（顽固性呃逆、恶心呕吐） | 罕见 || 病程 | 复发-缓解为主，**无继发进展** | 可转为继发进展型 || 复发后恢复 | **差**，常遗留严重残疾 | 相对较好 || 种族/性别 | 亚裔、女性更多见（女:男≈9:1） | 白种人多见（女:男≈2:1） |## 三、影像学差异**MRI是关键鉴别手段：**- **NMOSD**：脊髓病灶纵向延伸≥3个椎体节段，位于中央，可累及延髓最后区- **MS**：脊髓病灶短小（<2椎体），位于周边；脑内病灶符合Dawson手指征，侧脑室旁多见## 四、治疗策略差异**重要警示**：部分MS治疗药物**禁用于NMOSD**，可能加重病情！| | NMOSD | MS || 急性期 | 大剂量激素冲击 ± 血浆置换 | 大剂量激素冲击 || 预防复发 | 硫唑嘌呤、吗替麦考酚酯、**萨特利珠单抗**、伊奈利珠单抗 | 干扰素-β、那他珠单抗、奥克利珠单抗等 || 禁忌 | 那他珠单抗、芬戈莫德可能**有害** | — |## 五、预后- **NMOSD**：若不治疗，约半数患者5年内出现严重视力障碍或截瘫，致残率高- **MS**：病程多变，部分患者长期功能保存较好**结论**：两者虽都是中枢神经系统脱髓鞘疾病，但致病机制、靶点、影像表现和治疗方案有根本性差异。**AQP4-IgG抗体检测**是目前最重要的鉴别手段之一，建议有相关症状的患者尽早至神经眼科或神经内科专科就诊，完善相关检查。

**3、AQP4抗体阴性是否能诊断为NMOSD？**

\# AQP4抗体阴性与NMOSD诊断## 简短回答**可以。** AQP4抗体阴性并不排除NMOSD的诊断。## 详细解析### 诊断框架（2015年国际共识诊断标准）NMOSD（视神经脊髓炎谱系疾病）的诊断分为**两条路径**：| 路径 | AQP4-IgG状态 | 核心临床标准要求 || **路径一** | **阳性** | 满足≥1条核心临床特征，排除其他诊断 || **路径二** | **阴性或未知** | 需满足**更严格**的附加MRI标准 + ≥2条核心临床特征 |### AQP4抗体阴性时，需满足的条件**核心临床特征（至少2条，且其中1条须为①②③）：**1. 视神经炎2. 急性脊髓炎3. 极后区综合征（顽固性呃逆/恶心呕吐）4. 急性脑干综合征5. 症状性发作性睡病或急性间脑综合征（伴NMOSD典型间脑MRI病变）6. 症状性大脑综合征（伴NMOSD典型脑部MRI病变）**同时需满足附加MRI要求：**- 视神经炎：脑MRI正常，或视神经长节段病变（>1/2视神经长度）或累及视交叉- 脊髓炎：长节段脊髓病变（≥3个椎体节段）或≥3个椎体节段的局灶性脊髓萎缩- 极后区综合征：延髓背侧/极后区病变### 关于MOG抗体的重要补充> 对于**AQP4抗体阴性**的疑似NMOSD患者，**强烈建议检测MOG-IgG（MOG抗体）**- MOG抗体阳性 → 目前倾向于单独归类为 **MOGAD（MOG抗体相关疾病）**，是独立疾病实体- 两者在治疗策略和预后上存在**重要差异**### 临床建议：AQP4阴性疑似NMOSD患者的处理流程：1. 复查AQP4抗体（高灵敏度CBA法）2. 检测MOG-IgG3. 完善脊髓/视神经/脑部MRI4. 排除：MS、感染性、副肿瘤综合征等5. 结合临床表型综合判断**结论：** AQP4阴性NMOSD的诊断是成立的，但门槛更高，需要更充分的临床与影像学证据支撑，并注意与MOGAD及MS的鉴别诊断。如有具体病例情况，建议进一步讨论。

4、**NMOSD急性期的治疗方案有哪些？**

\# NMOSD（视神经脊髓炎谱系疾病）急性期治疗方案## 一、急性期治疗目标。尽快控制炎症、减少神经损伤、促进功能恢复。## 二、一线治疗### 1. 大剂量糖皮质激素冲击治疗（首选）- **方案**：甲泼尼龙 **1000 mg/天** 静脉滴注，连用 3~5 天- 随后口服泼尼松逐渐减量（通常数周内减完）- **注意**：激素减量过快可能诱发复发，NMOSD对激素依赖性较高，减量需缓慢### 2. 血浆置换（Plasma Exchange，PLEX）- **适应症**：- 激素治疗反应不佳（3~5天后无明显改善）- 病情严重（视力严重下降、脊髓完全损伤）- 作为一线治疗的联合方案- **方案**：每次置换 1~1.5 倍血浆量，隔日一次，共 5~7 次- 多项研究显示其对AQP4-IgG阳性患者效果较好## 三、二线/补充治疗### 3. 静脉注射免疫球蛋白（IVIg）- **方案**：0.4 g/kg/天，连用 5 天- 适用于：不能耐受激素或血浆置换的患者- 证据级别相对较弱，多作为备选## 四、急性期治疗流程（简化）。急性发作确认-甲泼尼龙冲击（1g/d × 3~5天）-评估治疗反应-有效 → 激素减量 + 尽快启动长期预防治疗-无效/严重 → 联合/序贯血浆置换（5~7次）-仍无效 → IVIg 或再次血浆置换## 五、眼科重点关注| 临床表现 | 处理重点 || 急性视神经炎（视力急剧下降） | 及早大剂量激素冲击，越早越好 || 视力 < 0.1 或无光感 | 强烈建议联合血浆置换 || 反复发作性视神经炎 | 急性期处理后**务必尽快启动长期免疫治疗** |## 六、重要提示 **NMOSD与MS的鉴别至关重要**：NMOSD急性期**不建议使用**某些MS一线药物（如干扰素β、那他珠单抗），可能加重病情。急性期治疗结束后，**须立即启动长期预防复发治疗**（如利妥昔单抗、萨特利珠单抗、伊奈利珠单抗等），以防再次发作造成不可逆损伤。如需进一步了解**长期预防治疗方案**或**AQP4-IgG阴性患者的特殊处理**，可继续提问。

**5、为什么NMOSD会出现顽固性呃逆、恶心和呕吐？**

\# NMOSD相关顽固性呃逆、恶心与呕吐的病理机制## 核心解剖基础。NMOSD（视神经脊髓炎谱系疾病）出现上述症状，根本原因在于**极后区（Area Postrema, AP）受累**。极后区位于：- 延髓背侧，第四脑室底部尾端- 属于**室周器官（circumventricular organ）**，缺乏完整的血脑屏障- 正是这一特点，使其成为循环中**AQP4抗体攻击的优先靶点**## 发病机制详解### 1️免疫攻击靶点。AQP4（水通道蛋白4）在极后区**星形胶质细胞终足高密度表达**，IgG型AQP4-Ab与之结合后。### 2️极后区的生理功能| 功能 | 受损后表现 || 化学感受器触发区（CTZ）| 呕吐反射失控 || 呼吸/心血管调控 | 呃逆中枢异常放电 || 迷走神经背核毗邻 | 恶心信号持续传入 || 孤束核连接 | 内脏感觉整合紊乱 |### 3️为何"顽固"？- 病灶位于**延髓实质**，非功能性抑制，而是**结构性损伤**- 普通止吐药（如甲氧氯普胺）针对外周/多巴胺通路，**难以逆转中枢结构损伤**- 病灶常同时累及**孤束核-迷走神经背核复合体（NTS-DMV complex）**，形成持续性异常神经环路## 临床特征提示 **重要临床警示**：顽固性呃逆（>48小时）、难治性恶心呕吐，尤其是**无明确消化道病因**的年轻女性，应高度警惕NMOSD极后区综合征，及时完善**AQP4-IgG**检测及头颅/延髓MRI。典型MRI表现：延髓背侧 $T_2$/FLAIR高信号，可延伸至颈髓。## 治疗原则。急性期需针对**免疫机制**本身干预：1. **大剂量糖皮质激素**冲击（一线）2. **血浆置换**（激素反应不佳时）3. 对症止吐（氯丙嗪、昂丹司琼）仅为辅助。长期需启动**疾病修正治疗（DMT）**：萨特利珠单抗、伊奈利珠单抗等靶向治疗。如需进一步了解鉴别诊断或具体诊疗流程，欢迎继续提问。

**6、如何有效预防NMOSD复发？**

\# NMOSD复发预防：专业医师建议## 疾病背景简述。视神经脊髓炎谱系疾病（NMOSD）是一种以**AQP4-IgG抗体**介导为主的自身免疫性中枢神经系统炎性疾病，具有高复发率和高致残性，预防复发是治疗核心目标。## 一、长期免疫抑制治疗（核心策略）### 传统一线方案| 药物 | 常用剂量 | 注意事项 || 硫唑嘌呤 | 2–3 mg/kg/d | 监测血常规、肝功能 || 吗替麦考酚酯 | 1–1.5 g bid | 育龄女性需避孕 || 小剂量泼尼松 | ≤10 mg/d（维持） | 长期使用注意骨质疏松 |### 新型靶向生物制剂（优先推荐，尤其AQP4-IgG阳性者）- **萨特利珠单抗（Satralizumab）**：IL-6受体拮抗剂，皮下注射- **伊奈利珠单抗（Inebilizumab）**：抗CD19，B细胞清除- **依库珠单抗（Eculizumab）**：补体C5抑制剂，静脉输注- **乌帕替尼/利妥昔单抗**：抗CD20，广泛使用（超适应证）**关键原则：不可擅自停药**，停药是复发的最常见诱因之一。## 二、急性发作的快速处理。复发时**尽早就医**，48小时内启动：- 大剂量甲泼尼龙冲击（1g/d × 3–5天）- 必要时血浆置换或静脉免疫球蛋白。早期干预可显著减少神经功能损伤。## 三、生活方式管理。重要触发因素需规避：****感染**：感冒、尿路感染等均可诱发复发，需积极预防和治疗**疫苗接种**：优先选择**灭活疫苗**，避免活疫苗；接种前与主治医师沟通**过度劳累与应激**：规律作息，避免极度疲劳**体温过高**（Uhthoff现象）：避免高温环境，热水浴需谨慎## 四、眼科专项监测。NMOSD常累及**视神经**，建议：- 定期视力、视野、色觉检查（每3–6个月）- **OCT检查**监测视网膜神经纤维层（RNFL）厚度- 视觉诱发电位（VEP）评估亚临床损害- 出现视力下降、眼痛、色觉改变需**立即就诊**，勿延误## 五、多学科随访要点神经科 ── 定期MRI复查（脑 + 脊髓）。眼科 ── 视神经功能监测。风湿免疫科 ── 自身抗体滴度（AQP4-IgG）动态监测。心理支持 ── 长期慢病管理，防治抑郁焦虑## 总结建议> **"坚持规范用药 + 定期随访 + 避免诱因"** 是预防NMOSD复发的三大支柱。AQP4-IgG阳性患者复发风险更高，更应积极使用靶向生物制剂。请务必在**神经科专科医师指导**下制定个体化方案，切勿自行调整治疗。如需进一步了解某一具体方面（如妊娠期管理、某种药物的副作用等），欢迎继续提问。

**7、目前NMOSD缓解期的一线预防药物有哪些？**

\# NMOSD缓解期一线预防治疗药物。作为神经眼科医师，以下是目前**视神经脊髓炎谱系疾病（NMOSD）**缓解期公认的一线预防（疾病修正）治疗方案：## 一、靶向治疗（单克隆抗体）### 1. Inebilizumab（伊奈利珠单抗）- **靶点**：抗CD19，清除B细胞- **用法**：静脉注射，首次给药后半年一次- **循证**：N-MOmentum试验，显著降低复发风险### 2. Satralizumab（萨曲利珠单抗）- **靶点**：抗IL-6受体- **用法**：皮下注射，每4周一次- **特点**：可皮下给药，患者依从性较好### 3. Eculizumab（依库珠单抗）- **靶点**：抗补体C5蛋白- **用法**：静脉注射，维持期每2周一次- **注意**：使用前须接种脑膜炎球菌疫苗## 二、传统免疫抑制剂（经典一线）| 药物 | 用法 | 注意事项 || **吗替麦考酚酯（MMF）** | 口服，1000–1500 mg bid | 妊娠禁用 || **硫唑嘌呤（AZA）** | 口服，2–3 mg/kg/d | 需监测血常规、肝功 || **利妥昔单抗（RTX）** | 静脉注射，每6–12月 | 抗CD20，国内应用广泛 |**利妥昔单抗**虽属超说明书用药，但在中国临床实践中已被广泛认可，多项指南推荐。## 三、治疗选择原则- **AQP4-IgG阳性**患者：上述靶向药物证据最充分- **MOG-IgG阳性**患者：靶向药物数据有限，目前多用MMF或RTX- **血清阴性**患者：方案选择参考临床表型## 重要提醒> 1. **不建议**使用干扰素-β或那他珠单抗（可能加重病情）> 2. 急性发作期首选**大剂量糖皮质激素**冲击，缓解期维持治疗需长期坚持> 3. 具体用药方案须由神经内科/神经眼科专科医师根据个体情况制定。如需进一步了解某一具体药物的用法、副作用或妊娠期用药建议，欢迎继续提问。

**8、新型的单抗类药物与传统免疫抑制剂相比，在治疗NMOSD中各自的优缺点是什么？**

\# NMOSD治疗：单抗类药物 vs 传统免疫抑制剂## 背景概述：视神经脊髓炎谱系疾病（NMOSD）以AQP4-IgG抗体阳性为主要特征，治疗目标为**预防复发、减少神经功能残疾积累**。## 一、新型单克隆抗体药物### 主要代表| 药物 | 靶点 | 机制 || **萨特利珠单抗** (Satralizumab) | IL-6受体 | 抑制IL-6信号通路 || **依奈利珠单抗** (Inebilizumab) | CD19 | 消耗B细胞 || **伊奈利珠单抗/乌利妥昔单抗** (Ublituximab) | CD20 | B细胞清除 || **依库珠单抗** (Eculizumab) | 补体C5 | 阻断补体介导损伤 |###优点- **疗效显著**：各大III期RCT均显示年复发率（ARR）降低约70–94%- **靶点精准**：针对AQP4-IgG致病机制的关键环节- **安全性更可预测**：不良反应谱相对清晰- **循证证据充分**：均有高质量RCT支持，已获FDA/EMA批准- **给药便利**：多为皮下注射或长间隔静脉输注##缺点- **费用极高**：年治疗费用可达数十万至百万人民币- **感染风险**：B细胞耗竭类药物增加肺炎、带状疱疹等风险- **依库珠单抗**：脑膜炎球菌感染风险，需预防接种- **长期数据有限**：上市时间较短，超10年数据缺乏- **可及性差**：国内部分药物尚未纳入医保或未上市## 二、传统免疫抑制剂### 主要代表- **硫唑嘌呤（AZA）**- **吗替麦考酚酯（MMF）**- **利妥昔单抗（Rituximab，CD20单抗，属过渡类）**- **甲氨蝶呤、环磷酰胺**（二线或难治性）###优点- **价格低廉**：纳入医保，可及性强- **临床经验丰富**：使用数十年，安全性数据充分- **总体有效**：AZA/MMF可使约60–70%患者减少复发- **口服给药**：依从性相对好（AZA、MMF）###缺点- **起效慢**：AZA需3–6个月才达治疗效果，早期存在复发窗口- **疗效不稳定**：仍有相当比例患者在治疗中复发- **监测负担**：需定期查血常规、肝肾功能- **毒副作用多**：- AZA：骨髓抑制、肝毒性、胃肠道反应- MMF：致畸、感染、淋巴瘤风险- 环磷酰胺：出血性膀胱炎、性腺毒性- **缺乏高级别循证**：多基于回顾性研究或小样本研究## 三、选择建议（个体化原则）高复发风险 / AQP4-IgG强阳性 / 经济条件允许-优先考虑新型单抗-经济受限 / 复发频率低 / 初诊轻症-传统免疫抑制剂起步，密切监测 **重要提示**：所有NMOSD治疗方案均需在**神经眼科或神经免疫专科医师**指导下制定，不可自行调整免疫治疗药物。急性发作期需及时就诊，把握激素冲击和血浆置换时间窗。如需进一步了解某一具体药物的用法、监测方案或与妊娠相关的用药选择，可继续提问。

**9、长期使用预防NMOSD复发的药物，主要的副作用有哪些？**

\# NMOSD复发预防药物的主要副作用。视神经脊髓炎谱系疾病（NMOSD）目前有多类预防复发的药物，各有不同的副作用谱。## 一、传统免疫抑制剂### 硫唑嘌呤（Azathioprine）- 骨髓抑制（白细胞减少、贫血）- 肝功能损害- 胃肠道反应（恶心、呕吐）- 长期使用增加淋巴瘤风险### 吗替麦考酚酯（MMF）- 胃肠道不适（腹泻、恶心）- 感染风险增加- 白细胞减少### 利妥昔单抗（Rituximab）- 输液反应- 低丙种球蛋白血症- **进行性多灶性白质脑病（PML）**（罕见但严重）- B细胞长期耗竭致感染风险升高## 二、新型靶向生物制剂### 伊奈利珠单抗（Inebilizumab）- 感染风险增加（尿路感染、鼻咽炎）- 输液相关反应- 低免疫球蛋白血症### 萨特利珠单抗（Satralizumab，IL-6受体拮抗剂）- 感染（尤其上呼吸道）- 注射部位反应- 肝酶升高- 血脂异常### 依库珠单抗（Eculizumab，补体C5抑制剂）- **脑膜炎奈瑟菌感染风险显著升高**（使用前必须接种疫苗）- 头痛、恶心- 费用极高## 三、长期监测要点| 监测项目 | 频率建议 || 血常规 + 肝肾功能 | 每3～6个月 || 免疫球蛋白水平 | 每6个月 || 结核/乙肝筛查 | 用药前及定期 || 感染症状 | 随时警惕 |##重要提示> 所有NMOSD预防用药均需在**神经科或眼科专科医师**的密切监管下使用，**切勿自行停药**——停药后复发风险极高，且每次复发可能造成不可逆的视力或神经功能损害。如需了解某一具体药物的详细副作用或用药注意事项，可进一步说明。

**10、在NMOSD用药期间可以接种疫苗吗？**

\# NMOSD用药期间的疫苗接种问题## 总体原则：NMOSD（视神经脊髓炎谱系疾病）患者的疫苗接种需**个体化评估**，核心考量是：**当前使用的免疫治疗药物类型**。## 按药物分类建议###需特别谨慎——禁用活疫苗：使用以下药物期间，**禁止接种减毒活疫苗**（如MMR、水痘、带状疱疹减毒活疫苗、黄热病疫苗等）：| 药物 | 原因 || 利妥昔单抗（Rituximab） | B细胞清除，免疫应答严重受损 || 伊奈利珠单抗（Inebilizumab） | 同上 || 萨特利珠单抗（Satralizumab） | IL-6受体拮抗，免疫抑制 || 依库珠单抗（Eculizumab） | 补体抑制，感染风险显著升高 || 硫唑嘌呤 / 吗替麦考酚酯 | 免疫抑制状态 || 口服激素（长期大剂量） | 免疫抑制 ###**推荐接种的灭活/重组疫苗**以下疫苗在NMOSD治疗期间通常**安全可接种**：- 流感疫苗（灭活，每年推荐）- 肺炎球菌疫苗（PCV13/PPSV23）- COVID-19疫苗（mRNA或灭活）- 乙肝疫苗- 带状疱疹重组疫苗（**Shingrix**，非活疫苗，推荐）- HPV疫苗## 特殊注意事项### 关于利妥昔单抗的接种时机> 这是临床最常见的情况- **用药前**：理想情况下应在首次输注**前≥4周**完成所需疫苗接种- **用药期间**：灭活疫苗可接种，但抗体应答可能**明显减弱**- **末次输注后**：建议间隔**≥6个月**再接种，以获得更佳免疫应答- **下次输注前**：尽量在输注前**2~4周**完成接种### 关于依库珠单抗> 补体通路被阻断，**脑膜炎球菌感染风险极高**- **强制要求**：用药前必须接种**脑膜炎球菌疫苗*（MenACWY + MenB）- 这是用药的前提条件之一## 给患者的实用建议1. **主动告知医师**目前所有在用药物2. **不要自行决定**疫苗接种，需由主治医师评估3. **家庭成员**建议保持疫苗接种更新，形成间接保护4. 每次**换用新药前**，提前规划疫苗接种计划**免责提示**：以上为医学科普信息，具体接种方案请结合个人病情，由您的神经内科或眼科主治医师制定个性化方案。

**11、如何缓解NMOSD引起的长期神经性疼痛、肌肉痉挛或顽固性瘙痒？**

\# NMOSD相关慢性神经症状的管理## 背景说明：视神经脊髓炎谱系疾病（NMOSD）引起的**神经性疼痛、肌肉痉挛和顽固性瘙痒**，均源于脊髓或视神经的中枢神经损伤，属于**慢性神经病理性症状**，治疗需分层、个体化。## 一、神经性疼痛### 一线药物| 药物 | 剂量参考 | 注意事项 || **加巴喷丁** | 300~3600mg/d，分次 | 肾功能调整剂量 || **普瑞巴林** | 75~600mg/d，分次 | 起效较快 || **阿米替林** | 10~75mg/d，睡前 | 心脏病患者慎用 || **度洛西汀** | 30~120mg/d | 兼顾抑郁共病|### 二线药物- **拉莫三嗪、卡马西平**：尤适合阵发性痛性痉挛- **曲马多**：短期辅助，警惕依赖- **利多卡因贴片/5%乳膏**：局部神经痛### 非药物手段- 经皮神经电刺激（TENS）- 认知行为疗法（CBT）- 心理支持干预## 二、肌肉痉挛（Spasticity/Tonic Spasm）NMOSD的**痛性强直痉挛（PTS）** 是其特征性表现，需积极处理。### 药物治疗- **巴氯芬**（首选）：起始5mg tid，逐渐加量至30~80mg/d；重症者可考虑**鞘内巴氯芬泵**- **替扎尼定**：2~36mg/d，注意肝功能监测- **地西泮**：短期辅助，镇静副作用明显- **大麻素类（如萘比隆）**：部分国家/地区批准，对顽固痉挛有效### 非药物- **规律康复训练**：牵伸、水疗、功能训练- **肉毒毒素注射**：局灶性痉挛的重要选择- **物理治疗**：体位管理、矫形器具## 三、顽固性瘙痒（Neuropathic Itch）NMOSD相关瘙痒机制独特，是**中枢性神经病理性瘙痒**，对普通止痒药无效。### 针对性治疗| 药物 | 机制 | 证据 || **加巴喷丁/普瑞巴林** | 降低中枢敏化 | 有效证据较充分 || **阿片受体拮抗剂（纳曲酮）** | 调节阿片通路 | 部分有效 || **米氮平** | 抗组胺+中枢作用 | 对顽固瘙痒有效 || **NK1受体拮抗剂（阿瑞匹坦）** | 阻断P物质通路 | 小样本有效报告 |>**抗组胺药通常无效**，因其为中枢性而非外周性瘙痒。## 四、整体原则：控制疾病活动（免疫治疗）→ 是症状管理的前提-充分镇痛/解痉/止痒药物治疗-多学科协作（神经科 + 疼痛科 + 康复科 + 心理科）-定期评估：VAS评分 / NPSI量表 / 生活质量评估### 特别强调- **勿随意停用免疫抑制治疗**（硫唑嘌呤、利妥昔单抗等），复发是症状加重的首要原因- 所有症状药物均需**从小剂量起始，缓慢滴定**-症状难控制时，建议转诊至**NMOSD专病中心**或**神经病理性疼痛专科**> **免责提示：** 以上信息为医学科普参考，具体用药方案须由您的主治医师结合病情、用药史及检查结果个体化制定，请勿自行调整治疗。

**12、NMOSD导致的大小便功能障碍怎么办？**

\# NMOSD相关膀胱/肠道功能障碍的管理## 一、病理机制简述。NMOSD（视神经脊髓炎谱系疾病）累及**脊髓**（尤其是长节段横贯性脊髓炎）时，可损伤支配膀胱、直肠的自主神经及躯体神经通路，导致排尿/排便功能障碍。## 二、膀胱功能障碍处理###评估优先- **尿动力学检查**（金标准）：明确是痉挛性膀胱、弛缓性膀胱还是括约肌协同失调- 泌尿外科/神经泌尿科会诊###治疗策略| 类型 | 处理方案 || **逼尿肌过度活动**（痉挛性）| 抗胆碱药（奥昔布宁、索利那新）或β₃激动剂（米拉贝隆）|| **逼尿肌-括约肌协同失调** | 间歇导尿 ± α受体阻滞剂（坦索罗辛）|| **弛缓性膀胱/尿潴留** | **清洁间歇导尿（CIC）**（首选非手术方案）|| **难治性** | 膀胱内肉毒素注射、骶神经调控 |>**注意**：定期监测残余尿量（>100ml需积极干预），预防反复泌尿系感染及上尿路损害## 三、肠道功能障碍处理###神经源性肠道常见表现- 便秘（多见）- 大便失禁- 排便感觉障碍###分级管理**基础措施：**- 高纤维饮食（25~30g/日）+ 充足水分- 规律排便训练（利用胃结肠反射，餐后30分钟）- 腹部按摩手法**药物治疗：**- 容积性泻药（车前子制剂）- 渗透性泻药（乳果糖、聚乙二醇）- 必要时栓剂（甘油栓）或小剂量灌肠**大便失禁：**- 盆底肌训练- 止泻药（洛哌丁胺）酌情使用## 四、疾病本身的控制（根本）大小便障碍属于**脊髓损伤的继发症状**，因此：- **急性期**：大剂量糖皮质激素冲击治疗（甲泼尼龙1g/日×5天）- **缓解期预防复发**： - 免疫抑制剂（吗替麦考酚酯、硫唑嘌呤）- 靶向生物制剂（**萨特利珠单抗、伊奈利珠单抗、依库珠单抗**）——抗AQP4抗体阳性患者优先推荐## 五、多学科协作建议：神经科（主导）├── 泌尿外科（膀胱管理）├── 消化科（肠道管理）├── 康复科（盆底康复）└── 心理科（情绪支持）> **温馨提示**：大小便功能障碍严重影响生活质量，患者应避免因羞于就诊而延误处理，及时与主治神经科医生沟通，进行规范的尿动力评估和个体化管理方案制定。

**13、NMOSD病情稳定后，应该如何进行科学、安全的康复训练？**

\# NMOSD视神经损伤康复训练指南## 一、康复前提条件。在开展康复训练前，需确认：- 病情处于**稳定缓解期**（至少3个月无复发）- 急性期炎症已控制，影像学无新发病灶- 已接受规范免疫抑制治疗（如利妥昔单抗、托珠单抗等）---## 二、视功能康复训练### 1. 视觉刺激训练- **对比敏感度训练**：使用渐变灰阶图卡，从高对比度逐步过渡至低对比度- **色觉恢复训练**：红绿色觉辨别练习（NMOSD易累及色觉纤维）- 每次 **15~20分钟**，每日1~2次，避免视觉疲劳### 2. 视野训练- 针对中心暗点或偏盲区域，进行**代偿性眼球扫视训练**- 借助视野计或专业视觉康复软件辅助- 推荐：**NovaVision VRT** 等经过验证的视觉康复系统### 3. 眼球运动训练- 平滑追踪、扫视训练（对合并脑干病变者尤为重要）- **Brock线法**、**Hart图法**均可使用- 注意：若存在核间性眼肌麻痹，需在医师指导下进行## 三、神经可塑性促进训练。NMOSD损伤以**AQP4抗体介导的星形胶质细胞损伤**为核心，视神经修复有限，但可通过以下方式促进功能代偿：| 方法 | 机制 | 推荐强度 || 有氧运动（步行、游泳） | 促进BDNF分泌，增强神经可塑性 || 认知-视觉双任务训练 | 强化视皮质代偿通路 | 经颅直流电刺激（tDCS） | 调节视皮质兴奋性（试验性） |## 四、全身康复注意事项###特别警示：Uhthoff现象。NMOSD患者对**体温升高极为敏感**：- 避免高温环境、热水浴、剧烈运动导致核心体温升高- 运动时使用**降温背心**或冷敷颈部- 出现视力一过性模糊应立即停止运动、降温### 其他注意事项- **疲劳管理**：遵循"能量节省原则"，运动与休息比约为 1:2- 避免**过度劳累**诱发免疫激活- 规律作息，保证睡眠（7小时）## 五、心理与认知康复- NMOSD患者抑郁发生率约**40%**，应同步进行心理疏导- 认知训练：记忆、注意力强化训练（脑干/皮质下白质受累时尤为重要）- 建议参加**患者支持团体**（如NMOSD专病患者群）## 六、随访监测。康复期随访建议：- 每3个月：视力、视野、OCT视网膜神经纤维层厚度- 每6个月：MRI（脑+脊髓+视神经）- 每年：AQP4-IgG滴度、免疫功能评估> **郑重提示**：以上为一般性康复指导原则，NMOSD个体差异较大，具体方案须结合您的**病灶位置、残余功能、用药方案**，由神经眼科医师或康复科医师制定个性化方案。任何新出现的症状（视力下降、肢体麻木加重）须立即就医，警惕复发。

**14、NMOSD能治愈吗？**

\# NMOSD（视神经脊髓炎谱系疾病）能治愈吗？## 直接回答。目前**NMOSD尚无法根治**，但通过规范治疗可以显著控制病情、减少复发、保护视力和神经功能。## 疾病特点。NMOSD是一种**自身免疫性中枢神经系统炎性疾病**，主要攻击视神经和脊髓，多数患者（>80%）体内存在**AQP4-IgG抗体**（水通道蛋白4抗体）。该病具有：- 反复**复发**的特性- 每次发作可能造成**累积性神经损伤**- 视力损害可能较严重甚至致盲## 治疗目标| 阶段 | 目标 || 急性发作期 | 尽快控制炎症，减少神经损伤 || 缓解期 | **预防复发**（长期免疫治疗） || 康复期 | 最大限度恢复神经功能 |## 现有治疗手段**急性期：**- 大剂量甲泼尼龙冲击治疗- 血浆置换（激素无效时）**长期预防复发（目前主流方案）：**- **萨曲珠单抗**（Satralizumab）- **伊奈利珠单抗**（Inebilizumab）- **依库珠单抗**（Eculizumab）- 传统免疫抑制剂：吗替麦考酚酯、硫唑嘌呤、利妥昔单抗> 上述靶向生物制剂已被证实可**将复发率降低70-90%**，部分患者可实现长期"无复发"状态。## 预后与展望- 坚持**规范长期治疗**的患者，预后明显改善- 医学界正在研究更精准的免疫调节疗法- **早期诊断、早期干预**是保护视力和运动功能的关键>**重要提示**：NMOSD需要神经眼科或神经内科专科医师长期随访管理，切勿自行停药。如有视力骤降、眼痛等症状，请**立即就医**如需进一步了解某一方面（如具体用药方案、随访频率等），欢迎继续提问。

**15、NMOSD的长期预后怎么样？**

\# NMOSD（视神经脊髓炎谱系疾病）的长期预后## 总体概述。NMOSD是一种**严重的中枢神经系统炎性疾病**，以视神经炎和长节段横贯性脊髓炎为核心表现。其长期预后较经典多发性硬化（MS）更差，但近年来随着靶向治疗的出现，预后已显著改善。## 视觉预后（眼科重点）### 视神经炎发作后| 预后指标 | 情况说明 || 视力恢复 | 约**50-60%**患者残留明显视力损害 || 严重视力丧失 | 反复发作后约**20-30%**可致法定盲（视力<0.1） || 恢复时间 | 通常发作后数周至3个月，但常不完全恢复 || 对比敏感度/色觉 | 即使视力表视力恢复，仍可能持续受损 |### 影响视力预后的关键因素- **发作次数**：每次复发均可累积不可逆损害- **治疗及时性**：急性期静脉大剂量激素治疗越早越好- **AQP4-IgG状态**：血清阳性者复发风险更高- **OCT检查**：视网膜神经纤维层（RNFL）厚度可客观反映轴突损伤程度## 神经系统整体预后- **复发率**：未经预防治疗，约**90%**患者为复发型病程- **残障积累**：5-10年内约**50%**患者需借助辅助装置行走- **死亡率**：部分患者因延髓受累（呼吸衰竭）而死亡，5年死亡率约**5-10%**（传统数据）## 影响长期预后的核心因素。预后较差 ←→ 预后较好。高复发频率	低复发频率AQP4-IgG阳性 MOG-IgG阳性（相对较好）延误诊断治疗	早期确诊干预。未使用预防治疗 规范免疫抑制治疗## 现代治疗对预后的改变。近年**靶向生物制剂**显著改变了预后格局：| 药物 | 机制 | 降低复发率 || **萨特利珠单抗**（Satralizumab）| IL-6R拮抗剂 | ~74% || **依奈利珠单抗**（Inebilizumab）| 抗CD19 | ~77% || **依库珠单抗**（Eculizumab）| 补体C5抑制剂 | ~94% || 传统方案（硫唑嘌呤/利妥昔单抗）| 免疫抑制 | 50-70% |>**关键信息**：早期启动长期免疫预防治疗是改善预后的**最重要干预措施**。## 给患者的重要建议1. **绝不自行停药** — NMOSD停止预防治疗后复发风险极高2. **定期眼科随访** — 包括视野、OCT、视觉诱发电位3. **警惕复发信号** — 视力突然下降、眼痛须立即就诊4. **多学科管理** — 神经科+眼科+康复科协作>**声明**：以上为一般性医学信息，个体预后差异较大，具体病情评估和治疗方案请结合您的主治医师综合判断。

**16、NMOSD患者可以怀孕生子吗？**

\# NMOSD患者妊娠问题## 简要回答：NMOSD（视神经脊髓炎谱系疾病）患者**可以妊娠**，但属于**高风险妊娠**，需要在神经科、眼科、产科多学科团队严密管理下进行。## 核心风险评估###疾病复发风险- 妊娠期（尤其**产后3个月**）是复发的高峰期- 产后复发风险显著高于非妊娠期，部分研究提示产后复发率可升高2~3倍- 每次复发可能造成**不可逆的视力损害或肢体功能障碍**##胎儿安全风险- 部分维持治疗药物对胎儿有致畸或毒性风险- 严重复发时使用大剂量激素对胎儿有潜在影响## 妊娠前的准备（关键！）| 评估项目 | 要求 || 疾病稳定期 | 建议**至少稳定≥12个月**再考虑妊娠 || AQP4-IgG状态 | 阳性患者风险更高，需更严格管理 || 残障程度评估 | EDSS评分、视力及脊髓功能基线评估 || 药物调整 | 停用/替换致畸药物（如吗替麦考酚酯、利妥昔单抗需提前规划） |## 妊娠期用药原则### 相对安全可使用：- **硫唑嘌呤（AZA）** — 妊娠期较常用的维持治疗选择- **小剂量泼尼松** — 必要时可使用- **静脉注射免疫球蛋白（IVIG）** — 可用于预防产后复发### 需停用或慎用：-**吗替麦考酚酯（MMF）** — 明确致畸，孕前需停用≥6周-**利妥昔单抗** — 孕前需停用至少6个月-**萨特利珠单抗/伊奈利珠单抗** — 妊娠安全数据有限## 产后管理重点> 产后3个月是**复发最危险的窗口期**- 分娩后尽快恢复或加强免疫治疗- IVIG可在产后早期预防性使用- 母乳喂养需结合用药方案个体化评估- 加强神经科随访频率（建议每月评估）## 综合建议1. **不建议在疾病活动期或频繁复发阶段妊娠**2. 妊娠计划需**提前6~12个月**与神经科医师共同制定方案3. 全程在**神经科+产科+眼科联合门诊**监管下进行4. AQP4-IgG强阳性患者需充分知情风险后作出决定>**重要提示**：本回答基于现有循证医学证据，具体决策须结合患者个人病情、抗体状态、用药史等综合评估。建议到神经免疫专科或脱髓鞘疾病专病门诊就诊，制定个体化方案。

**17、孕期和产后如何管理疾病，以降低NMOSD复发风险？**

\# 孕期及产后NMOSD管理指南## 疾病概述：视神经脊髓炎谱系疾病（NMOSD）在育龄女性中高发，妊娠期和产后期是复发高风险阶段，需要**多学科协作管理**（神经科、产科、眼科）。## 一、孕前准备### 疾病稳定性评估- 建议在**病情稳定至少6个月**后再考虑妊娠- 完善AQP4-IgG、MOG-IgG抗体滴度检测- 评估残余神经功能障碍程度### 药物调整（关键）| 需停用药物 | 停药时机 | 原因 || 吗替麦考酚酯 | 孕前≥6周 | 明确致畸性 || 利妥昔单抗 | 孕前≥6个月 | B细胞抑制影响胎儿 || 甲氨蝶呤 | 孕前≥3个月 | 强致畸性 |### 相对安全可继续使用- **硫唑嘌呤**（最常用，需充分评估）- **泼尼松**（低剂量，孕早期慎用）## 二、孕期管理### 监测频率：孕早期：每4周神经科随诊、孕中晚期：每4~6周随诊、眼科视力/视野监测：每孕期至少1次### 复发风险时间段{复发风险：孕早期} >{孕中晚期（相对稳定）}### 急性复发处理1. **首选**：大剂量甲泼尼龙冲击（1g/天×3~5天）2. **血浆置换**：激素无效时可选（孕期相对安全）3. **静脉注射免疫球蛋白（IVIG）**：可作为辅助方案## 三、产后管理（最高风险期）### 风险特征> 产后3~6个月是NMOSD复发的**最高峰期**，复发率可较孕前升高2~3倍，且复发后神经功能损伤更严重。### 产后预防策略#### 1. 尽早恢复/启动免疫抑制治疗- 分娩后**2~4周内**尽早恢复维持治疗- 优先考虑：- **利妥昔单抗**（若不哺乳，首选）- **硫唑嘌呤**- **萨特利珠单抗**（IL-6受体拮抗剂）#### 2. 哺乳问题。硫唑嘌呤 → 可谨慎哺乳（低乳汁分泌量）、利妥昔单抗 → 建议避免哺乳、泼尼松 → 服药后4小时后哺乳相对安全#### 3. 产后IVIG预防性使用- 部分中心推荐产后早期预防性使用IVIG，但循证级别尚低。## 四、眼科专项管理| 情况 | 处理要点 || 视神经炎急性发作 | 大剂量激素冲击，孕期需权衡 || 视力监测 | 每次产检同步记录视力变化 || OCT检查 | 监测RNFL厚度变化 || 视野检查 | 评估复发早期迹象 |## 五、多学科协作要点。神经科 ←→ 产科 ←→ 眼科-新生儿科（评估药物对新生儿影响）## 总结建议：1.孕前充分规划，疾病稳定后妊娠2.全程神经科随诊，避免自行停药3.产后尽早恢复免疫治疗，这是降低复发的**最关键措施**4.眼科定期随访，早期发现视神经炎复发5.任何用药调整须在专科医师指导下进行*以上建议基于现有临床证据，具体方案需结合患者个体情况由专科医师制定。NMOSD管理指南持续更新，建议参考最新ESN/ECTRIMS指南。*

**18、NMOSD会遗传给下一代吗？**

\# NMOSD（视神经脊髓炎谱系疾病）会遗传给下一代吗？## 一、NMOSD 简介**NMOSD（Neuromyelitis Optica Spectrum Disorder，视神经脊髓炎谱系疾病）** 是一种以视神经和脊髓受累为主要特征的中枢神经系统自身免疫性炎性疾病。其核心致病机制与体内产生的**抗水通道蛋白-4抗体（AQP4-IgG）** 密切相关，可导致视力下降甚至失明、肢体无力、感觉障碍等严重后果。## 二、NMOSD 是否具有遗传性？###总体结论：**NMOSD 不是经典的遗传性疾病，但存在一定的遗传易感性。**NMOSD **不会像血友病、白化病那样按照孟德尔遗传定律直接传递给后代**，但遗传因素在其发病中扮演了一定的"背景角色"。## 三、遗传易感性的证据| 维度 | 说明 || **家族聚集性** | 文献中有极少数家族性NMOSD病例报告，但总体发生率极低 || **HLA基因关联** | 研究显示特定HLA基因型（如HLA-DRB1）与NMOSD易感性相关|| **自身免疫背景** | NMOSD患者家族中自身免疫疾病（如系统性红斑狼疮、干燥综合征）发病率略高 || **双胞胎研究** | 同卵双胞胎并非100%同患，提示遗传因素并非决定性因素 |## 四、发病的主要影响因素。NMOSD的发病是**多因素共同作用**的结果，主要包括：1. **遗传易感基因**：提供了"易患体质"，但不等于一定发病2. **环境因素**：感染（如EB病毒、新冠病毒等）可能诱发或加重病情3. **免疫调节异常**：自身免疫耐受机制的失衡4. **激素水平**：NMOSD女性患者约占80%~90%，提示性激素可能参与发病> **通俗理解**：遗传因素更像是"装了一把枪"，而环境和免疫因素才是"扣动扳机"的关键。有遗传易感性不代表一定患病。## 五、对备孕/已孕患者的建议。若您或家人正患有NMOSD，并考虑生育，请注意以下几点：-**后代患NMOSD的绝对风险仍然很低**，不必过度焦虑-**孕期NMOSD可能复发**，需在神经科/眼科医师指导下严格管理-部分治疗药物（如利妥昔单抗、吗替麦考酚酯）在孕期需调整，**切勿自行停药**-建议在病情稳定至少**6个月以上**再考虑妊娠-可考虑进行**遗传咨询**，评估家族发病风险## 六、总结| 问题 | 回答 || NMOSD会直接遗传吗？ |不会按经典遗传方式传递 || 有无遗传易感性？ |有，但贡献较小 || 后代一定会患病吗？ |不一定，风险仍属较低 || 需要遗传咨询吗？ | 建议咨询，以便个体化评估 |>**免责提示**：本文内容仅供医学科普参考，不构成个人诊疗建议。如有具体病情问题，请务必前往正规医院神经内科或眼科就诊，由专业医师进行面对面评估。

**19、NMOSD有什么饮食禁忌？**

\# NMOSD（视神经脊髓炎谱系疾病）饮食注意事项。作为神经眼科医师，我来为您解答。## 一、需要注意的饮食方面###可能需要限制的食物**1. 高盐饮食**- 过多钠盐可能加重自身免疫性炎症反应- 研究提示高盐可激活Th17细胞，加剧自身免疫应答- 建议每日食盐 **< 5g****2. 高脂/高糖饮食**- 可能促进系统性炎症状态- 使用激素治疗期间尤其需控制，以防加重**血糖升高、体重增加****3. 饮酒**- 酒精可能干扰免疫调节药物代谢- 与激素联用加重消化道损伤风险**4. 葡萄柚**- 可影响部分免疫抑制剂（如他克莫司、环孢素）的代谢，**需避免**## 二、服用特定药物时的饮食禁忌| 药物 | 饮食注意 || 糖皮质激素 | 低盐、低糖、补钙、补钾 || 硫唑嘌呤 | 避免高嘌呤食物，注意消化道反应 || 吗替麦考酚酯 | 空腹或餐后服用需遵医嘱，避免葡萄柚 || 他克莫司 | **严格禁忌葡萄柚**，固定进餐时间服药 |## 三、建议积极摄入的食物-**富含Omega-3**的深海鱼（抗炎作用）-*新鲜蔬菜水果**（维生素D、抗氧化剂）-**适量乳制品**（补钙，对抗激素性骨质疏松）-适当补充**维生素D**（免疫调节，常见缺乏）##重要提示：> NMOSD本身无特异性"绝对禁忌"食物，**饮食禁忌主要取决于您正在使用的具体治疗方案**。建议您将目前用药告知医师，制定个性化饮食方案。如需针对您具体用药进行详细说明，欢迎进一步告知。

**20、面对NMOSD高复发风险和高致残率，我感到非常焦虑和抑郁，该怎么办？**

\# 面对NMOSD的心理压力：专业建议。您的焦虑和抑郁感受**完全可以理解**，这是许多NMOSD患者的真实体验。面对一种高复发风险的慢性病，心理负担往往和身体症状同样沉重。##为什么会有这种感受？NMOSD（视神经脊髓炎谱系疾病）的特点决定了心理压力的来源：- **不确定性**：复发时间、严重程度难以预测- **功能威胁**：视力和运动功能受损的恐惧- **长期治疗负担**：持续用药、随访的压力- **社会影响**：工作、家庭角色的潜在改变。研究显示，NMOSD患者中**抑郁和焦虑的发生率显著高于普通人群**，这是疾病本身的一部分，而非"心理脆弱"。##建议采取的行动### 第一步：医学层面——控制复发是核心> 减少焦虑最根本的方式，是切实降低复发风险- **规范使用预防性治疗药物**（如萨特利珠单抗、伊奈利珠单抗、利妥昔单抗等）- 严格遵医嘱随访，监测AQP4-IgG抗体水平- 避免感染、疲劳等诱发因素### 第二步：正视心理问题，寻求专业支持- **明确告知您的神经科医生**您目前的心理状态- **申请转诊心理科/精神科**——焦虑抑郁达到一定程度需要专业干预- 必要时可使用**抗焦虑/抗抑郁药物**（需评估与NMOSD治疗的相互作用）### 第三步：认知行为策略| 方法 | 具体做法 || 聚焦可控因素 | 专注于"我能做到的"（按时服药、健康生活） || 信息管理 | 限制过度搜索负面病例，选择权威来源 || 接纳不确定性 | 学习与"不确定"共存，而非消除它 |### 第四步：社会支持- 加入**NMOSD患者支持团体**（国内有相关病友社群）- 向家人坦诚病情，建立支持系统- 考虑家庭治疗，帮助家属理解您的处境##需要立即就医的信号。如果出现以下情况，请**立即联系医生或心理援助热线**：- 持续两周以上的严重抑郁- 出现自伤或轻生的念头- 完全无法进行日常活：**心理援助热线：** 北京 010-82951332 | 全国 400-161-9995## 最后想对您说，医学的进步让NMOSD的管理越来越规范，**有效预防复发是完全可能实现的目标**。您愿意主动面对这个问题，本身就是一种力量。请您在下次复诊时，把今天的感受**直接告诉您的主治医生**——心理健康是NMOSD综合管理不可缺少的一部分。*以上建议供参考，具体诊疗方案需结合您的个人病情由医生制定。*

**Deepseek**

1、**如何确诊NMOSD？**

您好，我是您的医生。关于视神经脊髓炎谱系疾病的确诊，这是一个需要神经内科和眼科医生共同协作完成的严谨过程。NMOSD的诊断并非依赖单一检查，而是基于**临床表现、影像学特征和血液生物标志物**三方面的综合判断。以下是确诊NMOSD的标准流程和核心检查：#### 1. 核心临床症候（医生会详细询问和检查）。这是诊断的起点。NMOSD有6个特征性的症候群，出现其中任何一项都需高度警惕：**视神经炎**：尤其是**双眼同时或相继发病**、视力损害严重、或恢复不佳的视神经炎。**急性脊髓炎**：通常为**长节段横贯性脊髓炎**，MRI显示病变连续超过3个或以上椎体节段。**极后区综合征**：顽固性的**呃逆、恶心、呕吐**，无法用其他原因解释。**急性脑干综合征**。**急性间脑综合征**。**大脑综合征**伴典型的NMOSD脑部MRI病灶。2. 关键辅助检查。**A. 血液学检查 —— 检测AQP4-IgG抗体*** 这是诊断的“金标准”**。通过抽血检测血清中是否存在**水通道蛋白-4抗体**。**结果解读****AQP4-IgG阳性** + 1项核心临床症候 → 即可确诊NMOSD。**AQP4-IgG阴性或无法检测** → 需进入下一步更严格的诊断标准。**B. 影像学检查**。**头颅及全脊髓磁共振成像**：**脊髓MRI**：寻找特征性的**长节段横贯性脊髓炎**病灶。**头颅MRI**：寻找NMOSD特征性脑部病灶（如围绕第三、四脑室、导水管周围、极后区等）。**眼眶MRI**：评估视神经有无受累、肿胀或强化。**C. 其他支持性检查****腰椎穿刺**：检查脑脊液。NMOSD患者的脑脊液常显示细胞数增多（以白细胞为主），但寡克隆带阳性率较低（这与多发性硬化不同）。**光学相干断层扫描**：评估视神经纤维层厚度，客观量化视神经损伤程度。**视觉诱发电位**：评估视神经传导功能是否受损。3. 诊断标准（2015年国际共识诊断标准）。对于**AQP4-IgG阴性**的患者，诊断要求更严格，必须满足以下所有条件：1. **至少2项核心临床症候**发生，并符合以下所有要求：其中至少1项是视神经炎、长节段横贯性脊髓炎或极后区综合征。这些症候具有**空间多发性**（累及不同部位）。符合NMOSD的MRI特征。2. **排除其他疾病4. 鉴别诊断（非常重要）在确诊前，必须排除症状相似的其他疾病，尤其是：**多发性硬化****MOG抗体相关疾病**其他自身免疫性疾病（如系统性红斑狼疮、干燥综合征合并神经系统损害）感染性、代谢性或肿瘤性疾病**给您的核心建议：**如果您或家人出现上述可疑症状（如突发严重视力下降、肢体麻木无力、顽固性呃逆等），**请立即前往正规医院的【神经内科】就诊**，部分大型医院设有专门的“神经免疫”亚专业。眼科医生会协同处理视神经炎的部分。诊断过程需要时间，医生会像侦探一样，一步步收集证据，最终做出准确判断。早期、正确的诊断是启动有效治疗、预防严重残疾复发的关键。**请注意：** 以上信息仅为医学科普，不能替代执业医师的面对面诊疗。请务必在专业医生指导下进行所有检查与诊断。

**2、NMOSD与多发性硬化有什么区别？**

您好，很高兴能以专业医师的身份为您解答这个问题。视神经脊髓炎谱系疾病（NMOSD）和多发性硬化（MS）是两种不同的中枢神经系统自身免疫性脱髓鞘疾病，它们在过去常被混淆，但现在已明确是两种独立的疾病。理解它们的区别，对于诊断、治疗和预后都至关重要。以下是两者的核心区别：### 核心区别概览| 特征 | 视神经脊髓炎谱系疾病 (NMOSD) | 多发性硬化 (MS) || **核心靶点** | **星形胶质细胞**（特别是水通道蛋白-4，AQP4） | **髓鞘**（包裹神经纤维的“绝缘层”）和少突胶质细胞 || **致病抗体** | **AQP4-IgG阳性**（约70-80%患者），这是诊断的关键标志物。 | 无特异性抗体。诊断主要依靠临床和影像学标准。 || **主要攻击部位** | **视神经**和**脊髓**（尤其是中央部分，可导致严重横贯性脊髓炎）。也可累及脑干、间脑等特定脑区。 | 中枢神经系统**白质**的任何部位，包括大脑、小脑、脑干、脊髓和视神经。病灶更弥散。 || **病理本质** | 更严重、更广泛的炎症、坏死和血管病变，可导致组织**空洞形成**和永久性损伤。 | 局灶性炎症和脱髓鞘，后期可伴有轴索损伤和胶质增生（瘢痕形成）。 || **发病机制** | 主要由**体液免疫**（抗体介导）驱动。 | 主要由**细胞免疫**（T细胞、B细胞等）驱动。 || **脑脊液检查** | 白细胞可能显著升高（>50个/mm³），寡克隆带**通常为阴性**（阳性率<30%）。 | 白细胞轻度升高，**寡克隆带阳性率高**（>80%）。 || **磁共振成像 (MRI) 特征** | **脊髓病灶**：长节段（通常≥3个椎体节段）、中央型、水肿明显。**脑部病灶**：多位于AQP4高表达区（如延髓最后区、丘脑、下丘脑、脑室周围）。 | **脊髓病灶**：短节段（通常<2个椎体节段）、偏侧、局灶性。**脑部病灶**：多位于侧脑室周围、皮层下、胼胝体、小脑、脑干（“ Dawson手指 ”征是典型表现）。 || **疾病进程** | 多为**复发型**，发作常更严重，且**缓解不完全**，残疾累积快。罕见原发进展型。 | 分为**复发缓解型 (RRMS)** 和**进展型 (PMS，包括原发进展和继发进展)**。 || **发作严重性** | **通常非常严重**，可导致单眼或双眼在数小时至数天内视力急剧下降至失明，或严重截瘫、感觉丧失、大小便障碍。 | 发作程度相对多样，可从轻微到严重，但单次发作的严重程度**通常低于NMOSD**。 || **治疗策略** | **急性期**：大剂量激素冲击，效果不佳时需尽早进行**血浆置换**或免疫吸附。**缓解期（预防复发）**：使用**B细胞耗竭剂**（如利妥昔单抗、伊奈利珠单抗）、**补体抑制剂**（如依库珠单抗）或免疫抑制剂（如吗替麦考酚酯、硫唑嘌呤）。**多数MS一线药物（如干扰素、特立氟胺、芬戈莫德）对NMOSD无效甚至可能加重病情。** | **急性期**：大剂量激素冲击。**缓解期**：药物选择广泛，包括干扰素、特立氟胺、芬戈莫德、富马酸二甲酯、克拉屈滨、B细胞耗竭剂（如奥法妥木单抗）、S1P受体调节剂等。 || **预后** | 若不进行有效预防，反复发作可导致**不可逆的严重神经功能残疾**（如失明、瘫痪）。早期诊断和强效预防治疗可极大改善预后。 | 个体差异大。RRMS患者经规范治疗可长期稳定。进展型MS则面临持续的神经功能衰退。总体而言，长期残疾风险通常低于未经治疗的NMOSD。 |### 给患者的通俗解释。您可以这样理解：- **MS** 像是“游击战”，免疫系统错误地攻击神经的“绝缘皮”（髓鞘），在脑和脊髓的多个地方造成小范围的、相对表浅的破坏。症状多样，但单次破坏力相对有限。- **NMOSD** 更像是“精准轰炸”，免疫系统产生了专门攻击“后勤支持细胞”（星形胶质细胞，特别是AQP4蛋白）的抗体。一旦攻击，就会在视神经和脊髓的核心区域造成**大面积、深度的炎症和坏死**，因此单次发作就可能导致非常严重的视力丧失或瘫痪。### 最重要的临床提示。1. **鉴别诊断至关重要**：因为两者的治疗方案有根本性不同。用错药（如将NMOSD误诊为MS并使用某些MS药物）可能导致NMOSD复发更频繁、更严重。2. **检测AQP4-IgG是金标准**：对于怀疑脱髓鞘病的患者，尤其是以严重视神经炎或长节段脊髓炎起病的，必须进行血清**AQP4-IgG**检测。部分AQP4抗体阴性的患者，还可能检测**MOG-IgG**，这代表了另一类疾病（MOGAD）。3. **治疗目标不同**：NMOSD的治疗核心是**强效预防复发**，因为每次复发都可能带来灾难性后果。MS的治疗则兼顾控制复发和延缓疾病进展。**总结**：虽然NMOSD和MS都可能影响视神经和脊髓，导致视力下降、肢体无力等症状，但它们是“病根”完全不同的两种病。NMOSD是由特异性抗体介导的、攻击更猛烈、更易致残的疾病。准确的诊断是获得正确、有效治疗的第一步。如果您或您的家人正在被相关问题困扰，**请务必前往正规医院的神经内科或神经免疫专科门诊就诊**，由医生进行全面的评估（包括病史、神经系统查体、MRI和血液/脑脊液检测），以获得明确的诊断和个体化的治疗方案。希望以上解答对您有帮助。此信息仅为医学科普，不能替代专业医疗建议。

**3、AQP4抗体阴性是否能诊断为NMOSD？**

您好，这是一个非常重要且专业的问题。作为您的医师，我将为您详细解释。**核心答案：AQP4抗体阴性，不能完全排除NMOSD的诊断。**AQP4抗体是诊断**视神经脊髓炎谱系疾病** 的一个关键生物标志物，但它并非诊断的唯一标准。目前的诊断是一个综合性的过程，需要结合临床表现、影像学特征和其他辅助检查。以下是详细的专业解释：### 1. AQP4抗体的角色*	**高特异性**：AQP4抗体阳性对NMOSD具有很高的特异性（>99%），意味着如果检测结果为阳性，几乎可以肯定诊断为NMOSD。*	**但非100%敏感**：其敏感性约为70-80%。这意味着，**约有20-30%的NMOSD患者血清中检测不到AQP4抗体**。这部分患者被称为“AQP4抗体阴性NMOSD”。### 2. 如何诊断AQP4抗体阴性的NMOSD？根据最新的国际诊断标准（2015年IPND标准），即使AQP4抗体阴性，只要满足以下条件，仍可诊断为NMOSD：**必须同时满足以下两点：**1. **至少一项核心临床症候**： *	**视神经炎**：急性视力下降、视野缺损、眼球转动痛。 *	**急性脊髓炎**：肢体无力、感觉障碍（常伴有痛性痉挛）、大小便功能障碍。 *	**极后区综合征**：无法用其他原因解释的顽固性呃逆、恶心、呕吐。 *	**急性脑干综合征**。 *	**症状性发作性睡病或急性间脑临床综合征**（伴有典型的间脑MRI病灶）。 *	**大脑综合征**（伴有典型的NMOSD大脑MRI病灶）。2. **通过MRI检查证实病灶符合NMOSD的特征**，并且**排除其他可能的疾病**。 *	**视神经**：MRI显示长节段（通常超过视神经长度的1/2或连续超过3个椎体节段）的脊髓炎病灶。*	**脊髓**：MRI显示长节段横贯性脊髓炎病灶（通常连续超过3个椎体节段）。*	**脑部**：存在特定的病灶模式（如极后区、丘脑、下丘脑、脑室周围等处的病灶）。### 3. 另一个重要抗体：MOG抗体。对于AQP4抗体阴性的患者，**必须检测MOG抗体**。*	**MOG抗体相关疾病** 是一种独立的疾病实体，其临床表现与NMOSD有重叠（如视神经炎、脊髓炎），但治疗反应和预后可能与典型的AQP4抗体阳性NMOSD有所不同。*	区分AQP4抗体阴性NMOSD和MOGAD对于选择治疗方案和判断预后至关重要。### 4. 诊断流程总结。当怀疑NMOSD时，诊断思路如下：1. **临床表现评估**：是否存在核心临床症候。2. **抗体检测**：*	首选检测 **AQP4抗体**。*	若为阴性，则必须检测 **MOG抗体**。3. **影像学检查**：进行头颅和脊髓的MRI增强扫描，寻找特征性病灶。4. **鉴别诊断**：需要排除其他疾病，如多发性硬化、系统性自身免疫病（如狼疮、干燥综合征）引起的神经系统损害、感染、血管病、肿瘤等。### 给您的建议。如果您或您关心的患者正在面临这个问题，请务必：1. **寻求专科诊疗**：在**神经内科**或**神经眼科**专科医师指导下进行全面的评估。2. **完善关键检查**：确保已完成AQP4抗体、MOG抗体检测以及高质量的神经系统MRI检查。3. **不要仅凭单一抗体阴性就否定诊断**：临床症候和影像学特征同等重要。AQP4抗体阴性的NMOSD仍然是需要积极治疗的严重自身免疫性疾病。4. **明确诊断是治疗的基础**：准确的诊断分型（AQP4阳性NMOSD、AQP4阴性NMOSD、MOGAD）直接关系到免疫抑制剂（如利妥昔单抗、吗替麦考酚酯等）的选择和治疗策略的制定。**总结：AQP4抗体阴性不能作为排除NMOSD的依据。诊断需要由经验丰富的神经科医生结合临床表现、影像学特征（特别是长节段脊髓炎病灶）并排除MOG抗体相关疾病及其他鉴别诊断后，综合做出判断。**希望以上解释对您有帮助。具体情况请务必与您的主治医生详细沟通。祝您早日康复！

4、**NMOSD急性期的治疗方案有哪些？**

您好，我是您的医生。关于视神经脊髓炎谱系疾病（NMOSD）急性期的治疗，这是一个非常关键且需要紧急处理的问题。其核心目标是**快速抑制强烈的免疫炎症反应，减轻对中枢神经系统的攻击，最大限度地挽救神经功能，减少永久性残疾**。治疗方案需要根据患者的具体情况（如发作严重程度、累及部位、水通道蛋白4抗体状态、既往治疗反应等）由神经内科或神经眼科医生个体化制定。主要方案如下：### 一线治疗（最常用，通常作为起始）1. **大剂量糖皮质激素冲击治疗** *	**方案**：通常采用**甲泼尼龙**静脉滴注，每日500mg至1000mg，连续3-5天。 *	**作用**：强效、快速地抑制炎症，是急性期治疗的基石。*	**后续**：冲击治疗后，会改为口服泼尼松，并遵循医嘱在数周至数月内缓慢减量，以避免反跳和副作用。2. **血浆置换***	**适用情况**：通常用于以下情况：*	对激素冲击治疗反应不佳（治疗5天后症状无改善或仍在加重）。*	发病时症状非常严重（如严重视力丧失、截瘫、呼吸肌受累）。*	因某些原因不能使用大剂量激素。*	**方案**：在1-2周内进行5-7次置换。其原理是直接清除血液中的致病性抗体（如AQP4-IgG）和炎症因子。*	**证据**：多项研究证实，早期联合血浆置换能显著改善重症患者的远期神经功能恢复。### 二线或联合治疗。当一线治疗效果不理想，或患者情况特殊时，会考虑以下方案：3. **静脉注射免疫球蛋白**	**方案**：按每日每公斤体重0.4g的剂量，静脉滴注，连续5天。*	**作用机制**：通过多种途径调节免疫系统，可能中和致病抗体。*	**适用情况**：常用于对激素和血浆置换有禁忌或效果不佳的患者，或在儿童、孕妇等特殊人群中考虑使用。4. **免疫抑制剂**	**药物**：如**环磷酰胺**（静脉冲击）。*	**作用**：在急性期作为强化治疗的一部分，尤其对于反复发作或难治性病例，能更强力地抑制免疫细胞。*	**注意**：因其潜在的副作用（如骨髓抑制、感染风险），需在严密监测下使用。### 急性期治疗的关键原则*	**尽早开始**：治疗越早，神经功能恢复的可能性越大。“时间就是神经”。*	**足量足疗程**：激素冲击需足量，减量过程必须平缓，切忌过快停药。*	**个体化评估**：医生会综合评估病灶部位（视神经、脊髓、脑干）、严重程度、抗体状态等选择最合适的方案。*	**支持与康复**：在免疫治疗的同时，针对疼痛、痉挛、尿潴留等症状给予对症支持治疗，并尽早开始康复训练。*	**衔接预防期治疗**：急性期控制后，**必须立即启动并长期坚持预防复发的免疫抑制治疗**（即“缓解期治疗”），这是防止再次发作、保护神经功能的核心。常用药物包括利妥昔单抗、萨特利珠单抗、伊奈利珠单抗、吗替麦考酚酯、硫唑嘌呤等。**重要提醒**：以上信息为医学知识科普，不能替代执业医师的面对面诊疗。NMOSD急性期属于神经科急症，如果您或家人出现疑似症状（如突发视力急剧下降、肢体麻木无力、大小便障碍等），**请立即前往有条件的医院神经内科急诊就诊**，由专业医生进行评估和救治。如果您有更具体的临床情况需要咨询，建议携带所有病历资料与您的主治医生深入沟通。祝您早日康复！

**5、为什么NMOSD会出现顽固性呃逆、恶心和呕吐？**

根据您的问题，NMOSD（视神经脊髓炎谱系疾病）患者出现顽固性呃逆、恶心和呕吐，是本病一个具有特征性的临床表现。这通常与**延髓最后区**的受累直接相关。以下从医学角度为您详细解释：### 核心机制：延髓最后区受累。1. **解剖定位**：延髓最后区是位于脑干底部、第四脑室底部的一个特殊结构。它缺乏血脑屏障，是脑内的“化学感受器触发区”，主要负责监测血液和脑脊液中的毒素或代谢异常，并触发呕吐反射。2. **病理基础**：NMOSD是一种主要由水通道蛋白4抗体介导的自身免疫性疾病。AQP4蛋白在中枢神经系统的星形胶质细胞上高密度表达，而**延髓最后区正是AQP4表达最丰富的区域之一**。因此，它成为NMOSD免疫攻击的常见和早期靶点。3. **炎症与损伤**：当AQP4抗体攻击此区域的星形胶质细胞时，会引起局部炎症、水肿和组织损伤。这种炎症性病灶直接刺激了最后区的神经元网络，导致其功能异常亢进，持续发放“异常信号”。### 导致症状的神经通路- **顽固性呃逆**：通常由刺激延髓的“呃逆反射弧”引起。最后区的炎症可能直接或间接影响控制膈肌和肋间肌的神经核团（如孤束核、网状结构等），导致膈肌阵发性、不自主的痉挛收缩。- **恶心和呕吐**：最后区本身是呕吐中枢的上游关键触发器。其持续受到炎症刺激，会不断向孤束核和呕吐中枢发送信号，从而产生持续的恶心感和难以控制的呕吐。### 临床意义。1. **特征性症状**：在NMOSD中，**顽固性呃逆和呕吐常可作为首发或孤立症状出现**，有时甚至早于视神经炎或脊髓炎数周或数月。因此，对于不明原因的顽固性呃逆/呕吐，尤其是对常规止吐治疗反应不佳时，神经科和眼科医生会高度警惕NMOSD的可能性。2. **疾病活动标志**：这些症状的出现或加重，往往提示延髓病灶活动，是疾病复发或活动的重要临床标志之一。3. **鉴别诊断**：需要与其他引起类似症状的疾病鉴别，如颅内压增高、其他脑干脑炎、代谢紊乱等。头颅磁共振成像（MRI）是关键的鉴别手段，NMOSD在延髓最后区常可见到特征性的**点状或斑片状长T2信号病灶**。### 总结。简单来说，NMOSD患者出现顽固性呃逆、恶心、呕吐，**根本原因是自身免疫攻击了脑干中控制呕吐反射的关键“开关”——延髓最后区**。这不仅是患者痛苦的症状来源，也是诊断和评估NMOSD病情活动的重要线索。**重要提示**：如果您或您所知的患者正在经历这些症状，尤其是伴有视力下降、肢体麻木无力、大小便障碍等其他神经功能缺损时，**务必尽快就医于神经内科或眼科**，进行血清AQP4抗体检测、头颅及脊髓MRI等检查，以便尽早明确诊断并启动免疫抑制治疗，防止不可逆的神经功能损伤。

**6、如何有效预防NMOSD复发？**

作为专业医师，我理解您对NMOSD（视神经脊髓炎谱系疾病）复发的担忧。NMOSD是一种自身免疫性疾病，主要影响视神经和脊髓，预防复发是长期管理的关键。以下是基于当前医学指南的核心建议：### **1. 核心预防策略：长期免疫抑制治疗**NMOSD的复发预防**必须依赖规范的药物治疗**，患者**不可自行停药或减量**。常用方案包括：- **单克隆抗体**：如依库珠单抗（eculizumab）、萨特利珠单抗（satralizumab）、伊奈利珠单抗（inebilizumab），这些是国际指南推荐的一线选择，可特异性抑制免疫攻击。- **传统免疫抑制剂**：如吗替麦考酚酯、硫唑嘌呤、甲氨蝶呤等，需定期监测血常规和肝肾功能。- **利妥昔单抗**：针对B细胞的靶向治疗，对部分患者有效。**关键提示**：药物选择需由神经科或眼科医生根据您的抗体状态（如AQP4-IgG是否阳性）、既往复发频率、合并疾病等个体化制定。### **2. 密切监测与定期随访**- **定期复查**：每3-6个月进行神经眼科评估（包括视力、视野、眼底检查）和脊髓MRI检查，即使无症状也需坚持。- **识别复发征兆**：如突发视力下降、眼痛、肢体麻木无力、排尿障碍等，需**立即就医**。### **3. 生活方式与风险规避**- **避免诱发因素**：感染（尤其是呼吸道/肠道感染）、疲劳、应激、疫苗接种（部分活疫苗可能诱发复发，接种前需咨询医生）。- **谨慎处理合并疾病**：如合并其他自身免疫病（如系统性红斑狼疮），需多学科协同管理。- **孕期管理**：计划怀孕前需与医生调整用药方案（部分药物有致畸风险），孕期及产后需严密监测。### **4. 患者自我管理**- **记录症状日记**：详细记录任何新发或加重的症状，复诊时提供信息。- **保持健康习惯**：均衡营养、适度锻炼（避免过度劳累）、戒烟限酒。### **重要提醒**- NMOSD的复发可能导致不可逆的神经损伤，**预防远比治疗复发更重要**。- 所有治疗调整都需在医生指导下进行，切勿听信非正规渠道的“偏方”或保健品宣传。- 若您正在备孕、怀孕或哺乳，请务必告知医生，以调整安全方案。如果您有具体用药疑问或新发症状，请及时联系您的主治医生进行个体化评估。希望这些信息能帮助您更好地管理疾病，祝您健康！

**7、目前NMOSD缓解期的一线预防药物有哪些？**

根据目前国内外权威指南（如《中国视神经脊髓炎谱系疾病诊断与治疗指南》等），NMOSD（视神经脊髓炎谱系疾病）缓解期的一线预防治疗药物主要包括以下几类，其核心目标是**降低复发风险、保护神经功能**：### 一、 核心一线药物（基于大型临床试验证据）1. **单克隆抗体***	**萨特利珠单抗（Satralizumab）**：一种靶向白介素-6受体（IL-6R）的人源化单克隆抗体。通过皮下注射给药，可显著降低AQP4-IgG阳性及阴性患者的复发风险。是目前国内外指南推荐的一线选择。*	**伊奈利珠单抗（Inebilizumab）**：一种靶向CD19的B细胞耗竭剂。通过静脉输注给药，主要耗竭B细胞，从而减少致病性抗体的产生。尤其对AQP4-IgG阳性患者疗效明确，也是一线推荐。*	**依库珠单抗（Eculizumab）**：一种靶向补体C5的单克隆抗体。通过强效抑制补体级联反应发挥作用，对AQP4-IgG阳性患者预防复发效果非常显著，但需注意其有较高的脑膜炎球菌感染风险，接种疫苗和预防性抗生素是强制要求。2. **免疫抑制剂***	**吗替麦考酚酯（霉酚酸酯，MMF）**：通过抑制T、B淋巴细胞增殖发挥作用。疗效确切，是传统免疫抑制剂中证据等级较高的一线选择，尤其适用于AQP4-IgG阳性患者。需监测血常规和肝肾功能。*	**硫唑嘌呤（AZA）**：通过干扰DNA合成抑制淋巴细胞。同样是一线口服免疫抑制剂，常与泼尼松联用。使用前建议检测TPMT基因型或活性，以指导剂量并减少骨髓抑制风险。*	**甲氨蝶呤（MTX）**：对于不能耐受上述药物或有生育考虑的患者，可作为备选的一线口服药物。### 二、 通常与免疫抑制剂联用的基础治疗*	**口服糖皮质激素（如泼尼松）**：在启动上述免疫抑制剂或单抗治疗的初期，通常需要联用中小剂量激素，并**在病情稳定后非常缓慢地减量**（例如每1-3个月减2.5-5mg），直至最小有效剂量或停用。**切忌快速减停**，否则易导致复发。### 三、 重要原则与个体化选择。1. **AQP4-IgG状态是关键**：治疗方案的选择强烈依赖于患者血清AQP4-IgG是否为阳性。上述单抗类药物对阳性患者疗效数据更充分。2. **治疗目标**：实现**临床无复发**（至少年复发率降低50%以上，理想状态为零复发）和**影像学无活动病灶**。3. **个体化决策**：选择需综合考虑：*	药物可及性与医保政策*	患者的年龄、合并症（如感染、肿瘤风险）*	生育计划*	对给药途径（口服、皮下注射、静脉输注）的偏好与依从性*	药物潜在副作用及监测要求4. **监测与随访**：无论使用何种方案，均需**定期随访**（通常每3-6个月），评估神经功能、复发情况、药物副作用（如感染、肝肾功能、血象等）及进行必要的影像学检查。### 总结。目前，**萨特利珠单抗**和**伊奈利珠单抗**因其高效和相对可控的安全性，已成为国际公认的一线首选。传统免疫抑制剂如**吗替麦考酚酯**和**硫唑嘌呤**联合小剂量激素，仍然是重要的基础一线治疗，尤其在经济条件或药物可及性受限时。**重要提示**：此信息仅为医学知识科普，不能替代执业医师的面对面诊疗。具体的治疗方案必须由您的主治神经内科或神经眼科医师，根据您的全面评估结果（包括抗体状态、既往治疗反应、全身情况等）来制定。请勿自行调整或更改药物。如果您有具体的用药选择困惑或副作用担忧，建议整理好您的病史资料，与您的主治医生进行深入沟通。

**8、新型的单抗类药物与传统免疫抑制剂相比，在治疗NMOSD中各自的优缺点是什么？**

您好，很高兴能以专业医师的身份为您解答关于视神经脊髓炎谱系疾病（NMOSD）治疗药物选择的问题。这是一个非常关键且前沿的领域，您的提问非常精准。首先，需要明确一个核心概念：NMOSD是一种由水通道蛋白4（AQP4）抗体介导的、以中枢神经系统炎性脱髓鞘为主的自身免疫性疾病。其治疗分为**急性期治疗**（大剂量激素、血浆置换等以控制发作）和**预防复发治疗**（即您问的长期维持治疗）。您所问的“新型单抗类药物”和“传统免疫抑制剂”都属于预防复发治疗的范畴。下面我将从作用机制、疗效、安全性、使用便利性等多个维度，对两者进行系统比较：### 一、 传统免疫抑制剂**代表药物**： 硫唑嘌呤、吗替麦考酚酯（骁悉）、甲氨蝶呤、环磷酰胺（后两者多用于难治或重症病例）、他克莫司等。**优点**：1. **历史悠久，临床经验丰富**： 如硫唑嘌呤、吗替麦考酚酯已使用数十年，医生对其长期疗效、副作用谱非常熟悉。2. **广谱免疫抑制**： 通过抑制T细胞、B细胞增殖等多种免疫环节起效，对部分AQP4抗体阴性的NMOSD患者也可能有效。3. **口服给药，相对方便**： 多为口服制剂，患者无需频繁住院。4. **价格相对低廉**： 多数已进入国家医保目录，长期治疗的经济负担较轻。**缺点**：1. **起效慢**： 通常需要3-6个月才能达到最佳预防效果，在此期间患者仍有复发风险，常需与口服激素重叠使用。2. **疗效非特异性**： 并非针对NMOSD的核心发病机制（AQP4-IgG/补体/IL-6通路），因此预防复发的效力**中等**，年复发率降低约50-70%。3. **副作用较多，需密切监测**：*	**骨髓抑制**： 导致白细胞、血小板减少。*	**肝肾功能损伤**。*	**感染风险增加**（如带状疱疹、肺部感染）。*	**潜在致癌风险**（如淋巴瘤、皮肤癌，风险与剂量和时长相关）。*	**致畸性**，育龄期女性需严格避孕。4. **个体差异大**： 疗效和副作用受患者代谢基因型（如TPMT酶活性对硫唑嘌呤的影响）等因素影响，需要个体化调整剂量。### 二、 新型单克隆抗体药物。这类药物是NMOSD治疗领域的革命性突破，它们像“生物导弹”一样精准作用于发病通路中的关键靶点。**代表药物**1. **靶向补体C5的**： **依库珠单抗**（Eculizumab） - 全球首个获批用于AQP4抗体阳性NMOSD的药物。2. **靶向IL-6受体的**： **萨特利珠单抗**（Satralizumab） - 可皮下注射，同时对AQP4抗体阳性和阴性患者有效。3. **靶向CD19的B细胞耗竭剂**： **伊奈利珠单抗**（Inebilizumab） - 耗竭产生抗体的B细胞。**优点**：1. **靶向精准，疗效卓越**： 直接阻断导致髓鞘和神经元损伤的核心环节（补体激活、IL-6炎症信号、B细胞）。临床试验显示，它们能将年复发率降低**74%-94%**，疗效显著优于传统免疫抑制剂。2. **起效迅速**： 通常在首次给药后即开始起保护作用，能快速降低复发风险。3. **安全性相对更优**（靶向性强带来的）：*	没有传统免疫抑制剂的广泛骨髓抑制、肝肾功能毒性。 *	不具传统意义上的致畸性（但妊娠期使用数据仍有限，需谨慎评估）。4. **用药方案明确**： 有固定的负荷剂量和维持剂量方案，无需像传统药物那样频繁调整剂量。**缺点**：1. **给药方式不便**： 均为静脉输注（依库珠单抗、伊奈利珠单抗）或皮下注射（萨特利珠单抗），需定期前往医院。2. **存在特有的、需严密防范的风险**：*	**感染风险**： 尤其是**脑膜炎球菌感染**（依库珠单抗相关风险极高，用药前必须接种疫苗甚至预防性使用抗生素）。所有单抗都会一定程度增加普通感染风险。*	**输液反应**。3. **价格极其昂贵**： 虽然部分药物已通过国家医保谈判大幅降价并进入医保，但年治疗费用仍然显著高于传统药物，且各地医保报销政策不一。4. **长期安全性数据仍在积累中**： 相较于有数十年使用历史的传统药物，单抗类药物的超长期（如10年以上）安全性仍需更多真实世界数据验证。5. **有明确的疗效人群限制**： 依库珠单抗和伊奈利珠单抗目前主要推荐用于**AQP4抗体阳性**的患者。萨特利珠单抗适用范围更广。### 总结与临床决策考量| 特性 | 传统免疫抑制剂 | 新型单抗类药物 || **核心机制** | 广谱免疫抑制 | **精准靶向**（补体/IL-6/B细胞） || **预防复发效力** | **中等** | **极高** || **起效速度** | 慢（3-6个月） | **快** || **主要给药途径** | 口服 | 静脉/皮下注射 || **常见副作用** | 骨髓抑制、肝损、感染 | **特定感染风险**（如脑膜炎球菌） || **长期数据** | **非常丰富** | 仍在积累 || **经济成本** | **低** | **非常高** |**作为医师，我们在为患者制定治疗方案时，会综合评估以下因素**：1. **疾病活动度与严重性**： 对于高复发频率、既往发作导致严重残疾（如失明、瘫痪）的**AQP4抗体阳性**患者，尤其是经济条件允许的情况下，会**强烈推荐优先使用新型单抗**，以最大程度地阻止不可逆的神经功能损伤。2. **抗体状态**： AQP4抗体阴性患者，萨特利珠单抗和部分传统免疫抑制剂仍是合理选择。3. **合并症与感染风险**： 患者是否有活动性感染、结核、肝炎等病史，是否适合接种必要疫苗。4. **生育计划**： 对于育龄期患者，需与生殖医学科、产科医生共同制定计划。5. **经济条件与医保政策**： 这是非常现实的决定因素。我们会帮助患者了解当地的医保报销细则。6. **患者意愿与生活便利性**： 患者能否接受定期去医院输液/打针，还是更倾向于口服用药。**结论**新型单抗类药物在**疗效和靶向性**上具有压倒性优势，是NMOSD治疗史上的里程碑，为患者带来了“零复发”的希望。传统免疫抑制剂则凭借其**经济性、口服便利性和丰富的临床经验**，在特定患者群体和医疗资源环境中仍有不可替代的价值。最终方案一定是医患双方在充分沟通上述利弊后，共同做出的个体化选择。建议您携带完整的病历资料，与您的神经内科或眼科专科医生进行深入讨论。希望以上解释对您有所帮助。祝您治疗顺利，病情稳定！

**9、长期使用预防NMOSD复发的药物，主要的副作用有哪些？**

您好，作为您的医生，很高兴能为您解答这个问题。您提出的问题非常关键，长期用药管理是NMOSD治疗中至关重要的一环，了解药物的潜在副作用，有助于我们更好地监测和配合，实现治疗获益的最大化。目前，用于预防NMOSD复发的主流药物主要包括几大类，它们的副作用谱各有特点。**请务必注意，以下信息仅为医学知识科普，绝不能替代您的主治医师根据您的具体情况（如肝肾功能、血常规基线、合并用药等）所制定的个体化方案和医嘱。任何用药调整都必须与您的医生充分沟通后进行。**以下是几类常用预防药物的主要副作用概述：#### 1. 免疫抑制剂。这类药物通过抑制免疫系统的过度活跃来发挥作用。*	**霉酚酸酯（骁悉）**：*	**常见**：胃肠道反应（如腹泻、恶心、腹痛）、白细胞减少、感染风险增加。*	**需要监测**：定期复查**血常规**和**肝肾功能**。育龄期女性需严格避孕，因有致畸风险。*	**硫唑嘌呤**：*	**常见**：骨髓抑制（导致白细胞、血小板减少）、肝毒性、胃肠道不适、流感样症状。*	**重要提示**：用药前需检测**TPMT酶活性**，以评估严重骨髓抑制的风险。需定期监测血常规和肝功能。*	**甲氨蝶呤**：*	**常见**：口腔溃疡、恶心、肝酶升高、骨髓抑制。*	**需要关注**：长期使用需警惕肺部毒性。服用时常需联合**叶酸**以减轻副作用。#### 2. 单克隆抗体（生物制剂）这类药物靶向性更强，直接作用于导致NMOSD的特定免疫环节。*	**萨特利珠单抗** 与 **依库珠单抗**：*	**核心风险**：**严重感染风险显著增加**，尤其是脑膜炎球菌感染。这是最需要警惕的副作用。*	**预防措施**：在开始治疗前，**必须接种脑膜炎球菌、肺炎球菌、流感疫苗等**（具体疫苗方案需医生制定）。*	**其他**：输液反应、头痛、上呼吸道感染等。依库珠单抗还有极少数发生严重溶血的报道。*	**利妥昔单抗***	**常见**：输液反应（发热、寒战等，尤其在首次输注时）、感染风险增加。*	**需要监测**：会导致B细胞耗竭，需定期监测**免疫球蛋白水平**和**血常规**，警惕迟发性中性粒细胞减少。#### 3. 皮质类固醇（激素）常用于急性期治疗或作为部分免疫抑制剂的“桥接”治疗，长期小剂量维持使用需非常谨慎。*	**长期副作用**：向心性肥胖、满月脸、血糖升高、高血压、骨质疏松、白内障、青光眼、情绪波动、失眠、感染风险增加等。### 给您的核心建议：1. **定期复查是生命线**：无论使用哪种药物，**严格遵循医嘱进行定期复查（如血常规、肝肾功能、尿常规、免疫球蛋白等）** 是早期发现和处理副作用最关键的一步。2. **主动沟通**：如果您出现任何不适，如持续发热、喉咙痛、异常出血或瘀伤、严重腹泻、视力变化等，请立即联系您的医疗团队。3. **疫苗接种**：在使用生物制剂或强效免疫抑制剂前，与医生讨论疫苗接种计划至关重要。4. **生活方式**：保持健康饮食，注意食品卫生（降低感染风险），根据身体状况进行适度活动，特别是使用激素时需注意钙和维生素D的补充以保护骨骼。治疗NMOSD是一个医患共同协作的长期过程。了解这些潜在风险，是为了更安全、更有效地进行治疗，从而最大程度地减少复发、保护神经功能。请务必与您的神经内科或眼科主治医师保持紧密联系。**免责声明：本回答仅为医学知识科普，不构成任何医疗建议或治疗方案。具体用药及管理请遵从您的主治医师的指导。**

**10、在NMOSD用药期间可以接种疫苗吗？**

**专业眼科医师解答：NMOSD用药期间疫苗接种注意事项**您好，作为您的医师，我理解您对疫苗接种安全的关切。NMOSD（视神经脊髓炎谱系疾病）患者在用药期间能否接种疫苗，**不能一概而论**，需要根据您的具体病情、用药方案和疫苗类型进行个体化评估。以下是关键考量因素：### **核心原则：平衡免疫抑制与感染风险**NMOSD的治疗药物（如利妥昔单抗、吗替麦考酚酯、硫唑嘌呤、托珠单抗等）大多通过抑制免疫系统来控制疾病活动。这可能导致两个问题：1. **疫苗效果可能降低**：免疫抑制可能削弱疫苗诱导保护性抗体的能力。2. **活疫苗风险**：接种活疫苗（如麻疹、风疹、水痘、带状疱疹活疫苗、口服脊髓灰质炎疫苗等）在免疫抑制状态下有引发疫苗相关感染的风险。### **一般性建议（需与您的主治医师最终确认）**#### **1. 灭活疫苗（安全性较高，通常推荐）**- **包括**：流感灭活疫苗、肺炎球菌疫苗、乙肝疫苗、HPV疫苗、COVID-19 mRNA/灭活疫苗、破伤风/白喉/百日咳疫苗等。- **建议**：- **时机**：尽量在免疫抑制剂剂量较低或用药间歇期接种（例如，利妥昔单抗治疗前4周或治疗后4-6个月抗体反应可能更好）。- **监测**：接种后可通过检测抗体滴度（如乙肝表面抗体、COVID-19中和抗体）评估免疫效果。- **强烈推荐**：NMOSD患者感染风险较高，接种**流感疫苗**和**肺炎球菌疫苗**对预防重症感染尤为重要。#### **2. 活疫苗（通常禁忌或严格限制）**- **包括**：麻疹-风疹-腮腺炎（MMR）、水痘、带状疱疹活疫苗（Zostavax）、黄热病疫苗等。- **建议**：- **绝对避免**在**高强度免疫抑制期**接种（如大剂量激素冲击、利妥昔单抗使用后3-6个月内）。- 如必须接种（如前往疫区），需由专科医师评估免疫状态，并可能需暂停免疫抑制剂数月。#### **3. 特殊疫苗注意事项**- **带状疱疹疫苗**：推荐接种**重组亚单位疫苗（Shingrix）**（非活疫苗），而非活疫苗Zostavax。- **COVID-19疫苗**：**强烈建议接种**（使用灭活或mRNA疫苗）。最佳时机通常为疾病稳定期、免疫抑制剂使用前或间隔期。部分患者接种后可能出现一过性免疫激活，但获益远大于风险。- **流感疫苗**：每年接种**灭活注射剂型**，避免使用鼻喷活疫苗。### **行动步骤建议**1. **与您的神经眼科或神经免疫专科医师沟通**：在接种任何疫苗前，务必告知医师您的全部用药（包括剂量、使用时间）和当前疾病活动情况。2. **优先疾病控制**：在NMOSD活动期（如近期复发），应先控制病情再考虑疫苗接种。3. **记录疫苗接种史**：保留完整的接种记录，便于医师评估。4. **注意防护**：即使接种疫苗，因免疫抑制状态，仍需保持戴口罩、勤洗手等防护措施。### **总结****可以接种疫苗，但必须谨慎规划。** 多数灭活疫苗在NMOSD稳定期、经医师评估后是安全且推荐的。活疫苗通常需避免。请勿自行决定，务必与您的主治团队共同制定接种计划，以在疾病控制与感染预防间取得最佳平衡。**此建议为通用医学知识科普，不替代个体化诊疗方案。请以您的主治医师的最终指导为准。**

**11、如何缓解NMOSD引起的长期神经性疼痛、肌肉痉挛或顽固性瘙痒？**

**专业医师解答：NMOSD相关神经性症状的管理策略**首先，请务必理解，视神经脊髓炎谱系疾病（NMOSD）是一种自身免疫性中枢神经系统疾病，其引起的神经性疼痛、肌肉痉挛和顽固性瘙痒属于**神经病理性症状**，源于免疫攻击导致的视神经、脊髓等部位的损伤。这些症状可能非常顽固，需要**神经科与疼痛科、康复科等多学科协作管理**，且治疗必须严格在专科医师指导下进行，**切勿自行调整用药**。以下为基于当前临床指南和循证医学的分层管理建议：### **一、 核心原则**1. **病因治疗是根本**：确保NMOSD本身处于稳定期，无新发炎症活动。这依赖于有效的**免疫抑制/调节治疗**（如利妥昔单抗、萨特利珠单抗、伊奈利珠单抗、吗替麦考酚酯、硫唑嘌呤等）。若疾病活动未控制，神经损伤持续，症状难以缓解。2. **综合治疗**：单一方法往往效果有限，需药物与非药物手段结合。3. **个体化治疗**：症状对药物的反应因人而异，需耐心尝试和调整。### **二、 分症状管理方案**#### **1. 神经性疼痛**- **一线药物**：- **加巴喷丁** 或 **普瑞巴林**：钙通道调节剂，是治疗神经病理性疼痛的基石。需从低剂量起始，缓慢滴定至有效剂量。- **三环类抗抑郁药**（如**阿米替林**、**去甲替林**）：小剂量即可起效，尤其对烧灼样、针刺样疼痛有效，但需注意其抗胆碱能副作用（口干、便秘、嗜睡）。- **二线或联合用药**：- **5-羟色胺和去甲肾上腺素再摄取抑制剂**（如**度洛西汀**、**文拉法辛**）：对合并情绪问题的患者尤其有益。- **局部用药**：如**利多卡因贴膏**，适用于局部性疼痛。- **阿片类药物**（如**曲马多**、**他喷他多**）：仅作为二线或三线选择，用于其他治疗无效的中重度疼痛，因有成瘾风险需严格监管。- **非药物治疗**：- **经皮神经电刺激**：对部分患者有效。- **心理治疗**：认知行为疗法等有助于应对慢性疼痛。 - **物理治疗**：保持关节活动度，防止挛缩。#### **2. 肌肉痉挛*- **一线药物**：- **巴氯芬**：GABA-B受体激动剂，是治疗脊髓相关性痉挛的首选口服药。需缓慢加量，突然停药可致反跳性痉挛加重。- **替扎尼定**：α2-肾上腺素能受体激动剂，可缓解痉挛并改善睡眠。- **二线或严重痉挛**：- **肉毒毒素A局部注射**：针对局灶性、目标明确的肌群痉挛，效果精确且全身副作用小。- **丹曲林钠**：作用于肌肉本身，但肝毒性风险需监测。 - **巴氯芬鞘内泵**：对于口服药无效的严重、广泛性痉挛，可通过植入泵将药物直接送入脑脊液，剂量小、效果好、全身副作用少。- **非药物治疗**：- **规律、温和的拉伸与物理治疗**：至关重要，可防止关节挛缩和肌肉缩短。- **矫形器**：如踝足矫形器，帮助维持功能位。#### **3. 顽固性瘙痒（神经性瘙痒）**- **重要提示**：这种瘙痒源于脊髓损伤，皮肤本身无异常，故常规抗组胺药（如西替利嗪）通常**无效**。- **首选药物**：- **加巴喷丁** 或 **普瑞巴林**：同样是治疗神经性瘙痒的一线选择，机制同神经性疼痛。- **其他可能有效的药物**- **阿米替林**。- **卡马西平** 或 **奥卡西平**（需注意监测血常规和电解质）。- **非药物治疗**：- **冷敷**：用凉毛巾或冰袋（包裹毛巾）敷于瘙痒区域。- **避免刺激**：穿着宽松、柔软的棉质衣物，避免过热、出汗。- **心理干预**：瘙痒常导致严重焦虑，形成“痒-抓-痒”恶性循环，行为疗法和放松训练有帮助。### **三、 重要提醒与建议**1. **立即就医指征**：若疼痛、痉挛、瘙痒突然**显著加重**，或伴有**新出现的无力、感觉丧失、视力下降、排尿困难**等，需立即就诊，排除NMOSD**急性复发**的可能。2. **记录症状日记**：详细记录症状发生的时间、部位、性质、强度（可用1-10分评分）、诱因及缓解因素，这对医生调整治疗方案极有帮助。3. **关注心理健康**：慢性神经性症状常导致抑郁、焦虑、睡眠障碍，积极寻求心理支持或药物干预是整体治疗的一部分。4. **生活方式**：保持均衡营养，适度活动（在康复师指导下），避免感染（可能诱发NMOSD复发和症状加重）。**总结**：管理NMOSD的神经性症状是一场“持久战”，需要您与神经科医生建立稳固的合作关系，通过**规范免疫治疗控制病根**，并**分层、联合、个体化地运用上述对症策略**，才能最大程度地改善生活质量。*注：以上信息为医学知识科普，不能替代执业医师的面对面诊疗。请务必与您的主治神经科医师详细讨论您的具体情况，制定专属治疗方案。*

**12、NMOSD导致的大小便功能障碍怎么办？**

您好，非常理解您对NMOSD（视神经脊髓炎谱系疾病）导致大小便功能障碍的担忧。作为医生，我需要首先强调：**这是一个需要神经内科和泌尿外科/康复科医生紧急评估和处理的严重医学问题，请务必尽快就医，切勿自行处理。**以下我将从专业角度为您解释这个问题以及目前的处理原则，希望能帮助您更好地与您的主治医生沟通。### 核心问题：为什么NMOSD会影响大小便？NMOSD是一种主要攻击中枢神经系统**星形胶质细胞**的自身免疫性疾病。其标志性抗体（AQP4-IgG）攻击的靶点，在脊髓中分布密集，尤其是在**胸、腰段的脊髓**。- **控制膀胱和直肠的“司令部”** 位于脊髓的腰骶段（圆锥、马尾神经）。- 当NMOSD的炎症病灶（纵向延伸的横贯性脊髓炎）累及这些部位时，就会破坏正常的神经信号传导。- 这会导致两种主要功能障碍：1. **膀胱功能障碍**：可能表现为**尿潴留**（感觉有尿但排不出，需要导尿）、**尿失禁**（不自主漏尿）、或两者交替的“矛盾性尿失禁”。2. **肠道功能障碍**：通常表现为**便秘**和/或**大便失禁**。### 怎么办？—— 综合管理策略：处理原则是 **“急性期治疗”与“长期康复管理”相结合**，目标是保护肾功能、提高生活质量、预防并发症。#### 第一步：紧急就医与全面评估（急性期及诊断期）1. **立即就诊科室**：**神经内科**（负责NMOSD原发病治疗）和**泌尿外科**或**康复医学科**（负责大小便功能评估与管理）。2. **关键检查**：- **尿动力学检查**：这是评估膀胱功能的“金标准”。可以明确膀胱是“收缩无力”还是“过度活动/痉挛”，以及尿道括约肌是否协调。这对制定精准治疗方案至关重要。- **泌尿系统B超**：测量**残余尿量**（排尿后膀胱内剩余的尿量）。残余尿过多是导致反复尿路感染和肾积水的元凶。- **肾功能检查**：定期抽血查肌酐、尿素氮，并做肾脏B超，监测长期影响。- **直肠指诊及肛门直肠测压**：评估直肠感觉、肛门括约肌张力及排便协调性。#### 第二步：针对性治疗与管理方案**A. 膀胱功能障碍的管理**1. **清洁间歇导尿**：如果确诊为**尿潴留**（残余尿量 > 100ml 或超过膀胱容量的1/3），这是**首选且核心**的管理方法。- **目的**：定期排空膀胱，降低膀胱内压力，预防肾积水和尿路感染。- **方法**：患者或家属学习使用一次性导尿管，每天定时导尿4-6次。- **优点**：最大程度保护上尿路（肾脏）安全，减少感染。2. **药物治疗**：- 对于膀胱**过度活动**（尿急、尿频、急迫性尿失禁）：可使用M受体拮抗剂（如索利那新、托特罗定）或β3受体激动剂（米拉贝隆）。- 对于**排尿困难**：可使用α受体阻滞剂（如坦索罗辛）松弛尿道。- **注意**：药物必须在医生评估后使用，错误用药可能加重尿潴留。3. **手术治疗**（适用于长期、顽固性病例）：- 膀胱扩大术、骶神经调节（膀胱起搏器）、尿道括约肌切开术等。**B. 肠道功能障碍的管理**1. **建立规律排便习惯**：尝试每天固定时间（如早餐后）排便，利用胃结肠反射。2. **饮食与水分调整**：增加膳食纤维、水分摄入，必要时使用温和的通便药物（如聚乙二醇、乳果糖）。3. **药物与辅助**：使用栓剂（如开塞露、比沙可啶栓）或进行**肛门直肠手指刺激**，诱发排便反射。4. **肠道训练计划**：在康复师指导下进行。#### 第三步：根本性治疗与长期随访- **NMOSD的免疫抑制治疗**：这是**治本**的关键。必须由神经内科医生制定长期的免疫抑制方案（如利妥昔单抗、萨特利珠单抗、伊奈利珠单抗、吗替麦考酚酯、硫唑嘌呤等），以预防新的脊髓炎发作，防止神经功能进一步恶化。- **多学科团队协作**：您需要神经内科、泌尿外科、康复科、甚至临床营养科的共同随访。- **预防并发症**：积极管理可极大降低**尿路感染、肾结石、肾衰竭、褥疮**等风险。### 给您的行动建议1. **立即预约**：联系您的神经内科主治医生，说明情况，请求进行泌尿系统相关评估或转诊。2. **记录症状日记**：详细记录每日排尿次数、尿量（如可能）、排尿感觉、漏尿情况、排便情况等，就诊时带给医生看。3. **不要害羞**：向医生坦诚告知所有症状，这是疾病的一部分，专业医生会理解并帮助您。4. **寻求支持**：可以咨询医院的康复科，康复治疗师能教您很多实用的膀胱和肠道管理技巧。**总结：NMOSD相关的大小便功能障碍是严重的，但通过及时的医疗干预、规范的神经保护治疗和系统的康复管理，是完全可以被有效控制和管理好的。请务必重视，并立即开始行动。**祝您早日得到有效管理，提高生活质量！

**13、NMOSD病情稳定后，应该如何进行科学、安全的康复训练？**

作为您的眼科医师，我必须首先强调：**NMOSD（视神经脊髓炎谱系疾病）的康复训练必须在神经内科或康复科医生全面评估并指导下进行，且需与神经免疫治疗同步。** 以下为基于当前医学共识的康复指导原则，**不可替代您的主治医生团队制定的个性化方案**。### **核心原则：安全第一，科学渐进，多学科协作**NMOSD是一种中枢神经系统自身免疫性疾病，主要累及视神经和脊髓。康复目标并非“治愈”疾病本身，而是 **最大限度地恢复和保留功能，预防并发症，提高生活质量**。### **康复训练的四大支柱**#### **1. 视力能康复（针对视神经炎后遗症）**- **低视力评估与辅助**：首先由眼科或低视力门诊进行专业评估。根据视力缺损（如中心暗点、视野缩小）情况，使用：- 光学辅助器具：如放大镜、望远镜。- 电子助视器：如电子屏幕放大器、语音软件。- 环境调整：改善家居照明、使用高对比度物品。- **视觉训练**：在治疗师指导下进行，可能包括：- 扫视与追踪训练：提高眼球运动控制能力。- 视野感知训练：学习利用剩余视野的技巧。- **注意**：训练强度需极低，避免视疲劳诱发复发。#### **2. 躯体功能康复（针对脊髓炎后遗症）**这是康复的核心，必须在**康复医师和物理治疗师**指导下进行。- **运动功能**： - **肌力训练**：针对无力肌群，以**等长收缩、非负重、低强度**为主。**严禁过度疲劳**，疲劳是NMOSD复发的重要风险因素。- **痉挛管理**：常见后遗症。包括药物（如巴氯芬）、牵伸、体位摆放、物理治疗（如冷热疗）。- **平衡与步态训练**：使用平行杠、助行器，在绝对安全环境下进行，严防跌倒。- **感觉障碍**：- 进行感觉再教育训练，如不同材质物品的辨别。- 加强皮肤护理，因感觉缺失易导致压疮、烫伤。- **膀胱与肠道功能**：- 制定定时排尿计划，学习间歇导尿技术（如必要）。 - 饮食调整、药物管理便秘。#### **3. 日常生活能力（ADL）与职业康复**- **作业治疗师**将帮助您： - 改造日常活动（穿衣、洗漱、烹饪）的方法和工具。- 进行手功能精细动作训练。- 评估工作环境，提供重返工作岗位的建议与适应性训练。#### **4. 疲劳管理与心理支持**- **疲劳**：是NMOSD最突出的症状之一，非普通劳累。- **能量节约技术**：学习将活动分段，安排休息优先级。- **有氧运动**：在医生许可下，进行**极低强度**的有氧活动，如温水游泳、卧位脚踏车。**心率与疲劳感是严格监控指标**。- **心理与认知**：- NMOSD常伴焦虑、抑郁及认知功能（如记忆力、注意力）下降。- 寻求心理科或神经心理科支持至关重要，可进行认知康复训练。### **【绝对禁忌与重要警告】**1. **避免过热**：严禁热水浴、桑拿、高温瑜伽等。**Uhthoff现象**（体温升高导致神经症状一过性加重）在NMOSD中极为常见。2. **严禁过度疲劳**：“今天练到位，明天躺三天”的模式极其危险，可能诱发免疫激活。**“无疲劳训练”是铁律**。3. **避免感染**：康复场所需清洁，注意保暖。感染是已知的复发重要诱因。4. **警惕新症状**：康复过程中出现任何**新发或加重的疼痛、麻木、无力、视力下降、排尿困难**，必须立即停止训练并联系您的主治神经内科医生，这可能是病情活动的信号。### **行动路线图**1. **获取全面评估**：联系您的神经内科主治医生，请求转介至**康复医学科**。2. **组建团队**：理想团队包括：神经内科医生、康复科医生、物理治疗师、作业治疗师、眼科/低视力医生、心理医生。3. **制定个性化计划**：与团队共同制定书面康复计划，明确目标、强度、频率和风险预案。4. **保持沟通与记录**：定期向团队反馈，记录训练后的感受和任何异常。**总结**：NMOSD稳定期的康复是一门精细的“平衡艺术”，是在不激发免疫系统的前提下，小心翼翼地挖掘神经系统的修复与代偿潜力。它需要极大的耐心、科学的指导和严格的自我观察。请务必在专业医疗团队的护航下开启您的康复之旅。祝您康复顺利！

**14、NMOSD能治愈吗？**

作为一名专业医师，我理解您对NMOSD（视神经脊髓炎谱系疾病）治疗前景的关切。目前，NMOSD尚无法被彻底“治愈”，但通过规范治疗，疾病可以得到有效控制，实现长期稳定，甚至达到无复发的状态。以下是关于NMOSD治疗的关键信息：1. **治疗目标**：现代治疗的核心是**控制病情、预防复发、减少神经功能损伤累积**，从而最大限度地保护视神经和脊髓功能，提高生活质量。2. **治疗方案**：*	**急性期治疗**：主要采用大剂量糖皮质激素冲击和血浆置换，目的是尽快抑制急性炎症、减轻发作导致的损伤。*	**缓解期预防治疗（维持治疗）**：这是**长期管理的关键**。目前已有多种靶向生物制剂获批，能显著降低复发风险（如针对水通道蛋白4抗体的药物）。此外，传统免疫抑制剂也常被使用。3. **预后与希望**：*	随着近年来靶向药物的出现，NMOSD的治疗已取得革命性进展。许多患者通过坚持长期、规范的预防治疗，可以长期不复发，稳定地工作和生活。*	早期诊断、尽早开始并坚持规范治疗至关重要，可最大程度避免不可逆的神经功能残疾（如视力严重下降、瘫痪等）。**重要建议**：NMOSD的管理需要个体化方案。请您务必在**神经内科或神经眼科专科医生**的指导下，进行定期随访、评估和调整治疗。切勿自行停药或更改方案。如果您是患者本人，请与您的主治医生保持密切沟通，共同制定最适合您的长期管理策略。虽然不能称为“治愈”，但实现疾病的长期稳定是完全可能的目标。

**15、NMOSD的长期预后怎么样？**

作为一名专业医师，我很理解您对NMOSD（视神经脊髓炎谱系疾病）长期预后的关切。这是一种罕见的自身免疫性疾病，主要影响视神经和脊髓，可能导致视力下降、肢体麻木无力、大小便障碍等症状。关于长期预后，我需要强调以下几点：1. **疾病特点**：NMOSD具有复发倾向，每次发作可能导致神经功能损伤累积，因此**早期诊断和规范治疗至关重要**。2. **治疗进展**：近年来，随着对疾病机制的深入了解，已出现多种靶向药物（如抗CD20单抗、补体抑制剂等），**显著降低了复发率，改善了长期预后**。3. **预后因素**：- **治疗依从性**：坚持长期免疫抑制治疗是减少复发的关键。- **发作严重程度**：首次发作后遗留的神经功能缺损程度会影响远期生活质量。- **并发症管理**：如疼痛、痉挛、膀胱功能障碍等的积极康复治疗。4. **长期管理目标**：- **减少复发**：通过药物将年复发率降至最低。- **功能保留**：通过康复训练最大限度保留神经功能。- **生活质量提升**：多学科团队（神经科、眼科、康复科等）协作管理。5. **重要提醒**：个体预后差异较大，需结合**具体抗体类型（如AQP4抗体阳性/阴性）、发作频率、治疗反应等因素综合评估**。**建议**：请务必在神经科或神经眼科专科医师指导下制定长期治疗计划，定期随访（包括临床评估、影像学及实验室检查），切勿自行调整药物。如果您能提供更多信息（如抗体状态、既往发作情况、当前治疗方案等），我可以给出更具针对性的分析。

**16、NMOSD患者可以怀孕生子吗？**

作为您的医生，我需要首先说明：**NMOSD（视神经脊髓炎谱系疾病）患者可以怀孕生子，但必须在神经科和产科医生的严密监测和科学管理下进行，以最大程度降低风险。**这是一个需要谨慎权衡的问题，以下是您需要了解的核心信息：### 1. 怀孕对NMOSD的影响：风险与机遇并存- **复发风险**：怀孕本身（尤其是孕早期和产后3-6个月）是NMOSD复发的高危时期。产后复发风险最高，可达30%-50%。这与孕期免疫状态剧烈变化有关。- **疾病活动性是关键**：如果怀孕前疾病已长期稳定（通常指**无临床复发≥1年**），孕期风险会显著降低。- **部分患者孕期反而稳定**：有研究显示，部分患者在孕中晚期（孕4-9个月）复发率可能降低，这得益于孕期免疫耐受状态。### 2. 妊娠前必须完成的准备工作（孕前咨询至关重要）在尝试怀孕前，您必须与**神经科医生和产科高危门诊医生**共同制定计划：- **评估疾病活动性**：确保病情稳定至少1年。- **调整治疗方案**：- **停用致畸药物**：如霉酚酸酯、甲氨蝶呤、环磷酰胺等，需提前至少3-6个月更换为孕期相对安全的药物。- **转换至孕期可用药物**：部分免疫抑制剂（如硫唑嘌呤、他克莫司）和小剂量激素在孕期可能被允许使用，需个体化评估。- **考虑使用生物制剂**：如**依库珠单抗**或**萨特利珠单抗**，这些新型靶向药物在孕期使用的数据正在积累，需与医生深入讨论获益与风险。- **进行全面检查**：包括眼科（视神经）、脊髓MRI、血液抗体（AQP4-IgG）滴度、肝肾功能等基线评估。### 3. 孕期与产后的管理要点- **严密监测**：孕期需定期进行神经科和产科随访，警惕任何新发的视力下降、肢体麻木无力、疼痛等症状。- **复发处理**：孕期若发生急性复发，**大剂量激素冲击**仍是首选治疗，对胎儿相对安全。血浆置换也可作为选项。- **分娩方式**：通常可经阴道分娩，除非有脊髓严重受累导致产力不足。剖腹产并非必须。- **产后关键期**：产后立即启动或加强预防治疗至关重要，以应对最高的复发风险。**母乳喂养**需要根据所用药物具体分析，部分药物（如激素、硫唑嘌呤）在乳汁中浓度很低，通常可以哺乳。### 4. 对胎儿的影响- NMOSD本身**不直接遗传**给胎儿（非传统遗传病）。- 主要风险来自**疾病活动导致的母体并发症**（如严重复发导致残疾）以及**部分药物的潜在影响**。在良好控制下，大多数患者可以诞下健康宝宝。### 给您的核心建议：1. **切勿自行尝试怀孕**：务必先与您的神经科医生进行孕前规划。2. **组建多学科团队**：确保您的医疗团队包括经验丰富的神经科医生、产科医生（最好是母胎医学专家）和新生儿科医生。3. **耐心等待最佳时机**：在疾病未得到稳定控制前，强烈建议采取可靠避孕措施。4. **保持积极但审慎的态度**：随着现代医学的进步，许多NMOSD患者已成功成为母亲。成功的关键在于 **“计划、预防和严密监测”**。**请立即预约您的神经科医生，进行正式的孕前咨询。** 医生会根据您的具体情况（如抗体状态、既往复发频率、器官受累情况、当前用药）为您制定最个体化的方案。*免责声明：以上信息为通用医学知识科普，不能替代您的主治医生的面对面诊疗意见。所有治疗决策必须与您的医疗团队共同做出。*

**17、孕期和产后如何管理疾病，以降低NMOSD复发风险？**

**专业医师建议：孕期及产后NMOSD（视神经脊髓炎谱系疾病）管理**您好，作为您的医师，我理解您对NMOSD在孕期和产后管理的担忧。NMOSD是一种自身免疫性疾病，主要攻击视神经和脊髓，孕期由于免疫状态变化，复发风险可能增加。以下是根据当前临床指南和循证医学证据提出的管理建议，请务必在神经科与产科医师共同监测下执行：### **一、孕前准备（计划怀孕阶段）**1. **疾病稳定期怀孕**：建议在病情稳定（无临床复发）≥12个月后再考虑怀孕，以降低孕期复发风险。2. **药物调整**：- **免疫抑制剂**：需提前调整药物。霉酚酸酯、甲氨蝶呤等有致畸风险，需停药至少3-6个月（遵医嘱替换为孕期相对安全的药物）。- **生物制剂**：利妥昔单抗（CD20单抗）可在孕前使用，但需在计划怀孕前4-6个月调整给药时间；依库珠单抗（补体抑制剂）需个体化评估。 - **糖皮质激素**：泼尼松在低剂量（如≤10mg/天）下相对安全，但需监测血糖、血压及骨质疏松。3. **多学科协作**：提前与神经科、产科、风湿免疫科医师共同制定孕产期管理方案。### **二、孕期管理**1. **免疫治疗维持**：- **核心药物**：小剂量泼尼松（5-10mg/天）或硫唑嘌呤可在孕期使用，需定期监测血常规、肝肾功能。- **生物制剂**：利妥昔单抗可谨慎用于中晚期孕期（权衡复发风险与胎儿暴露）。 - **避免使用**：环磷酰胺、霉酚酸酯等致畸药物。2. **复发监测**： - **症状预警**：如出现视力下降、肢体麻木无力、顽固性呃逆呕吐、尿潴留等，需立即就诊。- **定期评估**：每1-3个月进行神经科检查，必要时行MRI（避免造影剂，孕早期慎用）。3. **支持治疗**：- **维生素D补充**：维持骨骼健康，降低自身免疫活动风险。- **预防血栓**：孕晚期NMOSD患者可能活动减少，需评估血栓风险。### **三、产后管理（复发高风险期）**1. **产后免疫治疗重启**：- 产后1-3个月内复发风险最高，建议分娩后尽早恢复预防性治疗（如泼尼松联合免疫抑制剂）。- 母乳喂养期间药物选择：泼尼松、硫唑嘌呤、利妥昔单抗在乳汁中浓度低，通常可安全使用；具体需根据药物剂量、婴儿状况个体化评估。2. **哺乳与用药**：- 服药与哺乳时间间隔：如使用泼尼松，建议服药后4小时再哺乳。- 避免环孢素、甲氨蝶呤等通过乳汁分泌的药物。3. **情绪与休息**：- 产后抑郁可能加重免疫紊乱，需关注心理支持。- 保证睡眠，避免劳累，家人协助照顾新生儿。### **四、紧急情况处理**- 若孕期或产后出现疑似复发症状，**立即联系神经科医师**，可能需要：1. 大剂量甲强龙冲击治疗（孕期和哺乳期相对安全）。2. 血浆置换（孕期可慎用，尤其对激素不敏感者）。3. 静脉注射免疫球蛋白（IVIg）可作为替代选择。### **五、长期随访**- 产后至少每3个月复查一次，包括神经功能评估、血清AQP4抗体滴度（如有基础升高）、MRI（必要时）。- 后续生育计划需再次评估，NMOSD复发风险与抗体滴度、既往病史相关。**重要提示**：以上建议需根据您的具体病情（如AQP4抗体状态、既往复发频率、器官损伤程度）个体化调整。请勿自行停药或改药，务必与您的医疗团队保持密切沟通。祝您和宝宝健康平安！如有任何新症状，请及时就医。*（本回复基于2023年NMOSD国际共识及临床指南，具体治疗请遵循您的主治医师方案。）*

**18、NMOSD会遗传给下一代吗？**

根据您的问题，我将以专业医师的身份为您解答关于视神经脊髓炎谱系疾病（NMOSD）的遗传风险。**核心结论：NMOSD通常不被认为是典型的遗传性疾病，但存在一定的遗传易感性，且直接遗传给下一代的风险较低。**以下是更详细的分析：1. **遗传模式**：NMOSD不符合孟德尔遗传规律（如常染色体显性或隐性遗传）。这意味着它不会像某些单基因疾病那样，有明确的、高概率的遗传给子女的模式。2. **遗传易感性**：研究表明，NMOSD的发病与某些**人类白细胞抗原（HLA）基因型**相关，尤其是**HLA-DRB1*05:01**等位基因在AQP4抗体阳性的患者中较为常见。这意味着携带这些基因型的人，患NMOSD的风险可能比普通人群稍高，但**绝大多数携带这些基因型的人并不会发病**。3. **主要病因**：NMOSD本质上是一种**自身免疫性疾病**。其发病是**遗传易感背景、免疫系统异常（如产生针对水通道蛋白4的抗体）以及可能的环境触发因素（如感染）** 共同作用的结果。环境因素和免疫系统的随机错误在发病中起着关键作用，这解释了为什么即使有家族史，直系亲属的患病风险也远低于50%。4. **家族聚集性**：虽然罕见，但确实有极少数家族中出现多名NMOSD患者的报道。这提示了遗传因素在其中的作用，但这种情况非常少见。绝大多数NMOSD患者都没有家族史。**给您的具体建议：***	**风险评估**：如果您是NMOSD患者，您的子女患NMOSD的**绝对风险仍然很低**，远低于1%。目前不推荐对无症状的子女进行常规基因筛查。*	**关注症状**：重要的是让家庭成员（包括子女）了解NMOSD的**早期警示症状**，例如：*	严重的视力下降或眼痛。*	肢体麻木、无力或瘫痪。*	顽固性的恶心、呕吐或呃逆（可能累及延髓）。*	出现这些症状需要立即就医。*	**孕产期咨询**：如果您（患者）计划怀孕，**务必提前与您的神经科医生和产科医生进行详细规划**。因为孕期和产后是复发风险较高的时期，需要密切监测并可能调整治疗方案（许多NMOSD药物在孕期禁用或需提前更换）。*	**专业咨询**：如果家族中有多名成员患有自身免疫性疾病（如系统性红斑狼疮、干燥综合征等）或神经系统疾病，可以考虑咨询**遗传咨询师**，以获得更个体化的风险评估。**总结**：NMOSD具有轻微的遗传易感性，但**不会像遗传病那样直接“遗传”**。子女患病的风险很低，不必过度焦虑。当前的重点应是积极治疗控制病情、了解疾病知识、关注预警症状，并在计划生育时与专科医生紧密合作。*请注意，以上信息为医学知识科普，不能替代您的主治医师提供的个体化医疗建议。任何关于生育、遗传和治疗的决策，都应在与您的神经科医生充分沟通后做出。*

**19、NMOSD有什么饮食禁忌？**

您好，作为专业医师，很高兴能为您解答关于视神经脊髓炎谱系疾病（NMOSD）的饮食禁忌问题。首先，需要明确一个核心原则：**目前没有任何一种特定的饮食被临床研究证实可以治疗或根治NMOSD。** 治疗NMOSD的核心是规范的免疫抑制或免疫调节治疗（如使用单克隆抗体、激素、免疫抑制剂等），并需在神经内科或相关专科医生的指导下进行。然而，一个**健康、均衡、抗炎**的饮食模式，对于NMOSD患者维持整体健康、减少并发症、并可能间接辅助疾病管理具有重要意义。以下是基于医学原理和临床经验的饮食建议与禁忌：### 一、 建议遵循的饮食原则（宜）1. **抗炎饮食**： *	**核心**：多摄入富含抗氧化剂和Omega-3脂肪酸的食物，有助于减轻身体的慢性炎症状态。*	**推荐食物**： *	**蔬菜水果**：深色绿叶蔬菜（如菠菜、羽衣甘蓝）、浆果（蓝莓、草莓）、西兰花、番茄等。它们富含维生素C、E及多种植物化学物。*	**优质脂肪**：富含Omega-3的鱼类（如三文鱼、鲭鱼、沙丁鱼）、亚麻籽、奇亚籽、核桃。*	**全谷物**：燕麦、糙米、藜麦等，提供膳食纤维和B族维生素。2. **补充维生素D**： *	**重要性**：NMOSD患者常伴有维生素D缺乏，而充足的维生素D对免疫调节和骨骼健康至关重要。长期使用激素治疗更会增加骨质疏松风险。*	**来源**：在医生指导下进行血清水平检测，并通过日晒（避免暴晒）、食物（如强化牛奶、蛋黄、多脂鱼）或**医生处方的补充剂**来补充。3. **充足优质蛋白质**： *	**重要性**：维持肌肉力量，支持免疫系统正常功能，促进组织修复。*	**来源**：瘦肉、禽肉、鱼、蛋、豆类及豆制品。4. **充足饮水**：保证每日饮水量，有助于维持身体正常代谢。### 二、 需要谨慎或避免的饮食（忌/慎）1. **高盐、高糖、高饱和脂肪/反式脂肪食物**：*	**原因**：这类食物（如加工肉类、油炸食品、甜点、含糖饮料）会促进体内炎症反应，并增加肥胖、高血压、心血管疾病的风险，这些并发症会加重身体负担。*	**建议**：尽量减少或避免。2. **严格限制钠盐摄入（尤其在使用激素期间）**：*	**原因**：大剂量或长期使用糖皮质激素（如泼尼松）会导致水钠潴留，引起水肿、血压升高。控制盐分摄入至关重要。*	**建议**：烹饪时少放盐，避免咸菜、腊肉、罐头、酱油等高盐调味品和加工食品。3. **谨慎对待“增强免疫力”的保健品或补品**：*	**原因**：NMOSD是一种自身免疫性疾病，病理核心是免疫系统“过度攻击”自身组织。盲目服用声称能“增强免疫力”的产品（如某些灵芝孢子粉、蜂王浆、人参等），**可能刺激免疫系统，诱发疾病复发**。*	**核心原则**：**任何保健品、中草药或补品，在服用前都必须咨询您的主治医生**，确保其不会与现有治疗方案冲突或激活免疫系统。4. **注意食物安全，避免感染风险**： *	**原因**：患者因疾病本身或使用免疫抑制剂，抵抗力可能较低。*	**建议**：避免生食或未煮熟的肉类、海鲜、鸡蛋；确保食材新鲜，处理生熟分开，防止食源性感染。5. **关于特殊饮食法（如无麸质饮食、生酮饮食等）**：*	**目前没有证据支持这些饮食对NMOSD有特殊疗效。** 如果考虑尝试，务必先与您的医生和临床营养师详细讨论，评估其必要性和潜在风险，切勿自行开始，以免导致营养不良。### 三、 与药物相关的特别注意事项*	**激素（如泼尼松）**：需注意补充钙和维生素D以防骨质疏松；控制盐、糖摄入；可能增加食欲，需注意控制总热量，防止体重过快增加。*	**免疫抑制剂**：某些药物可能对肝功能有影响，需避免饮酒。具体请遵医嘱。### **总结与最终建议**1. **治疗优先**：饮食是**辅助**，绝不能替代正规的药物治疗和定期复诊。2. **个体化差异**：每位患者的病情、身体状况、用药方案不同，对食物的反应也可能不同。如果发现某种食物与不适或症状波动有关，可记录并告知医生。3. **咨询专业人士**：最理想的方案是，在您的主治医生（神经内科）指导下，咨询**临床营养科**的医生或营养师，他们可以根据您的具体情况（如用药、肝肾功能、体重、合并症等），制定一份个性化的饮食计划。4. **保持健康生活方式**：结合适度、力所能及的运动（如散步、瑜伽、游泳），保证充足睡眠，管理压力，对疾病稳定同样重要。**请务必与您的医疗团队保持密切沟通，将饮食管理作为综合治疗的一部分来规划。** 祝您健康！。

**20、面对NMOSD高复发风险和高致残率，我感到非常焦虑和抑郁，该怎么办？**

非常理解您此刻的心情。面对视神经脊髓炎谱系疾病（NMOSD）这种具有高复发风险和高致残率的疾病，产生焦虑和抑郁情绪是完全正常的反应。这不仅是心理上的压力，也是疾病本身可能对中枢神经系统（包括情绪调节区域）影响的一部分。请记住，您的感受是真实且重要的，而积极应对这些情绪是整体治疗中至关重要的一环。作为一名专业医师，我想从以下几个方面为您提供一些思路和建议：### 第一步：医学干预是基石——控制疾病本身。焦虑和抑郁的根源很大程度上来自于对疾病复发的恐惧。因此，**最根本的缓解方法是有效控制疾病活动**。1. **坚持规范治疗**：请务必与您的神经内科或眼科主治医生保持紧密联系。目前NMOSD已有多种有效的预防复发药物（如单克隆抗体、免疫抑制剂等）。确保您理解并遵从治疗方案，定期复查（包括血液学、影像学等），这是降低复发风险、从而减轻心理压力的最直接途径。2. **识别复发征兆**：与您的医生充分沟通，了解NMOSD复发的早期症状（如视力急剧下降、肢体麻木无力、顽固性呃逆呕吐、大小便障碍等）。掌握这些知识，并制定明确的“复发应急预案”（如知道何时、如何联系医院），能帮助您从“未知的恐惧”转变为“有准备的应对”，从而获得掌控感。### 第二步：正视并管理情绪——心理与精神支持1. **寻求专业心理帮助**：*	**心理咨询/治疗**：强烈建议您寻求具有慢性病或神经系统疾病患者辅导经验的心理咨询师或治疗师。他们可以帮助您：*	学习认知行为疗法技巧，调整对疾病和未来的灾难化思维。*	掌握正念、放松训练等方法，管理急性焦虑。*	处理因疾病带来的丧失感（如身体功能、生活计划等），重建生活意义。*	**精神科评估**：如果抑郁或焦虑情绪严重影响了您的日常生活、睡眠、食欲或治疗依从性，请务必进行精神科评估。医生可能会判断是否需要使用抗抑郁/抗焦虑药物来帮助您稳定情绪。**这并非意志薄弱的表现，而是像治疗高血压一样，是对一种医学状况的必要干预。**### 第三步：构建支持系统——您不是一个人在战斗1. **与家人朋友坦诚沟通**：让他们了解NMOSD是什么，以及它如何影响您（包括身体和情绪）。明确告诉他们您需要什么样的支持（是倾听、陪伴，还是具体的帮助）。2. **加入病友支持团体**：与有相似经历的人交流，可以获得独特的理解、实用的经验分享和情感共鸣。这能极大减少孤独感。可以咨询您的主治医院或在线寻找正规的病友社群。3. **与医疗团队保持开放沟通**：告诉您的神经科医生和眼科医生您的情绪困扰。他们是您整体健康的管理者，可以为您整合资源，或调整治疗策略以兼顾身心健康。### 第四步：生活方式的调整——在能力范围内找回掌控感1. **温和规律的运动**：在医生许可和身体条件允许下，进行如散步、瑜伽、太极等温和运动。运动是天然的抗抑郁剂，能改善情绪和睡眠。2. **均衡营养与充足睡眠**：保持健康饮食，避免感染（感染是常见复发诱因）。建立规律的睡眠习惯，这对神经修复和情绪稳定至关重要。3. **设定现实的目标与兴趣**：根据当前身体状况，调整对工作和生活的期望，设定一些短期、可实现的小目标。培养一些不受身体状况限制的兴趣爱好（如听书、音乐、手工等），将注意力部分转移到能带来愉悦感的事物上。### 重要提醒：*	**紧急情况**：如果您出现**自伤或自杀的念头**，请立即联系家人、朋友，或拨打心理援助热线（如全国希望24热线：400-161-9995），或直接前往医院急诊科。生命安全永远是第一位的。*	**NMOSD的治疗近年来已取得巨大进展**，许多患者通过有效的预防治疗，实现了长期不复发，保持了良好的生活质量。未来仍有更多新药和疗法在研发中。**总结一下：**您现在的焦虑和抑郁，是疾病挑战下的一种可理解的反应。请将它视为需要治疗的“症状”之一，而不仅仅是“心情不好”。**采取“生物-心理-社会”的综合模式来应对：***	**生物层面**：坚持NMOSD规范治疗，评估精神科用药可能。*	**心理层面**：接受专业心理干预。*	**社会层面**：积极利用家庭、病友和医疗团队的支持。您正在经历一场艰难的战斗，但请相信，现代医学和有效的支持策略能够帮助您同时管理好疾病和情绪。迈出寻求帮助的第一步，就是走向康复和更好生活质量的最重要一步。请务必与您的医疗团队深入讨论您的这些感受。

**Kimi**

1、**如何确诊NMOSD？**

\# NMOSD（视神经脊髓炎谱系疾病）确诊指南## 一、核心诊断标准（2015年国际共识标准）### 必备条件| 项目 | 具体要求 | **核心临床特征** | 至少出现以下1项：①视神经炎；②急性脊髓炎；③最后区综合征（顽固性呃逆/呕吐）；④急性脑干综合征；⑤症状性发作性睡病或急性间脑综合征；⑥症状性大脑综合征 | **AQP4-IgG检测** | 血清检测为诊断关键依据 |二、实验室确诊检查### 1. **血清AQP4-IgG抗体检测** 金标准- **检测方法**：细胞免疫荧光法（CBA）为首选，敏感性约70-80%- **临床意义**：阳性即可确诊NMOSD，无需其他条件### 2. **血清MOG-IgG抗体检测**- 用于鉴别MOGAD（MOG抗体相关疾病）- 约20-30%原诊为AQP4阴性NMOSD实际为MOGAD### 3. **脑脊液检查**- 细胞数升高（>5×10⁶/L，中性粒细胞为主）- 蛋白轻度升高- **寡克隆区带**：通常阴性（与MS的重要鉴别点）三、影像学特征。### 视神经MRI。典型表现：- 视神经长节段病变（>½视神经长度或累及视交叉）- T2高信号，增强扫描可见强化- 双侧同时或序贯受累### 脊髓MRI| 特征 | 描述 | 病变长度 | ≥3个椎体节段（"长节段横贯性脊髓炎"）| 位置偏好 | 颈髓、胸髓；中央灰质为主 | 急性期 | 脊髓肿胀、T2高信号、增强强化 | 慢性期 | 脊髓萎缩、空洞形成。### 脑部MRI（支持性表现）- 最后区（area postrema）病变- 第四脑室周围、下丘脑、丘脑、大脑脚病变- **注意**：不符合MS的Dawson指征或近皮层juxtacortical病变## 四、诊断分类。| 类型 | 标准 || **AQP4-IgG阳性NMOSD** | 1项核心临床特征 + AQP4-IgG阳性 || **AQP4-IgG阴性NMOSD** | 需满足：①视神经炎+急性脊髓炎；②附加MRI要求（视神经≥½长度或累及视交叉，脊髓≥3个节段）；③排除其他诊断 |五、关键鉴别诊断| 疾病 | 鉴别要点 || **多发性硬化（MS）** | 短节段脊髓病变、寡克隆区带阳性、Dawson指征、AQP4-IgG阴性 || **MOGAD** | MOG-IgG阳性、视神经鞘周围强化、脊髓圆锥受累常见、单相病程多见 || **急性播散性脑脊髓炎（ADEM）** | 儿童多见、感染/疫苗接种前驱史、弥漫性脑病症状 || **系统性红斑狼疮/干燥综合征** | 血清自身抗体、多系统受累 |## 六、临床建议1. **首次发作即检测AQP4-IgG**，避免延误诊断2. **CBA法阴性但临床高度怀疑** → 重复检测或送检更高滴度检测3. **AQP4-IgG与MOG-IgG双阴性** → 需严格满足MRI附加标准，并长期随访4. **眼科会诊**：视野检查、OCT（视网膜神经纤维层变薄）、VEP*医师提示**：NMOSD复发率高、致残严重，早期确诊并启动免疫治疗（如利妥昔单抗、萨特利珠单抗等）对改善预后至关重要。若您或家属存在急性视力下降伴脊髓症状，建议尽快至**神经内科/神经免疫专科**完善上述检查。如需了解具体治疗方案或预后评估，可进一步咨询。

**2、NMOSD与多发性硬化有什么区别？**

作为神经内科/眼科医师，我来为您详细解析**视神经脊髓炎谱系疾病（NMOSD）**与**多发性硬化（MS）**的核心区别。这两种疾病均可累及视神经，但临床处理截然不同。## 一、核心概念对比| 特征 | **NMOSD** | **多发性硬化（MS）** || **定义** | 自身免疫性星形胶质细胞病，以AQP4-IgG或MOG-IgG为标志 | 中枢神经系统的自身免疫性脱髓鞘疾病 || **流行病学** | 亚洲、非洲裔多见；女性显著高发（♀:♂ ≈ 9:1） | 高加索人群多见；女性较多（♀:♂ ≈ 3:1） || **发病年龄** | 中位发病年龄约40岁 | 中位发病年龄约30岁 || **遗传背景** | 与HLA-DRB1*16关联弱 | 与HLA-DRB1*15:01强相关 |## 二、视神经受累的关键差异（眼科医师重点关注）。NMOSD视神经炎特点| 特征 | 具体表现 || **严重程度** | **重度**（视力常降至光感/无光感） || **双侧性** | **同时或序贯双侧受累**（>50%病例） || **视盘表现** | 视盘水肿常见（前部视神经炎） || **恢复预后** | **差**，常遗留严重视力障碍 || **MRI特征** | 视神经病灶**长节段**（>1/2视神经长度或>17mm），可累及视交叉 || **复发倾向** | 高，若不预防治疗复发率>90% |MS视神经炎特点| 特征 | 具体表现 || **严重程度** | 轻-中度（视力多>20/200） || **双侧性** | 通常为**单侧** || **视盘表现** | 球后视神经炎更常见（早期视盘正常） || **恢复预后** | **较好**，多数恢复至20/40以上 || **MRI特征** | 视神经病灶**短节段**、偏心性，位于眶内段 || **复发模式** | 与MS整体复发模式一致 |## 三、脊髓与脑部病变差异NMOSD典型病灶分布：├── 脊髓：长节段横贯性脊髓炎（LETM，≥3个椎体节段）│	└── 中央灰质为主，可致严重瘫痪/尿便障碍├── 脑部：非特异性，但特征性位于│	├── 第四脑室周围（area postrema → 顽固性恶心呕吐）│	├── 丘脑、下丘脑│	└── 无" Dawson指征 "等MS典型表现│MS典型病灶分布：├── 脊髓：短节段、偏心性病灶├── 脑部：│	├── 脑室周围卵圆形病灶（Dawson指征）│	├── 皮质下白质、胼胝体│	├── 幕下（脑干、小脑）│	└── 符合DIS（空间多发）和DIT（时间多发）## 四、血清学标志物（诊断关键）| 抗体 | NMOSD | MS || **AQP4-IgG** | **阳性率~70-80%**（确诊标志） | 阴性 || **MOG-IgG** | 部分血清阴性NMOSD患者阳性 | 阴性 || **寡克隆区带（OCB）** | 阳性率<20% | **阳性率>90%**（脑脊液） |> **重要提示**：AQP4-IgG检测需采用**细胞免疫荧光法（CBA）**，ELISA法假阴性率高。## 五、急性期与长期治疗策略### 急性期治疗| 方案 | NMOSD | MS || **首选** | 大剂量甲泼尼龙冲击（1g/d × 3-5天） | 同左 || **激素抵抗** | **早期血浆置换/免疫吸附**（疗效确切） | 血浆置换证据较弱 || **IVIG** | 可作为替代 | 证据有限 |### 长期预防治疗| 药物类别 | NMOSD | MS || **一线选择** | **抗CD19单抗**（rituximab, inebilizumab）**抗IL-6R**（satralizumab, tocilizumab）**补体抑制剂**（eculizumab, ravulizumab） | 干扰素β、醋酸格拉替雷<br>芬戈莫德、特立氟胺等 || **禁忌/慎用** | **干扰素β、芬戈莫德可能加重病情** | — || **治疗目标** | 预防复发，保护神经功能 | 控制疾病活动，延缓残疾进展 |## 六、临床鉴别流程图` TDA[视神经炎患者] --> B{临床特征评估}B -->|重度/双侧/长节段病灶| C[NMSOD高度怀疑]B -->|轻中度/单侧/短节段| D[MS或其他] C --> E[血清AQP4-IgG检测]E -->|阳性| F[确诊NMOSD]E -->|阴性| G{MOG-IgG检测}G -->|阳性| H[MOGAD诊断]G -->|阴性| I[符合NMOSD诊断标准?]I -->|是| J[血清阴性NMOSD]I -->|否| K[进一步鉴别诊断]D --> L[脑脊液OCB+MRI DIS/DIT]L -->|符合| M[确诊MS] L -->|不符合| N[其他病因排查]## 七、对患者的核心建议1. **确诊前勿盲目治疗**：两种疾病治疗方案截然不同，误诊可能导致NMOSD患者接受有害治疗（如干扰素）2. **抗体检测至关重要**：AQP4-IgG和MOG-IgG是分型关键，建议至有资质的神经免疫中心检测3. **长期随访必要性**：NMOSD复发致残率高，需终身免疫抑制治疗；MS治疗窗口同样关键4. **眼科随访**：即使视力恢复，需定期评估视神经纤维层厚度（OCT）和视野。如需进一步了解特定方面（如妊娠期管理、儿童发病特点或最新靶向药物），请告知，我可以深入阐述。

**3、AQP4抗体阴性是否能诊断为NMOSD？**

作为神经眼科/神经免疫专科医师，我来为您详细解答AQP4抗体阴性与NMOSD诊断的关系。## 核心结论**AQP4抗体阴性仍可能诊断为NMOSD**，但需满足更严格的诊断标准。2015年国际NMO诊断小组（IPND）的修订标准已将疾病重新定义为**视神经脊髓炎谱系疾病（NMOSD）**，并纳入了AQP4抗体阴性的情况。## 2015年NMOSD诊断标准要点### 1. AQP4抗体阳性 NMOSD| 核心临床特征 | 诊断要求 || 视神经炎 | **1项核心临床特征** + AQP4抗体阳性 || 急性脊髓炎 | 排除其他诊断 || 最后区综合征（顽固性呃逆/呕吐） | || 急性脑干综合征 | || 急性间脑综合征 | || 大脑综合征伴NMOSD特征性MRI表现 | |### 2. **AQP4抗体阴性 NMOSD**（需满足更严格条件）必须满足**全部以下要求**:\{诊断} = \t{① 至少2个核心临床特征（且至少1个为视神经炎或急性脊髓炎）} \\\{② 空间播散性（≥2个不同部位）} \\\{③ 满足相应MRI附加条件} \\\{④ 采用最灵敏方法检测AQP4抗体仍为阴性} \\\ {⑤ 排除其他诊断}## AQP4抗体阴性NMOSD的关键MRI要求| 临床特征 | MRI附加条件 || **急性视神经炎** | 视神经MRI：T2高信号或T1增强病灶 **≥1/2视神经长度**，或累及视交叉 || **急性脊髓炎** | 脊髓MRI：病灶 **≥3个连续椎体节段**（LETM，长节段横贯性脊髓炎） || 最后区综合征 | 延髓背侧/最后区病灶 || 急性脑干综合征 | 脑干室管膜周围病灶 |## 重要鉴别：MOG抗体相关疾病。AQP4抗体阴性时需重点检测**MOG抗体**：| 特征 | AQP4抗体阳性NMOSD | MOG抗体相关疾病 || 性别倾向 | 女性为主（♀:♂ ≈ 9:1） | 男女比例相近或男性略多 || 视神经炎特点 | 常复发，视交叉受累，视力损害重 | 常双侧，视盘水肿明显，恢复较好 || 脊髓病灶 | 长节段、中央灰质为主 | 长或短节段，圆锥受累常见 || 脑脊液 | 寡克隆区带少见 | 可有 || 治疗反应 | 需长期免疫抑制 | 部分可单用激素，复发率较低 |> **临床提示**：MOG抗体相关疾病现已从NMOSD中独立出来，成为新的疾病实体。## 诊断流程建议（临床实践中）A[临床疑诊NMOSD视神经炎/脊髓炎/最后区综合征] --> B[血清AQP4抗体检测CBA法+组织法]B --> C{AQP4抗体结果}C -->|阳性| D[诊断: AQP4抗体阳性NMOSD] C -->|阴性| E[血清MOG抗体检测] E --> F{MOG抗体结果}F -->|阳性| G[诊断: MOG抗体相关疾病] F -->|阴性| H[评估是否符合AQP4抗体阴性NMOSD标准] H --> I{满足2015标准?} I -->|是| J[诊断: AQP4抗体阴性NMOSD]I -->|否| K[诊断待定: 单相/复发性ON或TM或其他诊断]## 临床关键要点1. **检测方法敏感性**：必须采用**细胞法（CBA）**检测，ELISA法敏感性不足，假阴性率高2. **抗体状态可能转化**：少数患者病程中抗体由阴转阳，建议复发时复查3. **治疗策略差异**：AQP4抗体阴性NMOSD的治疗反应和预后可能与阳性患者不同，需个体化4. **新型生物标志物**：部分"双阴性"（AQP4-/MOG-）患者可能存在**GFAP抗体**或其他靶点## 总结| 问题 | 答案 || AQP4抗体阴性能否诊断NMOSD？ | **可以**，但需严格满足2015年IPND标准 || 核心要求 | ≥2个核心临床特征 + 特征性MRI + 排除其他诊断 || 必须排除 | MOG抗体相关疾病、多发性硬化、血管炎等 || 关键检查 | CBA法AQP4抗体、MOG抗体、特征性MRI序列 |**建议**：若您或患者面临此情况，建议在**神经免疫专科中心**完成全面评估，包括高场强MRI（3.0T）、标准化抗体检测及脑脊液分析，以确保诊断准确性并制定最优治疗方案。如需进一步讨论具体病例的影像特征或治疗策略，请提供更多临床细节。

4、**NMOSD急性期的治疗方案有哪些？**

作为神经眼科专科医师，我来为您详细解答**视神经脊髓炎谱系疾病（NMOSD）急性期**的治疗方案。## 一、急性期治疗核心目标> 快速终止免疫攻击、减轻炎症反应、保护视神经及脊髓功能、预防不可逆神经损伤## 二、一线治疗方案### 1. 大剂量糖皮质激素冲击治疗（首选）| 项目 | 具体内容 || **甲泼尼龙** | 500–1000 mg/d 静脉滴注，连续3–5天 || **后续过渡** | 冲击后口服泼尼松 1 mg/kg/d，逐渐减量 || **注意事项** | 需配合胃黏膜保护、补钾、补钙、血糖监测 |> **关键点**：减量需缓慢，避免早期复发；部分患者需小剂量长期维持### 2. 血浆置换（PLEX/PE）**适应症：**- 激素冲击治疗48–72小时反应不佳者- 既往激素治疗无效史- 严重视神经炎（视力<0.1）或脊髓炎（截瘫）| 方案 | 每次置换1–1.5倍血浆容量，隔日1次，共5–7次 || 联合策略 | 可与激素冲击同步或序贯进行 |### 3. 免疫吸附（IA）- 适用于PLEX禁忌或无法实施时（如严重低蛋白血症、循环不稳定）- 选择性清除IgG抗体，保留血浆蛋白## 三、二线/挽救治疗方案。| 方案 | 适用情况 | 具体用药 || **IVIG** | 激素禁忌或PLEX不可行 | 0.4 g/kg/d × 5天 || **环磷酰胺** | 难治性、反复发作 | 750 mg/m² 静脉，每月1次 || **利妥昔单抗** | 早期启动长期预防 | 375 mg/m²/周 × 4周 |## 四、NMOSD急性视神经炎的特殊考量 A[急性视力下降] --> B{AQP4-IgG或MOG-IgG阳性?}B -->|是| C[立即激素冲击] B -->|阴性但临床典型| C --> D{72小时反应?} D -->|差| E[启动PLEX/IA] D -->|良好| F[口服激素序贯减量] E --> G[同步启动长期免疫治疗] F --> G## 五、关键监测指标| 监测内容 | 时间点 || 视力（LogMAR）、VEP、OCT（RNFL厚度） | 每日至每周 || 血清AQP4-IgG/MOG-IgG滴度 | 急性期及随访 || 脊髓MRI（长节段横贯性脊髓炎） | 基线及治疗后 || 感染筛查（乙肝、结核、CMV等） | 免疫抑制前必查 |## 六、重要提醒。1. **禁用干扰素-β、芬戈莫德**（可能加重病情）2. **尽早启动长期预防治疗**（如利妥昔单抗、萨特利珠单抗、伊奈利珠单抗等），急性期治疗后2–4周内过渡3. **复发率高**：未经预防治疗，年复发率>50%。如需了解具体药物的医保政策、妊娠期用药调整，或MOGAD与NMOSD的治疗差异，可进一步咨询。

**5、为什么NMOSD会出现顽固性呃逆、恶心和呕吐？**

作为神经眼科专科医师，我来解释NMOSD（视神经脊髓炎谱系疾病）患者出现顽固性呃逆、恶心呕吐的神经机制。## 核心机制：延髓最后区（Area Postrema）受累### 解剖学基础| 结构特点 | 临床意义 || 位于第四脑室底部，**血脑屏障薄弱** | 血清AQP4-IgG易穿透进入 || 富含**水通道蛋白4（AQP4）** | NMOSD特异性靶抗原高表达 || 含化学感受器触发区（CTZ） | 介导恶心呕吐反射 || 与孤束核、迷走神经背核相连 | 调控呃逆反射弧 |### 病理生理过程。AQP4-IgG与最后区星形胶质细胞AQP4结合-补体激活 → 星形胶质细胞损伤 → 血脑屏障破坏-炎症级联反应 → 神经元功能障碍├─ 化学感受器触发区激活 → 顽固性恶心呕吐└─ 呃逆反射弧（迷走神经/膈神经）失调 → 顽固性呃逆## 临床特征要点### 最后区综合征的典型表现| 症状 | 发生频率 | 特点 || **顽固性呃逆** | 30-40% | 持续>48小时，常规治疗无效 || **顽固性恶心呕吐** | 40-60% | 可先于其他症状出现 || 发作性咳嗽/打喷嚏 | 较少见 | 提示延髓广泛受累 |### 关键临床提示> **"最后区综合征"可作为NMOSD的首发或唯一表现**，极易误诊为消化系统疾病- 呃逆/呕吐与进食**无明确关联**- 胃镜检查**无结构性异常**- 止吐药（如5-HT₃拮抗剂）**效果不佳**## 诊断与鉴别### 影像学特征**MRI矢状位T2/FLAIR**：- 第四脑室底、延髓背侧**片状高信号**- 呈"**倒V字征**"或"**Y字征**"（特征性但非必需）### 血清学检测| 检测项目 | 阳性率 | 注释 || AQP4-IgG | 60-80% | 经典型NMOSD || MOG-IgG | 10-20% | 需鉴别MOGAD || 两者均阴性 | 10-20% | 考虑血清阴性NMOSD |## 治疗原则### 急性期处理| 目标 | 方案 || 终止免疫攻击 | 大剂量甲泼尼龙冲击（500-1000mg/d × 3-5d） || 难治病例 | 血浆置换或IVIG || 症状控制 | 加巴喷丁/巴氯芬（针对呃逆）；非典型抗精神病药 |### 预防复发- **长期免疫抑制**：利妥昔单抗、萨特利珠单抗、伊奈利珠单抗等- 最后区受累者复发风险高，需积极预防## 总结。NMOSD的顽固性呃逆、恶心呕吐并非"胃肠道问题"，而是**自身免疫攻击AQP4富集的延髓最后区**所致。神经科/眼科医师需保持高度警觉，对不明原因的顽固性呃逆呕吐患者及时行头颅MRI（含延髓薄层扫描）及AQP4-IgG检测，避免延误诊断导致不可逆神经损伤。*如需进一步了解NMOSD与视神经炎的关联，或具体免疫治疗方案的选择，可继续咨询。*

**6、如何有效预防NMOSD复发？**

作为神经内科/眼科专科医师，我将为您详细解答视神经脊髓炎谱系疾病（NMOSD）的复发预防策略。## 一、NMOSD复发预防的核心原则。NMOSD是一种**高复发性**的中枢神经系统自身免疫性疾病，视神经炎是其核心临床表现之一。预防复发的关键在于**长期免疫抑制治疗**和**综合管理**。## 二、一线预防性免疫治疗（证据等级A）### 1. 抗CD20单克隆抗体（首选方案）| 药物 | 作用机制 | 给药方案 | 注意事项 || **利妥昔单抗（Rituximab）** | 耗竭B细胞 | 375mg/m²/周×4周或1g×2次，每6个月监测CD19⁺B细胞 | 输注反应、感染风险 || **伊奈利珠单抗（Inebilizumab）** | 耗竭CD19⁺B细胞 | 300mg iv，第0、2周，后每6个月 | 需筛查HBV、结核 || **奥法妥木单抗（Ofatumumab）** | 皮下注射，耗竭B细胞 | 20mg sc，第0、1、2周，后每月 | 可居家注射 |### 2. 补体C5抑制剂- **依库珠单抗（Eculizumab）**：900mg iv qw×4周→1200mg iv 第5周→1200mg iv q2w- **雷夫利珠单抗（Ravulizumab）**：负荷剂量后每8周维持- **适用人群**：AQP4-IgG阳性、复发高风险患者- **关键要求**：必须接种**脑膜炎球菌疫苗**（至少2周前）### 3. IL-6受体拮抗剂- **萨特利珠单抗（Satralizumab）**：120mg sc，第0、2、4周，后每4周- **托珠单抗（Tocilizumab）**：8mg/kg iv 每月（超说明书用药）## 三、传统免疫抑制剂（替代/联合方案）│ 吗替麦考酚酯（MMF） 2-3g/日 分两次	││ 硫唑嘌呤（AZA）	2-3mg/kg/日	││ 需联合泼尼松 10-20mg/日（前6个月）	││ 定期监测：CBC、肝肾功能、TPMT活性	│*重要提示**：单用糖皮质激素**不能预防复发**，仅用于急性期治疗。## 四、AQP4-IgG与MOG-IgG阳性患者的差异管理| 特征 | AQP4-IgG阳性NMOSD | MOG-IgG相关疾病 || 复发倾向 | 极高（未经治疗>90%复发） | 较低，部分单相病程 || 首选药物 | 抗CD20、补体抑制剂 | 可考虑MMF、AZA或观察 || 利妥昔单抗反应 | 优异 | 可能需更短间隔给药 || 预后 | 视神经萎缩风险高 | 视力恢复相对较好 |## 五、非药物预防措施### 1. 避免已知的复发诱因| 类别 | 具体措施 || **感染预防** | 流感/肺炎疫苗接种、及时治疗呼吸道/泌尿道感染 || **妊娠管理** | 孕前咨询，避免产后3-6个月内停药 || **药物规避** | **禁用**：干扰素-β、芬戈莫德、那他珠单抗（可能加重NMOSD） || **热应激** | 避免桑拿、热水浴、剧烈运动后过热 |### 2. 定期监测方案{随访频率} = {治疗初期：每1-3个月} \\{稳定期：每3-6个月} \\{终身随访}**监测内容**：- 血清AQP4-IgG/MOG-IgG滴度（复发前常升高）- 视功能：视力、视野、OCT（RNFL厚度）- MRI：视神经、脊髓、脑部（每年或临床复发时）- 药物安全性：B细胞计数、IgG水平、感染筛查## 六、急性复发的识别与处理### 预警症状（需48小时内就诊）视神经炎复发警示：单眼或双眼视力急剧下降、眼球转动痛（90%特异性）、色觉减退（红绿色为主）、视野缺损扩大。脊髓炎复发警示：肢体无力/麻木平面上升、尿潴留或失禁、顽固性呃逆/呕吐（极后区综合征）### 急性期标准治疗| 方案 | 剂量 | 疗程 || 大剂量甲泼尼龙 | 1g iv qd | 3-5天 || 无效时血浆置换（PLEX） | 1-1.5倍血浆量 | 5-7次，隔日1次 || 或免疫吸附（IA） | 替代PLEX | 同样疗程 |## 七、特殊人群管理### 妊娠期- **计划妊娠前**：切换至妊娠期安全药物（如AZA、环孢素）- **利妥昔单抗**：可在妊娠早期后使用（胎盘转运低）- **补体抑制剂、IL-6拮抗剂**：妊娠期数据有限，通常建议停用### 儿童患者- 优先考虑利妥昔单抗- 注意疫苗接种计划调整（活疫苗需在B细胞耗竭前完成）## 八、患者教育要点> 1. **终身治疗认知**：NMOSD目前不可治愈，停药后复发风险极高> 2. **依从性关键**：即使无症状也需规律用药和随访> 3. **感染警觉**：免疫抑制期间发热需及时就医> 4. **视力保护**：复发后72小时内治疗是视力预后的决定因素## 总结：复发预防的"ABCDE"策略| 字母 | 含义 || **A** | **A**nti-CD20（抗CD20单抗为核心） || **B** | **B**细胞监测（指导利妥昔单抗再治疗时机） || **C** | **C**omplement inhibition（高危患者加用补体抑制剂） || **D** | **D**isease activity monitoring（疾病活动度监测） || **E** | **E**ducation & Avoidance（患者教育与诱因规避） |**建议**：请至有NMOSD诊治经验的神经免疫专科或眼科神经眼科亚专科建立长期随访档案，制定个体化预防方案。如有具体用药问题或近期复发迹象，请尽快联系您的主治医师。*本回答基于2023年国际NMOSD诊断小组（IPND）建议、中国NMOSD诊治指南及AQUAPORT、N-MOmentum等关键临床试验证据。*

**7、目前NMOSD缓解期的一线预防药物有哪些？**

作为神经内科/眼科专业医师，我来为您详细解答NMOSD（视神经脊髓炎谱系疾病）缓解期的一线预防治疗药物。## NMOSD缓解期一线预防药物### 一、国际指南推荐的一线药物（基于发病机制靶向）| 药物类别 | 代表药物 | 作用靶点 | 关键临床试验 | **抗CD19单抗** | 伊奈利珠单抗（Inebilizumab） | CD19+ B细胞 | N-MOmentum研究 || **抗CD20单抗** | 利妥昔单抗（Rituximab） | CD20+ B细胞 | 多项RCT及真实世界研究 || **补体C5抑制剂** | 依库珠单抗（Eculizumab） | 补体C5 | PREVENT研究 || **IL-6受体拮抗剂** | 萨特利珠单抗（Satralizumab） | IL-6R | SAkuraSky/Star研究 || | 托珠单抗（Tocilizumab） | IL-6R | TANGO研究 |### 二、各药物核心特点#### 1. **伊奈利珠单抗（Inebilizumab）**- **优势**：对浆母细胞和浆细胞更有效（CD19广谱），给药方案简化（第0、14天此后每6个月）- **适用**：AQP4-IgG阳性患者为首选之一- **注意**：需筛查乙肝、结核；疫苗接种需提前完成#### 2. **利妥昔单抗（Rituximab）**- **地位**：临床应用最广泛，尤其资源有限地区- **方案**：诱导治疗（375mg/m²每周×4或1000mg×2）+ 维持治疗（根据CD19+细胞计数或固定间隔）- **监测**：需定期检测CD19+ B细胞重建情况#### 3. **依库珠单抗（Eculizumab）**- **优势**：PREVENT研究显示年复发率降低94%- **关键限制**：需终身静脉输注，费用高昂，**脑膜炎球菌感染风险**→必须疫苗接种+预防性抗生素- **适用**：高复发风险、既往严重发作患者#### 4. **萨特利珠单抗（Satralizumab）**- **优势**：皮下注射，患者可居家给药，依从性好- **数据**：单药及联合治疗均显著降低复发风险- **适用**：AQP4-IgG阳性及阴性患者均有效### 三、MOGAD与NMOSD的药物选择差异 A[确诊NMOSD/MOGAD] --> B{抗体类型}B -->|AQP4-IgG阳性| C[一线: 伊奈利珠/依库珠/萨特利珠/利妥昔单抗]B -->|MOG-IgG阳性| D[首选: 利妥昔单抗/伊奈利珠单抗]B -->|双阴性| E[个体化选择，参考临床表型]C --> F[高复发风险→优先依库珠单抗]C --> G[依从性优先→萨特利珠单抗]D --> H[MOGAD复发特点不同，部分患者可尝试免疫抑制剂过渡]### 四、中国临床可及性现状| 药物 | 国内获批状态 | 医保情况 | 备注 || 利妥昔单抗 | 已获批（淋巴瘤适应症，超说明书用于NMOSD） | 医保 | 最经济选择 || 萨特利珠单抗 | 2021年获批NMOSD适应症 | 医保谈判中 | 皮下注射便利 || 伊奈利珠单抗 | 2022年获批 | 医保 | 给药间隔长 || 依库珠单抗 | 已获批PNH/aHUS，NMOSD适应症申请中 | 自费/罕见病渠道 | 需感染防控 || 托珠单抗 | 已获批（RA等，超说明书） | 医保 | IL-6通路替代选择 |### 五、治疗决策的关键考量因素。1. **抗体状态**：AQP4-IgG阳性 vs MOG-IgG阳性 vs 双阴性2. **复发严重程度**：既往有无视神经炎致严重视力丧失、长节段横贯性脊髓炎3. **合并疾病**：活动性感染、肿瘤史、疫苗接种状态4. **患者因素**：妊娠计划、依从性、经济承受能力、静脉通路条件5. **地域可及性**：药物供应、输注中心条件### 六、监测要点- **疗效评估**：定期MRI（视神经+脊髓）、视力功能、扩展残疾状态量表（EDSS）- **安全性监测**：- B细胞耗竭剂：感染筛查、IgG水平监测 - 补体抑制剂：脑膜炎球菌疫苗+抗生素预防- IL-6抑制剂：肝酶、血脂、中性粒细胞计数**临床建议**：对于新诊断的AQP4-IgG阳性NMOSD患者，若条件允许，优先选择具有NMOSD适应症的新型生物制剂（萨特利珠单抗、伊奈利珠单抗或依库珠单抗）；资源有限时，利妥昔单抗仍是有效选择。MOG-IgG相关疾病的治疗策略需更个体化，部分轻症患者可能不需要长期维持治疗。如需针对具体患者情况制定方案，建议提供：抗体类型、既往发作次数/严重程度、合并用药及基础疾病等信息。*参考：国际NMOSD诊断小组（IPND）2023更新、中国NMOSD诊治指南（2021）、AAN/SNM实践指南*

**8、新型的单抗类药物与传统免疫抑制剂相比，在治疗NMOSD中各自的优缺点是什么？**

作为神经内科/眼科医师，我来为您详细分析NMOSD（视神经脊髓炎谱系疾病）治疗中单抗类药物与传统免疫抑制剂的比较。## 一、传统免疫抑制剂### 常用药物| 药物类别 | 代表药物 || 嘌呤类似物 | 硫唑嘌呤（AZA）、霉酚酸酯（MMF） || 钙调神经磷酸酶抑制剂 | 他克莫司、环孢素A || 烷化剂 | 环磷酰胺 || 其他 | 甲氨蝶呤（MTX） |### 优点- **经济可及性**：价格低廉，医保覆盖广泛- **长期应用经验**：数十年临床数据积累，疗效确切- **口服给药**：患者依从性相对较好- **多靶点作用**：对T/B细胞均有抑制作用### 缺点| 问题 | 具体表现 || 起效慢 | 需3-6个月才能达到稳定血药浓度 || 感染风险 | 机会性感染（如JC病毒致PML）、带状疱疹 || 骨髓抑制 | 白细胞减少、贫血、血小板减少 || 肝肾功能损害 | 需定期监测肝酶、肌酐 || 致癌风险 | 长期使用淋巴瘤、皮肤癌风险增加 || 生育毒性 | 致畸作用，妊娠期禁用或慎用 || 复发控制不足 | 年复发率仍可达20-40% |## 二、新型单克隆抗体（靶向生物制剂）### 主要药物及靶点A[NMOSD单抗药物] --> B[抗CD19单抗]A --> C[抗CD20单抗]A --> D[抗IL-6R单抗]A --> E[补体C5抑制剂] B --> B1[Inebilizumab伊奈利珠单抗]C --> C1[Rituximab利妥昔单抗]C --> C2[Ofatumumab奥法妥木单抗]D --> D1[Tocilizumab托珠单抗]D --> D2[Satralizumab萨特利珠单抗]E --> E1[Eculizumab依库珠单抗]E --> E2[Ravulizumab雷夫利珠单抗]### 优点| 维度 | 具体优势 || **疗效卓越** | 复发风险降低>90%（PREVENT研究：Inebilizumab年复发率0.04 vs 安慰剂0.28） || **快速起效** | 数周内显著降低B细胞或阻断IL-6信号 || **精准靶向** | 特异性作用于致病环节，减少脱靶毒性 || **安全性改善** | 骨髓抑制、肝肾毒性显著低于传统药物 || **神经保护** | 部分药物（如抗IL-6R）可能直接减少星形胶质细胞损伤 || **妊娠数据** | 部分药物妊娠期相对安全（如Eculizumab） |### 缺点| 问题 | 具体表现 || **经济负担** | 年治疗费用10-40万元，医保覆盖有限 || **感染风险特殊** | B细胞耗竭→乙肝再激活、进行性多灶性白质脑病（PML）；补体抑制→脑膜炎球菌感染风险 || **给药方式** | 静脉/皮下注射，需医疗机构输注 || **长期数据有限** | 上市时间短，>10年安全性数据待积累 || **原发性失败** | 少数患者对特定靶点药物反应不佳 || **疫苗应答** | 接种效果减弱，活疫苗禁忌 |## 三、关键临床决策要点### 选择单抗类的优先考虑- 高复发活动度（≥2次/年或严重发作）- 传统免疫抑制剂治疗失败或不耐受- 合并AQP4-IgG高滴度阳性- 育龄期女性计划妊娠- 存在骨髓/肝肾基础疾病### 保留传统免疫抑制剂的情况- 低复发风险、经济受限- 单抗类药物不可及地区- 作为单抗的过渡/联合治疗（如Rituximab+MMF）## 四、特殊注意事项（眼科视角）> NMOSD视神经炎具有**复发率高、预后差**的特点，单次发作即可导致严重视力损害。- **急性期**：大剂量甲泼尼龙冲击+血浆置换/IVIG- **长期预防**：优先推荐单抗类药物，尤其针对**视神经脊髓炎型**患者- **视力监测**：OCT检查RNFL厚度、视野检查，即使"临床稳定"也可能存在亚临床进展## 五、总结对比表| 特征 | 传统免疫抑制剂 | 新型单抗类 || 疗效强度 | 中等 | 强效 || 起效速度 | 慢（月计） | 快（周计） || 安全性谱 | 骨髓、肝肾、致癌 | 感染（特定类型）、输液反应 || 经济成本 | 低 | 高 || 使用便捷性 | 口服方便 | 注射不便 || 长期经验 | 丰富 | 相对有限 || 精准性 | 非特异性 | 靶向精准 |**建议**：具体方案需结合患者复发频率、抗体类型（AQP4-IgG vs MOG-IgG）、合并症、经济状况及生育计划个体化制定。对于中重度NMOSD，目前国际指南（2023 AAN/2024中国指南）已将单抗类药物作为一线推荐。如需针对具体药物（如Inebilizumab vs Satralizumab的选择）进一步讨论，可提供更多临床信息。

**9、长期使用预防NMOSD复发的药物，主要的副作用有哪些？**

作为眼科医师，我将为您详细解答视神经脊髓炎谱系疾病（NMOSD）长期预防用药的主要副作用。## 一、一线治疗药物：抗CD19单克隆抗体### 1. 利妥昔单抗（Rituximab）| 副作用类别 | 具体表现 | 发生率/特点 ||-----------|---------|-----------|| 输注相关反应 | 发热、寒战、低血压、支气管痉挛 | 首次输注常见（10-25%） || 感染风险 | 呼吸道感染、尿路感染、乙肝再激活 | B细胞耗竭导致 || 低丙种球蛋白血症 | 反复感染 | 长期用药需监测IgG水平 || 进行性多灶性白质脑病（PML） | 罕见但致命 | JC病毒再激活 |### 2. 伊奈利珠单抗（Inebilizumab）- **主要副作用**：尿路感染（20%）、输液反应（12%）、下呼吸道感染- **特殊注意**：与利妥昔单抗类似，需监测B细胞计数和免疫球蛋白---## 二、补体C5抑制剂### 依库珠单抗（Eculizumab）/ 瑞利珠单抗（Ravulizumab）关键风险：脑膜炎球菌感染（风险增加1000-2000倍）| 管理要点 | 具体措施 || 疫苗接种 | 用药前至少2周接种脑膜炎球菌疫苗（MenACWY + MenB） || 预防性抗生素 | 青霉素V或阿奇霉素（如疫苗接种未完成） || 其他感染 | 肺炎链球菌、流感嗜血杆菌风险轻度增加 || 非感染性 | 头痛、上呼吸道感染、恶心、贫血 |## 三、IL-6受体拮抗剂### 萨特利珠单抗（Satralizumab）/ 托珠单抗（Tocilizumab）| 系统 | 副作用 | 监测建议 || 感染 | 上呼吸道感染、鼻咽炎、尿路感染 | 定期血常规、肝功能 || 实验室异常 | 中性粒细胞减少、转氨酶升高、血脂升高 | 每4-8周检测 || 注射部位反应 | 疼痛、红斑 | 通常轻微 || 胃肠道 | 腹泻、腹痛 | 对症处理 |**特殊注意**：活动性感染或结核潜伏感染需筛查和治疗后使用## 四、传统免疫抑制剂（部分患者仍在使用）### 硫唑嘌呤 / 霉酚酸酯 / 环磷酰胺| 药物 | 主要副作用 | 长期风险 || 硫唑嘌呤 | 骨髓抑制、肝毒性、胰腺炎、淋巴瘤风险 | 皮肤癌（尤其鳞癌） || 霉酚酸酯 | 腹泻、白细胞减少、感染、致畸性 | 进行性多灶性白质脑病（罕见） || 环磷酰胺 | 出血性膀胱炎、骨髓抑制、不孕、膀胱癌 | 继发性恶性肿瘤 |## 五、长期用药的系统性监测建议A[NMOSD患者长期随访] --> B[每3-6个月] A --> C[每6-12个月]A --> D[每年]B --> B1[血常规、肝肾功能] B --> B2[免疫球蛋白定量] B --> B3[感染筛查]C --> C1[淋巴细胞亚群] C --> C2[肿瘤标志物]D --> D1[全身肿瘤筛查]D --> D2[骨密度]D --> D3[眼科专科检查]## 六、患者教育要点1. **感染预警**：发热≥38.5°C持续24小时、咳嗽伴呼吸困难、排尿灼痛——**立即就医**2. **疫苗接种**：- 活疫苗：用药前完成（如MMR、水痘、黄热病）- 灭活疫苗：用药期间可接种，但效果可能减弱3. **妊娠计划**：需提前与神经科、产科医师共同制定方案（尤其MMF有致畸性）4. **定期随访**：切勿自行停药，复发可能导致不可逆视力损害**总结**：NMOSD预防用药的副作用管理核心是**感染防控**和**定期监测**。新型生物制剂（抗CD19、补体抑制剂、IL-6拮抗剂）相比传统免疫抑制剂，整体安全性更优，但各有特有风险，需个体化选择。如需了解特定药物的详细用药方案或副作用处理，建议携带具体药名至神经内科或眼科神经专科门诊进一步咨询。

**10、在NMOSD用药期间可以接种疫苗吗？**

作为眼科/神经眼科专业医师，我来为您解答NMOSD（视神经脊髓炎谱系疾病）患者用药期间的疫苗接种问题。## 核心结论**NMOSD患者在免疫抑制治疗期间接种疫苗需谨慎评估，多数情况下建议调整接种时机，而非完全禁止。**## 一、关键考量因素### 1. 当前用药方案（决定性因素）| 药物类型 | 疫苗接种建议 | 备注 || **糖皮质激素**（如甲泼尼龙、泼尼松） | 避免活疫苗；灭活疫苗可接种但效果可能减弱 | 泼尼松≥20mg/日或等效剂量，持续≥2周时免疫功能受抑 || **利妥昔单抗**（Rituximab，抗CD20单抗） | **接种前2-4周完成疫苗**；用药期间避免活疫苗 | B细胞清除导致体液免疫应答严重受损 || **萨特利珠单抗**（Satralizumab，IL-6受体拮抗剂） | 类似利妥昔单抗，建议用药前完成接种 | 2020年国内上市，用于AQP4-IgG阳性患者 || **伊奈利珠单抗**（Inebilizumab） | 同上 | 抗CD19单抗 || **吗替麦考酚酯/硫唑嘌呤** | 避免活疫苗；灭活疫苗可考虑 | 用药期间接种效果不确定 || **托珠单抗**（Tocilizumab） | 避免活疫苗 | IL-6抑制剂 |### 2. 疫苗类型区分：活疫苗（禁忌或慎用）灭活疫苗（相对安全）

├── 麻疹-风疹-腮腺炎(MMR)	├── 流感疫苗（推荐）

├── 水痘-带状疱疹活疫苗	├── 新冠疫苗（mRNA/灭活）

├── 口服脊髓灰质炎疫苗	├── 肺炎球菌疫苗

├── 黄热病疫苗	├── 乙肝疫苗

└── 鼻喷流感疫苗	└── 带状疱疹重组疫苗（Shingrix，非活疫苗，可用）## 二、具体临床建议###推荐接种的情况| 场景 | 具体建议 || **流感疫苗** | 每年接种灭活流感疫苗；用药期间可接种，但需告知患者保护效力可能降低 || **新冠疫苗** | 推荐接种mRNA或灭活疫苗；避免在B细胞清除剂输注后1-3个月内接种 || **肺炎球菌疫苗** | 推荐；PCV13→PPSV23序贯接种，用药前完成最佳 || **带状疱疹重组疫苗(Shingrix)** | **强烈推荐**；NMOSD患者带状疱疹风险增高；该疫苗为非活疫苗，用药期间可接种 |###需避免或推迟的情况- **利妥昔单抗输注后6个月内**：避免接种依赖抗体应答的疫苗- **大剂量激素冲击期间**（甲泼尼龙≥500mg/日）：暂缓非紧急疫苗接种- **疾病急性复发期**：待病情稳定后再评估## 三、特殊注意事项（眼科视角）NMOSD患者常因**视神经炎**就诊，需特别关注：1. **疫苗与复发风险**：目前无证据表明灭活疫苗增加NMOSD复发风险；但**活疫苗理论上存在诱发免疫激活的潜在风险**2. **用药前疫苗筛查**：建议所有确诊NMOSD、尚未开始免疫抑制治疗的患者，尽快完善：- 乙肝表面抗原/抗体- 水痘-带状疱疹病毒IgG- 麻疹、风疹IgG。抗体阴性者，应在免疫抑制治疗前**2-4周**补种相应疫苗3. **新冠疫苗特别说明**：mRNA疫苗（如复必泰）和灭活疫苗均安全；接种后如出现**新发视力下降、眼痛**，需警惕视神经炎复发，及时就诊## 四、实践流程建议。确诊NMOSD → 评估疫苗接种史 → 检测保护性抗体-开始免疫抑制治疗前2-4周-补种所有适龄灭活疫苗 + 必要的活疫苗（如抗体阴性且未用药）-开始免疫抑制治疗-治疗期间：每年流感疫苗 + 新冠加强针 + 肺炎球菌疫苗（如未接种）-活疫苗需停药后≥3-6个月（根据具体药物半衰期）## 五、关键文献依据- 2021 ACR指南：风湿病和自身免疫病患者疫苗接种- 2022 EULAR建议：自身免疫性炎症性风湿病患者疫苗接种- 2023 中国NMOSD诊治指南（免疫抑制治疗章节）## 总结建议> **请携带您的具体用药清单（药物名称、剂量、末次用药时间）至神经内科或风湿免疫科门诊，由主治医生根据当前免疫状态制定个体化接种方案。**如需紧急接种（如暴露后狂犬病疫苗），即使处于免疫抑制期也应接种，并可考虑同时给予**免疫球蛋白**增强保护。您是否方便告知目前使用的具体药物名称和剂量？我可以提供更精准的接种时机建议。

**11、如何缓解NMOSD引起的长期神经性疼痛、肌肉痉挛或顽固性瘙痒？**

作为神经内科/眼科专科医师，我将就视神经脊髓炎谱系疾病（NMOSD）相关症状的缓解策略进行专业解答。## 一、NMOSD神经性疼痛的管理### 1. 一线药物治疗| 药物类别 | 代表药物 | 起始剂量 | 注意事项 || 钙通道α2-δ配体 | **普瑞巴林** | 75mg bid | 水肿、体重增加、嗜睡 | | **加巴喷丁** | 300mg tid | 肾功能调整剂量 |

| 三环类抗抑郁药 | **阿米替林** | 10-25mg qn | 抗胆碱能副作用、心脏毒性 || SNRIs | **度洛西汀** | 30mg qd | 恶心、高血压 |### 2. 二线/难治性疼痛选择- **卡马西平/奥卡西平**：针对闪电样、枪击样疼痛（提示神经病理性疼痛中的阵发性成分）- **拉莫三嗪**：缓慢滴定，注意皮疹风险- **阿片类药物**（如曲马多、羟考酮）：仅作为最后选择，需严格评估成瘾风险### 3. 介入治疗- 脊髓电刺激（SCS）：针对顽固性中枢性疼痛- 经颅磁刺激（rTMS）：辅助缓解神经病理性疼痛## 二、肌肉痉挛的管理### 药物治疗阶梯第一阶梯：口服药物├── 巴氯芬 5mg tid起始，最大80mg/日├── 替扎尼定 2mg qn起始，最大36mg/日（需监测肝功能）├── 丹曲林钠 25mg qd（适用于局灶痉挛，肝毒性限制使用）── 苯二氮䓬类（如地西泮）：短期使用，注意镇静和依赖。第二阶梯：局部治疗├── 肉毒毒素A注射：针对局灶性痉挛，每3-4个月重复└── 鞘内注射：巴氯芬泵植入（ITB）——用于严重全身性痉挛### 非药物干预- **物理治疗**：牵伸训练、水疗、功能性电刺激- **矫形器**：踝足矫形器（AFO）预防挛缩- **运动疗法**：基于Bobath概念的神经肌肉促进技术## 三、顽固性瘙痒（神经病理性瘙痒）的管理> NMOSD相关瘙痒多为**中枢性瘙痒**，源于脊髓后角或延髓病变，常规抗组胺药效果差。### 针对性治疗策略| 机制 | 药物 | 证据等级 || 抑制神经兴奋性 | **加巴喷丁/普瑞巴林** | Ⅱ级证据 || 钠通道阻断 | **卡马西平** | 病例系列 || κ-阿片受体激动 | **纳布啡**（静脉）/ **布托啡诺**（鼻喷） | 病例报告 || 5-HT3拮抗 | **昂丹司琼** 4-8mg tid | 小型研究 |### 局部治疗- **辣椒素贴剂**（8%）：用于局限性瘙痒区域- **局部麻醉药**（利多卡因贴剂）## 四、NMOSD特异性考量### 关键原则1. **病因治疗优先**：确保患者正在接受稳定的免疫抑制治疗（如利妥昔单抗、萨特利珠单抗、伊奈利珠单抗等），控制疾病活动是症状管理的基础2. **识别发作 vs 后遗症**：- 新发疼痛/痉挛可能提示**复发**，需紧急MRI评估- 稳定期的症状多为**后遗症性**，按慢性症状处理3. **药物相互作用**：- 免疫抑制剂（如硫唑嘌呤）与替扎尼定存在代谢相互作用- 利妥昔单抗可能增加感染风险，侵入性操作需谨慎### 多学科协作建议NMOSD症状管理MDT团队├── 神经内科（主导）├── 疼痛科（介入治疗）├── 康复医学科（物理治疗）├── 心理科（慢性疼痛相关焦虑抑郁）└── 眼科（视神经病变随访）## 五、患者教育要点1. **疼痛日记**：记录疼痛性质、诱因、药物反应2. **温度管理**：NMOSD患者常存在温度敏感性，避免过热诱发症状（Uhthoff现象）3. **妊娠计划**：多数抗痉挛药和镇痛药需孕前调整（如丙戊酸禁用、巴氯芬需评估）**建议下一步行动**：如上述症状严重影响生活质量，建议至**NMOSD专病门诊**或**神经免疫中心**进行系统评估，制定个体化方案。对于难治性病例，可考虑参加临床试验或转诊至疼痛专科。是否需要针对某一具体症状展开更详细的讨论？

**12、NMOSD导致的大小便功能障碍怎么办？**

您好，我是您的眼科医师。首先需要向您澄清一个重要问题：**NMOSD（视神经脊髓炎谱系疾病）导致的大小便功能障碍属于神经系统症状，而非眼科疾病本身**。不过，作为经常诊治NMOSD患者的眼科医师，我对此病的多系统表现非常熟悉，可以为您提供专业建议：## 一、NMOSD与大小便功能障碍的关系。NMOSD是一种**中枢神经系统自身免疫性疾病**，主要攻击：- **视神经** → 视力下降、眼痛（眼科主要关注点）- **脊髓** → 长节段横贯性脊髓炎（LETM），常累及胸腰段- **延髓最后区** → 顽固性恶心呕吐**脊髓病变**是导致大小便功能障碍的直接原因，发生率约**60-80%**。## 二、评估与诊断要点| 评估项目 | 具体内容 ||---------|---------|| **脊髓MRI** | 明确病变节段、急性/慢性期、是否强化 || **尿动力学检查** | 区分逼尿肌过度活动 vs. 收缩无力 || **残余尿测定** | 评估排尿功能（>100ml为异常） || **肛门直肠功能评估** | 必要时行肛管直肠测压 |## 三、分层治疗策略### 1. 急性期处理（复发时）- **大剂量糖皮质激素冲击**：甲泼尼龙 500-1000mg/d × 3-5天- **血浆置换（PLEX）**：对激素反应差者，尤其脊髓病变严重者- **IVIG**：替代方案### 2. 症状针对性管理#### 神经源性膀胱| 类型 | 表现 | 处理 || **逼尿肌过度活动** | 尿急、尿频、尿失禁 | 抗胆碱能药（索利那新、托特罗定）、β3受体激动剂（米拉贝隆） || **逼尿肌收缩无力** | 排尿困难、残余尿增多 | 间歇清洁导尿（CIC）、α受体阻滞剂 || **逼尿肌-括约肌协同失调（DSD）** | 排尿困难+上尿路损害风险 | CIC为核心，必要时肉毒毒素注射 |#### 神经源性肠道功能障碍- **便秘为主**：膳食纤维、渗透性泻药（聚乙二醇）、促动力药- **排便失禁**：盆底肌训练、生物反馈、必要时灌肠方案### 3. 长期预防复发（关键！）- **一线免疫抑制剂**：吗替麦考酚酯、硫唑嘌呤、利妥昔单抗- **新型靶向药**：伊奈利珠单抗、萨特利珠单抗、依库珠单抗等## 四、重要提醒1. **上尿路保护**：长期残余尿增多可导致肾积水、肾功能损害，需定期泌尿系超声监测2. **多学科协作（MDT）**：建议联合**神经内科**（疾病修饰治疗）、**泌尿外科/尿控专科**（膀胱管理）、**康复医学科**（盆底康复）3. **与眼科的关联**：NMOSD患者常因**AQP4-IgG**或**MOG-IgG**阳性确诊，眼部复发与脊髓复发可能不同时发生，需全程免疫抑制## 五、建议下一步行动1. 若当前为急性发作 → 尽快神经内科就诊，评估是否需要激素冲击/PLEX2. 若为慢性期症状 → 泌尿外科/尿控门诊完善尿动力学检查3. 无论何种情况 → 确认免疫抑制方案是否充分，预防未来复发。您是否目前正处于急性发作期？或者想了解具体某种治疗方案的更多细节？我可以进一步协助您梳理。

**13、NMOSD病情稳定后，应该如何进行科学、安全的康复训练？**

作为您的眼科医师，我必须首先强调：**NMOSD（视神经脊髓炎谱系疾病）的康复训练必须在神经内科或康复科医生全面评估并指导下进行，且需与神经免疫治疗同步。** 以下为基于当前医学共识的康复指导原则，**不可替代您的主治医生团队制定的个性化方案**。### **核心原则：安全第一，科学渐进，多学科协作**NMOSD是一种中枢神经系统自身免疫性疾病，主要累及视神经和脊髓。康复目标并非“治愈”疾病本身，而是 **最大限度地恢复和保留功能，预防并发症，提高生活质量**。### **康复训练的四大支柱**#### **1. 视力能康复（针对视神经炎后遗症）**- **低视力评估与辅助**：首先由眼科或低视力门诊进行专业评估。根据视力缺损（如中心暗点、视野缩小）情况，使用：- 光学辅助器具：如放大镜、望远镜。 - 电子助视器：如电子屏幕放大器、语音软件。- 环境调整：改善家居照明、使用高对比度物品。- **视觉训练**：在治疗师指导下进行，可能包括：- 扫视与追踪训练：提高眼球运动控制能力。- 视野感知训练：学习利用剩余视野的技巧。 - **注意**：训练强度需极低，避免视疲劳诱发复发。#### **2. 躯体功能康复（针对脊髓炎后遗症）**这是康复的核心，必须在**康复医师和物理治疗师**指导下进行。- **运动功能**：- **肌力训练**：针对无力肌群，以**等长收缩、非负重、低强度**为主。**严禁过度疲劳**，疲劳是NMOSD复发的重要风险因素。- **痉挛管理**：常见后遗症。包括药物（如巴氯芬）、牵伸、体位摆放、物理治疗（如冷热疗）。- **平衡与步态训练**：使用平行杠、助行器，在绝对安全环境下进行，严防跌倒。- **感觉障碍**：- 进行感觉再教育训练，如不同材质物品的辨别。- 加强皮肤护理，因感觉缺失易导致压疮、烫伤。- **膀胱与肠道功能**：- 制定定时排尿计划，学习间歇导尿技术（如必要）。- 饮食调整、药物管理便秘。#### **3. 日常生活能力（ADL）与职业康复**- **作业治疗师**将帮助您：- 改造日常活动（穿衣、洗漱、烹饪）的方法和工具。- 进行手功能精细动作训练。- 评估工作环境，提供重返工作岗位的建议与适应性训练。#### **4. 疲劳管理与心理支持**- **疲劳**：是NMOSD最突出的症状之一，非普通劳累。- **能量节约技术**：学习将活动分段，安排休息优先级。- **有氧运动**：在医生许可下，进行**极低强度**的有氧活动，如温水游泳、卧位脚踏车。**心率与疲劳感是严格监控指标**。- **心理与认知**：- NMOSD常伴焦虑、抑郁及认知功能（如记忆力、注意力）下降。- 寻求心理科或神经心理科支持至关重要，可进行认知康复训练。### **【绝对禁忌与重要警告】**1. **避免过热**：严禁热水浴、桑拿、高温瑜伽等。**Uhthoff现象**（体温升高导致神经症状一过性加重）在NMOSD中极为常见。2. **严禁过度疲劳**：“今天练到位，明天躺三天”的模式极其危险，可能诱发免疫激活。**“无疲劳训练”是铁律**。3. **避免感染**：康复场所需清洁，注意保暖。感染是已知的复发重要诱因。4. **警惕新症状**：康复过程中出现任何**新发或加重的疼痛、麻木、无力、视力下降、排尿困难**，必须立即停止训练并联系您的主治神经内科医生，这可能是病情活动的信号。### **行动路线图**1. **获取全面评估**：联系您的神经内科主治医生，请求转介至**康复医学科**。2. **组建团队**：理想团队包括：神经内科医生、康复科医生、物理治疗师、作业治疗师、眼科/低视力医生、心理医生。3. **制定个性化计划**：与团队共同制定书面康复计划，明确目标、强度、频率和风险预案。4. **保持沟通与记录**：定期向团队反馈，记录训练后的感受和任何异常。**总结**：NMOSD稳定期的康复是一门精细的“平衡艺术”，是在不激发免疫系统的前提下，小心翼翼地挖掘神经系统的修复与代偿潜力。它需要极大的耐心、科学的指导和严格的自我观察。请务必在专业医疗团队的护航下开启您的康复之旅。

**14、NMOSD能治愈吗？**

作为神经眼科专业医师，我来为您详细解答NMOSD的治疗现状。## NMOSD目前**无法完全治愈**，但可实现长期缓解### 疾病本质：视神经脊髓炎谱系疾病（NMOSD）是一种**自身免疫性中枢神经系统疾病**，主要攻击：- 视神经 → 导致视力下降、失明- 脊髓 → 导致截瘫、感觉障碍- 延髓最后区 → 导致顽固性恶心呕吐。核心机制：AQP4-IgG抗体（约80%患者）或MOG-IgG抗体攻击星形胶质细胞。## 治疗目标与现状| 治疗阶段 | 目标 | 实现程度 || **急性期治疗** | 挽救神经功能、缩短发作 |成熟有效 || **预防复发治疗** | 降低年复发率 |显著改善 || **功能修复** | 逆转已形成的神经损伤 |有限 || **根治疾病** | 消除自身免疫异常 |尚未实现 |## 关键治疗进展（改变疾病预后）### 一、急性期治疗- **大剂量甲泼尼龙冲击**（500-1000mg/d × 3-5天）- 血浆置换（重症或激素反应差者）- 静脉免疫球蛋白（IVIG）### 二、预防复发治疗（里程碑式突破）| 药物类别 | 代表药物 | 作用机制 || 抗CD19单抗 | **伊奈利珠单抗**、利妥昔单抗 | 清除B细胞 || 补体C5抑制剂 | **依库珠单抗** | 阻断补体级联 || IL-6受体拮抗剂 | **萨特利珠单抗** | 抑制炎症通路 || 口服小分子 | **伊布替尼**等 | BTK抑制剂 |> **临床数据**：规范使用新型单抗后，年复发率可从**1.5-2次降至<0.1次**## 患者最关心的预后问题###积极方面- **视力保护**：早期规范治疗，>80%患者可保留有用视力- **致残率下降**：预防复发治疗使5年致残率从60%降至<20%- **生存期**：接近正常人群预期寿命###现实局限- 已发生的**轴突损伤不可逆**（如视神经萎缩、脊髓空洞）- 需**终身免疫抑制**（停药后复发风险极高）- 少数难治性病例仍存在治疗挑战## 给患者的核心建议1. **确诊即治疗**：AQP4抗体阳性者，无论单次发作还是复发病程，均需尽早启动预防治疗2. **定期随访**：每3-6个月评估EDSS评分、OCT（视网膜神经纤维层厚度）、MRI3. **感染防控**：免疫抑制状态下需警惕机会性感染（如乙肝再激活、JC病毒致PML）4. **妊娠规划**：需提前6个月调整用药（如换用硫唑嘌呤或他克莫司）## 未来研究方向- **CAR-T细胞治疗**：靶向B细胞的深度清除- **耐受性诱导**：重建免疫耐受而非单纯抑制- **神经修复**：干细胞移植、神经营养因子**总结**：NMOSD虽不能根治，但已从"高复发、高致残"的可怕疾病转变为**可控制的慢性病**。关键在于早期诊断、规范预防和终身管理。建议至有神经眼科或神经免疫专科的中心系统随访。如需了解具体用药选择或生育、疫苗接种等细节问题，可进一步咨询。

**15、NMOSD的长期预后怎么样？**

NMOSD（视神经脊髓炎谱系疾病）是一种罕见的中枢神经系统自身免疫性疾病，主要累及视神经和脊髓。关于其长期预后，我将从专业角度为您详细解答：## 一、总体预后特点**NMOSD是一种复发性疾病**，与多发性硬化（MS）不同，其预后通常较差，残疾累积风险较高。每次发作都可能造成新的、不可逆的神经系统损伤。## 二、关键预后因素### 1. **抗体状态**- **AQP4-IgG阳性**：经典NMOSD，复发率高，预后相对较差- **MOG-IgG阳性**：通常预后较好，单相病程比例较高，但部分患者也会复发- **双重阴性**：预后介于两者之间### 2. **发作严重程度与部位**- 视神经炎严重发作可导致**永久性视力丧失**- 长节段横贯性脊髓炎（LETM）可导致**截瘫、大小便功能障碍**- 延髓最后区综合征（顽固性恶心呕吐）影响生活质量### 3. **治疗时机**- **早期诊断和及时免疫抑制治疗**是改善预后的关键- 延误治疗会导致残疾累积## 三、长期功能预后数据| 系统受累 | 长期预后 || **视力** | 约50-60%患者单眼或双眼永久性视力损害，部分患者法定盲 || **运动功能** | 约30-40%患者最终需要辅助行走工具，15-20% wheelchair依赖 || **膀胱/肠道** | 脊髓受累者常见，严重影响生活质量 || **呼吸** | 高位颈髓病变可导致呼吸衰竭，是主要死因之一 || **认知** | 相对保留，优于MS，但部分患者可有执行功能损害 || **生存** | 10年生存率约70-90%（免疫治疗时代前较低，现代治疗已改善） |## 四、现代免疫治疗时代的预后改善**关键转折点**：2010年后，随着以下药物应用，预后显著改善：- **利妥昔单抗（Rituximab）**：B细胞清除，有效降低复发率- **萨特利珠单抗（Satralizumab）**：IL-6受体拮抗剂- **伊奈利珠单抗（Inebilizumab）**：CD19靶向- **依库珠单抗（Eculizumab）**：C5补体抑制剂，用于AQP4抗体阳性**现代治疗目标**：- 实现**无复发、无新病灶、无残疾进展**- 早期干预可使约70-80%患者达到疾病控制## 五、主要死亡风险1. **呼吸衰竭**：高位颈髓病变、神经源性肺水肿2. **严重感染**：长期免疫抑制治疗的并发症3. **顽固性疼痛**：神经病理性疼痛导致生活质量极差## 六、随访建议作为患者，以下措施对改善长期预后至关重要：1. **终身免疫抑制治疗**：不可擅自停药，复发风险在停药后很高2. **定期随访**：- 每3-6个月神经科随访- 每年视神经和脊髓MRI监测- 眼科定期评估（视神经受累者）3. **共病管理**：骨质疏松预防、感染预防、疫苗接种（避免活疫苗）4. **妊娠规划**：需孕前咨询，部分药物需调整## 七、总结> **NMOSD是一种需要终身管理的严重疾病，但在现代靶向免疫治疗时代，预后已显著改善。早期诊断、规范治疗、终身随访是改善长期预后的三大支柱。未经治疗的NMOSD预后较差，而规范治疗可使大多数患者维持功能独立和生活质量。****免责声明**：以上信息仅供医学教育参考，不能替代您的主治医师的个体化诊疗建议。NMOSD的预后存在显著的个体差异，请与您的神经免疫专科医生详细讨论您的具体情况。

**16、NMOSD患者可以怀孕生子吗？**

作为神经内科/眼科联合诊疗的专业医师，我来为您详细解答视神经脊髓炎谱系疾病（NMOSD）患者的妊娠相关问题。## 核心结论**NMOSD患者可以怀孕生子，但属于高危妊娠，需要严格的孕前规划、孕期监测和产后管理。** 近年来随着治疗进展，成功妊娠的案例已显著增加。## 一、疾病与妊娠的相互影响### 1. 妊娠对NMOSD的影响| 时期 | 复发风险特点 || **妊娠期** | 复发风险相对降低（尤其中后期，雌激素/孕激素的免疫调节作用） || **产后期** | **复发风险显著升高**（产后3-6个月为高危期，激素撤退+免疫重建） || **哺乳期** | 需权衡哺乳与免疫治疗的关系 |### 2. NMOSD对妊娠的影响- **视神经炎急性发作**：妊娠期视力急剧下降可能影响日常生活安全- **延髓/脊髓受累**：严重发作可导致呼吸、排尿功能障碍，增加妊娠风险- **自身抗体（AQP4-IgG）**：理论上可透过胎盘，但新生儿NMOSD极罕见## 二、关键孕前准备（至关重要）### 1. 疾病控制标准- **建议疾病稳定 ≥ 6-12个月** 后再计划妊娠- 近1年内无严重复发（如长节段横贯性脊髓炎、视神经炎）### 2. 抗体状态评估- 确认 **AQP4-IgG** 或 **MOG-IgG** 阳性状态- MOG-IgG相关疾病通常预后较好，复发模式不同### 3. 药物调整（关键决策点）| 药物类别 | 妊娠期建议 | 备注 || **硫唑嘌呤 (AZA)** |相对安全，可继续使用 | 首选维持药物 || **吗替麦考酚酯 (MMF)** |致畸，孕前需停用 ≥ 3个月 | 需提前换药 || **利妥昔单抗 (RTX)** |个案评估，孕早期后避免 | 计划性妊娠可在孕早期前使用 || **萨特利珠单抗、伊奈利珠单抗** | 数据有限，建议孕前停用 | 新型生物制剂 || **糖皮质激素** |必要时可用（泼尼松<20mg/d安全） | 控制急性复发 || **血浆置换/IVIG** |妊娠期可用 | 急性复发抢救治疗 |## 三、孕期管理要点### 多学科团队（MDT）协作- **神经内科**（主诊）+ **产科**（高危妊娠门诊）+ **眼科** + **麻醉科**### 监测方案：孕早期：每4-6周神经科随访；孕中晚期：每4-8周联合门诊：出现以下症状立即就诊：- 视力突然下降、眼痛- 肢体无力/麻木、排尿障碍 - 顽固性恶心呕吐（需鉴别延髓受累）### 分娩方式- **无绝对剖宫产指征**：疾病本身不强制要求剖宫产- **个体化决策**：若存在严重脊髓病变（如T6以上）、呼吸功能受累，建议剖宫产## 四、产后关键管理### 复发预防（重中之重）- **产后早期强化免疫治疗**：多数中心建议在分娩后2-4周内恢复/加强免疫抑制- 常用方案：IVIG预防性输注、或恢复孕前免疫抑制剂### 哺乳与用药| 药物 | 哺乳建议 || 泼尼松 | 可用（哺乳后服药，间隔4小时） || 硫唑嘌呤 | 可用（婴儿监测血常规） || 利妥昔单抗 | 建议暂停哺乳（B细胞清除） |## 五、给患者的具体建议1. **切勿自行停药**：尤其是MMF等致畸药物需有计划替换，而非突然停用2. **孕前专科门诊**：建议至有NMOSD妊娠经验的神经免疫中心评估3. **遗传咨询**：NMOSD非经典遗传病，但一级亲属患病风险轻度增高4. **心理支持**：疾病与妊娠的双重压力，建议加入患者互助组织## 参考文献与指南- 2023年《中国视神经脊髓炎谱系疾病诊断与治疗指南》- 2021年国际NMO诊断小组（IPND）妊娠管理建议- 欧洲神经病学学会（EAN）2022年指南**建议下一步行动**：预约神经免疫专科门诊，携带近期MRI、抗体检测报告及用药记录，制定个体化妊娠计划。您是否希望了解特定药物（如目前正在使用的免疫抑制剂）的具体调整方案？

**17、孕期和产后如何管理疾病，以降低NMOSD复发风险？**

作为神经内科/眼科专科医师，我将为您详细解答NMOSD（视神经脊髓炎谱系疾病）在孕期及产后的管理策略。## 一、NMOSD与妊娠的基本认知### 疾病特点- **高发人群**：育龄期女性（中位发病年龄约39岁，亚洲人群更早）- **核心机制**：AQP4-IgG或MOG-IgG介导的星形胶质细胞损伤- **妊娠影响**：雌激素/孕激素波动可能调节免疫状态，但产后复发风险显著升高### 关键数据| 时期 | 复发风险 || 妊娠期 | 相对较低（尤其孕中晚期） || **产后3-6个月** | **复发风险增加2-3倍** || 未治疗者产后年复发率 | 可达50-70% |## 二、孕前规划（Preconception Planning）### 1. 疾病控制目标- **建议**：病情稳定≥6-12个月后再计划妊娠- 复发次数：孕前2年内≤1次- EDSS评分稳定### 2. 药物调整策略| 药物类别 | 具体药物 | 孕前建议 || **一线维持治疗** | 利妥昔单抗（Rituximab） | 相对安全，建议孕前停用，孕早期必要时可用 || | 萨特利珠单抗（Satralizumab） | 数据有限，建议孕前停用 || | 伊奈利珠单抗（Inebilizumab） | 孕前停用 || **传统免疫抑制剂** | 硫唑嘌呤 | 相对安全，可继续使用 || | 霉酚酸酯 | **致畸风险高，孕前至少停用3-6个月** || | 环磷酰胺 | 孕前至少停用3个月 || **急性期治疗** | 甲泼尼龙冲击 | 妊娠期可用 || | 血浆置换 | 妊娠期可用 || **禁用/慎用** | 托珠单抗 | 数据不足 |### 3. 补充检查- AQP4-IgG/MOG-IgG滴度监测- 视神经MRI（评估既往病灶）- 眼科基线评估：视力、视野、OCT（RNFL厚度）## 三、妊娠期管理### 1. 复发监测。关键症状警示：├── 视神经炎：突发视力下降、眼痛（尤其眼球转动痛）├── 脊髓炎：肢体无力、感觉异常、尿便障碍├── 极后区综合征：顽固性恶心呕吐、呃逆└── 脑干/间脑症状：嗜睡、低钠血症### 2. 急性复发处理| 情况 | 处理方案 || 轻度复发 | 口服泼尼松 1mg/kg/d，逐渐减量 || 中重度复发 | **甲泼尼龙 500-1000mg/d IV×3-5天** || 激素反应差 | 血浆置换（PLEX）——妊娠期安全 || 难治性复发 | 丙种球蛋白（IVIG）0.4g/kg/d×5天 |### 3. 维持治疗决策**妊娠期可用方案：**- **硫唑嘌呤**：最常用，维持剂量1-2mg/kg/d- **利妥昔单抗**：如孕前已用且B细胞耗竭，孕早期单次输注可维持- **IVIG**：每月0.4g/kg作为替代维持治疗**停药后高风险患者管理：**- 建议孕晚期重启预防性治疗- 或产后立即强化治疗### 4. 产科协作要点- 神经科-产科联合门诊随访- 胎儿超声监测（尤其使用免疫抑制剂者）- 分娩方式：NMSD本身非剖宫产指征，视神经损伤严重者避免Valsalva动作## 四、产后管理——**降低复发风险的核心阶段**### 1. 关键时间窗。产后复发高峰：分娩后1-3个月。关键干预：产后立即（24-48小时内）重启/强化维持治疗### 2. 分层管理策略#### 方案A：孕前已停用生物制剂| 时间点 | 措施 || 产后24-72小时 | 启动利妥昔单抗 1g（或375mg/m²×4周） || 或 | 萨特利珠单抗/伊奈利珠单抗重启 || 替代方案 | 每月IVIG 0.4g/kg |#### 方案B：妊娠期使用硫唑嘌呤- 继续原方案- 评估后升级至生物制剂#### 方案C：母乳喂养考量| 药物 | 乳汁分泌 | 建议 || 泼尼松（<20mg/d） | 少量 | 可哺乳，服药后4小时哺乳 || 甲泼尼龙冲击 | 中等 | 暂停哺乳24-48小时 || 硫唑嘌呤 | 少量活性代谢物 | 通常认为安全 || 利妥昔单抗 | 极少 | 可哺乳（大分子蛋白不进入乳汁） || 萨特利珠单抗 | 未知 | 建议暂停哺乳 |### 3. 产后监测强化- **神经科随访**：产后1、3、6个月- **眼科检查**：每月视力、视野、OCT- **血清学**：AQP4-IgG滴度（预测价值有限，但可辅助）## 五、特殊注意事项### 1. MOG-IgG阳性患者- 复发模式不同：单相或复发性视神经炎多见- 产后管理原则类似，但部分患者病程更良性### 2. 既往严重视神经炎- 产后复发可致**不可逆视力丧失**- 建议更积极的预防性治疗### 3. 新生儿注意事项- 母体AQP4-IgG可经胎盘转移- 新生儿需监测：一过性肌张力低下、喂养困难（罕见）- 通常6个月内自行清除## 六、总结：降低产后复发风险的核心措施

│ 1. 孕前规划：病情稳定6-12个月后再妊娠	│

│ 2. 妊娠期：硫唑嘌呤维持，急性复发积极处理 │

│ 3. 关键窗口：产后24-48小时内重启生物制剂 │

│ 4. 首选药物：利妥昔单抗（证据最充分）	│

│ 5. 替代方案：IVIG每月维持	│

│ 6. 多学科协作：神经科-产科-眼科联合管理 │

**建议下一步行动：**1. 如您正在计划妊娠，请携带近期AQP4-IgG/MOG-IgG报告、头颅脊髓MRI、用药记录，预约神经免疫专科门诊2. 如已妊娠，建议每4-6周神经科随访，制定个体化维持方案3. 产后务必提前与主治医生确认重启治疗的具体时间。如需具体药物剂量调整或针对您个人病史的详细方案，请提供更多临床信息。

**18、NMOSD会遗传给下一代吗？**

作为神经内科/眼科医师，我来专业解答NMOSD的遗传问题：## NMOSD的遗传风险### 核心结论**NMOSD不属于经典遗传病，但存在遗传易感性。**### 遗传学特征| 特征 | 说明 || **遗传模式** | 非孟德尔单基因遗传 || **家族聚集性** | 约2-3%患者有家族史（一级亲属患病） || **遗传度** | 中等（估计约30-40%） |### 关键机制**1. 多基因遗传背景**- HLA-DRB1*16:02（亚洲人群）- HLA-DPB1*05:01- 多个免疫相关基因位点（如IL-17、IL-6通路基因）**2. 表观遗传与环境因素**- 病毒感染（EB病毒等）- 维生素D缺乏- 肠道菌群失调- 这些环境因素在遗传易感个体中触发自身免疫### 子代风险评估。普通人群患病率：约1-5/100,000。NMOSD患者一级亲属：相对风险 ↑ 10-20倍。绝对风险仍较低：约0.1-0.5%（1/1000至1/200）### 与经典遗传病对比| 疾病类型 | 遗传模式 | 子代再发风险 || **NMOSD** | 多基因+环境 | **低（<1%）** || 常染色体显性遗传病 | 单基因 | 50% || 常染色体隐性遗传病 | 单基因 | 25% |### 临床建议**1. 遗传咨询要点**- 无需常规产前诊断- 不建议因NMOSD终止妊娠- 可建议子代避免已知环境诱因**2. 子代监测**- 关注视神经炎、长节段横贯性脊髓炎症状- 出现相关症状时检测AQP4-IgG/MOG-IgG抗体**3. 备孕/孕期管理**- 疾病稳定期（≥6个月）再计划妊娠- 避免使用环磷酰胺、甲氨蝶呤- 羟氯喹、硫唑嘌呤相对安全（需个体化评估）### 总结> NMOSD的遗传模式复杂，**绝大多数患者子女不会发病**。与单基因遗传病不同，其发病需要遗传易感背景+环境触发因素共同作用。建议有生育需求的患者进行**神经内科遗传咨询**，而非过度担忧遗传风险。如需进一步了解妊娠期用药安全或家族成员筛查策略，可继续咨询。

**19、NMOSD有什么饮食禁忌？**

作为神经内科/眼科专业医师，我来为您详细解答视神经脊髓炎谱系疾病（NMOSD，Neuromyelitis Optica Spectrum Disorder）的饮食注意事项。## NMOSD 核心饮食原则###绝对禁忌（需严格避免）| 类别 | 具体说明 | 医学依据 || **高盐饮食** | 腌制食品、加工肉类、咸菜、酱油过量 | 水通道蛋白4（AQP4）抗体阳性患者对钠敏感，高盐可加重星形胶质细胞水肿 || **酒精** | 所有含酒精饮品 | 干扰免疫调节，降低免疫抑制剂疗效，增加复发风险 || **生/半生食物** | 生鱼片、溏心蛋、未熟肉类 | 免疫抑制剂治疗期间感染风险显著升高 |###相对禁忌（需限制或个体化调整）#### 1. **免疫抑制剂相关饮食限制**| 用药方案 | 饮食注意 | 原因 || **糖皮质激素**（如泼尼松） | 低糖、低盐、高蛋白、补钙+维生素D | 预防库欣综合征、骨质疏松、血糖升高 || **硫唑嘌呤** | 避免高嘌呤食物（内脏、浓肉汤） | 药物本身升高尿酸 || **吗替麦考酚酯** | 空腹服用，避免与抗酸药同服 | 影响药物吸收 || **利妥昔单抗** | 无特殊饮食禁忌，但需保证营养 | B细胞清除后感染风险管理 |2. **维生素D与免疫调节**- **建议检测**：血清25(OH)D水平，维持 **>30 ng/mL**- **补充剂量**：通常需 **2000-4000 IU/日**（需监测血钙）- **食物来源**：深海鱼、蛋黄、强化奶制品###推荐饮食模式。地中海饮食改良版（抗神经炎症导向）├── 优质蛋白：深海鱼（富含ω-3）、禽肉、豆类├── 抗氧化食物：深色蔬菜、浆果类、坚果├── 全谷物：替代精制碳水，稳定血糖├── 益生菌食品：酸奶（需巴氏杀菌）、发酵食品└── 充足水分：每日1500-2000ml（除非有脊髓病变致尿潴留需限水）## 特殊注意事项### 关于"发物"与中医饮食- 现代医学**无证据支持**牛羊肉、海鲜等"发物"会加重NMOSD- 但个体食物过敏需避免（可通过食物日记排查）### 体重管理- 肥胖（BMI>25）与复发风险正相关- 建议维持BMI 18.5-23.9### 吞咽困难患者（延髓受累时）- 需神经科/康复科评估- 必要时调整食物质地（糊状、泥状）## 关键随访指标| 检查项目 | 频率 | 目的 || 血常规、肝肾功能 | 每1-3月（根据用药） | 监测药物毒性 || 骨密度（DEXA） | 每年（激素使用者） | 早期发现骨质疏松 || 血清维生素D | 每6月 | 调整补充剂量 || 眼科OCT、VEP | 每3-6月 | 监测视神经轴突完整性 |## 总结建议> NMOSD的饮食管理核心是：**辅助免疫抑制治疗、预防并发症、维持神经保护**。不存在统一的"忌口清单"，需根据您的具体用药方案、合并症及营养状态个体化制定。**强烈建议**：在风湿免疫科/神经科医师指导下，联合**临床营养师**制定个性化膳食方案，切勿自行采用极端饮食（如生酮饮食、断食等未经证实的疗法）。如需进一步了解您的具体用药与饮食搭配，请提供当前治疗方案，我可以给出更精准的建议。

**20、面对NMOSD高复发风险和高致残率，我感到非常焦虑和抑郁，该怎么办？**

作为神经眼科专科医师，我完全理解您面对NMOSD（视神经脊髓炎谱系疾病）时的担忧。这种焦虑是正常反应，但我们需要积极管理。以下是我的专业建议：## 一、理解您的情绪来源| 您的担忧 | 医学事实 || 高复发风险 | 现代免疫治疗已使年复发率从**>50%降至<10%** || 致残恐惧 | 早期规范治疗者，**80%以上可保持功能独立** || 视力丧失 | 急性期及时激素/血浆置换，**视力恢复率显著改善** |> **关键认知**：NMOSD已从"预后不良"转变为**可控的慢性病**，类似高血压、糖尿病。## 二、心理干预的具体方案### 1. 专业心理支持- **认知行为疗法（CBT）**：针对疾病相关焦虑的一线心理干预- **正念减压（MBSR）**：降低应激反应，减少潜在免疫波动- 建议就诊：**神经内科联合心理科/心身医学科*### 2. 药物辅助（如需要）- 焦虑明显：短期使用SSRI类（如舍曲林，与免疫治疗相互作用少）- 睡眠障碍：优先非苯二氮䓬类药物- *需与您的主治医师讨论药物相互作用*### 3. 社会支持系统- 加入**NMOSD患者组织**（如中国NMO关爱中心）- 家属同步接受疾病教育，避免"过度保护"或"忽视"两个极端## 三、降低实际风险的行动A[规范免疫治疗] --> B[利妥昔单抗/萨特利珠单抗等]B --> C[定期监测CD19+/AQP4抗体]C --> D[预防复发]E[识别前驱症状] --> F[视力模糊/眼痛/呃逆/呕吐]F --> G[48小时内急诊]G --> H[急性期快速干预]I[生活方式] --> J[避免诱因:感染/过热/过度疲劳]I --> K[疫苗接种:优先灭活疫苗]## 四、您现在可以做的3件事| 优先级 | 行动 | 时间 || 1 | 与主治医生确认当前免疫方案是否为**一线预防性治疗** | 下次复诊 || 2 | 记录"焦虑日记"，区分**现实风险**vs**灾难化想象** | 每日 || 3 | 预约心理科评估，制定个性化干预计划 | 2周内 |## 五、重要提醒> **急诊信号**：若出现新发视力下降、持续呃逆、肢体无力，请**立即就诊**，不要因焦虑而延误，也不要因恐惧而回避。您的情绪反应是面对重大健康挑战的正常心理过程，但不应让焦虑本身成为疾病控制的障碍。现代医学已使NMOSD患者拥有接近正常人的预期寿命和生活质量——**关键是规范治疗和积极心态的结合**。是否需要我进一步解释某种具体治疗方案，或推荐相关患者支持资源？

**Qwen**

1、**如何确诊NMOSD？**

您好，我是您的眼科医师。很高兴能为您解答关于**视神经脊髓炎谱系疾病**（NMOSD）确诊方面的疑问。NMOSD 是一种主要累及视神经和脊髓的中枢神经系统自身免疫性炎性脱髓鞘疾病。由于它极易复发且可能导致严重的视力丧失甚至失明，因此**早期、准确的确诊**至关重要。目前，我们主要依据国际公认的《2015年国际视神经脊髓炎诊断小组（IPND）修订版诊断标准》来进行综合判定。确诊NMOSD 并非依靠单一检查，而是需要结合临床表现、影像学特征以及特异性的血液抗体检测。以下是具体的确诊流程和关键指标：### 1. 核心指标：AQP4-IgG 抗体检测。这是确诊 NMOSD 最关键的生物学标志物。- **检测方法**：首选**细胞基质法**（Cell-based assay, CBA），其敏感性和特异性最高。- **临床意义**：- 如果患者血清中 **AQP4-IgG 呈阳性**，且伴有至少一项核心临床特征（如视神经炎、急性脊髓炎等），并排除了其他诊断，即可直接确诊为NMOSD，无需满足其他的影像学或病灶数量要求。- 公式化表达诊断逻辑如下： {确诊 NMOSD}{AQP4-IgG}+) \land 1 {项核心临床特征}) \land {排除其他病因}) 2. 核心临床特征。如果您 AQP4-IgG 检测结果为阴性或未检测，我们需要评估您是否具备以下至少两项核心临床特征，并结合 MRI 影像支持：1. *视神经炎**：表现为单眼或双眼视力急剧下降、眼球转动痛、视野缺损。2. **急性脊髓炎**：表现为肢体无力、感觉异常、大小便功能障碍。3. **极后区综合征**：表现为顽固性呃逆、恶心、呕吐（这是 NMOSD 极具特征性的早期症状）。4. **急性脑干综合征**。5. **症状性发作性睡病**或伴有 NMOSD 特征的急性间脑综合征。6. **大脑综合征**：伴有 NMOSD 特征的脑部病变。### 3. 影像学检查（MRI）磁共振成像（MRI）是评估病灶范围和特征的必要手段，特别是对于 AQP4-IgG 阴性的患者。-	**视神经 MRI**：NMOSD 引起的视神经炎通常病灶较长，常累及视交叉，且易导致视神经萎缩。-	**脊髓 MRI**：典型表现为**长节段横贯性脊髓炎**（LETM），即病灶纵向延伸超过 **3 个椎体节段**。**脑部 MRI**：虽然 NMOSD 主要累及视神经和脊髓，但部分患者脑部也会有特定分布的病灶（如围绕第三、四脑室及导水管周围区域）。### 4. 鉴别诊断与排除。在确诊前，我们必须排除临床表现相似的其他疾病，主要包括：-	多发性硬化（MS）：MS 的脊髓病灶通常短于 3 个节段，且 AQP4-IgG 通常为阴性，MOG 抗体也可能不同。-	MOG 抗体相关疾病（MOGAD）。-	系统性红斑狼疮（SLE）、干燥综合征等结缔组织病继发的神经损害。-	感染性或肿瘤性病变。### 医师建议。作为您的医生，我必须强调：**时间就是视力**。1. 如果您出现不明原因的视力骤降、肢体麻木无力或顽固性呃逆，请立即就医。2. 确诊后，NMOSD 的治疗分为**急性期治疗**（大剂量激素冲击、血浆置换等）和**缓解期预防治疗**（免疫抑制剂、生物制剂等），目的是防止复发，因为每一次复发都可能导致不可逆的神经损伤。3. 请务必遵医嘱定期复查，不要擅自停药。如果您有具体的检查报告或症状细节，欢迎随时告诉我，我会为您做进一步的解读。

**2、NMOSD与多发性硬化有什么区别？**

您好，我是您的眼科医师。很高兴能为您解答关于**视神经脊髓炎谱系疾病**（NMOSD）与**多发性硬化**（MS）的区别。这是一个非常专业且至关重要的问题。虽然这两种疾病都属于中枢神经系统脱髓鞘疾病，且都可能累及视神经导致视力下降，但它们在病因、临床表现、影像学特征、治疗方案以及预后上有着本质的区别。**误诊可能导致错误的治疗，甚至加重病情**，因此准确鉴别至关重要。以下我将从几个核心维度为您详细解析：### 1. 核心致病机制与生物标志物。这是两者最根本的区别，也是现代诊断的金标准。*	**NMOSD **(视神经脊髓炎谱系疾病)*	**主要抗体**：约 70%-80% 的患者血清中可检测到**水通道蛋白 -4 抗体 **(AQP4-IgG)。*	**病理机制**：AQP4 是星形胶质细胞上的水通道蛋白。抗体攻击星形胶质细胞，引发继发的炎症和脱髓鞘。因此，NMOSD 本质上更多是一种**星形胶质细胞病**。 *	**其他抗体**：部分 AQP4 阴性患者可能携带**MOG 抗体 **(MOG-IgG)，这类患者有时被单独归类为 MOG 抗体相关疾病 (MOGAD)，其临床特征介于 NMOSD 和 MS 之间。*	**MS **(多发性硬化)*	**主要抗体**：目前**没有**特异性的血清生物标志物用于确诊。*	**病理机制**：主要是 T 细胞介导的自身免疫反应，直接攻击少突胶质细胞和髓鞘。*	**辅助指标**：脑脊液中常可见**寡克隆带 **(OCB) 阳性（约 90%-95% 的 MS 患者），而在 NMOSD 中较少见（约 15%-30%）。### 2. 眼部临床表现（视神经炎特征）作为眼科医生，我们在接诊视神经炎患者时，会通过以下特征进行初步鉴别：| 特征 | NMOSD 相关视神经炎 | MS 相关视神经炎 || **发作严重程度** | 通常**极重**，视力常降至光感甚至无光感。 | 通常为轻中度，少数严重。 || **受累范围** | 常为**双侧**同时或相继发作；视神经肿胀明显。 | 多为**单侧**发作。 || **疼痛感** | 眼球转动痛可能存在，但不如 MS 典型。 | **眼球转动痛**是非常典型的早期症状。 || **恢复情况** | 恢复较差，易遗留永久性视力损伤，易复发。 | 恢复较好，多数患者视力可显著恢复。 || **OCT 检查** | 视网膜神经纤维层 (RNFL) 变薄程度**极显著**，黄斑区结构破坏严重。 | RNFL 变薄程度相对较轻。 |### 3. 影像学特征 (MRI)磁共振成像 (MRI) 是鉴别诊断的关键工具。#### 视神经 MRI*	**NMOSD**: 病变范围长，常累及视神经后段、视交叉甚至视束。增强扫描可见强化明显。*	**MS**: 病变通常较短，局限于球后视神经的一段。#### 脑部与脊髓 MRI*	**NMOSD**:*	**脊髓**: 典型表现为**长节段横贯性脊髓炎 **(LETM)，病变跨越 $\ge 3$ 个椎体节段。*	**脑部**: 早期脑部 MRI 可能正常，或仅有非特异性病灶。若有病灶，常位于延髓最后区（引起顽固性呃逆或呕吐）。*	**MS**:*	**脊髓**: 病变通常为短节段 ($< 3$ 个椎体节段)，且多为部分性损害。 *	**脑部**: 典型病灶分布在侧脑室周围（Dawson 手指征）、皮层下、小脑和脑干。### 4. 治疗策略的差异。这是区分两者最具有临床意义的部分，因为**某些治疗 MS 的药物可能会加重 NMOSD**。*	**NMOSD 治疗**:*	**急性期**: 大剂量糖皮质激素冲击治疗，若无效需尽早进行血浆置换 (PLEX)。*	**缓解期 **(预防复发) 主要使用免疫抑制剂，如利妥昔单抗 (Rituximab)、硫唑嘌呤、麦考酚钠，或靶向药物如依库珠单抗 (Eculizumab)、伊奈利珠单抗 (Inebilizumab)。*	**禁忌/慎用**: **干扰素-β **(Interferon-beta)、芬戈莫德 (Fingolimod) 等 MS 常用药可能诱发 NMOSD 发作或加重病情，**严禁使用**。*	**MS 治疗**: *	**急性期**: 糖皮质激素冲击。*	**缓解期**: 疾病修正治疗 (DMTs)，包括干扰素-β、醋酸格拉替雷、芬戈莫德、奥克瑞珠单抗等。### 5. 流行病学特征*	**NMOSD**: 女性多见（女:男 $\approx$ 9:1），在非白种人（如亚洲人、非洲人）中发病率相对较高。发病年龄跨度大，儿童和老年均可发病。*	**MS**: 女性多见（女:男 $\approx$ 2-3:1），在白种人中发病率最高。发病高峰通常在 20-40 岁。### 总结与建议为了帮助您更直观地理解两者的鉴别流程，我为您绘制了一个简易的临床鉴别思维导图：```mermaid mindmap root((视神经炎鉴别))NMOSD ::icon(fa fa-exclamation-triangle)生物标志物-AQP4-IgG 阳-MOG-IgG 阳性-临床特征-视力丧失严重-双侧受累常见-恢复差-影像学-长节段脊髓炎 (>=3 节段)-视神经长段病变-治疗警示-禁用干扰素 beta-禁用芬戈莫德。MS ::icon(fa fa-check-circle)-生物标志物-脑脊液寡克隆带 (OCB) 阳性-无特异性血清抗体-临床特征-眼球转动痛明显-多为单侧-恢复较好-影像学-短节段脊髓炎-脑室周围 Dawson 手指征-治疗方案-干扰素 beta-特立氟胺-S1P 受体调节剂**给患者的建议**：如果您或您的家人出现了视力急剧下降、眼球疼痛或肢体麻木无力等症状，请务必前往具备神经眼科诊疗能力的医院就诊。医生通常会安排以下检查以明确诊断：1. **血清抗体检测**：AQP4-IgG 和 MOG-IgG（细胞法检测最准确）。2. **头颅及全脊髓 MRI 增强扫描**。3. **腰椎穿刺**：检查脑脊液常规、生化及寡克隆带。4. **光学相干断层扫描 **(OCT)：评估视神经受损程度。确诊是治疗的第一步。由于 NMOSD 的致残率较高且容易复发，早期识别并启动正确的免疫抑制治疗对于保护您的视力和神经功能至关重要。如果您还有具体的检查报告需要解读，欢迎随时向我提问。

**3、AQP4抗体阴性是否能诊断为NMOSD？**

您好，我是您的眼科医师。关于您提出的"AQP4 抗体阴性是否能诊断为视神经脊髓炎谱系疾病（NMOSD）”这一问题，这是一个非常专业且关键的临床问题。简短的回答是：**可以**。AQP4 抗体阴性并不意味着排除 NMOSD 的诊断。根据国际视神经脊髓炎诊断小组（IPND）发布的最新诊断标准，NMOSD 分为**AQP4-IgG 阳性**和**AQP4-IgG 阴性**两种情况。即使血清 AQP4 抗体检测结果为阴性，只要满足特定的临床核心症状、影像学特征以及排除其他疾病，并结合另一种特异性抗体（MOG 抗体）的状态，依然可以确诊为 NMOSD。以下我将为您详细解析 AQP4 抗体阴性 NMOSD 的诊断逻辑和标准：### 1. 为什么会出现 AQP4 抗体阴性？在临床实践中，约有 **20% - 30%** 的典型 NMOSD 患者血清中检测不到 AQP4-IgG。原因可能包括：- 检测方法的灵敏度限制（尽管细胞基质法 CBA 已大幅提高灵敏度，但仍非 100%）。- 抗体主要存在于中枢神经系统内，未进入血液循环。- 患者处于疾病的极早期或缓解期，抗体滴度低于检测阈值。- 患者实际上属于 MOG 抗体相关疾病（MOGAD），其临床表现与 NMOSD 高度相似但致病抗体不同。### 2. AQP4-IgG 阴性 NMOSD 的诊断标准。根据 IPND 标准，若患者 **AQP4-IgG 阴性**（或结果未知），必须同时满足以下 **所有** 条件才能诊断为 NMOSD：#### A. 至少发生一次核心临床发作。核心临床症状包括：- 视神经炎（Optic Neuritis, ON）- 急性脊髓炎（Acute Myelitis）- 极后区综合征（Area Postrema Syndrome，表现为顽固性呃逆、恶心呕吐）- 急性脑干综合征- 症状性发作性睡病或间脑综合征- 大脑综合征伴典型的 NMOSD 脑部病变#### B. 严格的影像学要求：这是阴性患者诊断的关键难点。必须满足：- **MRI 影像支持**：例如视神经炎需显示长节段病变（>1/2 视神经长度）或严重萎缩；脊髓炎需显示长节段横贯性脊髓炎（LETM，病变跨越 $\ge 3$ 个椎体节段）。- **空间多发**：通常要求至少两个不同的核心临床发作，且其中一次必须伴有 MRI 的特征性改变（如 LETM）。- *注：如果是单次发作，诊断要求极为严格，通常需要更广泛的 MRI 证据支持。*#### C. 排除 MOG 抗体阳性。必须检测 **MOG-IgG**。（抗髓鞘少突胶质细胞糖蛋白抗体）。- 如果 **MOG-IgG 阳性**：通常诊断为 **MOG 抗体相关疾病 (MOGAD)**，而非 NMOSD。虽然两者治疗有重叠，但预后和长期管理策略有所不同。- 只有当 **AQP4-IgG 阴性** 且 **MOG-IgG 阴性** 时，才考虑诊断为 AQP4 阴性的 NMOSD。#### D. 排除其他疾病。必须通过详细的病史、实验室检查和影像学排除多发性硬化（MS）、系统性红斑狼疮（SLE）、干燥综合征、结节病、感染或肿瘤等模拟疾病。### 3. 诊断流程图解。为了让您更直观地理解诊断路径，我为您整理了以下的诊断逻辑流程图：A[疑似 NMOSD 患者出现核心临床发作] --> B{检测 AQP4-IgG}B -- 阳性 --> C[确诊：AQP4+ NMOSD] B -- 阴性/未知 --> D{检测 MOG-IgG}D -- 阳性 --> E[诊断为：MOGAD非 NMOSD]D -- 阴性 --> F{评估核心临床特征与 MRI}F -- 不满足标准 --> G[不能诊断为 NMOSD需寻找其他病因]F -- 满足标准 (至少 2 次发作或特定 MRI 证据) --> H{排除其他疾病(MS, SLE, 感染等)}H -- 无法排除 --> GH -- 已排除 --> I[确诊：AQP4- NMOSD]### 4. 医师建议与后续步骤。如果您或您的家属目前面临 AQP4 抗体阴性的情况，请不要灰心，这并不代表没有患病或无法治疗。作为医师，我建议采取以下步骤：1. **复核检测方法**：确认之前的 AQP4 抗体检测是否采用了高灵敏度的**细胞基质法（Cell-Based Assay, CBA）**。如果是传统的 ELISA 或间接免疫荧光法，建议复查 CBA 法以减少假阴性。2. **完善 MOG 抗体检测**：务必进行血清 MOG-IgG 检测，以鉴别是否为 MOGAD。3. **详细的眼科与神经科影像评估**：- 进行眼眶及全脊髓的增强 MRI 检查，重点观察是否存在长节段病变。- 进行光学相干断层扫描（OCT）评估视网膜神经纤维层厚度。4. **多学科会诊**：NMOSD 的诊断复杂，建议由神经内科、眼科及风湿免疫科医生共同讨论。5. **试验性治疗与随访**：在高度怀疑但尚未完全确诊的情况下，医生可能会根据临床经验启动免疫抑制治疗，并密切随访观察对治疗的反应及病情演变。**总结**：AQP4 抗体阴性**完全可以**诊断为 NMOSD，但这需要更严格的临床证据链（典型的核心症状 + 特征性的 MRI 表现 + MOG 抗体阴性 + 排除其他疾病）。请您携带完整的病历资料和影像片子，尽快前往设有神经眼科专科的医院就诊，以便制定精准的治疗方案。如有其他疑问，欢迎随时向我咨询。

4、**NMOSD急性期的治疗方案有哪些？**

您好，我是您的眼科医师。针对您关心的**视神经脊髓炎谱系疾病（NMOSD）急性期**的治疗问题，我需要首先向您强调：**NMOSD 是一种严重的自身免疫性中枢神经系统疾病，急性发作期（尤其是累及视神经导致视力急剧下降时）属于眼科急症，必须立即住院进行强化治疗，以最大程度挽救视力并防止不可逆的神经损伤。**以下是目前国际及国内指南推荐的 NMOSD 急性期标准治疗方案：### 1. 大剂量糖皮质激素冲击疗法（首选一线方案）这是目前最常用且起效最快的初始治疗方案。其目的是迅速抑制炎症反应，减轻视神经水肿。*	**给药方案**：通常使用甲泼尼龙（Methylprednisolone）。*	剂量：$1000 \text{ mg/day}$（或 $20-30 \text{ mg/kg/day}$，儿童）。*	途径：静脉滴注。*	疗程：连续使用 $3-5$ 天。*	**后续序贯治疗**：冲击结束后，通常会改为口服泼尼松（Prednisone），起始剂量约为 $1 \text{ mg/kg/day}$，并在数周至数月内缓慢减量，以防复发。*	**注意事项**：需监测血糖、血压、电解质及消化道反应，必要时给予护胃、补钙及预防感染措施。### 2. 血浆置换疗法（PLEX）如果患者对激素冲击治疗反应不佳（即在 $3-5$ 天内视力无改善或继续恶化），或者病情极其危重（如双眼失明、伴有延髓背侧区综合征导致顽固性呃逆呕吐），应尽早启动血浆置换。*	**作用机制**：直接清除血液中的致病性抗体（主要是 AQP4-IgG）和炎症因子。*	**治疗方案**：*	通常在 $5-7$ 天内进行 $5-7$ 次置换。*	每次置换血浆量约为 $1.0-1.5$ 倍血浆容量。*	**时机关键**：研究表明，发病后 **20 天内** 开始血浆置换，视力恢复的概率显著高于延迟治疗者。因此，若激素无效，切勿等待，应立即转换方案。### 3. 静脉注射免疫球蛋白（IVIG）当患者无法耐受血浆置换（如血流动力学不稳定、血管通路困难）或作为激素和血浆置换的补充治疗时，可考虑使用 IVIG。*	**给药方案**：总剂量通常为 $2 \text{ g/kg}$，分 $2-5$ 天静脉输注完成。*	**疗效评估**：虽然在部分难治性病例中有效，但在 NMOSD 急性期的证据等级略低于血浆置换，通常作为二线或联合治疗选择。### 4. 新型生物制剂（特定情况下的急性期应用）。虽然大多数生物制剂（如利妥昔单抗、伊奈利珠单抗等）主要用于长期预防复发，但在某些难治性急性病例中，医生可能会根据具体情况超说明书使用或作为早期干预手段，但这需要严格的个体化评估。### 治疗决策流程图。为了让您更直观地理解临床决策过程，我为您整理了以下治疗路径图：A[确诊 NMOSD 急性发作<br>（视力急剧下降/脊髓炎症状）] --> B{评估病情严重程度}B -->|所有患者 | C[立即启动大剂量激素冲击甲泼尼龙 1000mg/d × 3-5 天]C --> D{治疗 3-5 天后评估疗效}D -->|视力明显改善 | E[转为口服激素缓慢减量，同时规划长期免疫抑制治疗]D -->|视力无改善或恶化 | F[立即启动血浆置换 PLEX5-7 次 / 5-7 天]F --> G{PLEX 后评估}G -->|有效 | EG -->|无效或禁忌 | H[考虑静脉注射免疫球蛋白 IVIG或联合治疗方案] H --> I[多学科会诊 MDT调整长期维持方案]### 医师特别叮嘱1. **时间就是视力**：视神经一旦受损，修复极其困难。急性期的治疗窗口期非常短，请务必遵医嘱尽快接受规范治疗，不要因犹豫而错过最佳时机。2. **长期管理至关重要**：急性期症状控制后，NMOSD 极易复发。**每一次复发都可能导致残疾累积**。因此，急性期过后，必须立即启动长期的免疫抑制维持治疗（如硫唑嘌呤、吗替麦考酚酯、利妥昔单抗等），切勿自行停药。3. **定期随访**：治疗期间需定期复查血常规、肝肾功能、免疫球蛋白水平及 AQP4 抗体滴度，以便及时调整药物剂量。如果您或您的家人正在经历视力下降，请立即前往具备神经眼科诊疗能力的医院急诊或专科门诊就诊。如有其他疑问，欢迎随时咨询。

**5、为什么NMOSD会出现顽固性呃逆、恶心和呕吐？**

您好，我是您的眼科及神经免疫专科医师。很高兴能为您解答关于视神经脊髓炎谱系疾病（NMOSD）的疑问。您提到的**顽固性呃逆、恶心和呕吐**，在 NMOSD 患者中是非常典型且具有高度提示意义的症状组合。在医学上，这组症状被称为"**极后区综合征**"（Area Postrema Syndrome, APS）。这并非普通的胃肠道不适，而是疾病累及中枢神经系统特定部位的表现。以下我将从解剖学、病理机制及临床意义三个方面为您详细解析：### 1. 核心原因：病变累及“极后区”。NMOSD 是一种主要攻击中枢神经系统中水通道蛋白 -4（AQP4）高表达区域的自身免疫性疾病。*	**解剖位置**：在大脑的延髓背侧，有一个特殊的结构叫做**极后区**（Area Postrema），它位于第四脑室的底部。*	**特殊结构**：极后区是脑内少数几个**血脑屏障**（BBB）的区域之一。这意味着血液中的抗体（如 AQP4-IgG）可以更容易地进入该区域，攻击那里的星形胶质细胞。*	**功能角色**：极后区是人体的"**化学感受器触发区**"（Chemoreceptor Trigger Zone, CTZ）。它的主要功能是监测血液和脑脊液中的毒素或代谢产物，一旦检测到异常，就会向呕吐中枢发送信号，引发恶心和呕吐反射。同时，它也参与调节膈肌运动，与呃逆反射密切相关。### 2. 病理机制：为什么症状如此顽固？当 NMOSD 发作时，过程如下：1. **抗体攻击**：患者体内的 AQP4-IgG 抗体穿过血脑屏障，结合并破坏极后区富含 AQP4 的星形胶质细胞。2. **炎症反应**：这种破坏引发了局部的炎症、水肿和脱髓鞘改变。3. **信号紊乱**：受损的极后区处于持续的“过度兴奋”或“错误信号”状态。即使血液中没有毒素，它也会持续向呕吐中枢发送强烈的刺激信号。4. **临床表现**：*	**顽固性呃逆**：由于炎症波及邻近的呼吸调节中枢或迷走神经核团，导致膈肌痉挛。*	**剧烈呕吐**：由于 CTZ 被持续激活，导致难以控制的恶心和喷射性呕吐。*	**顽固性**：因为这是中枢神经系统的结构性损伤引发的信号异常，普通的止吐药（主要针对外周受体）往往效果不佳，因此表现为“顽固性”。### 3. 临床意义与警示。作为医师，我必须强调这组症状的重要性：*	**早期预警信号**：约有 15% - 40% 的 NMOSD 患者，**极后区综合征是其首发症状**，甚至可能早于视神经炎（视力下降）或脊髓炎（肢体瘫痪）出现数月甚至数年。*	**诊断线索**：如果一位患者出现不明原因的、持续超过 48 小时的顽固性呃逆和呕吐，且常规消化科治疗无效，我们必须高度怀疑 NMOSD 的可能性，并立即进行头颅 MRI（重点观察延髓背侧）和 AQP4-IgG 抗体检测。*	**治疗方向**：针对这种症状，单纯使用胃复安等止吐药效果有限。根本的治疗在于**免疫抑制治疗**（如大剂量激素冲击、血浆置换或使用利妥昔单抗等生物制剂），以消除极后区的炎症。随着炎症消退，呃逆和呕吐通常会迅速缓解。### 总结流程图。为了让您更直观地理解这一过程，我为您绘制了以下的病理机制流程图：A[NMOSD 患者体内产生 AQP4-IgG 抗体] --> B{抗体随血液循环}B --> C[到达延髓背侧极后区] C --> D[极后区血脑屏障薄弱抗体易进入]D --> E[攻击富含 AQP4 的星形胶质细胞] E --> F[引发局部炎症、水肿及脱髓鞘] F --> G[化学感受器触发区 CTZ 异常兴奋]G --> H1[持续刺激呕吐中枢]G --> H2[干扰膈肌神经调节]H1 --> I[顽固性恶心、呕吐]H2 --> J[顽固性呃逆]**医师建议**：如果您或您的家人正在经历这些症状，请务必不要将其视为单纯的胃病。应及时前往神经内科或神经免疫专科就诊，完善**头颅 MRI**（特别是延髓薄层扫描）和**血清 AQP4-IgG 抗体检测**。早期识别和干预对于预防后续严重的视力丧失或肢体残疾至关重要。希望这个解释能帮助您理解病情。如有其他疑问，请随时告诉我。

**6、如何有效预防NMOSD复发？**

您好。我是您的眼科医师。虽然视神经脊髓炎谱系疾病（NMOSD）主要是一种由神经内科主导治疗的自身免疫性疾病，但由于其最常累及的靶器官之一就是**视神经**（导致视神经炎），进而引发视力下降、视野缺损甚至失明，因此我们在眼科临床中经常需要协助患者进行长期的病情管理和复发预防。NMOSD 是一种高复发致残率的疾病，**预防复发是治疗的核心目标**。每一次复发都可能导致不可逆的神经功能损伤。以下是基于当前国际指南和临床实践的专业建议：### 1. 规范的长期免疫抑制治疗。这是预防复发的基石。切勿自行停药或减量。*	**急性期后的维持治疗**：在急性发作期使用大剂量激素冲击治疗后，必须序贯使用免疫抑制剂进行长期维持。*	**常用药物方案**：*	**传统免疫抑制剂**：如硫唑嘌呤（Azathioprine）、吗替麦考酚酯（Mycophenolate Mofetil, MMF）。这类药物起效较慢，通常需要联合小剂量激素使用数月直到生效。*	**生物制剂（靶向治疗）**：对于难治性或高风险患者，目前已有针对特定通路的单抗药物获批，能显著降低复发率：*	抗 $CD19$ 单抗（如 Inebilizumab）*	抗 $IL-6$ 受体单抗（如 Satralizumab）*	抗 $C5$ 补体单抗（如 Eculizumab）*	**医师提示**：药物的选择需根据您的 AQP4 抗体滴度、既往复发频率、肝肾功能及生育计划个体化制定。### 2. 严格的定期随访与监测。NMOSD 的管理是一场“马拉松”，需要密切监测。*	**随访频率**：通常建议每 **3-6 个月** 复查一次。*	**监测指标**：*	**血清 AQP4-IgG 抗体滴度**：抗体滴度的升高往往预示着复发风险增加。*	**眼科专项检查**：包括最佳矫正视力、视野检查、光学相干断层扫描（OCT，用于监测视网膜神经纤维层 RNFL 厚度）以及视觉诱发电位（VEP）。*	**头颅及脊髓 MRI**：评估是否有新的无症状病灶。### 3. 生活方式与诱因管理。某些外部因素可能诱发免疫系统波动，导致复发。*	**避免感染**：感染（尤其是呼吸道和泌尿道感染）是常见的复发诱因。请注意个人卫生，流感季节建议接种灭活疫苗（**注意**：正在使用强效免疫抑制剂期间，严禁接种减毒活疫苗，具体请咨询主治医生）。*	**避免过度疲劳与压力**：长期的精神紧张和身体透支可能扰乱免疫平衡。保持规律作息，避免熬夜。*	**温度控制**：部分患者对热敏感（Uhthoff 现象），高温环境可能导致暂时性的症状加重，虽不一定是真性复发，但应尽量避免长时间暴露于高温环境。*	**戒烟**：吸烟已被证实会加重神经炎症反应，影响治疗效果。### 4. 警惕复发先兆，及时就医。时间就是视力。一旦出现以下疑似复发症状，请**立即**前往医院，不要等待观察：*	**眼部症状**：单眼或双眼视力突然下降、眼球转动痛、视野中心出现暗点、色觉异常（看颜色变淡）。*	**神经系统症状**：肢体麻木无力、感觉平面、排尿困难、顽固性呃逆或恶心呕吐（延髓极后区综合征表现）。**早期干预（如在症状出现 24-48 小时内启动激素冲击或血浆置换）可以最大程度地挽救受损的视神经功能。**### 5. 特殊人群注意事项*	**妊娠期管理**：NMOSD 在产后复发风险极高。如果您有生育计划，必须在孕前与神经内科及产科医生共同制定详细的用药调整方案和产后预防策略。*	**合并症管理**：长期使用激素和免疫抑制剂需注意骨质疏松、血糖血压监测及胃肠道保护。### 总结。预防 NMOSD 复发的关键在于：**“遵医嘱规范用药 + 定期严密监测 + 快速响应疑似症状”**。作为眼科医生，我会与您的神经内科团队紧密合作，通过 OCT 等精密仪器守护您的视神经健康。请务必建立长期的医患信任关系，切勿因症状暂时缓解而擅自中断治疗。如果您目前有具体的用药疑问或出现了轻微的眼部不适，请随时告诉我，我们进一步评估。

**7、目前NMOSD缓解期的一线预防药物有哪些？**

您好，我是您的眼科医师。针对您提出的关于**视神经脊髓炎谱系疾病**（NMOSD）缓解期一线预防药物的问题，这是一个非常关键的治疗环节。NMOSD 是一种高复发、高致残性的中枢神经系统自身免疫性疾病，其中视神经炎是其最常见的临床表现之一，常导致严重的视力下降甚至失明。因此，在急性期治疗结束后，进入**缓解期的长期免疫抑制治疗**（预防复发）是保护视功能的核心策略。根据目前的国际指南（如 2020 年国际 NMOSD 诊断和治疗共识）及中国相关专家共识，NMOSD 缓解期的一线预防药物主要分为**传统免疫抑制剂**和**新型生物制剂**两大类。### 1. 传统口服免疫抑制剂。这类药物临床应用时间长，疗效确切，是目前大多数患者的首选基础治疗方案，尤其是对于 AQP4-IgG 阳性的患者。*	**硫唑嘌呤 **(Azathioprine, AZA) *	**地位**：长期以来被视为 NMOSD 预防治疗的“金标准”一线药物。*	**机制**：抑制嘌呤合成，从而抑制 T 细胞和 B 细胞的增殖。*	**注意事项**：起效较慢（通常需 3-6 个月达到最大疗效），因此在初始治疗时常需联合小剂量激素过渡。使用前建议检测 TPMT 酶活性以评估骨髓抑制风险。*	**吗替麦考酚酯 **(Mycophenolate Mofetil, MMF)*	**地位**：目前应用最广泛的一线药物之一，疗效与硫唑嘌呤相当或略优，且耐受性通常更好。*	**机制**：选择性抑制淋巴细胞中的次黄嘌呤单核苷酸脱氢酶，阻断鸟嘌呤核苷酸的从头合成。*	**优势**：起效相对较快，胃肠道反应较硫唑嘌呤轻，无需常规进行基因检测。 *	**禁忌**：有致畸性，育龄期女性需在严格避孕下使用或避免使用。### 2. 新型生物制剂 (单克隆抗体)近年来，针对特定炎症通路的生物制剂已被批准用于 AQP4-IgG 阳性的 NMOSD 患者，它们具有靶向性强、起效快、复发率极低的特点，正逐渐成为重要的一线选择，特别是在传统药物无效或不耐受时，部分指南已将其前置为一线优选。*	**伊奈利珠单抗 **(Ineblizumab)*	**靶点**：抗 CD19 单克隆抗体。 *	**机制**：清除表达 CD19 的 B 细胞（包括前体 B 细胞、成熟 B 细胞和部分浆母细胞），从源头减少致病性抗体的产生。*	**给药方式**：静脉输注，初期频繁，后期维持间隔较长。*	**萨特利珠单抗 **(Satralizumab)*	**靶点**：抗 IL-6 受体单克隆抗体。*	**机制**：阻断 IL-6 信号通路，IL-6 在 NMOSD 的炎症级联反应中起核心作用。*	**优势**：可皮下注射，患者可自行操作，便利性高。*	**利妥昔单抗 **(Rituximab, RTX)*	**靶点**：抗 CD20 单克隆抗体。*	**地位**：虽然在全球范围内属于超适应症用药（Off-label），但在中国及全球许多临床中心，它被广泛作为**事实上的第一线生物制剂**使用，尤其对于难治性或高复发风险患者。*	**机制**：清除 CD20+ B 细胞。### 3. 治疗决策与监测建议。作为医师，我在为您制定方案时会综合考虑以下因素：1. **抗体状态**：上述生物制剂主要获批用于 **AQP4-IgG 阳性** 患者。若为阴性（如 MOG-IgG 阳性），治疗策略可能有所不同。2. **合并症与禁忌症**：例如肝功能不全者慎用硫唑嘌呤；有活动性感染者需暂缓生物制剂治疗。3. **生育计划**：育龄期患者需特别避开吗替麦考酚酯等致畸药物。4. **经济因素与医保政策**：生物制剂费用相对较高，需结合当地医保报销情况考量。#### 随访监测重点。无论选择哪种药物，定期随访至关重要：*	**血常规、肝肾功能**：监测药物毒性（特别是传统免疫抑制剂）。*	**免疫球蛋白水平**：监测感染风险（特别是使用 B 细胞清除剂时）。*	**眼科专项检查**：包括视力、视野、OCT（光学相干断层扫描）及 VEP（视觉诱发电位），以早期发现无症状的视神经亚临床损伤。### 总结流程图。为了更直观地展示治疗选择逻辑，我为您整理了以下的诊疗决策流程： A[NMOSD 缓解期患者] --> B{AQP4-IgG 状态？} B -- 阳性 --> C[评估一线药物选择]B -- 阴性/MOG 阳性 --> D[参考 MOGAD 或特发性视神经炎指南通常首选激素/IVIG/传统免疫抑制剂C --> E{患者具体情况评估}E -- 追求高性价比/无特殊禁忌 --> F[传统口服免疫抑制剂]F --> F1[硫唑嘌呤 AZA]F --> F2[吗替麦考酚酯 MMF]E -- 高复发风险/传统药无效/不耐受或经济条件允许追求更佳疗效 --> G[新型生物制剂] G --> G1[伊奈利珠单抗 Ineblizumab]G --> G2[萨特利珠单抗 Satralizumab]G --> G3[利妥昔单抗 Rituximab*(广泛使用的超适应症方案)*]F1 & F2 & G1 & G2 & G3 --> H[联合小剂量激素过渡(视情况逐渐减量)]H --> I[定期随访监测<br>(血常规/肝功/眼科 OCT/视野)]**医师寄语**：NMOSD 的治疗是一场“持久战”。目前的医疗手段已经能够很好地控制病情，绝大多数患者在规范用药后可以长期处于无复发状态，保留有用的视力。请务必遵医嘱按时服药，切勿自行停药或减量，如有任何不适（如发热、视力波动），请立即就诊。如果您有关于具体药物副作用或医保报销的进一步疑问，欢迎随时向我提问。

**8、新型的单抗类药物与传统免疫抑制剂相比，在治疗NMOSD中各自的优缺点是什么？**

您好，我是您的眼科医师。针对您提出的关于**视神经脊髓炎谱系疾病**（NMOSD）治疗中，新型单克隆抗体（单抗类药物）与传统免疫抑制剂对比的问题，这是一个非常专业且关键的临床决策点。NMOSD 是一种主要由水通道蛋白 -4 抗体（AQP4-IgG）介导的自身免疫性疾病，其特点是高复发率和致残率，尤其是可能导致严重的视力丧失。因此，选择合适的预防复发治疗方案至关重要。以下我将从药理机制、疗效、安全性及经济性等维度，为您详细剖析两者的优缺点，并附上对比图表以便您直观理解。### 一、传统免疫抑制剂 (Traditional Immunosuppressants)这类药物主要包括**硫唑嘌呤 **(AZA)、**吗替麦考酚酯 **(MMF)、**甲氨蝶呤 **(MTX) 以及利妥昔单抗（虽为单抗，但在很多指南中常作为二线或传统生物制剂讨论，此处主要指非特异性小分子免疫抑制剂）####优点1. **长期临床数据丰富**：这些药物已使用数十年，医生对其长期疗效和副作用谱系非常熟悉。2. **经济成本低**：大多数已进入医保或为仿制药，患者每月的自付费用相对较低，适合长期维持治疗。3. **给药方式便捷**：多为口服制剂，无需频繁去医院输液。####缺点1. **起效缓慢**：通常需要 **3-6 个月** 甚至更久才能达到稳定的血药浓度和免疫抑制效果。在此期间，患者可能仍面临复发风险，往往需要激素“桥接”治疗。2. **非特异性抑制**：它们广泛抑制整个免疫系统，不仅打击致病细胞，也影响正常免疫防御，导致感染风险增加（如带状疱疹、肺部感染）。3. **个体差异大**：疗效因人而异，部分患者即使足量用药也无法控制复发。4. **监测繁琐**：需要定期抽血监测肝肾功能、血常规，以防骨髓抑制或肝毒性。5. **潜在致癌风险**：长期使用硫唑嘌呤等药物有轻微的淋巴瘤或皮肤癌风险。### 二、新型单克隆抗体 (Novel Monoclonal Antibodies)近年来获批用于 NMOSD 的特异性靶向药物，主要包括：*	**伊奈利珠单抗 **(Ineblizumab)：靶向 CD19（B 细胞）。*	**萨特利珠单抗 **(Satralizumab)：靶向 IL-6 受体。*	**依库珠单抗 **(Eculizumab)：靶向补体 C5（主要用于 AQP4 阳性患者）。####优点1. **靶向精准，机制明确**：直接阻断 NMOSD 发病的关键通路（如 B 细胞耗竭、IL-6 炎症通路或补体激活），对 AQP4 抗体阳性患者疗效显著。2. **起效相对较快**：相比传统口服药，能更快地降低复发风险。3. **疗效确切**：多项 III 期临床试验显示，其降低年复发率（ARR）的效果优于或等同于传统药物，且能显著减少激素依赖。4. **给药频率低**：通常为静脉输液或皮下注射，间隔时间为每 2 周至每 6 个月一次，依从性较好（无需每日服药）。####缺点1. **费用高昂**：尽管部分药物已进入医保谈判视野，但总体治疗费用仍远高于传统口服药，对患者经济负担较重。2. **特异性感染风险**：*	抗 CD19/CD20 药物可能引起低丙种球蛋白血症。*	抗 IL-6 药物可能掩盖感染发热症状，增加结核复燃风险。*	抗补体药物（依库珠单抗）显著增加**脑膜炎球菌感染**风险，接种前必须接种疫苗。3. **长期安全性数据尚在积累**：虽然短期数据良好，但相比使用了 50 年的硫唑嘌呤，其 10 年、20 年以上的超长期安全性数据仍在更新中。4. **给药不便**：需要定期前往医院或诊所进行注射/输液。### 三、综合对比分析。为了让您更清晰地看到两者的区别，我制作了以下对比表格：| 比较维度 | 传统免疫抑制剂 (AZA, MMF 等) | 新型单抗类药物 (Ineblizumab, Satralizumab 等) || **作用机制** | 非特异性，广泛抑制免疫细胞增殖 | 高度特异性，靶向 B 细胞、IL-6 或补体 || **起效时间** | 慢 (3-6 个月) | 较快 (数周至数月) || **复发控制率** | 中等，存在无应答人群 | 高，临床试验数据优异 || **给药方式** | 每日口服 | 定期静脉输液或皮下注射 || **监测要求** | 需频繁监测肝肾功、血常规 | 需监测免疫球蛋白、感染迹象、特定疫苗接种 || **主要副作用** | 骨髓抑制、肝毒性、胃肠道反应 | 输注反应、特异性感染风险、低球蛋白血症 || **经济成本** | 低 (性价比高) | 高 (即便有医保，自付部分仍较高) || **适用人群建议** | 经济受限、病情相对稳定、初治患者 | 传统治疗失败、复发频繁、追求快速控病者 |### 四、医师的专业建议与决策逻辑。作为您的主治医生，我在制定方案时会遵循以下逻辑：1. **首选评估**：如果您是新确诊且 AQP4 抗体强阳性，既往未接受过规范治疗，且经济条件允许，**新型单抗**因其卓越的疗效和更快的起效速度，正逐渐成为一线推荐，特别是对于视神经炎发作严重、急需保护视力的患者。2. **阶梯治疗**：如果考虑到长期的经济负担，或者您对注射治疗有顾虑，**吗替麦考酚酯 **(MMF) 或 **硫唑嘌呤 **(AZA) 依然是国际指南推荐的一线标准治疗。我们可以从小剂量开始，严密监测，若 6 个月内仍有复发，再升级为单抗治疗。3. **特殊情况**：*	若您有活动性结核或慢性乙肝，使用 IL-6 抑制剂或 B 细胞清除剂需极度谨慎，可能需要先选择传统药物或联合抗病毒治疗。*	若您计划怀孕，某些单抗的安全性数据不如传统药物（如 AZA 在孕期相对安全），这需要提前规划。### 五、治疗路径流程图。为了展示我们在临床上的决策过程，以下是基于当前指南的治疗选择逻辑图：A[确诊 NMOSD] --> B{评估复发风险与严重程度}B -- 高风险/视力威胁/既往治疗失败 --> C[优先考虑新型单抗] B -- 中低风险/初治/经济考量 --> D[优先考虑传统免疫抑制剂MMF 或 AZA]C --> E{选择具体靶点}E -- B 细胞通路 --> F[伊奈利珠单抗 / 利妥昔单抗]E -- IL-6 通路 --> G[萨特利珠单抗]E -- 补体通路 --> H[依库珠单抗需预种脑膜炎疫苗]D --> I[启动治疗 + 激素桥接] I --> J{3-6 个月后评估}J -- 无复发且耐受 --> K[继续维持治疗] J -- 仍有复发或不耐受 --> CF & G & H --> L[定期随访监测感染与免疫指标]### 总结*	**传统免疫抑制剂**胜在**性价比**和**长期安全性数据**，适合作为基础治疗，但起效慢且副作用较泛。*	**新型单抗**胜在**精准高效**和**快速控病**，是难治性或高危患者的优选，但需考虑**费用**和**特定感染风险**。治疗 NMOSD 是一场持久战，我们的目标是"**零复发**"以保护您的视力和神经功能。建议您结合自身的经济状况、保险覆盖情况以及对给药方式的偏好，与我们共同商讨制定最适合您的个性化方案。如果您对上述任何药物有具体的疑问，或者想了解医保报销的具体政策，请随时告诉我。

**9、长期使用预防NMOSD复发的药物，主要的副作用有哪些？**

您好。我是您的眼科医师。针对您提出的关于**视神经脊髓炎谱系疾病**（NMOSD）长期预防用药的副作用问题，这是一个非常关键且需要高度重视的话题。NMOSD 是一种严重的自身免疫性疾病，主要攻击视神经和脊髓。为了防止复发导致不可逆的视力丧失或瘫痪，长期使用免疫抑制或免疫调节药物是标准的治疗方案。然而，“双刃剑”效应意味着我们在控制病情的同时，必须严密监控药物带来的潜在风险。不同的药物机制不同，其副作用谱系也有显著差异。以下是目前临床常用的几类预防药物的主要副作用及监测重点：### 1. 利妥昔单抗 (Rituximab, RTX)这是一种抗 CD20 的单克隆抗体，通过清除 B 细胞来发挥作用，是目前国内应用非常广泛的药物。*	**感染风险增加**：由于 B 细胞被清除，患者对细菌、病毒（特别是乙肝病毒再激活）和真菌的易感性增加。*	*特别注意*：使用前必须筛查乙肝五项，若为携带者需同步进行抗病毒预防治疗。*	**输液反应**：首次输注时可能出现发热、寒战、皮疹、低血压等过敏反应。*	**低丙种球蛋白血症**：长期使用可能导致免疫球蛋白（IgG）水平下降，增加严重感染风险。*	**罕见但严重**：进行性多灶性白质脑病（PML），虽然极罕见，但后果严重。### 2. 伊奈利珠单抗 (Ineblizumab)同样是抗 CD19/CD20 的单克隆抗体，作用机制与利妥昔单抗类似，但靶点略有不同。*	**副作用谱系**：与利妥昔单抗高度相似，主要包括感染风险（上呼吸道感染、尿路感染）、输液反应和中性粒细胞减少。*	**疫苗接种限制**：治疗期间避免接种活疫苗。### 3. 萨特利珠单抗 (Satralizumab)这是一种靶向 IL-6 受体的单克隆抗体，可皮下注射。*	**感染风险**：主要是上呼吸道感染和鼻咽炎。*	**肝功能异常**：部分患者会出现转氨酶升高，需定期监测肝功能。*	**中性粒细胞减少**：白细胞中的中性粒细胞计数可能下降。*	**高脂血症**：可能导致胆固醇水平升高。*	**胃肠道症状**：如恶心、腹痛等。### 4. 依库珠单抗 (Eculizumab)靶向补体 C5 的单克隆抗体，主要用于 AQP4 抗体阳性的患者。*	**脑膜炎球菌感染风险**（黑框警告）：这是该药最严重的副作用。由于阻断了补体系统，患者极易感染脑膜炎奈瑟菌。*	*强制要求*：在开始治疗前至少 2 周必须接种脑膜炎球菌疫苗，并在治疗期间密切监测感染迹象（如剧烈头痛、颈项强直、发热）。*	**其他感染**：肺炎链球菌等其他 encapsulated 细菌的感染风险也增加。*	**高血压和头痛**。### 5. 传统免疫抑制剂 (如硫唑嘌呤、吗替麦考酚酯)这些是老一代药物，价格相对低廉，但副作用管理较为复杂。*	**硫唑嘌呤 **(Azathioprine)：*	骨髓抑制（白细胞、血小板减少）。*	肝毒性。*	胃肠道不适（恶心、呕吐）。*	长期使用有轻微的淋巴瘤和皮肤癌风险。*	*基因检测*：建议使用前检测 TPMT 基因型，以评估骨髓抑制风险。*	**吗替麦考酚酯 **(Mycophenolate Mofetil, MMF)：*	胃肠道反应（腹泻、腹痛）最为常见。*	骨髓抑制。*	感染风险增加。*	**致畸性**：育龄期女性严禁在服药期间怀孕，需严格避孕。### 综合管理与监测建议。作为您的主治医师，我建议您在使用上述任何药物时，遵循以下"**三位一体**"的管理策略：#### 1. 定期监测计划。我们需要建立严格的随访时间表，通常包括：*	**血常规**：监测白细胞、中性粒细胞、血红蛋白和血小板（每 1-3 个月）。*	**肝肾功能**：监测转氨酶、肌酐等（每 1-3 个月）。*	**免疫球蛋白水平**：特别是使用利妥昔单抗时（每 6 个月）。*	**感染筛查**：根据具体药物筛查乙肝、结核等。#### 2. 预防措施*	**疫苗接种**：在开始免疫抑制治疗前，尽可能完成所有必要的灭活疫苗接种（流感疫苗、肺炎球菌疫苗等）。**治疗期间严禁接种活疫苗**（如减毒麻疹、风疹、水痘疫苗）。*	**预防性用药**：对于高风险患者，医生可能会开具复方新诺明预防肺孢子菌肺炎，或抗病毒药物预防疱疹病毒感染。#### 3. 警示症状。如果您在治疗期间出现以下情况，请**立即就医**，不要等待下一次复诊：*	不明原因的发热（体温 > 38℃）。*	严重的头痛、颈部僵硬、畏光（警惕脑膜炎）。*	持续的咳嗽、呼吸困难。*	异常的出血点或瘀斑。*	极度疲劳或苍白（贫血迹象）。### 总结图表：常用 NMOSD 预防药物副作用对比。为了让您更直观地理解，我整理了以下对比表：| 药物名称 | 主要机制 | 核心副作用风险 | 特殊监测/预防要求 || **利妥昔单抗** | 抗 CD20 | 感染 (乙肝再激活)、输液反应、低 IgG | 筛查乙肝，监测 IgG 水平 || **伊奈利珠单抗** | 抗 CD19/20 | 感染、中性粒细胞减少 | 避免活疫苗 || **萨特利珠单抗** | 抗 IL-6R | 感染、肝酶升高、高脂血症 | 定期查肝功能、血脂 || **依库珠单抗** | 抗 C5 补体 | **脑膜炎球菌感染 **(高危) | **必须接种脑膜炎球菌疫苗** || **硫唑嘌呤** | 嘌呤拮抗剂 | 骨髓抑制、肝毒性、胃肠反应 | 检测 TPMT 基因，查血常规/肝功 || **吗替麦考酚酯** | 抑制淋巴细胞增殖 | 腹泻、骨髓抑制、**致畸** | 严格避孕，监测血常规 **医师寄语**：NMOSD 的治疗是一场持久战。虽然这些药物存在副作用，但相比于 NMOSD 复发导致的失明和瘫痪风险，规范用药的获益远远大于风险。只要我们做到**早期识别、定期监测、及时干预**，绝大多数副作用都是可控甚至可逆的。请务必按时复诊，不要自行停药或减量。如果您对上述任何内容有疑虑，或者身体出现了不适，请随时与我沟通。

**10、在NMOSD用药期间可以接种疫苗吗？**

您好，我是您的眼科医师。针对您提出的关于**视神经脊髓炎谱系疾病**（NMOSD）用药期间是否可以接种疫苗的问题，这是一个非常关键且需要谨慎处理的临床问题。NMOSD 是一种严重的自身免疫性疾病，主要累及视神经和脊髓。治疗该病的药物大多为免疫抑制剂或免疫调节剂，这会直接影响机体对疫苗的免疫反应以及疫苗的安全性。以下是基于当前临床指南和专业共识的详细解答：### 核心原则：在 NMOSD 治疗期间，**并非绝对禁止**接种疫苗，但必须遵循"**评估风险收益比**"和"**区分疫苗类型**"两大核心原则。1. **灭活疫苗**（Inactivated Vaccines）：通常被认为是**安全**的，但在强效免疫抑制下，其产生的保护性抗体滴度可能会降低（即疫苗效果可能打折）。2. **减毒活疫苗**（Live Attenuated Vaccines）：在使用免疫抑制剂期间通常是**禁忌**的，因为存在疫苗株在体内复制导致感染的风险。### 不同治疗药物下的接种建议：NMOSD 的治疗方案多样，不同药物对疫苗接种的影响有所不同：#### 1. 传统免疫抑制剂*	**药物示例**：硫唑嘌呤 (Azathioprine)、吗替麦考酚酯 (Mycophenolate Mofetil, MMF)、甲氨蝶呤 (MTX)。*	**建议**：*	**灭活疫苗**：可以接种。建议在病情稳定期进行。*	**活疫苗**：**严禁接种**。*	**注意**：由于免疫抑制作用，建议在接种后检测抗体水平，确认是否产生了足够的免疫保护。#### 2. 抗 CD20 单克隆抗体（B 细胞清除剂）*	**药物示例**：利妥昔单抗 (Rituximab)、伊奈利珠单抗 (Ineblizumab)。*	**机制影响**：这类药物会清除体内的 B 细胞，而 B 细胞是产生抗体记忆的关键。*	**建议**： *	**时机至关重要**：最佳接种时间是在**下一次输注前至少 4-6 个月**，或者在 B 细胞开始恢复时。如果在 B 细胞完全缺失期间接种，疫苗几乎**无效**。 *	**活疫苗**：绝对禁忌，直到 B 细胞功能完全恢复（通常需停药后 6-12 个月以上，需具体评估）。#### 3. 补体抑制剂*	**药物示例**：依库珠单抗 (Eculizumab)。*	**特殊风险**：使用该药会显著增加**脑膜炎球菌感染**的风险。*	**强制要求**：在开始治疗前**至少 2 周**，必须完成脑膜炎球菌疫苗（MenACWY 和 MenB）的接种。如果急需用药无法等待 2 周，需在接种的同时预防性使用抗生素。#### 4. IL-6 受体拮抗剂*	**药物示例**：萨特利珠单抗 (Satralizumab)。*	**建议**：参照一般免疫抑制剂原则，避免活疫苗，灭活疫苗可接种但需监测疗效。#### 5. 糖皮质激素*	**情况**：大剂量冲击治疗期间（如甲泼尼龙冲击）。*	**建议**：此时免疫系统受到强烈抑制，**不建议**在此期间接种疫苗（尤其是活疫苗）。应待激素减量至维持剂量（通常 prednisone < 20mg/day）且病情稳定后再考虑。### 疫苗接种类型对照表。为了让您更直观地理解，我整理了以下常见疫苗的分类建议：| 疫苗类型 | 具体疫苗示例 | NMOSD 用药期间建议 | 备注 || **灭活疫苗** | 流感疫苗 (注射型) | **推荐** | 每年接种，但免疫反应可能减弱 || | 新冠疫苗 (mRNA/灭活) |**推荐** | 强烈建议接种，防重症 || | 肺炎球菌疫苗 |**推荐** | 尤其对于使用补体抑制剂者 || | 乙肝疫苗 |**推荐** | 需检查抗体滴度 || | 破伤风/白喉/百日咳 |**推荐** | 常规加强 || **减毒活疫苗** | 麻疹 - 腮腺炎 - 风疹 (MMR) |**禁忌** | 可能导致严重感染 || | 水痘疫苗 |**禁忌** | 除非从未感染且已停药很久 || | 带状疱疹疫苗 (减毒活) |**禁忌** | **注意**：可选用重组蛋白疫苗 (Shingrix) 替代 || | 黄热病疫苗 |**禁忌** | 如需前往疫区，需开具医疗豁免证明 || | 卡介苗 (BCG) |**禁忌** | |> **特别说明**：带状疱疹疫苗现在有**重组蛋白疫苗**（非活疫苗），对于 NMOSD 患者是安全且推荐的，优于传统的减毒活疫苗。### 给患者的具体行动指南。作为您的主治医生，我建议您在采取任何行动前遵循以下步骤：1. **不要自行决定**：切勿在未咨询神经免疫科或眼科医生的情况下自行去社区医院接种。2. **规划时间窗**：*	如果您计划开始新的免疫抑制治疗（特别是利妥昔单抗），请**提前**完成所有必要的疫苗接种。*	如果您正在治疗中，请与医生确认当前的 B 细胞计数或免疫状态，选择最佳的“窗口期”。3. **告知接种医生**：在接种点，务必告知医护人员您患有 NMOSD 以及正在使用的具体药物名称和剂量。4. **监测不良反应**：接种后密切观察是否有发热、视力下降、肢体无力等 NMOSD 复发迹象。虽然疫苗诱发复发的概率较低，但需保持警惕。### 总结。在 NMOSD 用药期间，**灭活疫苗通常是安全且推荐的**，特别是流感和新冠疫苗，因为感染本身诱发 NMOSD 复发的风险远高于疫苗。但是，**减毒活疫苗通常是禁止的**。最关键的是**时机**和**药物种类**的匹配。请您携带目前的用药清单，我们在门诊为您制定个性化的疫苗接种计划。如果您有具体的用药名称或即将面临的旅行计划（涉及特殊疫苗），请随时告诉我，我会为您做进一步的分析。

**11、如何缓解NMOSD引起的长期神经性疼痛、肌肉痉挛或顽固性瘙痒？**

您好。我是您的眼科及神经免疫专科医师。视神经脊髓炎谱系疾病（NMOSD）是一种主要累及视神经和脊髓的自身免疫性中枢神经系统疾病。虽然急性期的治疗重点是抑制炎症和挽救视力/运动功能，但您提到的**长期神经性疼痛、肌肉痉挛**（痉挛状态），往往是疾病慢性期或后遗症期最困扰患者、严重影响生活质量的症状。这些症状通常源于脊髓损伤后的神经通路异常放电或抑制性神经元受损。针对这三种顽固症状，我们需要采取**多学科综合管理策略**，包括药物治疗、物理康复及必要的介入治疗。以下是专业的诊疗建议：### 1. 神经性疼痛的管理 (Neuropathic Pain)NMOSD 引起的疼痛通常为中枢性疼痛，表现为烧灼感、电击感或紧束感，常规止痛药（如布洛芬、对乙酰氨基酚）通常无效。*	**一线药物选择**：*	**钙通道α2-δ配体**：加巴喷丁（Gabapentin）或普瑞巴林（Pregabalin）。这是目前的首选药物，能有效抑制神经元过度兴奋。需从小剂量开始滴定，以减少头晕和嗜睡副作用。*	**三环类抗抑郁药 **(TCAs)：如阿米替林（Amitriptyline）。不仅调节情绪，对夜间神经痛有显著疗效。*	**SNRI 类抗抑郁药**：如度洛西汀（Duloxetine），适用于伴有焦虑或抑郁情绪的疼痛患者。*	**二线及难治性方案**：*	若单药效果不佳，可考虑联合用药（例如：普瑞巴林 + 度洛西汀）。*	对于极度顽固的疼痛，在严格监控下可短期使用低剂量阿片类药物（如曲马多），但需警惕成瘾性和便秘副作用。### 2. 肌肉痉挛与僵硬的管理 (Spasticity)脊髓病变导致的上运动神经元损伤会引起肌张力增高，导致肢体僵硬、抽搐和疼痛。*	**口服药物治疗**：*	**巴氯芬 **(Baclofen)：GABA-B 受体激动剂，是治疗痉挛的金标准药物。需注意突然停药可能引发反跳性高热和惊厥。*	**替扎尼定 **(Tizanidine)：α2-肾上腺素能激动剂，对夜间痉挛效果较好，但需监测肝功能。*	**丹曲林 **(Dantrolene)：直接作用于骨骼肌，适用于上述药物无效者，但需严密监测肝毒性。*	**局部治疗**：*	**肉毒毒素注射 **(Botulinum Toxin)：针对局限性、严重的肌肉痉挛（如屈肌痉挛），局部注射可显著缓解张力，效果可持续 3-4 个月。*	**侵入性治疗**（针对极重度病例）：*	**鞘内巴氯芬泵 **(Intrathecal Baclofen Pump)：对于口服药物无效或副作用无法耐受的患者，可通过手术植入泵，直接将微量药物注入蛛网膜下腔，疗效显著且全身副作用小。### 3. 顽固性瘙痒的管理 (Intractable Pruritus)NMOSD 相关的瘙痒属于“神经源性瘙痒”，并非由组胺释放引起，因此抗组胺药（如氯雷他定）通常无效。这种瘙痒往往剧烈且难以定位。*	**特异性药物**：*	**加巴喷丁/普瑞巴林**：同样作为一线选择，对神经源性瘙痒有确切疗效。*	**米氮平 **(Mirtazapine)：具有抗组胺和抗 5-HT 作用，对夜间瘙痒伴失眠者效果极佳。*	**纳曲酮 **(Naltrexone)：阿片受体拮抗剂，部分研究显示其对难治性瘙痒有效。*	**非药物干预**：*	保持皮肤湿润，使用无香料润肤霜，避免热水烫洗（会加重神经敏感）。*	冷敷或经皮神经电刺激 (TENS) 可能提供暂时缓解。### 4. 综合康复与生活管理。药物只是治疗的一部分，系统的康复训练至关重要：| 干预措施 | 具体执行方案 | 预期目标 || **物理治疗 **(PT) | 每日进行被动/主动拉伸训练，防止关节挛缩；水疗可利用浮力减轻痉挛。 | 维持关节活动度，减轻肌肉僵硬。 || **作业治疗 **(OT) | 定制支具（如踝足矫形器 AFO），辅助行走，改善手部精细动作。 | 提高日常生活自理能力。 || **心理支持** | 认知行为疗法 (CBT)，应对慢性疼痛带来的焦虑和抑郁。 | 打破“疼痛 - 焦虑 - 疼痛加重”的恶性循环。 || **生活方式** | 避免过热环境（Uhthoff 现象可能加重症状），规律作息，戒烟。 | 减少症状诱发因素。 |### 5. 重要提示与随访1. **个体化滴定**：上述药物均需遵循“小剂量起始，缓慢加量”的原则，找到疗效与副作用的最佳平衡点。2. **基础病控制**：请确保您正在接受规范的免疫抑制治疗（如利妥昔单抗、伊奈利珠单抗或萨特利珠单抗等），预防 NMOSD 复发是防止症状进一步恶化的根本。3. **警示信号**：如果出现新的视力下降、肢体无力加重或大小便功能障碍，请立即就医，这可能提示疾病复发而非单纯的后遗症波动。**总结流程图**：A[NMOSD 慢性症状评估] --> B{症状类型}B -->|神经性疼痛 | C[一线：加巴喷丁/普瑞巴林] B -->|肌肉痉挛 | D[一线：巴氯芬/替扎尼定] B -->|顽固瘙痒 | E[一线：加巴喷丁/米氮平]C --> F{效果评估}D --> E --> F -->|有效 | G[维持治疗 + 康复训练] F -->|无效/不耐受 | H[调整方案] H --> I[联合用药] H --> J[局部肉毒毒素注射] H --> K[鞘内泵植入评估]G --> L[定期随访 & 免疫治疗监控]作为您的医生，我理解这些症状带来的痛苦是漫长且煎熬的。请不要独自忍受，我们有很多手段可以帮您减轻负担。建议您携带目前的用药清单，我们在门诊详细讨论调整方案。*(注：以上内容仅供医疗参考，具体处方请务必在面诊后由主治医生开具。)*

**12、NMOSD导致的大小便功能障碍怎么办？**

您好。我是您的眼科及神经免疫专科医师。首先，我需要向您澄清一个非常重要的医学概念：**视神经脊髓炎谱系疾病（NMOSD）** 虽然常以严重的视神经炎（导致视力下降、失明）为首发或主要症状，但它本质上是一种累及中枢神经系统（脑和脊髓）的自身免疫性疾病。您提到的**大小便功能障碍**（尿潴留、尿失禁、便秘或大便失禁），通常不是由眼部病变直接引起的，而是由于 NMOSD 侵犯了**脊髓**（特别是圆锥或马尾神经区域），导致了神经源性膀胱和神经源性直肠。这在 NMOSD 患者中是非常常见且严重影响生活质量的并发症。作为医师，针对 NMOSD 导致的大小便功能障碍，我们采取的是“急性期控制 + 慢性期管理 + 康复训练”的综合治疗策略。以下是详细的诊疗建议：### 1. 急性期处理：控制炎症风暴。如果大小便障碍是近期突然加重或新发的，这往往提示疾病处于**急性复发期**。*	**大剂量激素冲击治疗**：通常使用甲泼尼龙静脉滴注，以迅速抑制脊髓炎症，减轻水肿，尽可能恢复神经功能。*	**血浆置换（PLEX）或免疫吸附**：如果激素治疗效果不佳，需尽早进行血浆置换，清除血液中的致病抗体（如 AQP4-IgG）。*	**目的**：此阶段的目标是“抢救”受损的神经纤维，防止永久性损伤。### 2. 泌尿系统管理（神经源性膀胱）这是护理的核心，目的是保护肾功能、预防尿路感染并改善生活质量。*	**评估与监测**：*	必须进行**尿流动力学检查**，明确膀胱是“痉挛性”（储尿困难）还是“弛缓性”（排尿困难）。*	定期监测残余尿量（PVR）。*	**间歇性导尿（CIC）**： *	对于尿潴留患者，**清洁间歇性导尿**是金标准。建议每 4-6 小时导尿一次，避免长期留置导尿管，以减少感染风险。*	**药物治疗**：*	**逼尿肌过度活动**（尿频、急迫性尿失禁）：可使用抗胆碱能药物（如索利那新、托特罗定）或 $\beta_3$受体激动剂（米拉贝隆）。*	**排尿困难**：可尝试 $\alpha$-受体阻滞剂（如坦索罗辛）松弛膀胱颈。*	**肉毒素注射**：对于药物无效的严重膀胱痉挛，可考虑膀胱壁注射 A 型肉毒毒素。### 3. 肠道管理（神经源性直肠）目标是建立规律的排便习惯，避免便秘和失禁。*	**饮食调整**：增加膳食纤维摄入（蔬菜、水果、全谷物），保证每日饮水量（若无心脏/肾脏禁忌，建议 1.5L-2L/天）。*	**排便训练**：*	利用“胃结肠反射”，建议在早餐后尝试排便。*	建立固定的排便时间表。*	**药物辅助**：*	使用容积性泻药（如欧车前）、渗透性泻药（如乳果糖、聚乙二醇）。 *	必要时使用开塞露或进行人工辅助排便（数字刺激）。*	**生物反馈治疗**：对于仍有部分括约肌功能的患者，可尝试生物反馈训练。### 4. 长期免疫抑制治疗（预防复发）NMOSD 是一种高复发率的疾病，每一次复发都可能导致残疾累积。预防复发是防止大小便功能进一步恶化的根本。*	**传统免疫抑制剂**：硫唑嘌呤、吗替麦考酚酯、环磷酰胺等。*	**生物制剂**：利妥昔单抗（抗 CD20）、伊奈利珠单抗、萨特利珠单抗等。*	**注意**：需严格遵医嘱长期服药，不可随意停药。### 5. 康复与生活护理*	**盆底肌训练**：在康复师指导下进行凯格尔运动（Kegel exercises），增强盆底肌肉力量。*	**皮肤护理**：对于失禁患者，务必保持会阴部皮肤清洁干燥，使用护臀霜，预防压疮（褥疮）和湿疹。*	**心理支持**：大小便障碍极易导致患者焦虑和抑郁，家属的支持和专业心理咨询非常重要。##医师特别警示1. **警惕尿路感染**：NMOSD 患者一旦发生尿路感染，极易诱发疾病复发（假性复发或真性复发）。若出现发热、尿液浑浊、腰痛，请立即就医。2. **肾功能保护**：长期的神经源性膀胱若不妥善处理，会导致肾积水甚至肾衰竭。请务必定期复查泌尿系 B 超和肾功能。3. **鉴别诊断**：虽然您已确诊 NMOSD，但若症状模式发生剧烈改变，需排除是否合并其他问题（如结石、前列腺问题等）。**总结建议**：大小便功能障碍是 NMOSD 脊髓受累的典型表现，虽然棘手，但通过规范的**间歇导尿、药物调节、肠道训练以及严格的免疫抑制治疗**，绝大多数患者的症状可以得到有效控制，生活质量可以显著改善。请您尽快前往医院的**神经内科**联合**泌尿外科**及**康复科**进行多学科会诊（MDT），制定个性化的治疗方案。不要因为难以启齿而延误治疗。祝您早日稳定病情，重获舒适生活。

**13、NMOSD病情稳定后，应该如何进行科学、安全的康复训练？**

您好，我是您的眼科医师。很高兴听到您的视神经脊髓炎谱系疾病（NMOSD）病情已经进入稳定期。这是一个非常关键的阶段，科学的康复训练不仅能帮助您恢复受损的视觉功能，还能提高生活质量，预防并发症。但在开始任何训练之前，我必须强调一个核心原则：**NMOSD 的康复必须个体化，且需在神经眼科医生和康复医师的共同指导下进行**。错误的训练强度或方式可能诱发疲劳，甚至增加复发风险。以下是针对 NMOSD 病情稳定期患者的科学、安全康复训练指南：### 一、康复前的必要评估。在启动训练计划前，我们需要确认以下指标：1. **疾病活动性确认**：确保至少 3-6 个月内无新发发作，MRI 检查无新发病灶，免疫抑制治疗方案稳定。2. **基线功能评估**：*	视力及视野检查（评估视神经损伤程度）。*	对比敏感度测试。*	眼球运动功能评估（是否存在复视或眼肌麻痹）。 *	全身体能状态（EDSS 评分参考）。### 二、视觉功能专项康复训练。针对 NMOSD 常见的视神经炎后遗症，我们可以采取以下策略：#### 1. 残余视力优化训练。如果存在视力下降但仍有光感或残余视力：*	**注视稳定性训练**：利用微视野计（Microperimetry）生物反馈技术，训练患者使用视网膜敏感区域进行注视。*	**扫视与追踪训练**：通过计算机辅助软件，练习眼球的快速跳动（扫视）和平滑追随运动，改善阅读困难和物体追踪能力。#### 2. 复视（双影）管理。若遗留眼肌麻痹导致复视：*	**棱镜适应训练**：在验配棱镜眼镜后，进行融合范围训练，扩大单视范围。*	**遮盖疗法交替**：对于暂时无法融合的严重复视，采用交替遮盖法防止抑制性弱视，同时减轻视觉混淆带来的头晕。#### 3. 低视力辅助器具适配。对于视力损伤较重者，尽早引入助视器：*	**光学助视器**：如高倍放大镜、望远镜式眼镜。*	**电子助视器**：利用对比度增强、字体放大功能。*	**环境改造**：增加照明亮度（NMOSD 患者常对强光敏感，需使用防眩光滤镜），使用高对比度的生活用品。### 三、全身性与生活方式康复。NMOSD 是一种中枢神经系统疾病，眼部症状往往伴随全身疲劳或肢体功能障碍。#### 1. 能量管理（Pacing Strategy）这是预防复发和过度疲劳的关键。*	**4P 原则**：Plan（计划）、Prioritize（优先排序）、Pace（节奏控制）、Position（体位调整）。*	**避免过热**：体温升高可能导致暂时性的症状加重（Uhthoff 现象）。训练环境温度应控制在 20- 24度，避免热水浴或高温瑜伽。#### 2. 适度有氧运动*	**推荐项目**：游泳（水温适宜）、快走、固定自行车。*	**强度控制**：心率控制在最大心率的 50%-60%*	**频率**：每周 3-4 次，每次 20-30 分钟，循序渐进。#### 3. 心理支持与认知康复。长期的视力障碍可能导致焦虑或抑郁，建议结合心理咨询，并进行必要的认知功能训练（如记忆力、注意力练习）。### 四、康复训练流程示例为了让您更直观地理解每日的训练安排，我为您制定了一个简化的流程图：A[开始每日康复] --> B{自我状态评估}B -- 疲劳/发热/不适 --> C[暂停训练，休息监测] B -- 状态良好 --> D[热身环节 5-10 分钟] D --> E[视觉专项训练 20 分钟] E --> F[全身有氧/平衡训练 20 分钟] F --> G[放松与冷身 10 分钟] G --> H{记录训练日志}H --> I[结束]注意事项J[保持环境凉爽]K[避免过度用眼]L[如有视力波动立即停止]### 五、重要警示信号（Red Flags）在康复过程中，如果出现以下情况，请**立即停止训练**并联系您的主治医生：1. **视力突然下降**或视野缺损扩大。2. 出现新的**复视**或眼球转动疼痛。3. 肢体无力、麻木感加重或大小便功能障碍。4. 持续的、无法通过休息缓解的极度疲劳。5. 发热或感染迹象（感染是 NMOSD 复发的常见诱因）。### 六、随访计划。康复不是一蹴而就的，我们需要动态调整方案：*	**初期**：每 4-6 周复查一次，评估训练效果及安全性。*	**稳定期**：每 3-6 个月进行一次全面的眼科及神经系统评估。*	**长期监测**：定期检查血清 AQP4-IgG 抗体滴度及补体水平。**医师寄语**：NMOSD 的康复是一场马拉松，而非短跑。请您保持耐心，严格遵医嘱用药（免疫抑制剂不可擅自停用），并在专业团队的陪伴下科学训练。您的每一次进步，都是我们共同的努力成果。如果您对上述任何环节有疑问，或需要具体的训练器械推荐，请随时告诉我。

**14、NMOSD能治愈吗？**

您好，我是您的眼科医师。关于您提出的**视神经脊髓炎谱系疾病**（NMOSD）能否治愈的问题，这是一个非常严肃且关键的医学话题。作为医生，我需要给您一个诚实、科学且负责任的回答。### 核心结论：目前尚无法“彻底治愈”，但可“有效控制”很遗憾地告诉您，以目前的医学水平，NMOSD **暂时无法被彻底治愈**（即无法通过一次治疗永久清除病因并不再复发）。它是一种慢性的、自身免疫性的中枢神经系统疾病。但是，"**不可治愈”绝不等于“无药可救**"。通过规范的长期管理，我们完全可以将病情控制在**临床缓解期**，极大程度地降低复发率，保护您的视力和肢体功能，使患者能够回归正常的生活和工作。### 为什么 NMOSD 难以治愈？NMOSD 的核心病理机制是体内产生了错误的抗体（主要是 **AQP4-IgG**），攻击视神经和脊髓中的水通道蛋白 -4。目前的医疗手段还无法从根本上“纠正”免疫系统的这种错误记忆，使其永远不再产生致病抗体。因此，治疗的重点在于**抑制异常免疫反应**和**预防复发**。### 我们的治疗策略：从“救火”到“防火”虽然不能根治，但我们有一套成熟的治疗方案，分为两个阶段：#### 1. 急性期治疗（救火）当疾病发作（如突发视力下降、肢体无力）时，目标是迅速抑制炎症，挽救受损的神经功能。*	**大剂量糖皮质激素冲击疗法**：首选方案。*	**血浆置换**（PLEX）或**免疫吸附**：用于激素效果不佳的重症患者。*	**静脉注射免疫球蛋白**（IVIG）。#### 2. 缓解期治疗（防火/预防复发）—— **这是终身管理的关键**NMOSD 具有高复发率的特点，每一次复发都可能导致不可逆的残疾（如失明、瘫痪）。因此，**长期维持治疗**是核心。近年来，靶向生物制剂的出现极大地改善了预后。以下是目前主流的治疗药物分类：| 药物类别 | 代表药物 | 作用机制 | 适用情况 || **B 细胞清除剂** | 利妥昔单抗 (Rituximab) | 清除产生抗体的 B 细胞 | 一线常用，疗效确切 || **IL-6 受体拮抗剂** | 萨特利珠单抗 (Satralizumab) | 阻断炎症因子 IL-6 通路 | 皮下注射，方便居家使用 || **补体 C5 抑制剂** | 依库珠单抗 (Eculizumab) | 阻断补体级联反应 | 针对难治性病例，起效快 || **传统免疫抑制剂** | 硫唑嘌呤、麦考酚钠 | 广谱抑制免疫系统 | 经济型选择，需监测副作用 |> **注**：具体用药方案需根据您的 AQP4 抗体状态、MOG 抗体状态、肝肾功能及经济状况个体化制定。### 给患者的预后展望。虽然我们需要长期服药，但请不要灰心。随着新型靶向药物的应用，NMOSD 的治疗格局已经发生了翻天覆地的变化：1. **复发率显著降低**：许多患者在使用新型生物制剂后，可以实现**零复发**。2. **生活质量提高**：只要不复发，神经功能通常可以保持稳定，不影响寿命和日常活动。3. **定期随访至关重要**：我们需要定期监测眼底 OCT、视野检查以及血清抗体滴度。### 医师建议1. **切勿自行停药**：这是导致复发的最常见原因。即使感觉良好，也必须遵医嘱维持治疗。2. **警惕复发先兆**：一旦出现视力模糊、眼球转动痛、肢体麻木无力或大小便障碍，请**立即**就医，争取黄金治疗时间。3. **生活方式管理**：避免过度劳累、感染（感冒、发烧常诱发复发）和精神压力过大。**总结来说**：NMOSD 目前虽不能像感冒一样“断根”，但它已经逐渐转变为一种像高血压、糖尿病一样的**可控慢性病**。只要我们医患配合，坚持规范治疗，您完全可以拥有光明的未来。如果您有具体的检查报告或正在考虑某种治疗方案，欢迎随时与我沟通，我们将为您制定最合适的个性化诊疗计划。

**15、NMOSD的长期预后怎么样？**

您好。我是您的眼科医师。关于您关心的**视神经脊髓炎谱系疾病（NMOSD）**的长期预后问题，这是一个非常严肃且需要个体化评估的话题。在过去，NMOSD 曾被认为是多发性硬化（MS）的一种亚型，但自从 2004 年发现水通道蛋白 -4 抗体（AQP4-IgG）以来，我们认识到它是一种独立的、通常比 MS 更具破坏性的自身免疫性疾病。其长期预后主要取决于**复发频率、急性期治疗的及时性以及是否进行了规范的长期免疫抑制治疗**。以下我将从视力预后、残疾累积、生存率及影响因素几个维度为您详细解读：### 1. 视力预后：最大的挑战。对于眼科患者而言，NMOSD 最显著的后果是**视神经炎（ON）**导致的视力损害。***复发与累积损伤**：NMOSD 具有高度的复发倾向。约 90% 的患者在病程中会经历复发。每一次视神经炎的发作都可能导致不可逆的视神经萎缩。*	单次严重发作即可导致单眼失明或重度视力下降。*	若未进行预防性治疗，约 50% 的患者在发病后 5 年内会出现双眼严重视力受损（最佳矫正视力低于 0.1）甚至失明。*	**恢复能力较差**：与多发性硬化相关的视神经炎相比，NMOSD 引起的视神经炎急性期过后的自然恢复能力较差。许多患者在急性期治疗后，视力只能部分恢复，遗留视野缺损或色觉异常。### 2. 运动功能与整体残疾。除了眼部症状，NMOSD 常累及脊髓，导致**横贯性脊髓炎**。*	**行走能力**：反复发作的脊髓炎会导致下肢无力、感觉障碍和括约肌功能障碍（大小便失禁）。据统计，未经有效治疗的患者中，约三分之一在发病后 5-10 年内需要依靠轮椅辅助行走。*	**EDSS 评分进展**：在扩展残疾状态量表（EDSS）上，NMOSD 患者的评分上升速度通常快于 MS 患者，主要是因为严重的单次发作即可导致残疾阶梯式跳跃，而非缓慢进展。### 3. 生存率与死亡率*	**历史数据 vs. 现代数据**：在免疫抑制疗法普及之前，NMOSD 的致死率较高，主要死因是高位颈髓病变导致的呼吸衰竭或严重的并发症（如感染）。*	**当前状况**：随着利妥昔单抗（Rituximab）、伊奈利珠单抗（Ineblizumab）、萨特利珠单抗（Satralizumab）等靶向生物制剂的应用，患者的长期生存率已显著提高，接近普通人群。目前的死亡风险主要来自于严重的急性发作处理不当或长期免疫抑制带来的机会性感染。### 4. 影响预后的关键因素。为了让您更直观地理解哪些因素决定了病情的走向，我整理了以下关键变量：| 影响因素 | 对预后的负面影响 | 对预后的正面影响 || **抗体状态** | AQP4-IgG 阳性且滴度高 | MOG-IgG 阳性（通常预后优于 AQP4+）或血清阴性 || **治疗依从性** | 未使用预防性免疫抑制剂，或随意停药 | 规范使用一线生物制剂或免疫抑制剂，定期随访 || **发作处理** | 急性期激素冲击治疗延迟 >48 小时 | 发病早期立即进行大剂量激素冲击或血浆置换 || **种族/性别** | 非白人种族（亚洲、非洲裔发病率及严重程度较高） | 早期诊断并干预 || **初始发作 severity** | 首发即为重症视神经炎或长节段脊髓炎 | 首发症状轻微且完全恢复 |### 5. 给患者的专业建议。作为您的医生，针对 NMOSD 的长期管理，我必须强调以下几点：1. **“预防重于治疗”**：NMOSD 的核心策略是**防止复发**。一旦确诊（特别是 AQP4 抗体阳性），必须尽早启动长期的免疫抑制治疗。传统的硫唑嘌呤、吗替麦考酚酯，以及新型的补体抑制剂或 IL-6 受体拮抗剂都能显著降低复发率。2. **避免误诊误治**：切勿将 NMOSD 当作多发性硬化（MS）治疗。某些用于 MS 的药物（如干扰素$\beta$、芬戈莫德）可能会**加重**NMOSD 的病情。3. **定期监测**：*	**眼科检查**：每 3-6 个月进行一次 OCT（光学相干断层扫描）和视野检查，监测视网膜神经纤维层（RNFL）厚度变化。*	**影像学**：定期复查头颅及全脊髓 MRI。4. **生活方式**：避免过度劳累、感染和极端温度变化，这些可能是复发的诱因。### 总结。虽然 NMOSD 是一种潜在的致残性疾病，特别是在视力方面威胁巨大，但**它不再是“绝症”**。只要您能坚持规范的长期免疫治疗，绝大多数患者可以控制复发，保留有用的视力，并维持良好的生活质量。请务必与您的神经内科和眼科医生保持紧密合作，制定个性化的治疗方案。如果您有关于具体药物选择或近期症状的疑问，欢迎随时向我提问。

**16、NMOSD患者可以怀孕生子吗？**

您好，我是您的眼科及神经免疫专科医师。非常理解您作为 NMOSD（视神经脊髓炎谱系疾病）患者对于生育问题的关切与担忧。**简短的回答是：是的，绝大多数 NMOSD 患者在病情稳定、经过严密评估和科学管理的前提下，是可以怀孕并生下健康宝宝的。**但是，NMOSD 是一种高复发风险的自身免疫性疾病，妊娠过程确实伴随着特殊的挑战，尤其是**产后复发风险显著增加**。因此，这绝不是一个“能不能”的简单问题，而是一个需要神经内科、产科、新生儿科甚至眼科医生共同参与的“如何安全进行”的系统工程。以下我将从专业角度为您详细拆解关键的风险点与管理策略：### 1. 核心风险评估。在考虑怀孕前，我们必须清醒地认识到以下几个关键事实：*	**妊娠期风险相对较低**：研究表明，在妊娠期间（尤其是中晚期），由于体内激素水平变化（如雌激素升高、免疫耐受增强），NMOSD 的复发率通常较低，甚至低于非妊娠期。*	**产后复发风险极高**：这是最大的风险窗口。**产后 3-6 个月**是复发的高峰期，约有 20%-30% 的患者会在这一时期经历复发。复发可能导致严重的视力下降（视神经炎）或肢体瘫痪（脊髓炎）。*	**药物致畸性**：许多用于维持治疗或预防复发的免疫抑制剂（如吗替麦考酚酯、甲氨蝶呤、环磷酰胺等）具有明确的致畸性，必须在备孕前足够长的时间停药或替换。### 2. 备孕前的关键准备（Pre-conception Counseling）在您尝试受孕之前，必须满足以下条件并与医生制定详细计划：#### A. 病情稳定性评估*	**无复发期**：通常建议在最后一次复发后至少 **6-12 个月** 内无新发症状，且病情处于稳定缓解期。*	**抗体状态**：确认 AQP4-IgG（水通道蛋白 4 抗体）的状态。阳性患者复发风险更高，需更严格的监控。#### B. 药物调整方案：这是最关键的一步。您需要将致畸药物转换为妊娠安全性较高的药物。| 药物类别 | 常见药物 | 妊娠期安全性 | 备孕建议 || **绝对禁忌** | 吗替麦考酚酯 (MMF), 甲氨蝶呤 (MTX), 环磷酰胺 (CTX) | **致畸** | 必须提前停药（通常 MMF 需停 6 周以上，MTX 需停 3-6 个月），并在医生指导下切换药物。 || **相对安全/可用** | 硫唑嘌呤 (AZA), 他克莫司 | **较安全** | 可在整个孕期使用，需监测血药浓度和肝功能。 || **生物制剂** | 利妥昔单抗 (Rituximab) | **需谨慎** | 通常在孕前单次使用以维持长期缓解，或在孕早期使用。需权衡利弊。 || **急性期治疗** | 大剂量甲泼尼龙，血浆置换 (PLEX) | **可用** | 若孕期复发，可作为急救手段。 |#### C. 多学科会诊 (MDT)在备孕前，请务必联合**神经内科医生**（主导）、**高危产科医生**和**眼科医生**进行一次全面评估。### 3. 妊娠期管理策略。一旦成功受孕，管理重点在于“监测”与“预防”：1. **密切随访**：产检频率应高于普通孕妇。除了常规产科检查外，需每月或每季度进行神经系统查体。2. **眼科专项监测**：*	由于 NMOSD 常累及视神经，建议每 trimester（三个月）进行一次详细的眼科检查，包括视力、视野、色觉及 OCT（光学相干断层扫描）。*	若出现视力模糊、眼球转动痛或视野缺损，需立即就医，排除视神经炎复发。3. **分娩方式**：*	NMOSD 本身不是剖宫产的绝对指征。若无产科并发症，**阴道自然分娩**通常是首选。*	但若患者存在严重的脊髓损伤导致下肢无力或感觉障碍，可能会影响第二产程，此时需产科医生评估是否辅助分娩或剖宫产。### 4. 产后高危期管理（重中之重）如前所述，产后是复发的高危期，必须采取主动防御措施：*	**预防性治疗**：对于高风险患者（如 AQP4 阳性、既往复发频繁），医生可能会建议在分娩后立即恢复使用免疫抑制剂（如硫唑嘌呤）或在产后尽早使用利妥昔单抗。*	**母乳喂养与用药的平衡**：*	部分药物（如硫唑嘌呤、低剂量激素）在哺乳期可能是安全的。*	但如果您需要使用强效免疫抑制剂或生物制剂，可能需要暂停母乳喂养。*	**决策原则**：母亲的健康是第一位的。如果为了喂奶而停用必要的预防药物导致失明或瘫痪，对患者和家庭都是巨大的打击。请与医生讨论“药物 - 哺乳”的最佳平衡点。### 5. 给患者的特别建议流程图。为了让您更清晰地理解整个过程，我为您梳理了以下的管理流程：A[NMOSD 患者有生育意愿] --> B{病情是否稳定？近 6-12 个月无复发}B -- 否 --> C[继续强化治疗，推迟备孕计划] B -- 是 --> D[多学科会诊 MDT，神经科 + 产科 + 眼科]D --> E{当前用药是否致畸？}E -- 是 (如 MMF, MTX) --> F[清洗期：停药并切换至，妊娠安全药物 (如 AZA) E -- 否/已切换 --> G[开始备孕]G --> H[妊娠期监测；定期神经/眼科检查]H --> I{是否出现复发迹象？}I -- 是 --> J[紧急干预：激素冲击/血浆置换] I -- 否 --> K[顺利分娩]K --> L[产后高危期管理；立即重启/调整免疫治疗]L --> M{是否母乳喂养？} M -- compatible 药物 --> N[可母乳喂养] M -- incompatible 药物 --> O[暂停母乳优先保障母亲健康]### 总结。作为医师，我的建议是：**不要因恐惧而放弃做母亲的权利，也不要因盲目乐观而忽视风险。**NMOSD 患者怀孕生子是一条需要精心规划的道路。只要您做到：1. **计划妊娠**（绝不意外怀孕）；2. **规范换药**（提前清除致畸因素）；3. **严密监测**（特别是产后）；您完全有机会迎来一个健康的宝宝，同时保全自己的视力和身体功能。请您尽快预约您的主治医生，开启备孕前的评估程序。祝您早日如愿，母子平安！

**17、孕期和产后如何管理疾病，以降低NMOSD复发风险？**

您好。我是您的眼科及神经眼科专科医师。视神经脊髓炎谱系疾病（NMOSD）是一种主要累及视神经和脊髓的自身免疫性疾病。对于女性患者而言，**孕期和产褥期**（产后）。由于 NMOSD 与多发性硬化（MS）不同，其在产后复发风险显著增加，且发作可能导致不可逆的视力丧失或肢体瘫痪，因此需要极其严密的“全周期管理”。以下是基于当前临床指南的专业建议：### 一、核心原则：风险分层与多学科协作。在备孕前，我们必须进行严格的风险评估。NMOSD 的管理不仅仅是眼科或神经内科的事，需要**神经内科、产科、新生儿科和眼科**的共同协作（MDT）。*	**抗体状态确认**：确认 AQP4-IgG（水通道蛋白 4 抗体）状态。阳性患者复发风险极高，需更积极的干预。*	**疾病活动度评估**：建议在病情稳定至少 6-12 个月后再考虑受孕。### 二、孕期管理策略。孕期免疫耐受机制通常会使某些自身免疫病缓解，但 NMOSD 的表现较为特殊，部分患者在孕中晚期仍可能复发。#### 1. 药物调整原则。我们需要在“控制母体疾病”和“保障胎儿安全”之间寻找平衡。| 药物类别 | 具体药物 | 孕期建议 | 备注 || **急性期治疗** | 大剂量甲泼尼龙 | **可用** | 若孕期复发，首选冲击治疗。虽然可通过胎盘，但短期使用相对安全。 || **维持治疗** | 硫唑嘌呤 (AZA) | **谨慎/可用** | 部分指南认为可继续使用，但需监测肝功能及血常规；也有观点建议孕前停药。 || **维持治疗** | 利妥昔单抗 (RTX) | **计划性停药** | 建议在受孕前 6-12 个月停用最后一剂，以清除 B 细胞并让药物代谢，同时维持孕期低复发率。 || **维持治疗** | 吗替麦考酚酯 (MMF) | **禁用** | 具有明确致畸性，必须在孕前至少 6 周停用并转换为安全药物。 || **维持治疗** | 依库珠单抗 | **数据有限** | 仅在获益明显大于风险时由专家会诊决定。 |#### 2. 监测频率*	**眼科随访**：每 3 个月进行一次详细的眼科检查，包括视力、视野、色觉及光学相干断层扫描（OCT），监测视网膜神经纤维层（RNFL）厚度变化。*	**神经系统评估**：任何新的感觉异常、无力或视力下降都应立即就医，不要等待常规复诊。### 三、产褥期（产后）管理：最关键的时刻**这是 NMOSD 患者最危险的时期**。产后免疫系统“反弹”，加上激素水平剧烈波动，导致复发风险急剧升高。据统计，产后 3 个月内的复发率可高达 20%-30%。#### 1. 预防性治疗方案。我们强烈建议在分娩前就制定好产后立即启动的治疗计划：*	**方案 A**（重启生物制剂）：如果孕前使用的是利妥昔单抗（RTX），可在分娩后即刻（通常在产后 1-2 周内，甚至出院前）重新给药。RTX 通过乳汁分泌极少，通常被认为兼容母乳喂养（需权衡利弊）。*	**方案 B**（小剂量激素桥接）：在重启长期免疫抑制剂之前，可使用口服泼尼松（如 $10-20 \text{mg/day}$）作为过渡，持续数周至数月，直到维持药物起效。*	**方案 C**（静脉注射免疫球蛋白 IVIG）：对于不适合使用上述药物的患者，可考虑定期输注 IVIG。#### 2. 母乳喂养与用药的权衡。这是一个复杂的决策过程，公式化表达我们的决策逻辑如下：*	**一般建议**：鉴于 NMOSD 复发可能导致永久性失明或截瘫，**优先保证母亲不复发**通常是第一原则。*	**药物兼容性**：*	**利妥昔单抗**：分子量大，进入乳汁量极微，且婴儿肠道吸收率低，多数专家认为可哺乳。*	**硫唑嘌呤**：代谢产物进入乳汁量极低，通常认为可以哺乳。*	**吗替麦考酚酯**：**严禁**哺乳期使用。### 四、紧急情况应对流程图。若您在孕期或产后出现视力模糊、眼球转动痛或肢体麻木，请立即遵循以下流程：A[出现疑似复发症状] --> B{症状评估}B -- 视力下降/视野缺损 --> C[立即前往急诊/神经眼科]B -- 肢体无力/感觉异常 --> C --> D[紧急 MRI 检查/脑/脊髓/视神经] D --> E[确诊复发] E --> F[启动急性期治疗] F --> G[大剂量甲泼尼龙冲击<br>500-1000mg/d × 3-5 天] G --> H{疗效评估} H -- 有效 --> I[逐渐减量激素 + 调整维持方案] H -- 无效/重症 --> J[血浆置换 PLEX<br>或 免疫吸附] J --> I### 五、给患者的特别叮嘱。1. **切勿自行停药**：很多患者因担心药物影响胎儿而自行停用免疫抑制剂，这往往是导致孕期或产后严重复发的主要原因。2. **感染预防**：孕期和产后免疫力波动，易发生感染（如尿路感染、呼吸道感染），感染是诱发 NMOSD 复发的重要诱因。请注意个人卫生，必要时接种灭活疫苗（避免减毒活疫苗）。3. **心理支持**：焦虑和抑郁也会干扰免疫系统。请保持与家人、医生的沟通，必要时寻求心理干预。**总结**：NMOSD 患者完全可以拥有健康的宝宝，但前提是**“计划妊娠”**和**“严密监控”**。请务必在孕前与我们制定详细的用药转换计划，并在产后第一时间重启预防性治疗。如果您有具体的用药史或目前的身体状况描述，请告诉我，我可以为您制定更个性化的建议。

**18、NMOSD会遗传给下一代吗？**

您好，我是您的眼科医师。关于您关心的**视神经脊髓炎谱系疾病**（NMOSD）是否会遗传给下一代的问题，这是一个非常专业且许多患者家庭都高度关注的话题。为了给您最准确的解答，我们需要从疾病的发病机制、遗传模式以及风险评估几个维度来详细分析。### 1. 核心结论：NMOSD 不是典型的“遗传病”。首先，请您放心，**NMOSD 不属于单基因遗传病**（如红绿色盲或血友病那样由单一基因突变直接决定并遵循孟德尔遗传规律）。*	**非直接遗传**：父母患有 NMOSD，并不意味着孩子一定会患病。*	**多因素致病**：目前医学界公认，NMOSD 是一种**自身免疫性疾病**。它的发生是**遗传易感性**（基因背景）、**环境因素**（如病毒感染、维生素 D 缺乏等）以及**免疫系统异常**共同作用的结果。### 2. 遗传易感性与 HLA 基因。虽然 NMOSD 不会直接“传染”或“必然遗传”，但确实存在**家族聚集现象**和**遗传易感性**。*	**HLA 基因关联**：研究发现，某些人类白细胞抗原（HLA）基因型与 NMOSD 的发病风险相关。例如，在亚洲人群中，$HLA-DPB1^*05:01$ 等位基因与 AQP4 抗体阳性的 NMOSD 有显著相关性。*	**风险传递**：如果父母携带这些特定的易感基因，子女继承这些基因的概率会增加，从而使得子女相对于普通人群，患病的**基础风险略高**。*	**数据参考**：据统计，NMOSD 患者的一级亲属（父母、子女、兄弟姐妹）患病风险约为普通人群的几倍到十几倍，但绝对风险值仍然很低（通常低于 5%）。这意味着，绝大多数患者的子女并不会发病。### 3. AQP4 抗体与 MOG 抗体的区别。NMOSD 主要分为两类，其遗传背景略有不同：1. **AQP4-IgG 阳性 NMOSD**：这是最常见的类型，与特定的 HLA 基因型关联较强，具有一定的家族易感倾向。2. **MOG-IgG 阳性疾病**（MOGAD）：部分曾被归类为 NMOSD 的患者实为 MOG 抗体相关疾病。这类疾病的遗传模式尚不完全明确，但同样不被视为直接遗传病。### 4. 给患者的建议与风险评估。作为医师，针对您是否有生育计划或对孩子的担忧，我给出以下专业建议：#### 风险评估表| 因素 | 说明 | 对后代的影响 || **直接遗传** | 单基因突变导致 | **无**。NMOSD 不符合此模式。 || **易感基因** | HLA 等位基因携带 | **轻微增加**。子女携带易感基因概率增加，但需环境触发才发病。 || **环境触发** | 感染、压力、光照等 | **关键因素**。即使有易感基因，若无环境触发，通常不发病。 || **总体概率** | 综合统计 | **低**。绝大多数子女终身健康。 |#### 临床指导1. **无需过度恐慌**：目前的证据表明，NMOSD 患者完全可以拥有健康的宝宝。疾病的遗传概率远低于许多常见的慢性病患者家庭的担忧。2. **孕前咨询**：如果您正在备孕，建议进行详细的孕前咨询。重点在于评估您目前的病情是否稳定（通常建议在缓解期备孕），以及调整用药方案（某些免疫抑制剂在孕期需停用或替换）。3. **新生儿监测**：孩子出生后，不需要进行特殊的基因筛查（因为目前没有单一的预测基因）。只需像普通儿童一样正常养育，注意避免严重的病毒感染，保持健康的生活方式即可。4. **关注症状**：虽然概率低，但如果孩子未来出现不明原因的视力下降、肢体麻木或无力，应及时就医并告知医生家族病史，以便早期排查。### 总结。NMOSD **不会**像色盲那样直接遗传给下一代。它只是将一种“更容易生病的体质（易感性）”传给了孩子，而是否真正发病，很大程度上取决于后天的环境因素和免疫状态。**绝大多数 NMOSD 患者的子女都是健康的**。请您保持乐观的心态，规范治疗，控制好自身的病情，这对您和未来的宝宝都是最好的保护。如果您有具体的备孕计划或需要调整药物，请务必前往神经内科或眼科专科门诊进行面对面的详细评估。

**19、NMOSD有什么饮食禁忌？**

您好，我是您的眼科医师。针对您关心的**视神经脊髓炎谱系疾病**（NMOSD）的饮食禁忌问题，我需要首先向您说明一个核心概念：目前医学界**并没有针对 NMOSD 本身的绝对“饮食禁忌”**（即吃了就会直接导致病情复发的特定食物）。NMOSD 是一种自身免疫性疾病，其发病机制主要与体内产生的水通道蛋白 -4 抗体（AQP4-IgG）有关，而非由食物过敏或摄入直接触发。然而，作为临床医生，我非常强调"**治疗相关性饮食管理**"。因为 NMOSD 的治疗通常需要长期使用免疫抑制剂（如利妥昔单抗、硫唑嘌呤、吗替麦考酚酯等）或激素（如泼尼松），这些药物会带来特定的副作用。因此，我们的饮食调整主要是为了**对抗药物副作用**、**维持免疫系统平衡**以及**预防并发症**。以下是基于临床经验的详细饮食指导：### 1. 核心原则：没有“忌口”，只有“优选”请不要盲目相信民间流传的“发物”说法（如牛羊肉、海鲜等），除非您个人对这些食物明确过敏。盲目忌口可能导致营养不良，反而降低身体抵抗力，增加感染风险，而**感染**才是诱发 NMOSD 复发的最常见因素之一。### 2. 针对不同治疗阶段的饮食建议#### A. 如果您正在使用糖皮质激素（如泼尼松）激素治疗容易引起血糖升高、血压升高、骨质疏松和水钠潴留。*	**低盐饮食**：每日食盐摄入量建议控制在 $<5\text{g}$。避免腌制食品（咸菜、腊肉），以减轻水肿和高血压风险。*	**低糖饮食**：减少精制碳水化合物（甜点、含糖饮料、白米饭过量），预防类固醇性糖尿病。*	**高钙与维生素 D**：激素会加速钙流失。*	多食用：牛奶、酸奶、深绿色蔬菜、豆制品。*	必要时遵医嘱补充钙剂和维生素 D3，预防骨质疏松。*	数学表达骨骼健康指标参考：骨密度 T 值应维持在 $T \ge -1.0$ 为正常范围。#### B. 如果您正在使用免疫抑制剂

这类药物会抑制免疫系统，使您更容易受到细菌、真菌和病毒的感染。*	**食品安全第一**（低菌饮食）：*	**严禁**食用生食或未煮熟的食物（如生鱼片、半熟牛排、生鸡蛋、未巴氏消毒的牛奶）。*	水果必须削皮后食用，避免食用难以清洗彻底的莓果类（除非充分加热或确认来源安全）。*	剩饭剩菜必须彻底加热，最好避免食用隔夜菜。*	**避免葡萄柚**（西柚）：如果您服用某些特定的免疫抑制剂（如环孢素、他克莫司等），葡萄柚中的呋喃香豆素会抑制肝脏细胞色素 P450 酶系（具体为 $CYP3A4$），导致药物血药浓度异常升高，增加毒性风险。#### C. 针对视神经保护的通用建议。虽然饮食不能治愈视神经损伤，但良好的微循环和抗氧化环境有助于神经修复。*	**富含抗氧化剂的食物**：深色蔬菜（菠菜、西兰花）、浆果（蓝莓）、坚果。*	**Omega-3 脂肪酸**：深海鱼类（三文鱼、鲭鱼），有助于抗炎。*	**B 族维生素**：特别是维生素 B12 和叶酸，对神经系统健康至关重要。### 3. 需要警惕的“伪禁忌”与误区| 误区类型 | 常见说法 | 医师专业解读 || **盲目忌口** | “不能吃鸡肉、鸡蛋、海鲜，否则复发” | **错误**。优质蛋白是修复组织的基础。除非过敏，否则应正常摄入。 || **过度进补** | “多吃人参、灵芝提高免疫力” | **风险**。NMOSD 是免疫“过度活跃”攻击自身，盲目使用增强免疫的补品可能理论上存在激惹免疫系统的风险，需咨询主治医生。 || **完全素食** | “吃素能排毒治病” | **不推荐**。长期纯素可能导致维生素 B12 缺乏，加重神经病变。 |### 4. 总结与建议流程图。为了帮助您更好地执行饮食管理，我为您梳理了以下决策流程： A[NMOSD 患者饮食决策] --> B{是否处于急性发作期？}B -- 是 --> C[清淡易消化，保证充足水分，严格食品卫生] B -- 否/维持期 --> D{当前主要用药是什么？}D -- 激素治疗 --> E[低盐，低糖，高钙 +VitD] D -- 免疫抑制剂 --> F[严禁生食，避免葡萄柚，注意食品卫生] D -- 无特殊用药 --> G[均衡饮食，地中海饮食模式] E --> H[定期监测血糖/血压/骨密度] F --> I[定期监测血常规/肝肾功能] G --> J[保持健康体重，适度运动] H --> K[预防感染，避免复发]### 医师寄语：对于 NMOSD 患者而言，**预防感染**比任何单一的饮食禁忌都重要。请保持均衡的营养摄入，维持良好的身体状态去应对长期的药物治疗。如果您有具体的合并症（如糖尿病、高血压）或对某种食物有疑虑，请务必在复诊时告知我，我们会根据您的具体生化指标（如血糖、血钙等）制定个性化的方案。祝您病情稳定，视力健康！

**20、面对NMOSD高复发风险和高致残率，我感到非常焦虑和抑郁，该怎么办？**

您好。作为一名眼科医师，我非常理解您此刻的心情。视神经脊髓炎谱系疾病（NMOSD）确实是一种具有挑战性的自身免疫性疾病，尤其是当它累及视神经时，会对视力造成威胁。面对“高复发风险”和“高致残率”这些冷冰冰的医学统计数据，产生焦虑、恐惧甚至抑郁的情绪，是**非常正常且普遍的反应**。这并不代表您脆弱，而是说明您非常在乎自己的健康和未来。请记住，医学在不断进步，现在的诊疗手段已经与十年前大不相同。我们不仅要治疗眼睛，更要治疗“患病的人”。以下是我为您制定的身心综合应对策略：### 1. 认知重构：用科学数据对抗恐惧。焦虑往往源于对未知的恐惧和对旧有信息的刻板印象。我们需要更新您对 NMOSD 的认知：*	**治疗手段的革命性突破**：过去 NMOSD 确实缺乏特效药，但近年来，针对 B 细胞（如利妥昔单抗）、补体系统（如依库珠单抗）以及 IL-6 受体（如萨特利珠单抗）的靶向生物制剂已经广泛应用。*	临床数据显示，规范使用新型免疫抑制剂或生物制剂，可以将年复发率降低 **70% - 90%** 以上。*	这意味着，只要规范治疗，绝大多数患者可以实现长期无复发生存，从而避免残疾累积。*	**“高致残率”的前提是“反复发作”**：NMOSD 的致残性主要来源于多次复发后的累积效应。如果我们通过药物成功阻止了复发，那么“高致残率”这个统计概率在您身上就不会发生。**预防复发就是保护视力，就是防止残疾。**### 2. 医疗行动：建立坚固的防线。作为您的医生，我建议您立即采取以下行动，将主动权掌握在自己手中：*	**严格遵医嘱用药**：这是控制病情的基石。切勿自行减量或停药。*	**定期监测指标**： *	定期进行眼底检查、OCT（光学相干断层扫描）和视野检查，监控视神经状态。*	监测免疫指标（如 AQP4 抗体滴度、淋巴细胞亚群等），评估药物疗效。*	**制定应急预案**：与我们约定好，一旦出现视力下降、眼球转动痛等疑似复发征兆，应在 **24-48 小时内** 就医。早期大剂量激素冲击治疗可以最大程度挽救视力。### 3. 心理干预：正视并处理情绪问题。焦虑和抑郁本身会降低免疫系统的稳定性，可能间接影响病情。因此，心理治疗是 NMOSD 整体治疗方案中不可或缺的一部分。*	**寻求专业心理支持**：如果您的焦虑和抑郁情绪已经影响到睡眠、食欲或日常生活，请务必咨询精神科医生或心理治疗师。必要时，短期使用抗焦虑或抗抑郁药物是安全且有效的辅助手段。*	**正念与放松训练**：*	尝试每日进行 10-15 分钟的腹式呼吸或正念冥想，有助于降低皮质醇水平，缓解紧张情绪。*	您可以参考以下的放松流程： A[感到焦虑/恐慌] --> B{暂停当前活动}B --> C[深呼吸：吸气 4 秒] C --> D[屏气：保持 4 秒] D --> E[呼气：缓慢 6 秒] E --> F[重复 5-10 次] F --> G[评估情绪变化] G -->|仍紧张 | H[进行肌肉渐进式放松]G -->|缓解 | I[回归日常] H --> I*	**加入病友支持团体**：与同样患有 NMOSD 但生活良好的病友交流，看到他们正常工作、生活的例子，是打破“必残”诅咒最有力的证据。### 4. 生活方式管理：做自己健康的 CEO。除了药物，生活方式的调整也能显著改善预后：*	**避免诱因**：感染、过度疲劳、高温环境都可能诱发复发。请注意保暖，预防感冒，避免桑拿或长时间暴晒。*	**规律作息**：保证充足的睡眠，避免熬夜。*	**适度运动**：在体力允许的情况下，进行散步、瑜伽等温和运动，有助于分泌内啡肽，改善心情。### 医师寄语。我想对您说：**NMOSD 是一场马拉松，而不是短跑。** 虽然路途充满挑战，但您绝不是独自在奔跑。我们有先进的药物作为盾牌，有专业的医疗团队作为后盾，更有您自己强大的求生欲作为动力。目前的焦虑是对未来的预警，它在提醒您要重视治疗，但请不要让它吞噬您的现在。请把关注点从“万一复发了怎么办”转移到“今天我如何更好地照顾自己”上来。如果您愿意，我们可以一起制定一个详细的随访计划表，让您对未来的每一步都清晰可见，从而减少不确定性带来的恐慌。请相信，只要控制得当，您依然可以拥有明亮的世界和精彩的人生。如有任何眼部不适或情绪波动，随时联系我。
